# Telomere-to-telomere gap-free genome assembly of the endangered Yangtze finless porpoise and East Asian finless porpoise

**DOI:** 10.1093/gigascience/giae067

**Published:** 2024-09-16

**Authors:** Denghua Yin, Chunhai Chen, Danqing Lin, Zhong Hua, Congping Ying, Jialu Zhang, Chenxi Zhao, Yan Liu, Zhichen Cao, Han Zhang, Chenhe Wang, Liping Liang, Pao Xu, Jianbo Jian, Kai Liu

**Affiliations:** Key Laboratory of Freshwater Fisheries and Germplasm Resources Utilization, Ministry of Agriculture and Rural Affairs, Freshwater Fisheries Research Center, Chinese Academy of Fishery Sciences, Wuxi 214081, China; BGI Genomics, Shenzhen 518083, China; Key Laboratory of Freshwater Fisheries and Germplasm Resources Utilization, Ministry of Agriculture and Rural Affairs, Freshwater Fisheries Research Center, Chinese Academy of Fishery Sciences, Wuxi 214081, China; Key Laboratory of Freshwater Fisheries and Germplasm Resources Utilization, Ministry of Agriculture and Rural Affairs, Freshwater Fisheries Research Center, Chinese Academy of Fishery Sciences, Wuxi 214081, China; Key Laboratory of Freshwater Fisheries and Germplasm Resources Utilization, Ministry of Agriculture and Rural Affairs, Freshwater Fisheries Research Center, Chinese Academy of Fishery Sciences, Wuxi 214081, China; Key Laboratory of Freshwater Fisheries and Germplasm Resources Utilization, Ministry of Agriculture and Rural Affairs, Freshwater Fisheries Research Center, Chinese Academy of Fishery Sciences, Wuxi 214081, China; BGI Genomics, Shenzhen 518083, China; Key Laboratory of Freshwater Fisheries and Germplasm Resources Utilization, Ministry of Agriculture and Rural Affairs, Freshwater Fisheries Research Center, Chinese Academy of Fishery Sciences, Wuxi 214081, China; National Demonstration Center for Experimental Fisheries Science Education, Shanghai Ocean University, Shanghai 201306, China; National Demonstration Center for Experimental Fisheries Science Education, Shanghai Ocean University, Shanghai 201306, China; BGI Genomics, Shenzhen 518083, China; BGI Genomics, Shenzhen 518083, China; Key Laboratory of Freshwater Fisheries and Germplasm Resources Utilization, Ministry of Agriculture and Rural Affairs, Freshwater Fisheries Research Center, Chinese Academy of Fishery Sciences, Wuxi 214081, China; Wuxi Fisheries College, Nanjing Agricultural University, Wuxi 214081, China; BGI Genomics, Shenzhen 518083, China; Guangdong Provincial Key Laboratory of Marine Biotechnology, Shantou University, Shantou 515063, China; Key Laboratory of Freshwater Fisheries and Germplasm Resources Utilization, Ministry of Agriculture and Rural Affairs, Freshwater Fisheries Research Center, Chinese Academy of Fishery Sciences, Wuxi 214081, China; Wuxi Fisheries College, Nanjing Agricultural University, Wuxi 214081, China; National Demonstration Center for Experimental Fisheries Science Education, Shanghai Ocean University, Shanghai 201306, China

**Keywords:** telomere-to-telomere, genome assembly, Yangtze finless porpoise, gap-free, HiFi sequencing, Hi-C sequencing

## Abstract

**Background:**

The Yangtze finless porpoise (*Neophocaena asiaeorientalis asiaeorientalis*, YFP) and the East Asian finless porpoise (*Neophocaena asiaeorientalis sunameri*, EFP) are 2 subspecies of the narrow-ridged finless porpoise that live in freshwater and saltwater, respectively. The main objective of this study was to provide contiguous chromosome-level genome assemblies for YFP and EFP.

**Results:**

Here, we generated and upgraded the genomes of YFP and EFP at the telomere-to-telomere level through the integration of PacBio HiFi long reads, ultra-long ONT reads, and Hi-C sequencing data with a total size of 2.48 Gb and 2.50 Gb, respectively. The scaffold N50 of 2 genomes was 125.12 Mb (YFP) and 128 Mb (EFP) with 1 contig for 1 chromosome. The telomere repeat and centromere position were clearly identified in both YFP and EFP genomes. In total, 5,480 newfound genes were detected in the YFP genome, including 56 genes located in the newly identified centromere regions. Additionally, synteny blocks, structural similarities, phylogenetic relationships, gene family expansion, and inference of selection were studied in connection with the genomes of other related mammals.

**Conclusions:**

Our research findings provide evidence for the gradual adaptation of EFP in a marine environment and the potential sensitivity of YFP to genetic damage. Compared to the 34 cetacean genomes sourced from public databases, the 2 new assemblies demonstrate superior continuity with the longest contig N50 and scaffold N50 values, as well as the lowest number of contigs. The improvement of telomere-to-telomere gap-free reference genome resources supports conservation genetics and population management for finless porpoises.

## Introduction

**Figure 1:**
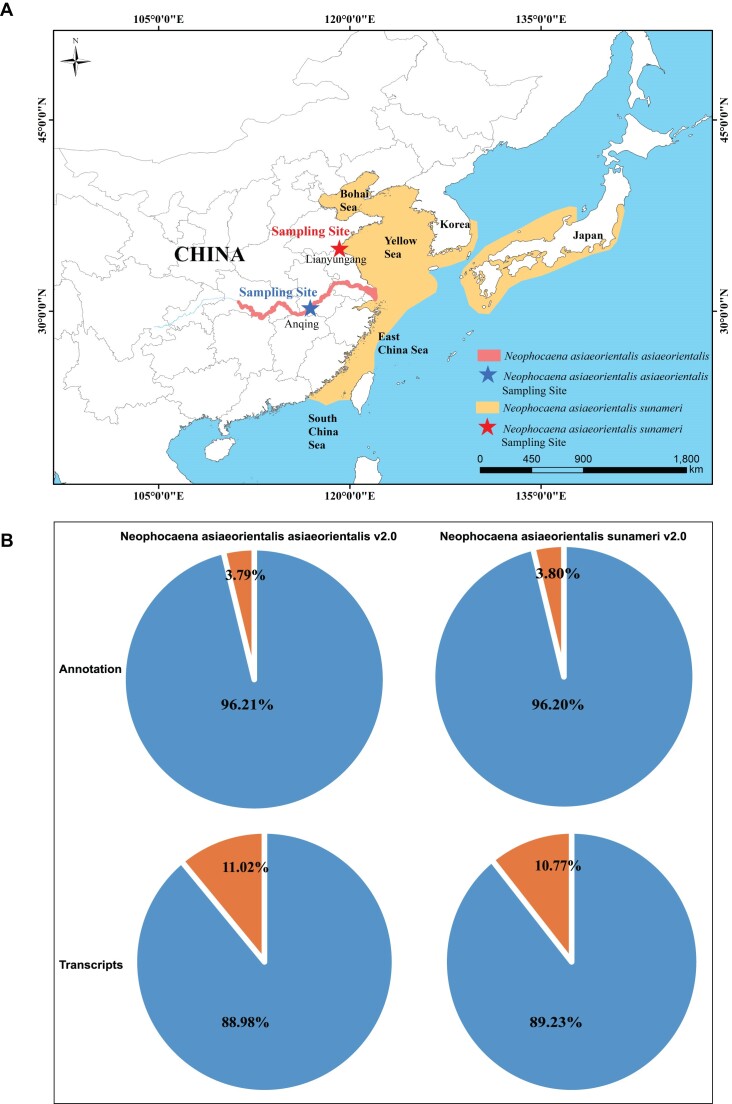
Sample site and genome assessment of YFP v2.0 and EFP v2.0. (A) Location distribution and sampling site of the YFP and EFP. (B) Proportions of genes that could be functionally annotated and transcriptionally detected in YFP v2.0 and EFP v2.0.

Finless porpoises (*Neophocaena* spp.) are small toothed whales capable of inhabiting freshwater (Yangtze River) and saltwater (coastal waters of southern and eastern Asia) environments [1, 2]. They are characterized by a blunt, rounded head; an equal-width upper and lower jaw; and lack of an obvious dorsal fin [3, 4]. Based on morphological characteristics, geographic distribution, and molecular genetic evidence, it is generally believed that the finless porpoise can be divided into 2 species, namely, the Indo-Pacific finless porpoise (*Neophocaena phocaenoides*) and the narrow-ridged finless porpoise (*Neophocaena asiaeorientalis*) [5, 6]. In China, there exist 2 subspecies of the narrow-ridged finless porpoise: one is the freshwater Yangtze finless porpoise (*N. a. asiaeorientalis*, YFP), which exclusively inhabits the middle and lower reaches of the Yangtze River and adjacent Dongting and Poyang lakes. The other subspecies is the marine East Asian finless porpoise (*N. a. sunameri*, EFP), which can be found in the coastal waters of the Yellow Sea and Bohai Sea, as well as in the northern waters of the East China Sea [5, 6] (Fig. 1A).

The investigation of the evolutionary origins and conservation genetics of finless porpoises is a pressing concern for scientists. Yang et al. [7] identified a significant genetic structure between either the Yangtze River population or the Yellow Sea population and the South China Sea population by analyzing the sequences of mitochondrial DNA (mtDNA) control region of finless porpoises in Chinese waters. This result was supported by subsequent mtDNA sequences, nuclear DNA microsatellites, single nucleotide polymorphisms (SNPs), and major histocompatibility complex (MHC) loci [8–12]. The genetic diversity of East Asian finless porpoises surpasses that of the other 2 populations, indicating it as the likely center of origin for this species [7]. Zheng et al. [13] analyzed the sequences of the mtDNA control region of 7 local populations of Yangtze finless porpoises in the middle and lower reaches of the Yangtze River and found that the overall level of genetic diversity was low. Notably, the downstream population showed richer genetic variation than the midstream population. Such a genetic pattern reflects, to some extent, the marine origin and evolutionary history of the Yangtze population. Based on genomic analysis of finless porpoise populations, a significant genetic structure was identified among the 3 populations, indicating local adaptive evolution and emphasizing the evolutionary distinctiveness and conservation significance of the Yangtze finless porpoise [14].

Availability of a high-quality genome assembly not only is critical for the genomic studies of finless porpoises but also would be a valuable resource for comparative genomics and evolutionary studies of cetaceans. The first draft of the YFP genome assembly (GCF_003031525.2) was published in 2018 with a size of 2.3 Gb generated by short-read sequencing on the Illumina HiSeq 2000 platform [14]. The assembly comprised 13,698 scaffolds, with a scaffold N50 of 6.3 Mb, excluding the minimum sequence length (100 bp) consideration. Although progress had been made in genome-wide studies of YFP through the availability of this draft, such as immune changes with age and gene expression profiles in different habitats [15, 16], the lack of chromosomal information limits the potential application of the data. Recent advances in ultra-long ONT and PacBio HiFi sequencing technologies, as well as assembly algorithms, have facilitated the development of telomere-to-telomere (T2T) genome assemblies. The completion of the T2T human genome sequence and the full Y chromosome sequence represents a significant milestone in the field of human genomics research [17, 18]. The T2T genome has emerged as a hotspot in genomic research, demonstrating extensive applications to other animal species like chicken and fish [19, 20]. T2T genome assemblies can serve as a benchmark with enhanced accuracy and comprehensive genomic references for future studies, facilitating the confident identification and annotation of genes, regulatory elements, and other functional components.

A high-quality genome can support finer genetic analyses, such as the length, number, and distribution of key indicators of inbreeding, such as runs of homozygosity and identical by descent [21, 22], and these analyses are more urgent than ever for the conservation of endangered species. In this study, we utilize PacBio HiFi, Nanopore, and Hi-C data to generate improved telomere-to-telomere gap-free genomes of Yangtze and East Asian finless porpoises. We compare the quality of the newly drafted assemblies with previously available versions and explore synteny blocks, structural similarities, phylogenetic relationships, gene family expansion, and inference of selection in relation to several other mammals. Finless porpoises serve as a representative example for comprehending speciation, evolution, and population genetics. The high-quality chromosomal-level references help elucidate ecological and aquatic adaptation mechanisms in cetaceans.

## Results

### Genome sequencing and gap-free assembly

We integrated PacBio HiFi long reads, ultra-long ONT reads, and Hi-C sequencing data to generate chromosome-level genome assemblies for YFP and EFP. Henceforth, we refer to this new T2T genome as 2.0 and the original genome as 1.0. We generated approximately 123 Gb (49×) PacBio HiFi reads, 279 Gb (111×) Hi-C reads, and 225 Gb (90×) ONT reads for YFP v2.0 (Supplementary Table S1). In this study, we supplemented the existing dataset comprising 62× PacBio HiFi and 85× Hi-C reads of the EFP [23] with an additional 215 Gb (86×) of ONT reads (Supplementary Table S1). The YFP v2.0 genome assembly comprised 23 scaffolds, with both contig N50 and scaffold N50 measuring 125.12 Mb (Table 1). These scaffolds were assembled into 21 autosomal chromosomes, 1 X chromosome, and 1 mitochondrial chromosome, resulting in a final assembly size of 2.48 Gb (Supplementary Table S2). Similarly, the EFP v2.0 genome assembly consisted of 24 scaffolds, with both contig N50 and scaffold N50 measuring 128.00 Mb (Table 1). These scaffolds were also assembled into 21 autosomal chromosomes, 1 X + Y chromosome, and 1 mitochondrial chromosome, with a final assembly size of 2.50 Gb (Supplementary Table S2).

**Table 1:** Genome assembly statistics of YFP and EFP

	YFP v2.0	YFP v1.0	EFP v2.0	EFP v1.0
Total size of assembled genome (Gb)	2.48	2.27	2.50	2.50
Contig N50 (Mb)	125.12	0.09	128.00	84.69
Contig N90 (Mb)	83.62	0.02	80.21	29.54
Number of contigs	22	66,345	23	51
Scaffold N50 (Mb)	125.12	6.34	128.00	122.40
Scaffold N90 (Mb)	83.62	1.13	80.21	80.21
Scaffolds number	22	13,698	23	23
Number of base chromosomes	22	22	23	23
Number of gap-free chromosomes	22	0	23	7
Number of gaps	0	52,647	0	28
Number of telomeres (pairs/single)	20/2	0/0	21/2	21/2
Number of estimated centromeres	22	0	23	23
TE size	42.54%	NA	42.80%	42.23%
GC content	41.70%	41.00%	41.70%	41.70%
BUSCO* (genome)	95.2%	94.0%	95.3%	95.4%
Gene number	23,139	18,479	23,101	22,814
Newfound gene number	5,480	NA	1,453	NA
Functional proteins	96.21%	NA	96.20%	97.31%
BUSCO* (protein)	97.5%	94.6%	97.6%	97.9%
Data source	This study (PRJNA915046)	GCF_003031525.2	This study (PRJNA859258)	GCA_026225855.1

Note: The term “v2.0” denotes the updated genome assembly and annotation produced in the present study, while “v1.0” refers to the initial draft of the genome assembly and annotation that was previously published. “BUSCO*” indicates the percentage of complete BUSCO evaluation. “pairs/single”: “pairs” indicate that the telomeres were found at both ends of the chromosomes; “single” indicates that the telomeres were found only at the one end of chromosomes. “Newfound genes” indicate that the genes were predicted in the extra sequence segments from the current assembly and were annotated in the current assembly.

**Figure 2:**
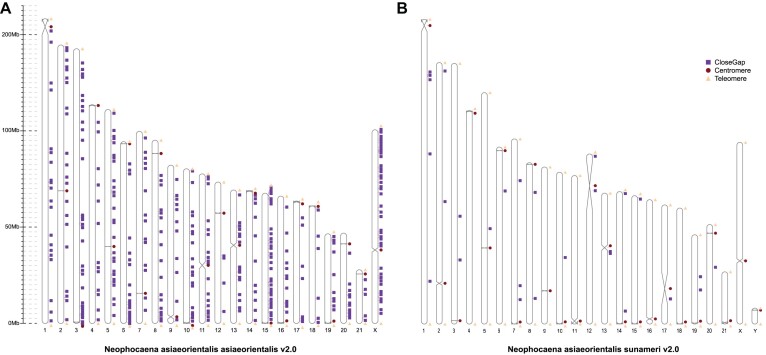
T2T-resolved assembly of YPF v2.0 and EFP v2.0 and functional enrichment of genes in the centromere region. Structure of T2T and gap-free chromosomes in (A) YFP v2.0 and (B) EFP v2.0. All 21+X/Y chromosomes are drawn to scale and the ruler indicates chromosome length. Triangles indicate the presence of telomere sequence repeats. Circles represent the locations of centromeric regions. The gap positions in the v1.0 genome assemblies are marked with squares to the right of the chromosome in the v2.0 genome assemblies.

We have significantly enhanced the contiguity, accuracy, and completeness of the YFP v1.0 (GCA_003031525.2) and EFP v1.0 (GCA_026225855.1) assemblies. The contig N50 values of the 2 new genomes were consistent with their respective chromosome lengths, and a single contig represented a complete chromosome, which is notably superior to the recently published finless porpoise genomes (e.g., 125.12 Mb vs. 0.09 Mb for YFP and 128.00 Mb vs 84.69 Mb for EFP) (Table 1). The YFP v1.0 and EFP v1.0 genome assemblies had 52,647 and 28 gaps, respectively, whereas the new assembly process produced gap-free genomes, obviously improving contiguity (Fig. 2A, B). As of May 2024, the killer whale genome assembly (GCF_937001465.1) stands out among the 34 publicly available cetacean genomes sourced from the NCBI and CNGBdb (China National GeneBank DataBase) databases. It features an N50 length of 45.6 Mb and is composed of 570 contigs. In our study, the 2 newly assembled genomes exhibit a marked improvement in contiguity. The contig N50 length achieved in our assemblies is 3-fold that of the killer whale genome, with the number of contigs comprising only 4.03% of the latter. Moreover, the analysis of the blue whale genome (GCF_009873245.2) shows a contig N50 value that is only 4.0% of our assemblies, while its contig count is 42 times higher [24] (Supplementary Table S3). The comparative metrics highlight the superior continuity of the YFP and EFP genome assemblies in this study.

The quality values obtained from Merqury’s *k*-mer analysis [25] for YFP v2.0 and EFP v2.0 were calculated as 60.18 and 64.38, respectively (Supplementary Table S4). These values indicated the superior quality of our assemblies. It is essential to emphasize that Merqury quality values span from 0 to 255, with higher values denoting superior quality. These values indicate a foundational accuracy level of 99.999%, confirming the high quality of our assembly for each component. Moreover, the mapping rates of RNA reads to the 2 genome assemblies were 95.47% and 95.42%, respectively, whereas they were 70.01% and 92.34% for previously published assemblies (Supplementary Table S5). Moreover, the mapping results of ONT long reads with a mapping quality >20 indicate a read coverage of 99.99% for YFP v2.0 and EFP v2.0 whole genomes (Supplementary Fig. S1). Using the BUSCO evaluation, our results represent a completeness of 95.20% for YFP and 95.30% for EFP (Table 1 and Supplementary Fig. S2). Additionally, we have identified/predicted both the telomeric repeat and centromere candidate region in YFP v2.0 and EFP v2.0 (Fig. 2A, B and Supplementary Tables S6–S7). Telomeric repeat units of the YFP v2.0 genome were detected at both ends of 18 chromosomes and at 1 end of 3 chromosomes. Similarly, telomeric repeat units of the EFP v2.0 genome were detected at both ends of 20 chromosomes and at 1 end of 2 chromosomes. Telomeric repeat units were detected at 85% and 90% of the chromosome at both ends in YFP v2.0 and EFP v2.0 genomes, respectively. Our estimations of the centromere region are novel and were not present in YFP v1.0. Finally, we also utilized Hi-C data for chromosome sequencing and orientation, resulting in a comprehensive Hi-C matrix that effectively illustrates genome-wide interactions (Supplementary Fig. S3). These findings suggest that the new genome assemblies of YFP and EFP represent a significant improvement over the previously published genome assembly, achieving near T2T completeness.

### Gene prediction and annotation

Two strategies, including *de novo* and homolog-based methods, were applied to annotate repeat elements. The genomes of YFP v2.0 and EFP v2.0 contained 1,058.09 Mb (42.54%) and 1,069.10 Mb (42.80%) of repetitive sequences, respectively (Supplementary Fig. S4 and Table S8). Long interspersed nuclear elements (LINEs) were the most abundant type of annotated transposable elements, accounting for 38.88% and 39.10% of the genomes of YFP v2.0 and EFP v2.0, respectively (Supplementary Table S9). In total, the number of predicted protein-coding genes was 23,139 in the YFP v2.0 genome and 23,101 in the EFP v2.0 genome (Table 1). The roughly comparable number of predicted protein-coding genes for both T2T genomes is further evidence supporting the gene models (Supplementary Tables S10–S13). It is worth noting that the length distribution of gene models at the levels of genes, coding sequence, exons, and introns showed a similar trend when compared to those of YFP v1.0 (GCA_003031525.2), EFP v1.0 (GCA_026225855.1), and Bottlenose Dolphin (GCF_011762595) (Supplementary Fig. S5). In the predicted gene models of YFP v2.0 and EFP v2.0, the BUSCO analysis identified 97.5% and 97.6% complete conserved single-copy mammalian genes (odb10), respectively (Table 1 and Supplementary Fig. S2). A total of 22,263 (96.21%) gene models in the YFP v2.0 genome and 22,224 (96.20%) gene models in the EFP v2.0 genome were annotated in at least 1 database, including NR, SwissProt, KEGG, KOG, TrEMBL, Gene Ontology, and InterPro (Fig. 1B and Table 1). Furthermore, 71.22% (16,480) of YFP v2.0 genes and 71.24% (16,457) of EFP v2.0 genes were annotated in 5 functional databases, namely NR, SwissProt, KEGG, KOG, and InterPro (Supplementary Fig. S6 and Table S14). Finally, 20,589 (88.98%) and 20,613 (89.23%) genes from the YFP v2.0 and EFP v2.0 genomes, respectively, were determined to be transcriptionally active based on the analysis of 24 RNA sequencing (RNA-seq) datasets (Fig. 1B). In conclusion, our annotation of 2 gene sets of reliably high quality has established a robust foundation for further research.

### Analysis of centromere-related genes

The centromere plays a necessary role in cell division for eukaryotes. The intrinsic mechanisms underlying the evolution of centromere structure among different species have not been fully revealed due to the challenge of assembling highly repetitive sequences [26]. In this study, we conducted predictions on repeat monomers within the YFP v2.0 and EFP v2.0 genomes, potentially constituting the centromeres (Fig. 2A, B and Supplementary Tables S15–S16). The monomeric sequences vary in length from 99 to 201 bp, with the 144-bp, 150-bp, and 138-bp monomers being the most abundant. Centromeres are composed of more than 1 repeat monomer and are located within transposable element (TE)– and tandem repeat-enriched regions, which are areas with relatively lower gene density (Supplementary Fig. S7). A total of 235 and 237 genes were identified in the candidate centromere regions for YFP v2.0 and EFP v2.0, respectively, through predictions generated by centromere-finding software. Moreover, the newly discovered centromere regions for YFP v2.0 hosted approximately 56 genes, while only 20 genes were found in analogous regions for EFP v2.0. The “newly identified centromere regions” refer to specific areas identified in the genome that have been recently assembled but were not included in the previously published version. This discovery may suggest the presence of novel, previously uncharacterized centromere sites. We further compared the genes located in the centromere and noncentromere regions in the genomes of YFP v2.0 and EFP v2.0, respectively, and found that there were no significant differences in the expression patterns of these genes in different transcripts (Supplementary Fig. S8 and Supplementary Tables S17–S18). Additionally, we analyzed the functional enrichment of genes located in the centromere regions of the 2 genomes. These genes were mainly associated with keratin filament (GO:0045095), intermediate filament (GO:0005882), gamete generation (GO:0007276), and channel activity (GO:0015267), as indicated by Gene Ontology (GO) enrichment (Supplementary Table S19). These findings are consistent with results obtained through analysis using the KEGG database, highlighting the essential role of centromeres in the segregation and positioning of chromosomes (Supplementary Fig. S9 and Tables S20–S21). Centromeres play a vital role in providing structural support to the nucleus, thereby ensuring the integrity of chromosomes, and are essential for the processes of mitosis and meiosis. Moreover, centromeric regions have the potential to engage with mechanisms related to cell cycle regulation, cell division signaling pathways, and channel activity, thereby impacting the progression of cell division [27].

### Variations between YFP and EFP genomes

**Figure 3:**
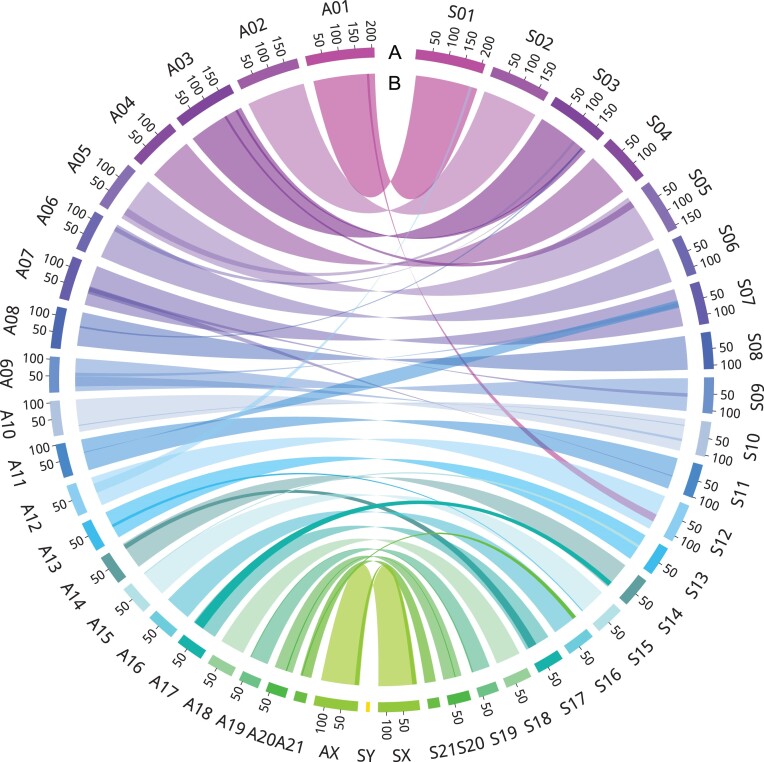
Synteny analysis of YFP v2.0 and EFP v2.0 genomes. (A) chromosomes scale, unit length is Mb. (B) Syntenic blocks between YFP v2.0 and EFP v2.0.

**Figure 4:**
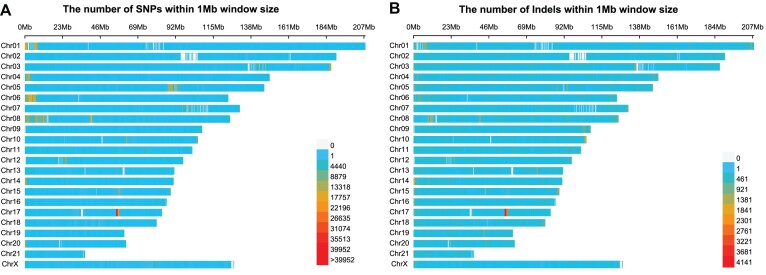
Structure variant between YFP v2.0 and EFP v2.0 genome assembly with YFP v2.0 genome assembly for reference. The density plot of (A) SNPs and (B) indels between YFP v2.0 and EFP v2.0 genome assembly. The numerical values adjacent to the color legend indicate the count of SNPs/indels per megabase (MB) window, where the gray legend represents zero. Each color corresponds to a specific range of values. For instance, in panel A, the initial blue legend represents the range from 1 to 4,440.

Synteny analysis comparing the gene order between YFP v2.0 and EFP v2.0 identified 907 significant shared syntenic blocks, covering 89.59% (41,428) of the genes, and revealed 17 chromosomal rearrangements (Fig. 3). Further comparison of the genomic sequences of the YFP v2.0 and EFP v2.0 genomes, however, yielded numerous variations between the two, including 3,887,060 SNPs (Fig. 4A) and 704,944 short insertions/deletions (indels) (Fig. 4B). The SNPs exhibited predominant occurrences in intergenic regions (65.28%) and intronic regions (32.66%), with minimal presence in exon regions (0.64%). Similarly, the distribution of variations in indels was less concentrated in exon regions, accounting for only 0.23% (Supplementary Table S22). Of the exon region variations, 12,203 SNPs (11,987 nonsynonymous SNPs, 189 stop-gain SNPs, and 27 stop-loss SNPs) and 953 indels (464 frameshift deletion, 467 frameshift insertion, 20 stop-gain indels, and 2 stop-loss indels) were identified and functionally associated with 6,158 and 582 genes, respectively. KEGG enrichment analysis revealed that these genes were significantly (*P* < 0.05) enriched in “NF-kappa B signaling pathway,” “complement and coagulation cascades,” “antigen processing and presentation,” and “intestinal immune network for IgA production” (Supplementary Fig. S10 and Table S23).

The genes coding for the mutated regions of the YFP and EFP are widely enriched in immune-related pathways. This association may be intricately linked to the distinct pathogenic microorganisms unique to freshwater and seawater environments. Marine mammals exhibit a diminished MHC diversity attributed to decreased encounters with microparasitic diversity in their marine habitat relative to their terrestrial origin. This phenomenon implies that mammals encounter distinct pathogenic pressures in varied ecological settings, potentially influencing the evolution of immune-related genes [28]. Evolutionary analyses of the innate immune pattern recognition receptor (toll-like receptors) in the YFP and the marine finless porpoise indicate that the YFP has undergone specific adaptive changes [29]. The microbial diversity and pathogenicity of freshwater and seawater environments vary, leading to distinct effects of pathogenic microorganisms on the organisms in these 2 types of environments [30]. Therefore, the YFP and EFP would be expected to undergo adaptive evolution to adapt to the pathogen stresses specific to their respective ecological environments, freshwater and seawater.

By comparing the assembly results of the YFP and EFP versions, it was found that the YFP v2.0 assembly included 5,480 new genes, while the EFP v2.0 assembly included 1,453 new genes compared to the previous version of the assembly (Table 1 and Supplementary Table S24). These genes were expressed in all 24 samples (Supplementary Fig. S11 and Tables S25–S26). GO and KEGG functional enrichment analysis indicated that these genes are primarily enriched in immunity and iron binding, among other functions. These biological processes encompass a wide range of aspects, including cellular structure and function, protein synthesis, signal transduction, immune response, and energy metabolism (Supplementary Tables S27–S30).

### Phylogeny analysis

**Figure 5:**
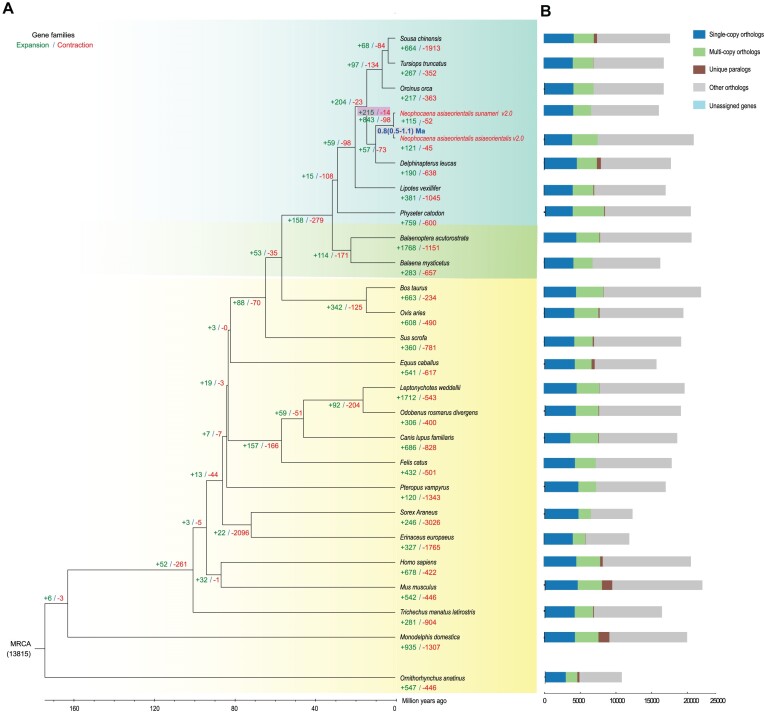
Genome evolution of YFP v2.0 and EFP v2.0. (A) Divergence time between YFP v2.0 and EFP v2.0 and number of expanded and contracted gene families. Green and red numbers indicate gene family expansions and contractions, respectively. Ma: million years ago; MRCA: most recent common ancestor. (B) A comparison of gene families associated with orthologs and paralogs in YFP v2.0 and EFP v2.0 and 24 other mammal species.

Gene family analysis was performed on 506,098 protein-coding sequences derived from 10 cetaceans and 16 terrestrial mammalian species. The sequences were clustered into 22,196 gene families, which include 594 species-specific genes identified in YFP v2.0 and EFP v2.0 (Supplementary Table S31). Among the dataset, a total of 2,161 single-copy gene families were identified. Subsequently, multiple sequence alignments were conducted, which were then followed by the reconstruction of evolutionary trees. The resulting phylogenetic tree produced a topology consistent with previous studies [31], highlighting its reliability and alignment with established scientific knowledge. This analysis notably revealed the topological arrangement within branches of mammals. The emergence of the EFP branch alongside its YFP counterpart was particularly noteworthy (Supplementary Fig. S12). Our analysis had revealed that the divergence time between the YFP and EFP ranges from 0.5 to 1.1 million years ago (Fig. 5A, B and Supplementary Fig. S13), which is the first estimate at the molecular level since their classification as 2 distinct subspecies [14].

### Gene family and positive selection analysis

**Figure 6:**
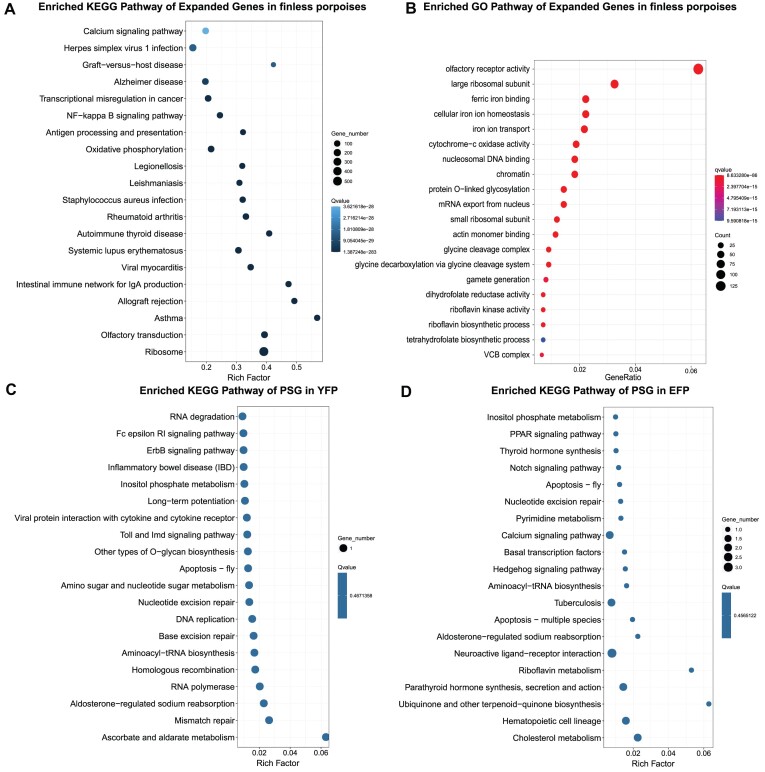
Functional enrichment of genes. Significant (A) KEGG and (B) GO enrichment of expanded gene families in YFP and EFP lineage. KEGG enrichment analysis of positively selected genes in (C) YFP and (D) EFP, respectively.

We used CAFÉ v4.0 to analyze the evolution of gene families based on orthologous clusters of protein-coding sequences from 26 mammals. Upon comparing the genomes of YFP v2.0 and EFP v2.0 with their most recent common ancestor, it was observed that 843 gene families underwent expansion while 98 gene families experienced contraction (Fig. 5A, B). Among the 215 expanded gene families identified in the YFP v2.0 and EFP v2.0 lineage, a total of 2,674 genes were determined to be significantly associated (*P* < 0.05) (Supplementary Table S32). We observed an expansion for genes significantly enriched in several KEGG pathways, including “antigen processing and presentation,” “intestinal immune network for IgA production,” “oxidative phosphorylation,” and the “calcium signaling pathway” (Fig. 6A). The significantly enriched GO terms, including “ferric iron binding,” “iron ion transport,” “riboflavin biosynthetic process,” “tetrahydrofolate biosynthetic process,” and “cytochrome-c oxidase activity,” also expanded (Fig. 6B).

The Codeml program in PAML with a branch-site model was employed to test for selective pressure based on orthologous clusters of 10 cetaceans, including *N. a. asiaeorientalis* (Yangtze finless porpoise; NCBI taxon ID:1706337), *N. a. sunameri* (East Asian finless porpoise; NCBI taxon ID:861190), *Tursiops truncatus* (bottlenose dolphin; NCBI taxon ID:9739), *Orcinus orca* (killer whale; NCBI taxon ID:9733), *L. vexillifer* (Yangtze River dolphin; NCBI taxon ID:118797), *Physeter catodon* (sperm whale; NCBI taxon ID:9755), *Balaenoptera acutorostrata* (Minke whale; NCBI taxon ID:9767), *Balaena mysticetus* (bowhead whale; NCBI taxon ID:27602), *Delphinapterus leucas* (beluga whale; NCBI taxon ID:9749), and *Sousa chinensis* (Indo-Pacific humpback dolphin; NCBI taxon ID:103600). We identified 41 positively selected genes (PSGs) in the YFP lineage, which were functionally enriched in “RNA degradation,” “nucleotide excision repair,” “DNA replication,” “mismatch repair,” and “homologous recombination” pathways (*P* < 0.05) (Fig. 6C and Supplementary Table S33). Additionally, a total of 44 PSGs within the EFP lineage were involved in “sodium-dependent phosphate transport,” “sodium symporter activity,” “aldosterone-regulated sodium reabsorption,” and “calcium signaling pathway” (Fig. 6D and Supplementary Table S34).

## Discussion

The availability of reliable chromosome-level genome assemblies provides a remarkable improvement in identifying genes, characterizing genomic regions, and performing comparative genomic analyses. In the present study, we assembled telomere-to-telomere and gap-free Yangtze finless porpoise and the East Asian finless porpoise genomes by combining PacBio long reads, Hi-C, and short-read sequencing technologies. The new assemblies have higher contiguity and completeness, as well as more complete single-copy BUSCO genes with fewer fragmented or missing genes than the first drafts. Compared to the 34 cetacean genomes sourced from public databases, the 2 genomes newly assembled in our study exhibit the longest contig N50 and scaffold N50, along with the lowest number of contigs, thus achieving superior continuity.

Gene family expansion analysis revealed significantly enriched pathways and GO terms associated with the regulation of immune resistance and hypoxic tolerance. Gene families associated with “oxidative phosphorylation,” “cytochrome-c oxidase activity,” “riboflavin biosynthetic process,” “ferric iron binding,” and “iron ion transport” exhibited expansion in finless porpoises. Iron ions play a crucial role in numerous essential physiological functions within living organisms, such as oxygen transportation, electron transport chains, and various metabolic pathways. Several sets of redox reactions are necessary to maintain effective gas exchange in water for cetaceans, and iron is often used in these reactions as an electron acceptor [32]. The expansion of redox reaction and iron ion gene families in cetaceans potentially improved the efficacy of oxygen utilization and facilitated adaptation to hypoxic conditions in aquatic habitats. An increase in the number of oxidation reduction and iron-binding gene families was also observed in *Lipotes vexillifer*, a species that encounters hypoxic conditions during dives [33]. The changes in pathogenic microorganisms that occur during the reintroduction of cetaceans from land to sea present a significant challenge to their survival and may have influenced the evolution and adaptation of immune genes [34]. The expansion of immune-related gene families in finless porpoises in this study enhanced the process of antigen presentation and conferred resistance against various pathogenic microorganisms amid changes in their habitat. Genomic studies of the *S. chinensis* have revealed that cetaceans have developed various species-specific gene families related to immunity and DNA repair, which are linked to potential adaptive mechanisms [35]. We suggest these genes play a crucial role in facilitating hypoxic tolerance and enhancing immune resistance in finless porpoises, thereby reflecting potential mechanisms of adaptation to the aquatic environment. Further investigations are required to elucidate the specific functions of these gene families and their potential significance in the biology of finless porpoises.

Selection pressure analysis identified genes associated with DNA damage repair in the YFP. The selective pressure to evolve DNA damage repair pathways implied that the Yangtze finless porpoise might be experiencing increased threats to genome stability. The mechanism of DNA damage repair plays a crucial role in preserving genome integrity by enabling cells to identify and repair DNA damage, thereby averting the accumulation of harmful mutations [36]. In a comparative genomic analysis between the South China tiger and the Amur tiger, it was noted that genes related to DNA repair underwent positive selection in the South China tiger [21]. The observed phenomenon could be explained by the higher probability of genome instability in the temperate and subtropical habitats of the South China tiger. This may be linked to metabolites generated by intestinal microflora, which possess the ability to trigger DNA damage [37]. The stability of the genome or gene expression system in the Yangtze finless porpoise across different organs and life stages remains uncertain. However, one potential interpretation of these data is the suggestion that the Yangtze finless porpoise could be vulnerable to genomic instability triggers in the Yangtze River, such as water pollutants, which may increase the likelihood of DNA damage [38]. Pollutants found in the Yangtze River possess the capacity to accumulate within the food chain, leading to cellular DNA damage and impacting the genome stability of the Yangtze finless porpoise [39, 40]. This makes a compelling case for the improved conservation of the species and the development of more rigorous water pollution mitigation practices.

Genes associated with high salt tolerance in the EFP were also identified through selection pressure analysis. These metabolic pathways play an important role in regulating sodium levels in the body [41]. Among these PSGs, 6 were potentially associated with the adaptation of EFP to a high-osmolarity environment, including the Na(+)/H(+) exchange regulatory cofactor *NHE-RF2* and sodium-dependent phosphate cotransporter *SLC34*. These genes identified are likely to have significant implications in the control of urine formation and the preservation of water–salt metabolic balance [41], suggesting that the East Asian finless porpoise may have a different urine formation process. Previous genome-selective sweep analyses in the finless porpoises revealed that the *SLC14A* in the East Asian finless porpoise underwent positive selection [14]. Comparative analyses conducted at the transcriptome level between the Yangtze finless porpoise and the East Asian finless porpoise revealed a notable upregulation of the *NHE3* in the kidneys of the East Asian finless porpoise. These results imply some adaptive enhanced osmoregulatory capability in the East Asian finless porpoise [42]. The aforementioned studies suggest that the East Asian finless porpoise has evolved a complex and efficient osmoregulatory mechanism as it acclimatized to the hypertonic marine environment, demonstrating adaptations at both molecular and transcriptional levels.

Identification of the centromere, telomere, and associated genes can serve as valuable resources for a comprehensive understanding of chromosome stability, recombination, repair mechanisms, and evolutionary processes. Overall, these are the most continuous cetacean genome assemblies to date, with chromosome-scale contigs and no gaps. This study will lay a foundation for population genomics studies at the whole genome level and deepen the scientific understanding of issues related to population conservation and adaptation mechanisms.

## Materials and Methods

### Sample collection, DNA extraction, and sequencing

We collected an adult dead female *N. a. asiaeorientalis* (NCBI taxon ID:1,706,337) sample from Lianzhou Lake, Anqing City, Anhui Province, China (N30°15ʹ32ʺ, E116°54ʹ38ʺ) in 2021 and a dead juvenile male *N. a. sunameri* (NCBI taxon ID:861,190) sample from the Yellow Sea near Lianyungang City, Jiangsu Province, China (N34°55ʹ27ʺ, E119°11ʹ37ʺ) in 2019 for sequencing (Fig. 1A). The Office of Fishery Supervision and Management in the Yangtze River Basin, Ministry of Agriculture and Rural Affairs of the People’s Republic of China has designated our research institution to perform postmortem analysis and genetic preservation on deceased porpoises. No ethical considerations were taken into account in this study. DNA were extracted from muscle tissues following the phenol/chloroform DNA extraction method. DNA extracted from the YFP were utilized to construct PacBio HiFi, Hi-C, and Oxford Nanopore Technologies (ONT) libraries. DNA extracted from an EFP were utilized to construct an ONT library. The PacBio HiFi library was constructed using SMRTbell Prep Kit 3.0 (Pacific Biosciences) and subsequently sequenced on the PacBio Sequel II system (RRID:SCR_017990) in circular consensus sequence (CCS) mode. To collect data for the Hi-C library, the muscle tissues were first fixed in 1% formaldehyde (Sigma) for cross-linking and resuspended in lysis buffer. Then, MboI (NEB) restriction endonucleases were used to fragment the chromatin in the muscle to fragment DNA. The DNA fragments were captured by utilizing streptavidin-coated magnetic beads (Thermo Fisher Scientific) following biotin labeling and crosslinking using T4 DNA Ligase (ENZYMATICS). The Hi-C library was finally sequenced on a BGI MGISEQ platform. To generate and sequence ONT libraries, we isolated genomic DNA using the CTAB [43] method, selected fragments exceeding 5 kb in size with the SageHLS HMW library system (Sage Science), processed the DNA with the Ligation sequencing 1D kit (SQK-LSK109; Oxford Nanopore Technologies), and subsequently sequenced the ONT libraries on a PromethION platform (Oxford Nanopore Technologies) at the BGI.

### Gap-free genome assembly and quality assessment

We utilized SMRTLink v11.0.0 [44] to filter PacBio HiFi reads, applying the following criteria: a minimum requirement of 3 full-length subreads for generating CCS, a draft length threshold of 500 bp before polishing, and aiming for a predicted accuracy of 0.99. To filter Hi-C reads, index reads were removed, and reads were filtered using the following SOAPNUKE v2.0 (RRID:SCR_015025) [45] parameters: N rate ≥0.01, low quality ≤20, and low quality rate ≥0.1. Subsequently, ONT reads were filtered based on a length <5 kb and a quality value <7. The Necat pipeline v 20,200,119 (RRID:SCR_025350) [46] was utilized for the enhancement of ONT reads. This was achieved through the application of error correction algorithms to evaluate quality scores, *k*-mer frequencies, and alignment methods. This process enhanced the accuracy of reading and generated refined results suitable for further analyses. To achieve gap-free chromosome-level assemblies, we employed both the Hifiasm v0.15.1 (RRID:SCR_021069) [47] and Necat pipeline v20200119 (RRID:SCR_025350) [46] to separately assemble PacBio HiFi reads and ONT-corrected reads into the initial contigs. The Purge_haplotigs program (RRID:SCR_017616) [48] was utilized to eliminate redundant contigs that exhibited similar sequences but distinct haplotypes, specifically targeting those with aligned coverage below 30%. This strategic approach significantly optimizes the assembly process by removing redundant information, thus improving the accuracy of genome assembly. We utilized Hi-C data to cluster, order, and orient the contigs into pseudo-chromosomes through the implementation of the Juicer v1.5 (RRID:SCR_017226) [49] and 3D-DNA v180922 (RRID:SCR_017227) [50] pipelines. Ultra-long ONT reads and contigs were used to generate gapless scaffolds through the LR Gapcloser v1.0 (RRID:SCR_016194) [51] and TGSgapcloser (v 1.0.1) [52] pipelines.

Various metrics were used to evaluate the quality of the gap-free genome assemblies, including contiguity, accuracy, and completeness. First, we calculated the length metrics of genomic sequences to evaluate contiguity and subsequently used Merqury v1.3 (RRID:SCR_022964) [25] with *k*-mer set to 21 to assess the accuracy. Second, BUSCO (RRID:SCR_015008) [53] evaluation was conducted to assess the completeness. Third, we also mapped PacBio HiFi, ONT, and RNA-seq data into the genome assemblies using Minimap2 (RRID:SCR_018550) [54] and Hisat2 v2.1.0 (RRID:SCR_015530) [55] to assess the completeness. In addition, we utilized the quarTeT pipeline (RRID:SCR_025258) [56] to search for telomere repeat sequences and centromere regions in YFP and EFP.

### Genome annotation

Repetitive sequence annotation was identified using both *de novo* and homology-based approaches. For the *de novo* strategy, RepeatModeler v1.0.4 (RRID:SCR_015027) [57] was employed to identify repetitive elements, whereas LTR_Finder v1.0.7 (RRID:SCR_015247) [58] was used for the specific annotation of long terminal repeats. For homolog-based prediction, RepeatMasker v4.0.7 (RRID:SCR_012954) [59] was utilized to detect DNA TEs, while RepeatProteinMasker (v4.0.7) was employed to identify protein-based TEs, both based on the Repbase database v21.12 (RRID:SCR_021169). Additionally, Tandem Repeat Finder v4.10.0 (RRID:SCR_022193) [60] was used to identify tandem repeats. The utilization of these tools facilitated a comprehensive annotation of repetitive sequences, leading to a substantial improvement in the accuracy and detail of the analysis.

A combination of RNA-seq, homology-based, and *de novo* prediction strategies was utilized to identify protein-coding genes in the genomes of YFP and EFP. RNA-seq data [15, 16] were mapped to genome assemblies with Hisat2 v2.1.0 (RRID:SCR_015530) [61] with the following parameters: –sensitive –no-discordant –no-mixed -I 1 -X 1000 –max-intronlen 1,000,000. The produced BAM alignments were further assembled into gene models with StringTie v1.3.5 (RRID:SCR_016323) [62] with the following parameters: -f 0.3 -j 3 -c 5 -g 100 -s 10,000 and validated using PASA v2.5.2 (RRID:SCR_014656) [63]. The coding sequences were identified by TransDecoder v5.5.0 (RRID:SCR_017647) [64] with default parameters. Utilizing 149,956 genes from 8 closely related cetacean species and transcriptomic sequencing data from 24 YFPs as input files, the GeMoMa v1.9 (RRID:SCR_017646) [65] software was employed to conduct homology-based prediction analysis (Supplementary Tables S8, S35–S36). A set of 1,000 high-quality genes, which were predicted by the GeMoMa software v1.9 (RRID:SCR_017646) and validated by OrthoDB (RRID:SCR_011980) for mammals, were randomly selected for training the predictors in Augustus v3.2.1 (RRID:SCR_008417) [66]. The Augustus (v3.2.1; RRID:SCR_008417) program was used to perform *de novo* prediction. We used GeMoMa software (v1.9; RRID:SCR_017646) to integrate all predicted protein-coding genes and annotated them with NR, Swissprot [67], KEGG (RRID:SCR_012773) [68], KOG, TrEMBL, InterPro (RRID:SCR_006695) [69], and GO (RRID:SCR_002811) [70] databases.

### Genome comparison and identification of newly assembled genes

SNPs and indels were identified using methods following those previously described [71]. Genome alignment was conducted utilizing the NUCmer program integrated within MUMmer v4.0.0 (RRID:SCR_018171) [72] to compare the v2.0 assembly with the v1.0 assembly, as well as the YFP assembly with the EFP assembly. Utilizing the maximum unique matches (MUM) mode involved setting parameters such as a minimum MUM length of 1,000 bp, a minimum similarity threshold of 90%, and the exclusion of matches below 40 bp. Alignment blocks were identified using the delta-filter program, while SNPs and indels were detected using the show-snps program and Syri v1.6.3 (RRID:SCR_023008) [73], respectively. Functional annotation of SNPs and indels was conducted using ANNOVAR (RRID:SCR_012821) [74] to assess their impacts on gene structure and function. Additionally, we utilized the CMplot R package (RRID:SCR_024514) [75] to visually depict the density distribution of these genetic variations. The objective of these analyses was to reveal structural variations between the v1.0 and v2.0 genome assemblies, as well as between the YFP and EFP genome assemblies. Variants were meticulously annotated utilizing the ANNOVAR package (RRID:SCR_012821) [74].

Genes were categorized as newly assembled when the gene region in the initial draft genome assembly showed a deletion of at least 50 bp and had a minimum overlap of 30% within that specific region.

### Gene family and phylogenomic analysis

Gene families of 26 species (Supplementary Table S31) were identified and clustered by OrthoFinder v2.3.11 (RRID:SCR_017118) [76]. We utilized MAFFT v7.310 (RRID:SCR_011811) and PhyML v3.3 (RRID:SCR_014629) software tools to align single-copy orthologous genes (1:1:1) and construct a maximum likelihood phylogenetic tree, respectively [77, 78]. The HKY85 model, known for its capacity to accommodate nucleotide substitution patterns, was applied in the tree construction procedure. To evaluate the reliability of the generated tree, 1,000 bootstrap replications were performed, providing statistical evidence for the branching structures. Species divergence time was calculated using MCMCTREE in PAML v4.9 (RRID:SCR_014932) [79]. Four divergence time points from TimeTree (RRID:SCR_021162) [80] were used to calibrate the divergence times: (a) *Ornithorhynchus anatinus* (Platypus; NCBI taxon ID:9258) and *Monodelphis domestica* (Opossum; NCBI taxon ID:13616) (163.7–185.9 million years ago [Mya]), (b) *Homo sapiens* (Human; NCBI taxon ID:9606) and *Mus musculus* (Mouse; NCBI taxon ID:10090) (81.3–91.0 Mya), (c) *B. mysticetus* and *B. acutorostrata* (21.3–28.8 Mya), and (d) *S. chinensis* and *T. truncatus* (2.0–3.8 Mya). The core-orthologous gene sets were identified by BLAST v2.0.14 (RRID:SCR_004870) [81] with an E-value threshold of 1 × E^−10^ (at least 10 syntenic genes allowed), and syntenic blocks were defined using MCscanX v1.5.2 (RRID:SCR_022067) [82]. Circos (RRID:SCR_011798) was used to plot the synteny results.

### Gene family expansion and contraction analysis

Protein sequences of YFP, EFP, and 24 published mammals were used to search homologs. Based on the gene families clustered by OrthoFinder v2.3.11 (RRID:SCR_017118), the CAFÉ v4.0 (RRID:SCR_005983) [83] software was used to perform expansion and contraction analyses in the clade of finless porpoises. Random birth and death models were employed to study gains and losses of gene families in a user-specified phylogeny. The global parameter λ, which describes both the gene birth (λ) and death (μ = −λ) rate for gene families in all branches of the tree, was estimated using maximum likelihood. Then the *P* value was calculated for each gene family, and *P* ≤ 0.01 was defined as a “significantly expanded or contracted gene family.” KEGG and GO enrichment analyses were conducted among these significantly expanded and contracted gene families.

### Gene positive selection analysis

Protein sequences of 2 finless porpoise and another 8 cetaceans were used to identify single-copy orthologs with OrthoFinder v2.3.11 (RRID:SCR_017118). Then Ka/Ks ratios for these single-copy orthologs were calculated by following steps. Initially, the single-copy orthologs underwent global alignment using PRANK (RRID:SCR_017228). Subsequently, alignment refinement via Gblocks (RRID:SCR_015945) was utilized to remove inadequately aligned positions and divergent regions, thereby isolating conserved blocks from the multiple alignment. Codeml from the PAML package [79] was ultimately employed to compute Ka/Ks ratios across various branches, utilizing the free-ratio model. Genes that showed values of Ka/Ks higher than 1 along the branch leading to finless porpoise were reanalyzed using the codon-based branch site tests implemented in PAML v4.9 (RRID:SCR_014932). The branch site model allowed ω to vary both among sites in the protein and across branches, and it was used to detect episodic positive selection.

### Gene expression analysis

The raw RNA-seq reads (21 RNA samples) of *N. a. asiaeorientalis* underwent quality control using SOAPnuke v2.0 (RRID:SCR_015025). Reads were filtered out if they had an N rate ≥0.01, low quality ≤20, or low quality rate ≥0.1 or contained index sequences. Subsequently, the clean reads were aligned to the YFP v2.0 and EFP v2.0 genomes utilizing the Hisat2 v2.1.0 (jztwsjrtswzRRID:SCR_015530) high-sensitivity model, excluding discordant pairs and mixed alignments. The alignment process involved setting a minimum insert size of 1 bp and a maximum insert size of 1,000 bp. We utilized featureCounts (RRID:SCR_012919) [84] and transcripts per million method to generate an estimated mapped read count matrix and calculate the gene expression level, respectively.

## Supplementary Material

giae067_Supplemental_File

giae067_GIGA-D-23-00359_Revision_1

giae067_GIGA-D-23-00359_Revision_2

giae067_GIGA-D-23-00359_Revision_3

giae067_GIGA-D-23_00359_Original_Submission

giae067_Response_to_Reviewer_Comments_Original_Submission

giae067_Response_to_Reviewer_Comments_Revision_1

giae067_Response_to_Reviewer_Comments_Revision_2

giae067_Reviewer_1_Report_Original_Submission Phillip A Morin, Ph.D. -- 1/10/2024

giae067_Reviewer_2_Report_Original_Submission Magnus Wolf -- 1/11/2024

giae067_Reviewer_2_Report_Revision_1 Magnus Wolf -- 5/21/2024

giae067_Reviewer_2_Report_Revision_2 Magnus Wolf -- 6/24/2024

## Data Availability

Raw sequencing data and genome assemblies in this study have been deposited in the NCBI database (BioProject ID PRJNA915046 and PRJNA859258). All supporting data and materials are available in the *GigaScience* database, GigaDB [85]. Results of repeat annotation, gene structure annotation, and gene functional annotation are available in the *Figshare* repository [86].

## Additional Files

**Supplementary Fig. S1**. Coverage depth of ONT long reads mapped across the 22 chromosomes of the YFP v2.0 genome and 23 chromosomes of the EFP v2.0 genome.

**Supplementary Fig. S2**. The BUSCO assessments display proportions.

**Supplementary Fig. S3**. A heatmap displaying Hi-C interactions (500-kb bins) within and between the chromosomes of YFP v2.0 and EFP v2.0.

**Supplementary Fig. S4**. Divergence distribution of repetitive elements in the genomes of YFP v2.0 and EFP v2.0.

**Supplementary Fig. S5**. Distribution of gene, coding sequence, exon and intron lengths, and exon number in the finless porpoise genome compared to other genomes.

**Supplementary Fig. S6**. Gene function annotation results in the 5 databases of NR, InterPro, KEGG, SwissProt, and KOG statistics Venn diagram in the genomes of YFP v2.0 and EFP v2.0.

**Supplementary Fig. S7**. Genome characteristics of YFP v2.0 and EFP v2.0 genomes.

**Supplementary Fig. S8**. Boxplots illustrating the gene expression levels in the centromere region and noncentromere region of YFP v2.0 and EFP v2.0.

**Supplementary Fig. S9**. KEGG enrichment of centromere genes of YFP v2.0 and EFP v2.0.

**Supplementary Fig. S10**. KEGG enrichment of genes located in SNP regions of YFP v2.0 compared to EFP v2.0.

**Supplementary Fig. S11**. Heatmaps illustrating the gene expression levels of the newfound genes in YFP v2.0 and EFP v2.0.

**Supplementary Fig. S12**. Species evolutionary topology.

**Supplementary Fig. S13**. The species tree of 26 manually selected species.

**Supplementary Table S1**. A summary of the sequencing data obtained for WGS libraries, PacBio HIFI library, ONT libraries, and Hi-C libraries is provided.

**Supplementary Table S2**. The length of chromosomes in finless porpoises.

**Supplementary Table S3**. Assembly statistics for 36 species of cetaceans.

**Supplementary Table S4**. Statistics of assembly quality and completeness evaluation using Merqury.

**Supplementary Table S5**. The mapping rate of HiFi, ONT, and Hi-C for YFP v2.0 and EFP v2.0 genome assemblies.

**Supplementary Table S6**. Total counts of telomere repeats in the YFP v2.0 genome.

**Supplementary Table S7**. Total counts of telomere repeats in the EFP v2.0 genome.

**Supplementary Table S8**. Summary of transposon element families in the YFP v2.0 and EFP v2.0 genomes based on various methods.

**Supplementary Table S9**. Statistics of classified repeat in the YFP v2.0 and EFP v2.0 genomes.

**Supplementary Table S10**. Comparison of predicted protein-coding genes in the YFP v2.0 genome with those in other representative cetacean genomes.

**Supplementary Table S11**. Comparison of predicted protein-coding genes in the EFP v2.0 genome with those in other representative cetacean genomes.

**Supplementary Table S12**. The evidence supporting the gene models of the YFP v2.0 genome.

**Supplementary Table S13**. The evidence supporting the gene models of the EFP v2.0 genome.

**Supplementary Table S14**. Statistics of genes with functional classification by various methods in YFP v2.0 and EFP v2.0 genomes.

**Supplementary Table S15**. Predicted centromere position of YFP v2.0 genome assembly.

**Supplementary Table S16**. Predicted centromere position of EFP v2.0 genome assembly.

**Supplementary Table S17**. Expression of predicted centromere genes in the YFP v2.0 genome.

**Supplementary Table S 18**. Expression of predicted centromere genes in the EFP v2.0 genome.

**Supplementary Table S19**. GO enrichment of predicted centromere genes in the YFP v2.0 genome.

**Supplementary Table S20**. KEGG enrichment of predicted centromere genes in the YFP v2.0 genome.

**Supplementary Table S21**. KEGG enrichment of predicted centromere genes in the EFP v2.0 genome.

**Supplementary Table S22**. The annotation summary of SNPs and indels detected between YFP v2.0 and EFP v2.0 genome assemblies.

**Supplementary Table S23**. KEGG enrichment analysis was conducted on genes located in SNP regions of the YFP v2.0 genome in comparison to the EFP v2.0 genome.

**Supplementary Table S24**. Newfound gene in YFP v2.0 and EFP v2.0 genomes.

**Supplementary Table S25**. Expression of newfound genes in the YFP v2.0 genome.

**Supplementary Table S26**. Expression of newfound genes in the EFP v2.0 genome.

**Supplementary Table S27**. GO enrichment of newfound genes in the YFP v2.0 genome.

**Supplementary Table S28**. KEGG enrichment of newfound genes in the YFP v2.0 genome.

**Supplementary Table S29**. GO enrichment of newfound genes in the EFP v2.0 genome.

**Supplementary Table S30**. KEGG enrichment of newfound genes in the EFP v2.0 genome.

**Supplementary Table S31**. Gene families are clustered.

**Supplementary Table S32**. Expanded genes families in YFP v2.0 and EFP v2.0 genomes.

**Supplementary Table S33**. Positively selected genes (PSGs) in the YFP v2.0 genome.

**Supplementary Table S34**. Positively selected genes (PSGs) in the EFP v2.0 genome.

**Supplementary Table S35**. Data for analysis are provided in this study.

**Supplementary Table S36**. RNA sample statistics.

## Abbreviations

bp: base pair; BLAST: Basic Local Alignment Search Tool; BUSCO: Benchmarking Universal Single-Copy Orthologs; CCS: circular consensus sequence; CNGBdb: China National GeneBank DataBase; Gb: gigabase; HMW: ultra-high molecular weight; Hi-C: chromosome conformation capture; HiFi: high fidelity; Mb: megabase; Mya: million years ago; NCBI: National Center for Biotechnology Information; ONT: Oxford Nanopore Technologies; OrthoDB: orthologous database; PacBio: Pacific Biosciences; RNA-seq: RNA sequencing; SMRT: single molecule real time; SNP: single nucleotide polymorphism.
