# Supplementary material for: Telomere-to-telomere gap-free genome assembly of the endangered Yangtze finless porpoise and East Asian finless porpoise
Source: Gigascience. 2024 Sep 16;13:giae067. doi: 10.1093/gigascience/giae067 (PMC11403816; doi:10.1093/gigascience/giae067)
Supplement: giae067_GIGA-D-23-00359_Revision_1 [file giae067_giga-d-23-00359_revision_1.pdf]

GigaScience

Telomere-to-telomere gap-free genome assembly of the endangered Yangtze finless porpoise and East Asian finless porpoise

--Manuscript Draft--

Manuscript Number:	GIGA-D-23-00359R1
Full Title:	Telomere-to-telomere gap-free genome assembly of the endangered Yangtze finless porpoise and East Asian finless porpoise
Article Type:	Research
Funding Information:	the National Key R&D Program of China (2021YFD1200304) — Doctor Danqing Lin
Abstract:	The Yangtze finless porpoise (Neophocaena asiaeorientalis asiaeorientalis, YFP) and the East Asian finless porpoise (Neophocaena asiaeorientalis sunameri, EFP) are two subspecies of the narrow-ridged finless porpoise that live in fresh and salt water, respectively. They serve as ideal models for the study of freshwater adaptation and evolution. The main objective of this study was to provide contiguous chromosome-level genome assemblies for YFP and EFP. Here, we generated and upgraded the genomes of YFP and EFP at the telomere-to-telomere level through the integration of PacBio HiFi long reads, ultra-long ONT reads and Hi-C sequencing data. The total sizes of the genomes are 2.48 Gb and 2.50 Gb, respectively. The scaffold N50 of two genomes was 125.12 Mb (YFP) and 128 Mb (EFP) with one contig for one chromosome. The telomere repeat and centromere position were clearly identified in both YFP and EFP genomes. Telomeric repeat units were detected at 85% and 90% of the chromosome at both ends in YFP and EFP genomes, respectively. In total, 5,480 new-found genes were detected in the YFP genome, including 56 genes located in the newly identified centromere regions. Additionally, synteny blocks, comparative genomes, phylogenetic relationships, gene family expansion and inference of selection were studied in connection with the genomes of other related mammals. Significant improvements were observed in genome contiguity, the number of scaffolds and gaps, and annotation compared to the first drafts of the YFP (GCA_003031525.2) and EFP (GCA_026225855.1) genome assemblies. The improvement of telomere-to-telomere gap-free reference genome resources support conservation genetics and population management for finless porpoises.
Corresponding Author:	Kai Liu, Ph.D<br>CAFS FFRC: Chinese Academy of Fishery Sciences Freshwater Fisheries Research Center<br>Wuxi, CHINA
Corresponding Author Secondary Information:	
Corresponding Author's Institution:	CAFS FFRC: Chinese Academy of Fishery Sciences Freshwater Fisheries Research Center
Corresponding Author's Secondary Institution:	
First Author:	Denghua Yin
First Author Secondary Information:	
Order of Authors:	Denghua Yin
	Chunhai Chen
	Danqing Lin
	Zhong Hua
	Congping Ying
	Jialu Zhang
	Chenxi Zhao

Powered by Editorial Manager® and ProduXion Manager® from Aries Systems Corporation

	Yan Liu
	Zhichen Cao
	Han Zhang
	Chenhe Wang
	Liping Liang
	Pao Xu
	Jianbo Jian
	Kai Liu, Ph.D
**Order of Authors Secondary Information:**	
**Response to Reviewers:**	Dear Dr. Hans Zauner,<br>Thanks for your kind consideration and comments regarding to our manuscript entitled “Telomere-to-telomere gap-free genome assembly of the endangered Yangtze finless porpoise and East Asian finless porpoise” (GIGA-D-23-00359). We have revised our manuscript (details are provided below) in accordance with all comments and suggestions from the reviewers. We hope the revised manuscript have been improved and meet the standard requirement. We look forward to hearing from GigaScience soon.<br><br>Sincerely yours,<br><br><br>Correspondence: Kai Liu<br>Freshwater Fisheries Research Center, Chinese Academy of Fishery Sciences, Wuxi 214081, China.<br>E-mail: liuk@ffrc.cn<br><br>Response to reviewer 1’s comments:<br>Reviewer #1: The authors have assembled new telomer-to-telomer reference genomes for two finless porpoise species, the fresh-water Yangtze finless porpoise and the oceanic East Asian finless porpoise. These genome assemblies appear to be ultra-high quality, with substantial description of the genomic features (repeats, genes, assembly characteristics), improvements over previous assemblies for the same species, and comparison of the genomes for some features including genomic structure, variation, novel genes and genes under selection. These represent substantial contributions to cetacean genomics and to understanding the evolution of these two sister species in their unique habitats.<br>The manuscript requires substantial revision to provide adequate methods for the reader to understand how the genomes were assembled and analyzed, and the figures need to be modified substantially to be useful. As they are, the legends do not adequately describe what's in many of the figures, the figures often try to present too much information in a single plot or set of plots, and the text is often too small to read. In my detailed comments below, I suggest ways to modify the figures and legends to make them more informative and useful.<br>The manuscript is generally well written, but requires some editing for standard English, and several sections repeat information presented in tables and figures, and could be edited to reduce length and focus on interpretation rather than just repetition. In particular, although the improvements in the new genome assemblies are substantial compared to previous assemblies, these improvements are a normal process in genome assembly, and not of great biological importance. I suggest reducing description and discussion of the upgrades to the genomes, and focus on the interesting characteristics of, and comparisons between, the two new genomes.<br>Response: Thanks for your valuable suggestions on our work. The comments are providing important guiding significance to our research. Based on the comments we received, detailed modifications are provided below. According to your advice, this manuscript was edited for proper English language, grammar, punctuation, spelling, and overall style by one native English speaker.<br>Specific comments (by line number)<br>Comment 1:28. change 'the chromosome both ends' to 'the chromosomes at both ends'

	Response: We are sorry for the mistake. We have changed 'the chromosome both ends' to 'the chromosomes at both ends' in the revised manuscript.<br><br>Comment 2:31. 'selection pressure' was not studied, only inference of selection.<br>Response: Thank you very much for your suggestion. We have revised 'selection pressure' to 'inference of selection'.<br><br>Comment 3:33. The accession ID given for YFP, GCF000442215, is for Lipotes vexillifer, not for the YFP genome.<br>Response: We are sorry for the mistake. We have changed 'GCF000442215' to 'GCA_003031525.2'<br><br>Comment 4:43. is there a word missing between 'clearly' and 'dorsal fin? Or, change from 'a clearly dorsal fin' to 'an obvious dorsal fin'.<br>Response: Thank you very much for your suggestion. We have revised 'a clearly dorsal fin' to 'an obvious dorsal fin'.<br><br>Comment 5:54. It's not clear what you mean by 'populations', since the comparisons are among species and subspecies. The term 'population' means something different from species and subspecies, so this section should be revised to use the appropriate terms.<br>Response: Thank you very much for your suggestion. We have revised the sentence 'Yang et al. identified significant genetic structure between the Indo-Pacific finless porpoise and the other two populations…' to 'Yang et al. identified significant genetic structure between either the Yangtze River population or the Yellow Sea population and the South China Sea population…' in the revised manuscript.<br><br>Comment 6:67. Insert 'a' before 'high-quality genome'<br>Response: We have inserted 'a' before 'high-quality genome' in the revised manuscript.<br><br>Comment 7:69. change 'cetacean' to 'cetaceans'<br>Response: We have change 'cetacean' to 'cetaceans' in the revised manuscript.<br><br>Comment 8:70. delete 'which was'<br>Response: We have deleted 'which was' in the revised manuscript.<br><br>Comment 9:71. The number of scaffolds for the previous version of the genome is misleading. The number given, 104, is based only scaffolds that are larger than the N50 length. According to the supplemental materials for Zhou et al. 2018, there were actually 97,387 scaffolds >100bp, 2179 scaffolds >2kb, and 423 scaffolds larger than the N90 size. If not all scaffolds are going to be counted, then it's important to specify which scaffolds were counted.<br>Response: Thank you for bringing this to our attention. We have made revisions in the description of the initial genome assembly of YFP. As reported on the NCBI website (https://www.ncbi.nlm.nih.gov/datasets/genome/GCF_003031525.1/), the assembly comprised 13,698 scaffolds, with a scaffold N50 of 6.3 Mb, excluding the minimum sequence length (100 bp) consideration.<br><br>Comment 10:80. change to 'a hotspot of genomic research, with extensive applications…'<br>Response: We have changed '…a hotspot genomic research fields now, extensive applications…' to '…a hotspot of genomic research, demonstrating extensive applications…'<br><br>Comment 11:81. insert 'and' between 'chicken' and 'fish'.<br>Response: We have inserted 'and' between 'chicken' and 'fish'.<br><br>Comment 12:99. change 'Based on' to 'In addition to'<br>Response: We have changed 'Based on the previously sequenced 62x PacBio HiFi and 85x Hi-C reads of the EFP, we generated 215 Gb (86x) ONT reads in this study' to 'In this study, we supplemented the existing dataset comprising 62 × PacBio HiFi and 85 × Hi-C reads of the EFP with an additional 215 Gb (86 ×) of ONT reads'.<br><br>Comment 13:116. What do the Merqury quality values mean? Are these from a range

(e.g., 1-100), or do they represent a log value? The reader can't interpret this without some context, and you can't assume they are familiar with every program.
Response: The quality values obtained from Merqury's k-mer analysis for YFP v2.0 and EFP v2.0 were calculated as 60.18 and 64.38, respectively. These values indicate a foundational accuracy level of 99.999%, confirming the high quality of our assembly for each component. It is essential to emphasize that Merqury quality values span from 0 to 255, with higher values denoting superior quality.
Ref:
Rhie, A, Walenz, B, Koren, S, et al. Merqury: reference-free quality, completeness, and phasing assessment for genome assemblies. Genome Biol 2020;21(1):245.

Comment 14:133-156. The whole section on gene prediction and annotation results could be reduced so that it repeats less of what is already presented in Table 1. Simple summary values such as average length of coding sequences don't need to be presented in the results, especially if they are not discussed in the discussion section as being important.
Response: Thank you for your suggestion concerning 'Gene prediction and annotation' section. We have revised the text with simple summary values at lines 163-172 in the revised MS to minimize redundancy and emphasize the main discoveries. The revised section now offers a succinct overview of the predicted protein-coding genes in the YFP and EFP genomes, supported by evidence and a comparison of length distributions. Supplementary Tables 9-12 contain further information, such as the average lengths of coding sequences, exons, and introns, as recommended.
We have changed 'In total, we predicted 23,139 and 23,101 protein-coding genes in the YFP and EFP genomes, respectively (Table 1), where the average length of coding sequence (CDS) was 1,507 bp and 1,510 bp, respectively. The average length of exon was both 175 bp, and the average length of intron was 6,082 and 6,107 bp, respectively (Supplementary Table S11-S12). The protein-coding genes in the YFP and EFP genomes were supported by at least one evidence with a CDS overlap ratio greater than 80% at a level of 99.96% and 99.95%, respectively (Supplementary Table S13-S14). ' to 'In total, the number of predicted protein-coding genes was 23,139 in the YFP v2.0 genome and 23,101 in the EFP v2.0 genome (Table 1). The roughly comparable number of predicted protein-coding genes for both T2T genomes is further evidence supporting the gene models (Supplementary Table S9-S12).'

Comment 15:138. The sentence starting 'For gene content assessment…' should be in the methods, not results. It's also not clear what you mean by '8 homologous proteins' (proteins, or databases, or ???). Also, change 'RNAseq data' to 'RNAseq datasets' (as in line 156).
Response: We apologize for any inconvenience caused. What I meant to convey is: "Utilizing 149,956 genes from 8 closely related cetacean species and transcriptomic sequencing data from 24 Yangtze finless porpoises as input files, the GeMoMa software was employed to conduct homology-based prediction analysis."

Comment 16:143-145. The sentence starting with 'The protein-coding genes in…' is difficult to understand, and I'm not sure it's important. Does it belong in the methods, or does it say something about the quality of the gene identification?
Response: We have revised the text with simple summary values at lines 163-172 in the revised MS to minimize redundancy and emphasize the main discoveries.
We have changed 'In total, we predicted 23,139 and 23,101 protein-coding genes in the YFP and EFP genomes, respectively (Table 1), where the average length of coding sequence (CDS) was 1,507 bp and 1,510 bp, respectively. The average length of exon was both 175 bp, and the average length of intron was 6,082 and 6,107 bp, respectively (Supplementary Table S11-S12). The protein-coding genes in the YFP and EFP genomes were supported by at least one evidence with a CDS overlap ratio greater than 80% at a level of 99.96% and 99.95%, respectively (Supplementary Table S13-S14). ' to 'In total, the number of predicted protein-coding genes was 23,139 in the YFP v2.0 genome and 23,101 in the EFP v2.0 genome (Table 1). The roughly comparable number of predicted protein-coding genes for both T2T genomes is further evidence supporting the gene models (Supplementary Table S9-S12).'

Comment 17:147. The wrong accession ID is given for the YFP, and I don't understand why the accession numbers are being presented here at all, since this whole section is about the new YFP and EFP genomes.

Powered by Editorial Manager® and ProduXion Manager® from Aries Systems Corporation

Response: We are sorry for the mistake. We have corrected the accession ID and changed the sentence to 'It is worth noting that the length distribution of gene models at the levels of genes, CDS, exons and introns showed a similar trend when compared to those of YFP v1.0 (GCA_003031525.2), EFP v1.0 (GCA_026225855.1) and Bottlenose Dolphin (GCF_011762595).'

Comment 18:155. 'Finally, 20, 589 (88.98%) and 20, 613 (89.23%)' of what? Genes? It's unclear what you are referring to for these numbers.
Response: We are sorry for the unclear description. We have changed 'Finally, 20,589 (88.98%) and 20,613 (89.23%) could be transcriptionally detected by the 24 RNA-seq datasets.' to 'Finally, 20,589 (88.98%) and 20,613 (89.23%) genes from the YFP v2.0 and EFP v2.0 genomes, respectively, were determined to be transcriptionally active based on the analysis of 24 RNA-seq datasets.'

Comment 19:162. What are 'repeat monomers'? is this a particular sequence that is unique to centromere repeats? One more sentence to describe what you are using to infer centromeres would be useful.
Response: We are sorry for the unclear description. We have added the description: 'In this study, we conducted predictions on repeat monomers within the YFP v2.0 and EFP v2.0 genomes, potentially constituting the centromere. The monomeric sequences vary in length from 99 to 201 bp, with the 144-bp, 150-bp, and 138-bp monomers being the most abundant. Centromeres are composed of more than one repeat monomer and are located within TE- and TR-enriched regions, which are areas with relatively lower gene density.'

Comment 20:165. Does 'newly identified centromere regions' refer to regions that are newly discovered in centromeres, or newly discovered in the new genome assemblies (compared to the old ones)? The sentence is a little confusing.
Response: We are sorry for the unclear description. We have changed 'In total, 235 and 237 genes were identified in the YFP and EFP candidate centromere region, respectively, while 56 and 20 genes of YFP and EFP were discovered in the newly identified centromere regions.' to 'A total of 235 and 237 genes were identified in the candidate centromere regions for YFP v2.0 and EFP v2.0, respectively, through predictions generated by centromere-finding software. Moreover, the newly discovered centromere regions for YFP v2.0 hosted approximately 56 genes, while only 20 genes were found in analogous regions for EFP v2.0. The "newly identified centromere regions" refer to specific areas identified in the genome that have been recently assembled but were not included in the previously published version. This discovery may suggest the presence of novel, previously uncharacterized centromere sites.'

Comment 21:179-180. please explain what 'stop-gain SNPs' and 'stop-loss SNPs' are. I think this means SNPs that result in the gain or loss of a stop codon, but I don't know the specific term (jargon).
Response: I apologize for any inconvenience caused. In genetics, these terms mean:
‘stop-gain SNPs’ (nonsense): A mutation that results in the premature termination of protein synthesis by introducing a premature stop codon, leading to the production of a truncated and often non-functional protein.
‘stop-loss SNPs’: A mutation where a stop codon is lost, allowing the translation machinery to continue synthesizing the protein beyond its intended termination point.

Comment 22:183. Why did you identify these 5 pathways from Figure 4C? Were they the only ones with functional variants, or are they just random examples from all of the ones in Figure 4C?
Response: We are sorry for the unclear description. We have modified the description of these pathways: 'KEGG enrichment analysis revealed that these genes were significantly (P value < =0.05) enriched in “NF-kappa B signaling pathway”, “complement and coagulation cascades”, “antigen processing and presentation” and “Intestinal immune network for IgA production”'.
In addition, we have enriched the discussion on these pathways: "The genes coding for the mutated regions of the YFP and EFP are widely enriched in immune-related pathways. This association may be intricately linked to the distinct pathogenic microorganisms unique to freshwater and seawater environments. Marine mammals exhibit a diminished histocompatibility complex (MHC) diversity attributed to decreased encounters with microparasitic diversity in their marine habitat relative to their terrestrial

Powered by Editorial Manager® and ProduXion Manager® from Aries Systems Corporation

origin. This phenomenon implies that mammals encounter distinct pathogenic pressures in varied ecological settings, potentially influencing the evolution of immune-related genes. [1]. Evolutionary analyses of the innate immune pattern recognition receptor (TLRs) in the YFP and the marine finless porpoise indicate that the YFP has undergone specific adaptive changes [2]. The microbial diversity and pathogenicity of freshwater and seawater environments vary, leading to distinct effects of pathogenic microorganisms on the organisms in these two types of environments [3]. Therefore, the YFP and EFP would be expected to undergo adaptive evolution to adapt to the pathogen stresses specific to their respective ecological environments, freshwater and seawater."

Ref

[1] Slade, R and McCallum, H. Overdominant vs. frequency-dependent selection at MHC loci. Genetics 1992;132(3):861–64.

[2] Tian, R, Chen, M, Chai, S, et al. Divergent Selection of Pattern Recognition Receptors in Mammals with Different Ecological Characteristics. J Mol Evol 2018;86(2):138-49.

[3] Lokesh, J and Kiron, V. Transition from freshwater to seawater reshapes the skin-associated microbiota of Atlantic salmon. Sci Rep 2016;25(6):19707.

Comment 23:186. Delete 'Here', and start the sentence with 'The genes coding…'
Response: Thank you very much for your suggestion. We have deleted 'Here' in the revised manuscript.

Comment 24:190. change 'will' to 'would be expected to'
Response: Thank you very much for your suggestion. We have changed 'will' to 'would be expected to'.

Comment 25:192-193. Delete the last sentence of the paragraph, as it is just repeating what is said above.
Response: Thank you very much for your suggestion. We have deleted the repeated sentence.

Comment 26:194. Delete 'Specifically,' and start the sentence with 'By comparing…' .
Response: Thank you very much for your suggestion. We have deleted 'Specifically,' and start the sentence with 'By comparing…' in the revised manuscript.

Comment 27:195. change 'YFP assembled 5480 new genes, while EFP assembled 1453…' to 'The YFP assembly included 5480 new genes, while the EFP assembly included 1453…'
Response: Thank you very much for your suggestion. We have corrected it in the revised manuscript.

Comment 28:198. Change 'discovered' to 'indicated'
Response: Thank you very much for your suggestion. We have changed 'discovered' to 'indicated' in the revised manuscript.

Comment 29:203. Change 'including' to 'include'
Response: Thank you very much for your suggestion. We have changed 'including' to 'include' in the revised manuscript.

Comment 30:209. Insert 'structural' or 'chromosomal' before 'conservation'
Response: Thank you very much for your suggestion. We have changed 'Synteny analysis demonstrated that YFP displayed a greater level of conservation than EFP' to 'Synteny analysis of the gene order between YFP v2.0 and EFP v2.0 revealed 907 large shared syntenic blocks, encompassing 89.59% (41428) genes, and 17 chromosomal rearrangements' in the revised manuscript.

Comment 31:209-214. Figure 1B should be its own figure, and changed to include only the synteny information. It's too small and too dense to be useful as it is. The full figure can be presented in supplemental materials. The text describing the figure could be expanded to describe what was found rather than just general observations of 'similar patterns' gene frequency, density, etc., or it could be reduced to just focus on what was different (specific chromosome rearrangements).
Response: Thank you for the valuable suggestions provided. We have separated

subfigures A and F from Figure 1B into individual new Figure 3. The remaining subfigures have been consolidated to create a new figure, now referred to as Supplemental Figure S7.

Comment 32:218. What do you mean by the 'last common ancestor'? This 'ancestor' doesn't exist, so is it a reconstructed ancestral genome?
Response: We apologize for the error in the description. We have changed 'When comparing the genomes of YFP and EFP with their last common ancestor, it was found that 843 gene families expanded while 98 contracted. 'to 'Upon comparing the genomes of YFP v2.0 and EFP v2.0 with their most recent common ancestor, it was observed that 843 gene families underwent expansion, while 98 gene families experienced contraction.'
When studying the evolution of gene families, the root of the phylogenetic tree is typically described using the term "Most Recent Common Ancestor" (MRCA), indicating the point of origin for these gene families during the evolutionary process, where they initially diversified into different genes. This term emphasizes the shared origin among different members of the gene family and their relationship to the common ancestor.

Comment 33:220. What does 'involved with statistical significance' mean?
Response: We are sorry for the unclear description. We have changed 'Out of the 215 expanded gene families identified in the YFP and EFP lineage, a total of 2,674 genes were found to be involved with statistical significance ($P<0.05$)' to 'Among the 215 expanded gene families identified in the YFP v2.0 and EFP v2.0 lineage, a total of 2,674 genes were determined to be significantly associated ($P<0.05$)'.

Comment 34:232-234. The first time these species are mentioned, the Latin name needs to be included. In the methods, only the Latin names are used, so the reader can't easily know what species the common names refer to (e.g. the Chinese white dolphin is called the Indo-Pacific humpback dolphin in the Society for Marine Mammalogy's taxonomy list).
Response: We are sorry for the unclear description. We have supplemented the Latin names in the sentence 'Neophocaena asiaeorientalis asiaeorientalis (Yangtze finless porpoise), Neophocaena asiaeorientalis sunameri (East Asian finless porpoise), Tursiops truncatus (Bottlenose dolphin), Orcinus orca (Killer whale), Lipotes vexillifer (Yangtze River dolphin), Physeter catodon (Sperm whale), Balaenoptera acutorostrata (Minke whale), Balaena mysticetus (Bowhead whale), Delphinapterus leucas (Beluga whale), Sousa chinensis (Indo-Pacific humpback dolphin)'.
In addition, we have added the common names in the methods '(a) Ornithorhynchus anatinus (Platypus) and Monodelphis domestica (Opossum) (163.7–185.9 Ma), (b) Homo sapiens (Human) and Mus musculus (Mouse) (81.3–91.0 MYA), (c) Balaena mysticetus and Balaenoptera acutorostrata (21.3–28.8 Ma) and (d) Sousa chinensis and Tursiops truncatus (2.0–3.8 Ma).'

Comment 35:237-239. What evidence is there for the statement that 'The evolution of DNA damage repair pathways implied the existence of additional triggers for genomic instability in the Yangtze River, including human activities such as wading projects, dredging and quarrying'? It seems odd to invoke pressure from the last century or less for evolutionary changes at the gene pathway level.
Response: We are sorry for the mistake. We have revised and expanded upon this paragraph: ' The selective pressure to evolve DNA damage repair pathways implied that the Yangtze finless porpoise might be experiencing increased threats to genome stability. The mechanism of DNA damage repair plays a crucial role in preserving genome integrity by enabling cells to identify and repair DNA damage, thereby averting the accumulation of harmful mutations [1]. In a comparative genomic analysis between the South China tiger and the Amur tiger, it was noted that genes related to DNA repair underwent positive selection in the South China tiger [2]. The observed phenomenon could be explained by the higher probability of genome instability in the temperate and subtropical habitats of the South China tiger. This may be linked to metabolites generated by intestinal microflora, which possess the ability to trigger DNA damage [3]. The stability of the genome or gene expression system in the Yangtze finless porpoise across different organs and life stages remains uncertain. However, one potential interpretation of this data is the suggestion that the Yangtze finless porpoise could be vulnerable to genomic instability triggers in the Yangtze River, such as water

Powered by Editorial Manager® and ProduXion Manager® from Aries Systems Corporation

pollutants, which may increase the likelihood of DNA damage [4]. Pollutants found in the Yangtze River possess the capacity to accumulate within the food chain, leading to cellular DNA damage and impacting the genome stability of the Yangtze finless porpoise [5-6]. This makes a compelling case for the improved conservation of the species and the development of more rigorous water pollution mitigation practices.'

Ref

[1] Chatterjee, N and Walker, G. Mechanisms of DNA damage, repair, and mutagenesis. Environ Mol Mutagen 2017;58(5):235-63.
[2] Zhang, L, Lan, T, Lin, C, et al. Chromosome-scale genomes reveal genomic consequences of inbreeding in the South China tiger: A comparative study with the Amur tiger. Mol Ecol Resour 2023;23(2):330-47.
[3] Puschhof, J and Sears, C. Microbial metabolites damage DNA. Science 2022;378(6618):358-59.
[4] Lv, W, Gu, H, He, D, et al. Polystyrene nanospheres-induced hepatotoxicity in swamp eel (Monopterus albus): From biochemical, pathological and transcriptomic perspectives. Sci Total Environ 2023;893:164844.
[5] Zhang, K, Qian, Z, Ruan, Y, et al. First evaluation of legacy persistent organic pollutant contamination status of stranded Yangtze finless porpoises along the Yangtze River Basin, China. Sci Total Environ 2020;710:136446.
[6] Xiong, X, Qian, Z, Mei, Z, et al. Trace elements accumulation in the Yangtze finless porpoise (Neophocaena asiaeorientalis asiaeorientalis)-A threat to the endangered freshwater cetacean. Sci Total Environ 2019;686:797-804.

Comment 36:256-257. The 'QIAGEN Blood & Cell Culture DNA Midi Kit' appears to be a DNA extraction kit, not a library preparation kit, so it cannot have been used to construct PacBio HiFi libraries. This section needs more detail to specify exactly what kits or methods were used to construct libraries, as the library methods impact downstream DNA sequence processing.

Response: Thanks for the suggestion. We have modified and enriched the methods: 'The PacBio HiFi library was constructed using SMRTbell Prep Kit 3.0 (Pacific Biosciences, USA) and subsequently sequenced on the PacBio Sequel II system in circular consensus sequence (CCS) mode. To collect data for the Hi-C library, the muscle tissues were first fixed in 1% formaldehyde (Sigma) for cross-linking and resuspended in lysis buffer. Then, MboI (NEB) restriction endonucleases were used to fragment the chromatin in the muscle to fragment DNA. The DNA fragments were captured by utilizing Streptavidin-coated magnetic beads (Thermo Fisher SCIENTIFIC) following biotin labeling and crosslinking using T4 DNA Ligase (ENZYMATICS). The Hi-C library was finally sequenced on a BGI MGISEQ platform.'

Comment 37:261. Cite a reference for the 'CTAB method'

Response: Thanks for bringing this to our attention. We have cited a reference for the 'CTAB method'.

Ref:

Yan, M, Wei, G, Pan, X et al. A method suitable for extracting genomic DNA from animal and plant-modified CTAB method. Agric Sci Technol, 2008, 9(2):39-41.

Comment 38:267. What filters were used to 'filter the Hi-C reads? Are there specific quality filters, or read trimming that are needed?

Response: Thank you for drawing our attention to this. To filter Hi-C reads, index reads were removed and reads were filtered using the following SOAPNUKE v2.0 parameters: N rate >= 0.01, low quality <= 20, low quality rate >= 0.1. Correspondingly, we have amended the relevant descriptions in the revised MS at line 406-408.

Comment 39:268. What do you mean by 'refine' the ONT reads? This is a meaningless term, and since the Perl scripts are described as 'self-designed', this provides no information about what was done. Likewise, the statement that 'ONT reads were subsequently corrected' is also too vague. What was actually done to 'correct' the reads?

Response: Thank you for your valuable suggestions. ONT reads were filtered based on a length < 5 kb and a quality value < 7. The Necat pipeline (v 20200119) was utilized for the enhancement of ONT reads. This was achieved through the application of error correction algorithms to evaluate quality scores, k-mer frequencies, and alignment methods. This process enhanced the accuracy of reading and generated refined results suitable for further analyses. Correspondingly, we have amended the

Powered by Editorial Manager® and ProduXion Manager® from Aries Systems Corporation

relevant descriptions in the revised MS at line 408-414.

Comment 40:272. There are a lot of programs used to process data, and while it's good that the actual parameters are given for some of them, it would be useful to also state whether the parameters were the default parameters or modified for these data, and describe what the parameter do (e.g., what does '-j 80 -s 80 -a 30' do?)
Response: Thank you for your valuable suggestions. The Purge-Haplotigs program was utilized to eliminate redundant contigs that exhibited similar sequences but distinct haplotypes, specifically targeting those with aligned coverage below 30%. This strategic approach significantly optimizes the assembly process by removing redundant information, thus improving the accuracy of genome assembly. Correspondingly, we have amended the relevant descriptions in the revised MS at line 416-420.

Comment 41:275. What do you mean by 'bridge the gaps in the gapless PacBio assembly? Do you mean that ONT reads and contigs were used to bridge gaps in the contig assembly in order to generate the gapless scaffolds?
Response: We are sorry for the unclear description. We have changed the sentence to ' Ultra-long ONT reads and contigs were used to generate gapless scaffolds through the LR_Gapcloser (v1.0) and TGSgapcloser (v 1.0.1) pipelines.'

Comment 42:281. Change 'were' to 'was'
Response: We have changed 'were' to 'was' in the revised manuscript.

Comment 43:283-4. insert 'the' before 'quartet pipeline' and change 'region' to 'regions'.
Response: We have inserted 'the' before 'quartet pipeline' and changed 'region' to 'regions'.

Comment 44:287. Change 'conducted' to 'used'
Response: We have changed '…RepeatModeler (v1.0.4) and LTR-FINDER (v1.0.7) were conducted to identify repetitive elements and annotate long terminal repeats, respectively.' to '…RepeatModeler (v1.0.4) was employed to identify repetitive elements, whereas LTR-FINDER (v1.0.7) was used for the specific annotation of long terminal repeats.' in the revised manuscript.

Comment 45:294. If RNAseq data were mapped to both assemblies, then change 'assembly' to 'assemblies'.
Response: We have changed 'assembly' to 'assemblies' in the revised manuscript.

Comment 46:299. I think 'combing' should be changed to 'combining'
Response: We have changed 'combing' to 'combining'.

Comment 47:320. Change 'Consistent phylogeny with previous study' to 'A phylogeny consistent with a previous study'
Response: We have changed 'Consistent phylogeny with previous study' to 'A phylogeny consistent with a previous study'.

Comment 48:327. Change 'Blast' to 'BLAST'
Response: We have changed 'Blast' to 'BLAST'.

Comment 49:328. It's unclear what you mean by 'defined syntenic blocks by MCscanX'. Do mean that 'syntenic blocks were defined using MCscanX'?
Response: We have changed 'defined syntenic blocks by MCscanX' to 'syntenic blocks were defined using MCscanX'

Comment 50:332. delete 'branch of', or change to 'analysis in the finless porpoise clade'.
Response: We have changed 'analysis in branch of finless porpoise' to 'analysis in the finless porpoise clade'

Comment 51:336. should the meaning of P-value≤0.01 be 'significantly expanded and contracted gene families', or is it either expanded OR contracted 'significantly expanded or contracted gene families'?
Response: We have changed 'significantly expanded and contracted gene families' to

Powered by Editorial Manager® and ProduXion Manager® from Aries Systems Corporation

'significantly expanded or contracted gene families'.

Comment 52:338. Change 'family' to 'families'
Response: We have changed 'family' to 'families'.

Comment 53:339. Change the section title to 'Gene positive selection analysis'
Response: We have changed the section title to 'Gene positive selection analysis'.

Comment 54:343. What filtering of alignments was done with Gblocks?
Response: Thank you for drawing our attention to this. Alignment refinement via Gblocks was utilized to remove inadequately aligned positions and divergent regions, thereby isolating conserved blocks from the multiple alignment. Correspondingly, we have amended the relevant descriptions in the revised MS at line 528-530.

Comment 55:345. delete the comma after 'branch'
Response: We have deleted the comma after 'branch'.

Comment 56:350. 'quality controlled' is vague. What was done to the reads to make them 'clean'? Please include the parameters or steps, e.g., reads were trimmed to remove adapter sequences and base calls with quality <30 (or whatever your parameters were).
Response: Thank you for your valuable suggestions. The raw RNA-seq reads underwent quality control using SOAPnuke (v2.0). Reads were filtered out if they had an N rate ≥ 0.01, low quality ≤ 20, low quality rate ≥ 0.1, or contained index sequences. Subsequently, the clean reads were aligned to the EFS v2.0 genome utilizing the Hisat2 high sensitivity model, excluding discordant pairs and mixed alignments. The alignment process involved setting a minimum insert size of 1 bp and a maximum insert size of 1000 bp. Correspondingly, we have amended the relevant descriptions in the manuscript at line 539-543.

Comment 57:357. change 'assembly' to 'assemblies'
Response: We have change 'assembly' to 'assemblies'.

Comment 58:359. delete 'two' (not needed because you name the species genomes in the same sentence)
Response: We have deleted 'two'.

Comment 69:372. What is 'undesired recombination'? Undesired by whom? I think you can just delete that word, and say the genome data can serve as a valuable resources for understanding… recombination, etc.
Response: We have deleted 'undesired '.

Comment 60:373. change 'Overall, this is the most continuous genome assembly to date' to 'Overall, these are the most continuous cetacean genome assemblies to date'
Response: We have changed 'Overall, this is the most continuous genome assembly to date' to 'Overall, these are the most continuous cetacean genome assemblies to date'.

Comment 61:375. change 'deepen the scientific issues' to 'deepen the scientific understanding of issues'
Response: We have changed 'deepen the scientific issues' to 'deepen the scientific understanding of issues'.

Comment 62:387. Authors' contributions: The initials vary between 2 and 3 initials to represent authors' names, but the names in the authors list are all only two names, so there should be only 2 initials for each.
Response: We have corrected the initials of each name to 2. ' K.L., J.J. and P.X. designed and conceived the study. D.Y., C.Y. and J.Z. collected and prepared the samples. C.C. and C.Z. performed the data analysis. D.Y., C.C. and J.J. wrote the manuscript with significant contributions from Y.L., Z.C., H.Z., C.W. and L.L. K.L., Z.H. and D.L. provided the financial support. All authors read and approved the final version of the manuscript.'

Comment 63: Table 1. In the legend, provide the accession ID's for each genome

Powered by Editorial Manager® and ProduXion Manager® from Aries Systems Corporation

version in the note below the table.
It's not clear why there is "C:" in front of each percentage for the two BUSCO lines. I think it would be clearer to remove "C:" from each number, put an asterisk (*) after BUSCO, and in the note, write "* percentage of complete BUSCO evaluation".
Response: Thank you for your valuable suggestions. We have provided the accession IDs for each genome except for the finless porpoise v2.0 genome assembly, as it is under control by NCBI but not yet public. Additionally, we have replaced "C:" with "BUSCO*" in the legend of Table 1, and mentioned "BUSCO*" as the percentage of complete BUSCO evaluation.

Figures.
Comment 64: Figure 1 contains too many unrelated figures, and too dense information, some of which is never discussed in the paper. I suggest that Figure 1 should include only A and F, B should be its own figure, and C,D,E should be moved to supplemental materials.
For the new figure (B), only the rings A (chromosome length) and F (syntenic blocks) should be included, as the others are too tiny and dense to be useful to the reader. Those can be put into supplemental materials with better descriptions to allow the reader to see the detail and to explain the colors and scale. All colors within the figures need to be explained or include a color legend.
Response: Thank you very much for your suggestion. We have separated subfigures A and F from Figure 1B into individual new Figure 3. The remaining subfigures have been consolidated to create a new figure, now referred to as Supplemental Figure S7.

Figure 1. Sample site and Genome assessment of YFP v2.0 and EFP v2.0.
A: Location distribution and sampling site of the YFP and EFP.
B：Proportions of genes that could be functionally annotated and transcriptionally detected in YFP v2.0 and EFP v2.0.

Figure 3. Synteny analysis of YFP v2.0 and EFP v2.0 genomes: A) chromosomes scale, Unit length is Mb; B) syntenic blocks between YFP v2.0 and EFP v2.0.

Comment 65: Figure 2. There is no visible "ruler" in the figure (but described in the legend).
The legends for A and B repeat most of the information. They can be combined to reduce repetition, e.g.,
Structure of T2T and gap-free chromosomes in A) Yangtze finless porpoises v2.0, and B) East Asian finless porpoises (v2.0). All 21+Xchromosomes are drawn to scale and the ruler indicates chromosome length. Triangles indicate the presence of telomere sequence repeats. Circles represent the locations of centromeric regions. The gap positions in the v1.0 genome assemblies are marked with squares to the right of the chromosome in the v2.0 genome assemblies.
Response: We have added "ruler" to the figure and combined the legends of A and B according to your helpful suggestion: 'Structure of T2T and gap-free chromosomes in A) YFP v2.0, and B) EFP v2.0. All 21+X/Y chromosomes are drawn to scale and the ruler indicates chromosome length. Triangles indicate the presence of telomere sequence repeats. Circles represent the locations of centromeric regions. The gap positions in the v1.0 genome assemblies are marked with squares to the right of the chromosome in the v2.0 genome assemblies'.

Comment 66: Figure 3. A and B are described as 'Heatmaps', but they appear to be box plots, not heatmaps. They are only described in one sentence in the text, so I suggest that they could be moved to supplemental materials, and include only C and D in the figure.
In C and D, the legend says "GO enrichment analysis of genes", but the figure is not showing analysis, but results. Please describe the plot and results, e.g., number of genes in centromere regions associated with GO pathways" (that may not be correct, but I'm not sure what the figure is showing, so it needs to be described better).
Figure 4 D and E could be added to Figure 3 to show all of the same types of plots in one figure instead of splitting data into different figures.
Response: Thank you for your suggestion. Inspired by your guidance, We have made the following adjustments to Figure 3：
A. Figures 3A and 3B have been relocated to the supplemental materials and re-titled as follows: Supplementary Figure S8 Box plots illustrating the gene expression levels

Powered by Editorial Manager® and ProduXion Manager® from Aries Systems Corporation

	in the centromere region and non-centromere region of YFP v2.0 and EFP v2.0. Corresponding gene expression data are presented in Supplementary Tables S16 and S17.<br>B. We sincerely apologize for the previous error in presenting the enrichment analysis in Figures 3C and 3D. In the revised manuscript, we have corrected this and now display the revised enrichment analysis results in the supplemental materials as: Supplementary Figure S9 KEGG enrichment of centromere genes of YFP v2.0 and EFP v2.0. Additionally, the KEGG enrichment analysis are presented in Supplementary Tables S19 and S20.<br>C. We extend our sincere apologies for the earlier error regarding the presentation of the enrichment analysis in Figure 4D and 4E. In the revised manuscript, we have made the necessary corrections by removing Figure 4D and 4E. The updated enrichment analysis results are now appropriately presented in the supplemental material, specifically in Supplementary Tables S26 to S29.<br><br>Comment 67: Figure 4.<br>A. what do the numbers next to the color legend represent? Is that the maximum number of SNPs per MB window? Is each color a range of values?<br>C. I don't understand what you mean by genes located in the Indel region or in the SNP region. What are the Indel and SNP 'regions'? These terms have not been used anywhere else or defined.<br>D-E. Consider moving to Figure 3. Are these 'new-found' genes relative to the old (V1) versions of the genomes, or genes that are unique to each species?<br>Response: I apologize for any inconvenience caused.<br>A. To improve the clarity of the presentation, we have made the following revisions to Figure 4 in the revised manuscript: the new Figure 4 now only retains sub-figures A and B; sub-figures C-E have been removed and replaced with the results of the KEGG enrichment analysis, which are now presented in Supplementary Figure S10 and Table S22.<br>B. The numerical values adjacent to the color legend indicate the count of SNPs/InDels per megabase (MB) window, where the gray legend representing zero. Each color corresponds to a specific range of values. For instance, in Figure 4A, the initial blue legend represents the range from 1 to 4440.<br>C. To address your inquiries regarding the terms "genes located in the Indel region" and "genes located in the SNP region," allow me to provide clarification:<br>Following the detection of SNPs (single nucleotide polymorphisms) and InDels (insertions or deletions), we employed Annovar for functional annotation. This process evaluates how these genetic variations might impact gene structure. The phrases in question describe the spatial relationship between genes and the aforementioned genetic alterations:<br>Indel Region: An Indel refers to a segment of DNA that has been inserted or deleted within the genome. When we mention a gene located in an Indel region, it indicates that the gene is situated at the site of such an insertion or deletion event.<br>SNP Region: A SNP represents a variation involving a single nucleotide within the genome. If a gene is described as being in a SNP region, it suggests that the gene contains one or more SNPs.<br>In summary, these terms denote the proximity of genes to genetic variations that may have functional implications.<br>D. The 'new-found' genes mentioned in this manuscript are relative to the old (V1) versions of the genomes, and the associated gene functional enrichment results are presented in Supplementary Tables S26 to S29.<br>Ref:<br>Wang, K, Li, M and Hakonarson, H. ANNOVAR: functional annotation of genetic variants from high-throughput sequencing data. Nucleic Acids Res 2010;38(16):e164.<br><br>Comment 68: Figure 5.<br>A. "Ma: Million years ago" can be deleted, as "Ma" is not used in the figure.<br>B. what are the units for the scale? Is this a count of gene families?<br>C-D. define what "Rich Factor" means. What do the colors mean?<br>E. There was no description of E, and it is too small to read. I suggest moving it to supplemental materials to allow it to be described in detail and large enough for the reader to see if interested.<br>Response: We have retained A and B of Figure 5 and treated C, D, E as a separate Figure 6.

Powered by Editorial Manager® and ProduXion Manager® from Aries Systems Corporation

A."Ma" is used in the Figure 5A.
B. The units of the scale are "millions of years ago," which are used to represent the divergence times between species.
C-D. In the context of KEGG enrichment analysis, the "Rich Factor" is a measure that represents the ratio of the number of candidate genes annotated to a specific pathway to the total number of genes annotated to that pathway. It is an indicator of how significantly the differentially expressed genes are enriched within a particular KEGG pathway compared to the whole genome background. A higher Rich Factor implies a greater degree of enrichment, suggesting that the pathway is more relevant to the biological changes observed in the study.
The colors in a KEGG enrichment plot typically represent the significance of the enrichment. They are often associated with the q-value, which is a corrected p-value that adjusts for multiple hypothesis testing. The color intensity or shade—commonly ranging from blue to red—indicates the level of significance, with darker shades or redder colors signifying more significant enrichment (lower q-values). This visual representation helps researchers quickly identify pathways that are potentially important in the biological context of their study.
To summarize, the "Rich Factor" is a measure of enrichment significance, and the colors in a KEGG enrichment plot are a visual representation of the statistical significance of the enrichment, with redder colors indicating more significant enrichment.
E. To present the information more clearly, we have replaced Figure 5E with Figure 6C and 6D.

Figure 5. Genome evolution of YFP v2.0 and EFP v2.0.
A: Divergence time between YFP v2.0 and EFP v2.0, and number of expanded and contracted gene families. green and red numbers indicate gene family expansions and contractions, respectively. MRCA: Most Recent Common Ancestor. Ma: Million years ago.
B: A comparison of gene families associated with orthologs and paralogs in YFP v2.0 and EFP v2.0, and other 24 mammal species.

Figure 6. Functional Enrichment of genes.
Significant A) KEGG and B) GO enrichment of expanded gene families in YFP and EFP lineage.
KEGG enrichment analysis of positively selected genes in C) YFP and D) EFP, respectively.

Reviewer #2: In this work, Yin et al. present two newly assembled genomes of two narrow-ridged finless porpoise (Neophocaena asiaeorientalis) subspecies (Yangtze, YFP and East Asia, EFP) as well as a comprehensive report on its quality and differences in terms of genotype changes related to their different habitats. I overall enjoyed reading this manuscript, I did not find too many downfalls in their methods, and I think these two genomes are of the highest quality and one of if not the most continuous reference genomes for cetaceans published thus far. Apart of this, however, I found several downfalls of the manuscript text itself which leads me to conclude that a major revision is needed for this manuscript to be considered for publication in GigaScience. I will first talk about general issues, point out the few downfalls within their methods and then provide a more detailed list of text issues which may help the authors to revise the manuscript.
Response: Thank you very much for your comments on our manuscript. The comments are all valuable and very helpful for revising and improving our paper. Based on the comments we received, detailed modifications are provided below.

General issues:
Comment 1: The text of the manuscript has multiple grammatical errors and I invite the authors to revise the language of the manuscript critically and carefully. I will list the errors I found down below but will not guarantee that I found everything. Furthermore, many statements made by the authors come without a proper citation which I will also highlight down below.
Response: We value your correction of language and citation errors. In the revised version, we have double-checked the paper for grammar, spelling, formatting, and citation issues and corrected them.

	Comment 2: The "Results and Discussion" part is missing a discussion. The authors invest the vast majority of this part describing their results and do not attempt to contextualize their results with recent literature. For example, the authors could have compared the quality of these reference genomes and annotations to other cetacean genomes to emphasize their superior quality. Furthermore, the authors did find, through various analyses, genes that might have played a role in their adaptions to different habitats but do not describe what is known about these genes and gene categories and do not describe how these gene might have help in their adaption. Also, the few sentences that do mention these possibilities come without proper citations, again.<br>Response: Thank you very much for your suggestion. We have added detailed description about genes with proper citations, which can be found in comment 23,29,30 and 31.<br><br>Comment 3: The material and method section is missing details. I assumed to find them in some sort of supplementary methods or data repository but failed to find them. If these details are indeed missing, I strongly suggest to provide more details in one of both ways (supplementary methods or within a data repository). I'll list the missing parts down below. Furthermore, the data repository made by the authors on figshare does lack a proper commentary README file and interested scientists are left to figure out the content of these files by themselves. I would strongly recommend providing an extensive README file for this repository to assure reproducibility.<br>Response: We have made revisions based on your suggestions provided. An enhanced version of the documentation file has been revised and is now accessible on the Figshare database. The documentation file contains the following contents:<br>For more details, please visit the Figshare website: https://figshare.com/s/1fc632fd4f3cab36b776.<br><br>Methods:<br>Comment 4: One part that is entirely missing in the Methods is the description of how SNPs were generated. Hence, I cannot evaluate the quality of their calls. I highly recommend adding the respective description because otherwise, these steps are not reproducible.<br>Response: Thank you for your insightful recommendations. Single nucleotide polymorphisms (SNPs) and insertions/deletions (InDels) were identified using methods similar to those previously...
**Additional Information:**	
**Question**	**Response**
Are you submitting this manuscript to a special series or article collection?	No
**Experimental design and statistics**<br><br>Full details of the experimental design and statistical methods used should be given in the Methods section, as detailed in our Minimum Standards Reporting Checklist. Information essential to interpreting the data presented should be made available in the figure legends.<br><br>Have you included all the information requested in your manuscript?	Yes
**Resources**	Yes

Powered by Editorial Manager® and ProduXion Manager® from Aries Systems Corporation

A description of all resources used, including antibodies, cell lines, animals and software tools, with enough information to allow them to be uniquely identified, should be included in the Methods section. Authors are strongly encouraged to cite Research Resource Identifiers (RRIDs) for antibodies, model organisms and tools, where possible.<br><br>Have you included the information requested as detailed in our Minimum Standards Reporting Checklist?	
**Availability of data and materials**<br><br>All datasets and code on which the conclusions of the paper rely must be either included in your submission or deposited in publicly available repositories (where available and ethically appropriate), referencing such data using a unique identifier in the references and in the “Availability of Data and Materials” section of your manuscript.<br><br>Have you have met the above requirement as detailed in our Minimum Standards Reporting Checklist?	Yes

Manuscript Click here to access/download;Manuscript;Manuscript.docx

Telomere-to-telomere gap-free genome assembly of the endangered Yangtze finless porpoise and East Asian finless porpoise

Denghua Yin[1†], Chunhai Chen[2†], Danqing Lin[1†], Zhong Hua[1], Congping Ying[3], Jialu Zhang[1], Chenxi Zhao[2], Yan Liu[1], Zhichen Cao[4], Han Zhang[4], Chenhe Wang[2], Liping Liang[2], Pao Xu[1,3], Jianbo Jian[2,*] and Kai Liu[1,3,4,*]

[1]Key Laboratory of Freshwater Fisheries and Germplasm Resources Utilization, Ministry of Agriculture and Rural Affairs, Freshwater Fisheries Research Center, Chinese Academy of Fishery Sciences, Wuxi 214081, China.

[2]BGI Genomics, BGI-Shenzhen, Shenzhen 518083, China.

[3]Wuxi Fisheries College, Nanjing Agricultural University, Wuxi 214081, China.

[4]National Demonstration Center for Experimental Fisheries Science Education, Shanghai Ocean University, Shanghai 201306, China.

[†]Authors contributed equally to this work.

[*]Correspondence address: E-mail: jianjianbo@bgi.com, liuk@ffrc.cn.

Abstract

The Yangtze finless porpoise (*Neophocaena asiaeorientalis asiaeorientalis*, YFP) and the East Asian finless porpoise (*Neophocaena asiaeorientalis sunameri*, EFP) are two subspecies of the narrow-ridged finless porpoise that live in fresh and salt water, respectively. They serve as ideal models for the study of freshwater adaptation and evolution. The main objective of this study was to provide contiguous chromosome-level genome assemblies for YFP and EFP. Here, we generated and upgraded the genomes of YFP and EFP at the telomere-to-telomere level through the integration of PacBio HiFi long reads, ultra-long ONT reads and Hi-C sequencing data. The total sizes of the genomes are 2.48 Gb and 2.50 Gb, respectively. The scaffold N50 of two genomes was 125.12 Mb (YFP) and 128 Mb (EFP) with one contig for one chromosome. The telomere repeat and centromere position were clearly identified in both YFP and EFP genomes. Telomeric repeat units were detected at 85% and 90% of the chromosome at both ends in YFP and EFP genomes, respectively. In total, 5,480 new-found genes were detected in the YFP genome, including 56 genes located in the newly identified centromere regions. Additionally, synteny blocks, comparative genomes, phylogenetic relationships, gene family expansion and inference of selection were studied in connection with the genomes of other related mammals. Significant improvements were observed in genome contiguity, the number of scaffolds and gaps, and annotation compared to the first drafts of the YFP (GCA_003031525.2) and EFP (GCA_026225855.1) genome assemblies. The improvement of telomere-to-telomere gap-free reference genome resources support conservation genetics and population management for finless porpoises.

Keywords: telomere-to-telomere; genome assembly; Yangtze finless porpoise; gap-free; HiFi sequencing; Hi-C sequencing

Introduction

Finless porpoises (*Neophocaena* spp.) are small toothed whales capable of inhabiting freshwater (Yangtze River) and saltwater (coastal waters of southern and eastern Asia) environments [1, 2]. They are characterized by a blunt, rounded head, an equal width upper and lower jaw, and lack of an obvious dorsal fin [3, 4]. Based on morphological characteristics, geographic distribution and molecular genetic evidence, it is generally believed that the finless porpoise can be divided into two species, namely the Indo-Pacific finless porpoise (*N. phocaenoides*) and the narrow-ridged finless porpoise (*N. asiaeorientalis*) [5, 6]. In China, there exist two subspecies of the narrow-ridged finless porpoise: one is the freshwater Yangtze finless porpoise (*N. a. asiaeorientalis*, YFP), which exclusively inhabits the middle and lower reaches of the Yangtze River and adjacent Dongting and Poyang lakes. The other subspecies is the marine East Asian finless porpoise (*N. a. sunameri*, EFP), which can be found in the coastal waters of the Yellow Sea and Bohai Sea, and in the northern waters of the East China Sea [5, 6] (Figure 1A).

The investigation of the evolutionary origins and conservation genetics of finless porpoises is a pressing concern for scientists. Yang *et al.* identified significant genetic structure between either the Yangtze River population or the Yellow Sea population and the South China Sea population by analyzing the sequences of mtDNA control region of finless porpoises in Chinese waters [7]. This result was supported by subsequent mtDNA sequences, nuclear DNA microsatellites, single nucleotide polymorphisms (SNPs) and MHC loci [8-12]. The genetic diversity of East Asian finless porpoises surpasses that of the other two populations, indicating it as the likely center of origin for this species [7]. Zheng *et al.* analyzed the sequences of the mtDNA control region of seven local populations of Yangtze finless porpoises in the middle and lower reaches of the Yangtze River, and found that the overall level of genetic diversity was low. Notably, the downstream population showed richer genetic variation than the midstream population. Such a genetic pattern reflects, to some extent, the marine origin and evolutionary history of the Yangtze population [13]. Based on genomic analysis of finless porpoise populations, significant genetic structure was identified among the three populations, indicating local adaptive evolution and emphasizing the evolutionary distinctiveness and conservation significance of the Yangtze finless porpoise [14].

Availability of a high-quality genome assembly is not only critical for the genomic studies of

finless porpoises, but also would be a valuable resource for comparative genomics and evolutionary studies of cetaceans. The first draft of the YFP genome assembly (GCF_003031525.2) was published in 2018 with a size of 2.3 Gb generated by short-read sequencing on the Illumina HiSeq 2000 platform [14]. The assembly comprised 13,698 scaffolds, with a scaffold N50 of 6.3 Mb, excluding the minimum sequence length (100 bp) consideration. Although progress had been made in genome-wide studies of YFP through the availability of this draft, such as immune changes with age and gene expression profiles in different habitats [15, 16], the lack of chromosomal information limits the potential application of the data. Recent advances in ultra-long ONT and PacBio HiFi sequencing technologies, as well as assembly algorithms, have facilitated the development of telomere-to-telomere (T2T) genome assemblies. The completion of the T2T human genome sequence and the full Y chromosome sequence represents a significant milestone in the field of human genomics research [17, 18]. The T2T genome has emerged as a hotspot genomic research, demonstrating extensive applications to other animal species like chicken and fish [19, 20]. T2T genome assemblies can serve as a benchmark with enhanced accuracy and comprehensive genomic references for future studies, facilitating the confident identification and annotation of genes, regulatory elements, and other functional components.

A high-quality genome can support finer genetic analyses, such as the length, number, and distribution of key indicators of inbreeding, such as ROH and IBD [21, 22], and these analyses are more urgent than ever for the conservation of endangered species. In this study, we utilize PacBio HiFi, Nanopore and Hi-C data to generate improved telomere-to-telomere gap-free genomes of Yangtze and East Asian finless porpoises. We compare the quality of newly drafted assemblies with previously available versions and explore the synteny blocks, comparative genomes, phylogenetic relationships, gene family expansion and inference of selection in relation to the several mammals. Finless porpoises serve as a representative example for comprehending speciation, evolution, and population genetics. The high-quality chromosomal-level references help elucidate ecological and aquatic adaptation mechanisms in cetaceans.

Results and Discussion

Genome sequencing and gap-free assembly

We integrated PacBio HiFi long reads, ultra-long ONT reads and Hi-C sequencing data to

generate chromosome-level genome assemblies for YFP and EFP. Henceforth, we refer to this new T2T genome as 2.0 and the original genome as 1.0. We generated approximately 123 Gb (49 ×) PacBio HiFi reads, 279 Gb (111 ×) Hi-C reads and 225 Gb (90 ×) ONT reads for YFP v2.0 (Supplementary Table S1). In this study, we supplemented the existing dataset comprising 62 × PacBio HiFi and 85 × Hi-C reads of the EFP [23] with an additional 215 Gb (86 ×) of ONT reads (Supplementary Table S1). The YFP v2.0 genome assembly comprised 23 scaffolds, with both contig N50 and scaffold N50 measuring 125.12 Mb (Table 1). These scaffolds were assembled into 21 autosomal chromosomes, one X chromosome, and one mitochondrial chromosome, resulting in a final assembly size of 2.48 Gb (Supplementary Table S2). Similarly, the EFP v2.0 genome assembly consisted of 24 contigs or scaffolds, with both contig N50 and scaffold N50 measuring 128.00 Mb (Table 1). These scaffolds were also assembled into 21 autosomal chromosomes, one X+Y chromosome, and one mitochondrial chromosome, with a final assembly size of 2.50 Gb (Supplementary Table S2).

We have significantly enhanced the contiguity, accuracy and completeness of the YFP v1.0 (GCA_003031525.2) and EFP v1.0 (GCA_026225855.1) assemblies. The contig N50 values of the two genomes were consistent with their respective chromosome lengths, and a single contig represented a complete chromosome, which is notably superior to the recently published finless porpoise genomes (e.g., 125.12 Mb vs. 0.09 Mb for YFP and 128.00 Mb vs 84.69 Mb for EFP) (Table 1). The YFP v1.0 and EFP v1.0 genome assemblies had 52,647 and 28 gaps, respectively, whereas the new assembly process produced a gap-free genome, obviously improving contiguity (Figure 2A and Figure 2B). The quality values obtained from Merqury's k-mer analysis [24] for YFP v2.0 and EFP v2.0 were calculated as 60.18 and 64.38, respectively. (Supplementary Table S3). These values indicate a foundational accuracy level of 99.999%, confirming the high quality of our assembly for each component. Moreover, the mapping rates of RNA reads to the two genome assemblies were 95.47% and 95.42%, respectively, whereas they were 70.01% and 92.34% for previously published assemblies (Supplementary Table S4). Moreover, the mapping results of ONT long reads with a mapping quality >20 indicate a read coverage of 99.99% for YFP v2.0 and EFP v2.0 whole genome (Supplementary Figure S1). Using Benchmarking Universal Single-Copy Orthologs (BUSCO) evaluation, it is suggested our results represent 95.20% completeness of YFP and 95.30% completeness of EFP (Table 1 and Supplementary Figure S2). Additionally, we have

identified/predicted both the telomeric repeat and centromere candidate region in YFP v2.0 and EFP v2.0 (Figure 2A, Figure 2B and Supplementary Table S5-S6). Telomeric repeat units of YFP v2.0 genome were detected at both ends of 18 chromosomes and at one end of 3 chromosomes. Similarly, telomeric repeat units of EFP v2.0 genome were detected at both ends of 20 chromosomes and at one end of 2 chromosomes. Telomeric repeat units were detected at 85% and 90% of the chromosome at both ends in YFP v2.0 and EFP v2.0 genomes, respectively. Our estimations of the centromere region are novel and were not present in YFP v1.0. Finally, we also utilized Hi-C data for chromosome sequencing and orientation, resulting in a comprehensive Hi-C matrix that effectively illustrates genome-wide interactions (Supplementary Figure S3). These findings suggest that the new genome assemblies of YFP and EFP represent a significant improvement over the previously published genome assembly, achieving near telomere-to-telomere (T2T) completeness.

Gene prediction and annotation

Two strategies including *de novo* and homolog-base methods were applied to annotate repeat elements. The genomes of YFP v2.0 and EFP v2.0 contained 1,058.09 Mb (42.54%) and 1,069.10 Mb (42.80%) of repetitive sequences, respectively (Supplementary Figure S4 and Table S7). Long interspersed nuclear elements (LINEs) were the most abundant type of annotated transposable elements, accounting for 38.88% and 39.10% of the genomes of YFP v2.0 and EFP v2.0, respectively (Supplementary Table S8). In total, the number of predicted protein-coding genes was 23,139 in the YFP v2.0 genome and 23,101 in the EFP v2.0 genome (Table 1). The roughly comparable number of predicted protein-coding genes for both T2T genomes is further evidence supporting the gene models (Supplementary Table S9-S12). It is worth noting that the length distribution of gene models at the levels of genes, CDS, exons and introns showed a similar trend when compared to those of YFP v1.0 (GCA_003031525.2), EFP v1.0 (GCA_026225855.1) and Bottlenose Dolphin (GCF_011762595) (Supplementary Figure S5). In the predicted gene models of YFP v2.0 and EFP v2.0, the BUSCO analysis identified 97.5 % and 97.6% complete conserved single copy mammalian genes (odb10), respectively (Table 1 and Supplementary Figure S2). In total, 22,263 (96.21%) gene models in the YFP v2.0 genome and 22,224 (96.20%) gene models in the EFP v2.0 genome were annotated in at least one database (NR, SwissProt, KEGG, KOG, TrEMBL, Gene Ontology and InterPro) (Figure 1B and Table 1), whereas 71.22% (16,480) of YFP v2.0 genes and 71.24% (16,457) of EFP v2.0 genes are annotated in five functional databases (NR,

SwissProt, KEGG, KOG and InterPro) (Supplementary Figure S6 and Table S13). Finally, 20,589 (88.98%) and 20,613 (89.23%) genes from the YFP v2.0 and EFP v2.0 genomes, respectively, were determined to be transcriptionally active based on the analysis of 24 RNA-seq datasets (Figure 1B). In conclusion, our annotation of two gene sets of reliably high quality has established a robust foundation for further research.

Analysis of centromere related genes

The centromere plays a necessary role in cell division for eukaryotes. The intrinsic mechanisms underlying the evolution of centromere structure among different species has not been fully revealed due to the challenge of assembling highly repetitive sequences [25]. In this study, we conducted predictions on repeat monomers within the YFP v2.0 and EFP v2.0 genomes, potentially constituting the centromere (Figure 2A, Figure 2B and Supplementary Table S14-S15). The monomeric sequences vary in length from 99 to 201 bp, with the 144-bp, 150-bp, and 138-bp monomers being the most abundant. Centromeres are composed of more than one repeat monomer and are located within TE- and TR-enriched regions, which are areas with relatively lower gene density (Supplementary Figure S7). A total of 235 and 237 genes were identified in the candidate centromere regions for YFP v2.0 and EFP v2.0, respectively, through predictions generated by centromere-finding software. Moreover, the newly discovered centromere regions for YFP v2.0 hosted approximately 56 genes, while only 20 genes were found in analogous regions for EFP v2.0. The "newly identified centromere regions" refer to specific areas identified in the genome that have been recently assembled but were not included in the previously published version. This discovery may suggest the presence of novel, previously uncharacterized centromere sites. We further compared the genes located in the centromere and non-centromere regions in the genomes of YFP v2.0 and EFP v2.0, respectively, and found that there were no significant differences in the expression patterns of these genes in different transcripts (Supplementary Figure S8 and Supplementary Table S16-S17). Additionally, we analyzed the functional enrichment of genes located in the centromere regions of the two genomes. These genes were mainly associated with Keratin filament (GO:0045095), Intermediate filament (GO:0005882), Gamete generation (GO:0007276) and Channel activity (GO:0015267), as indicated by GO enrichment (Supplementary Table S18). These findings align with those produced by analysis using the KEGG database, and highlight the essential role of centromeres in chromosome segregation and positioning

(Supplementary Figure S9 and Table S19-S20). Centromeres provide structural support to the nucleus, ensuring chromosome integrity, and are crucial in mitosis and meiosis. Moreover, centromeric regions may interact with cell cycle regulation, cell division signaling pathways, and channel activity, impacting cell division progression.

Variations between YFP and EFP genomes

Synteny analysis of the gene order between YFP v2.0 and EFP v2.0 revealed 907 large shared syntenic blocks, encompassing 89.59% (41,428) genes, and 17 chromosomal rearrangements (Figure 3). Further comparison of the genomic sequences of the YFP v2.0 and EFP v2.0 genomes, however, yielded numerous variations between the two, including 3,887,060 single-nucleotide polymorphisms (SNPs) (Figure 4A) and 704,944 short insertions/deletions (InDels) (Figure 4B). The variations in SNPs were primarily located in the intergenic regions (65.28%) and intronic regions (32.66%), and rarely in the exon regions (0.64%). Similarly, variations in InDels were less concentrated in exon regions (0.23%) (Supplementary Table S21). Of the exon region variations, 12,203 SNPs (11,987 non-synonymous SNPs, 189 stop-gain SNPs, and 27 stop-loss SNPs) and 953 InDels (464 frameshift deletion, 467 frameshift insertion, 20 stop-gain InDels, and 2 stop-loss InDels) were identified and functionally associated with 6,158 and 582 genes, respectively. KEGG enrichment analysis revealed that these genes were significantly (P value < =0.05) enriched in "NF-kappa B signaling pathway", "complement and coagulation cascades", "antigen processing and presentation" and "Intestinal immune network for IgA production" (Supplementary Figure S10 and Table S22).

The genes coding for the mutated regions of the YFP and EFP are widely enriched in immune-related pathways. This association may be intricately linked to the distinct pathogenic microorganisms unique to freshwater and seawater environments. Marine mammals exhibit a diminished histocompatibility complex (MHC) diversity attributed to decreased encounters with microparasitic diversity in their marine habitat relative to their terrestrial origin. This phenomenon implies that mammals encounter distinct pathogenic pressures in varied ecological settings, potentially influencing the evolution of immune-related genes [26]. Evolutionary analyses of the innate immune pattern recognition receptor (TLRs) in the YFP and the marine finless porpoise indicate that the YFP has undergone specific adaptive changes [27]. The microbial diversity and pathogenicity of freshwater and seawater environments vary, leading to distinct effects of

pathogenic microorganisms on the organisms in these two types of environments [28]. Therefore, the YFP and EFP would be expected to undergo adaptive evolution to adapt to the pathogen stresses specific to their respective ecological environments, freshwater and seawater.

By comparing the assembly results of the YFP and EFP versions, it was found that the YFP v2.0 assembly included 5,480 new genes, while the EFP v2.0 assembly included 1,453 new genes compared to the previous version of the assembly (Table 1 and Supplementary Table S23). These genes were expressed in all 24 samples (Supplementary Figure S11 and Table S24-S25). GO and KEGG functional enrichment analysis indicated that these genes are primarily enriched in immunity and iron binding, among other functions. These biological processes encompass a wide range of aspects, including cellular structure and function, protein synthesis, signal transduction, immune response, and energy metabolism (Supplementary Table S26-S29).

Phylogeny analysis

Gene family analysis was performed on 506,098 protein-coding sequences derived from ten cetaceans and sixteen terrestrial mammalian species. The sequences were clustered into 22,196 gene families, which include 594 species-specific genes identified in YFP v2.0 and EFP v2.0 (Supplementary Table S30). Among the dataset, a total of 2,161 single-copy gene families were identified. Subsequently, multiple sequence alignments were conducted, which were then followed by the reconstruction of evolutionary trees. The resulting phylogenetic tree produced a topology consistent with previous studies [29], highlighting its reliability and alignment with established scientific knowledge. This analysis notably revealed the topological arrangement within branches of mammals. The emergence of the EFP branch alongside its YFP counterpart was particularly noteworthy. (Supplementary Figure S12). Our analysis had revealed that the divergence time between the YFP and EFP ranges from 0.5 to 1.1 million years ago (Figure 5A, Figure 5B and Supplementary Figure S13), which is the first estimate at the molecular level since their classification as two distinct subspecies.

Gene family and positive selection analysis

We used CAFÉv4.0 to analyze the evolution of gene families based on orthologous clusters of protein coding sequences from twenty-six mammals. Upon comparing the genomes of YFP v2.0 and EFP v2.0 with their most recent common ancestor, it was observed that 843 gene families underwent expansion while 98 gene families experienced contraction (Figure 5A and Figure 5B).

Among the 215 expanded gene families identified in the YFP v2.0 and EFP v2.0 lineage, a total of 2,674 genes were determined to be significantly associated ($P<0.05$) (Supplementary Table S31). We observed an expansion for genes significantly enriched in several KEGG pathways, including "antigen processing and presentation", "intestinal immune network for IgA production", "oxidative phosphorylation", and the "calcium signaling pathway" (Figure 6A). The significantly enriched GO terms, included "ferric iron binding", "iron ion transport", "riboflavin biosynthetic process", "tetrahydrofolate biosynthetic process" and "cytochrome-c oxidase activity", also expanded (Figure 6B).

Gene families associated with "oxidative phosphorylation", "cytochrome-c oxidase activity", "riboflavin biosynthetic process", "ferric iron binding" and "iron ion transport" exhibited expansion in finless porpoises. Iron ions play a crucial role in numerous essential physiological functions within living organisms, such as oxygen transportation, electron transport chains, and various metabolic pathways. Several sets of redox reactions are necessary to maintain effective gas exchange in water for cetaceans, and iron is often used in these reactions as an electron acceptor [30]. The expansion of redox reaction and iron ion gene families in cetaceans potentially improved the efficacy of oxygen utilization and facilitated adaptation to hypoxic conditions in aquatic habitats. An increase in the number of oxidation-reduction and iron-binding gene families was also observed in *L. vexillifer*, a species that encounters hypoxic conditions during dives [31]. The changes in pathogenic microorganisms that occur during the reintroduction of cetaceans from land to sea present a significant challenge to their survival and may have influenced the evolution and adaptation of immune genes [32]. The expansion of immune-related gene families in finless porpoises in this study enhanced the process of antigen presentation and conferred resistance against various pathogenic microorganisms amidst changes in their habitat. Genomic studies of the *S. chinensis* have revealed that cetaceans have developed various species-specific gene families related to immunity and DNA repair, which are linked to potential adaptive mechanisms [33]. We suggest these genes play a crucial role in facilitating hypoxic tolerance and enhancing immune resistance in finless porpoises, thereby reflecting potential mechanisms of adaptation to the aquatic environment. Further investigations are required to elucidate the specific functions of these gene families and their potential significance in the biology of finless porpoises.

The Codeml program in PAML with a branch-site model was employed to test for selective

pressure based on orthologous clusters of 10 cetaceans, including *Neophocaena asiaeorientalis asiaeorientalis* (Yangtze finless porpoise), *Neophocaena asiaeorientalis sunameri* (East Asian finless porpoise), *Tursiops truncatus* (Bottlenose dolphin), *Orcinus orca* (Killer whale), *Lipotes vexillifer* (Yangtze River dolphin), *Physeter catodon* (Sperm whale), *Balaenoptera acutorostrata* (Minke whale), *Balaena mysticetus* (Bowhead whale), *Delphinapterus leucas* (Beluga whale), *Sousa chinensis* (Indo-Pacific humpback dolphin) . We identified 41 positively selected genes (PSGs) in the YFP lineage, which were functionally enriched in "RNA degradation", "nucleotide excision repair", "DNA replication", "mismatch repair", and "homologous recombination" pathways (P<0.05) (Figure 6C and Supplementary Table S32). The selective pressure to evolve DNA damage repair pathways implied that the Yangtze finless porpoise might be experiencing increased threats to genome stability. The mechanism of DNA damage repair plays a crucial role in preserving genome integrity by enabling cells to identify and repair DNA damage, thereby averting the accumulation of harmful mutations [34]. In a comparative genomic analysis between the South China tiger and the Amur tiger, it was noted that genes related to DNA repair underwent positive selection in the South China tiger [21]. The observed phenomenon could be explained by the higher probability of genome instability in the temperate and subtropical habitats of the South China tiger. This may be linked to metabolites generated by intestinal microflora, which possess the ability to trigger DNA damage [35]. The stability of the genome or gene expression system in the Yangtze finless porpoise across different organs and life stages remains uncertain. However, one potential interpretation of this data is the suggestion that the Yangtze finless porpoise could be vulnerable to genomic instability triggers in the Yangtze River, such as water pollutants, which may increase the likelihood of DNA damage [36]. Pollutants found in the Yangtze River possess the capacity to accumulate within the food chain, leading to cellular DNA damage and impacting the genome stability of the Yangtze finless porpoise [37, 38]. This makes a compelling case for the improved conservation of the species and the development of more rigorous water pollution mitigation practices.

Interestingly, a total of 44 PSGs within the EFP lineage were involved in "sodium-dependent phosphate transport", "sodium symporter activity", "aldosterone-regulated sodium reabsorption", and "calcium signaling pathway" (Figure 6D and Supplementary Table S33). These metabolic pathways play an important role in regulating sodium levels in the body [39]. Among these PSGs,

six were potentially associated with the adaptation of EFP to high osmolarity environment, including the Na(+)/H(+) exchange regulatory cofactor *NHE-RF2* and sodium-dependent phosphate cotransporter *SLC34*. These genes identified are likely to have significant implications in the control of urine formation and the preservation of water-salt metabolic balance [39], suggesting that the East Asian finless porpoise may have a different urine formation process. Previous genome-selective sweep analyses in the finless porpoises revealed that the *SLC14A* in the East Asian finless porpoise underwent positive selection [14]. Comparative analyses conducted at the transcriptome level between the Yangtze finless porpoise and the East Asian finless porpoise revealed a notable upregulation of the *NHE3* in the kidneys of the East Asian finless porpoise. These results imply some adaptive enhanced osmoregulatory capability in the East Asian finless porpoise [40]. The aforementioned studies suggest that the East Asian finless porpoise has evolved a complex and efficient osmoregulatory mechanism as it acclimatized to the hypertonic marine environment, demonstrating adaptations at both molecular and transcriptional levels.

Methods

Sample collection, DNA extraction, and sequencing

We collected an adult dead female YFP sample from Lianzhou Lake, Anqing City, Anhui Province, China (N30°15′32″, E116°54′38″) in 2021 and a dead juvenile male EFP sample from the Yellow Sea near Lianyungang City, Jiangsu Province, China (N34°55′27″, E119°11′37″) in 2019 for sequencing (Figure 1A). The Office of Fishery Supervision and Management in the Yangtze River Basin, Ministry of Agriculture and Rural Affairs of the People's Republic of China has designated our research institution to perform post-mortem analysis and genetic preservation on deceased porpoises. No ethical considerations were taken into account in this study. DNA were extracted from muscle tissues following the phenol/chloroform DNA extraction method. DNA extracted from the YFP was utilized to construct PacBio HiFi, Hi-C, and Oxford Nanopore Technologies (ONT) libraries. DNA extracted from an EFP was utilized to construct an ONT library. The PacBio HiFi library was constructed using SMRTbell Prep Kit 3.0 (Pacific Biosciences, USA) and subsequently sequenced on the PacBio Sequel II system in circular consensus sequence (CCS) mode. To collect data for the Hi-C library, the muscle tissues were first fixed in 1% formaldehyde (Sigma) for cross-linking and resuspended in lysis buffer. Then, MboI (NEB) restriction endonucleases were used to

fragment the chromatin in the muscle to fragment DNA. The DNA fragments were captured by utilizing Streptavidin-coated magnetic beads (Thermo Fisher SCIENTIFIC) following biotin labeling and crosslinking using T4 DNA Ligase (ENZYMATICS). The Hi-C library was finally sequenced on a BGI MGISEQ platform. To generate and sequence ONT libraries, we isolated genomic DNA using the CTAB [41] method, selected fragments exceeding 5 kb in size with the SageHLS HMW library system (Sage Science), processed the DNA with the Ligation sequencing 1D kit (SQK-LSK109, Oxford Nanopore Technologies, Oxford, UK), and subsequently sequenced the ONT libraries on a PromethION platform (Oxford Nanopore Technologies) at the BGI (Wuhan, China).

Gap-free genome assembly and quality assessment

We utilized SMRTLink v11.0.0 (https://www.pacb.com/support/software-downloads) to filter PacBio HiFi reads, applying the following criteria: a minimum requirement of three full-length subreads for generating CCS, a draft length threshold of 500 bp before polishing, and aiming for a predicted accuracy of 0.99. To filter Hi-C reads, index reads were removed and reads were filtered using the following SOAPNUKE v2.0 [42] parameters: N rate >= 0.01, low quality <= 20, low quality rate >= 0.1. Subsequently, ONT reads were filtered based on a length < 5 kb and a quality value < 7. The Necat pipeline (v 20200119) [43] was utilized for the enhancement of ONT reads. This was achieved through the application of error correction algorithms to evaluate quality scores, k-mer frequencies, and alignment methods. This process enhanced the accuracy of reading and generated refined results suitable for further analyses. To achieve gap-free chromosome-level assemblies, we employed both the Hifiasm (v0.15.1) [44] and Necat pipeline (v20200119) [43] to separately assemble PacBio HiFi reads and ONT corrected reads into the initial contigs. The Purge-Haplotigs program [45] was utilized to eliminate redundant contigs that exhibited similar sequences but distinct haplotypes, specifically targeting those with aligned coverage below 30%. This strategic approach significantly optimizes the assembly process by removing redundant information, thus improving the accuracy of genome assembly. We utilized Hi-C data to cluster, order, and orient the contigs into pseudo-chromosomes though the implementation of the Juicer (v1.5) [46] and 3D-DNA (v180922) [47] pipelines. Ultra-long ONT reads and contigs were used to generate gapless scaffolds through the LR_Gapcloser (v1.0) [48] and TGSgapcloser (v 1.0.1) [49] pipelines.

Various metrics were used to evaluate the quality of the gap-free genome assemblies, including contiguity, accuracy, and completeness. First, we calculated the length metrics of genomic sequences to evaluate contiguity and subsequently used Merqury (v1.3) [24] with k-mer set to 21 to assess the accuracy. Second, Benchmarking Universal Single-Copy Orthologs (BUSCO) [50] evaluation was conducted to assess the completeness. Third, we also mapped PacBio HiFi, ONT and RNA-seq data into the genome assemblies using Minimap2 [51] and Hisat2 (v2.1.0) [52] to assess the completeness. In addition, we utilized the quartet pipeline [53] to search for telomere repeat sequences and centromere regions in YFP and EFP.

Genome annotation

Repetitive sequence annotation was identified using both *de novo* and homology-base approaches. For the *de novo* strategy, RepeatModeler (v1.0.4) [54] was employed to identify repetitive elements, whereas LTR-FINDER (v1.0.7) [55] was used for the specific annotation of long terminal repeats. For homolog-based prediction, RepeatMasker (v4.0.7) [56] was utilized to detect DNA transposable elements (TEs), while RepeatProteinMasker (v4.0.7) was employed to identify protein-based TEs, both based on the Repbase database. Additionally, Tandem Repeat Finder (v4.10.0) [57] was used to identify Tandem repeats. The utilization of these tools facilitated a comprehensive annotation of repetitive sequences, leading to a substantial improvement in the accuracy and detail of the analysis.

A combination of RNA-seq, homology-based and *de novo* prediction strategies was utilized to identify protein-coding genes in the genomes of YFP and EFP. RNA-seq data [15, 16] were mapped to genome assemblies with Hisat2 v2.1.0 [58] with the following parameters: --sensitive --no-discordant --no-mixed -I 1 -X 1000 --max-intronlen 1000000. The produced BAM alignments were further assembled into gene models with StringTie v1.3.5 [59] with the following parameters: -f 0.3 -j 3 -c 5 -g 100 -s 10000 and validated using PASA v2.5.2 [60]. The coding sequences were identified by TransDecoder (v5.5.0) (https://github.com/TransDecoder/TransDecoder) with default parameters. Utilizing 149,956 genes from 8 closely related cetacean species and transcriptomic sequencing data from 24 YFPs as input files, the GeMoMa v1.9 [61] software was employed to conduct homology-based prediction analysis (Supplementary Table S7, S34-S35). A set of one thousand high quality genes, which were predicted by the GeMoMa software and validated by OrthoDB for mammals, were randomly selected for training the predictors in Augustus v3.2.1 [62]. The Augustus v3.2.1 program was used to perform *de novo* prediction. We used GeMoMa software

to integrate all predicted protein-coding genes, and annotated them with NR, Swissprot [63], KEGG [64], KOG, TrEMBL, InterPro [65] and GO [66] databases.

Genome comparison and Identification of newly assembled genes

Single nucleotide polymorphisms (SNPs) and insertions/deletions (InDels) were identified using methods following those previously described [67]. Genome alignment was conducted utilizing the NUCmer program integrated within MUMmer4 (v4.0.0) [68] to compare the v2.0 assembly with the v1.0 assembly, as well as the YFP assembly with the EFP assembly. Utilizing the Maximum Unique Matches (MUM) mode involved setting parameters such as a minimum MUM length of 1000 bp, a minimum similarity threshold of 90%, and the exclusion of matches below 40 bp. Alignment blocks were identified using the delta-filter program, while SNPs and InDels were detected using the show-snps program and Syri (v1.6.3) [69], respectively. Functional annotation of SNPs and InDels is conducted using ANNOVAR [70] to assess their impacts on gene structure and function. Additionally, we utilize the CMplot R package [71] to visually depict the density distribution of these genetic variations. The objective of these analyses was to reveal structural variations between the v1.0 and v2.0 genome assemblies, as well as between the YFP and EFP genome assemblies. Variants were meticulously annotated utilizing the ANNOVAR package (v 2013-06-21) [70].

Genes were categorized as newly assembled when the gene region in the initial draft genome assembly showed a deletion of at least 50 bp and had a minimum overlap of 30% within that specific region.

Gene family and phylogenomic analysis

Gene families of 26 species (Supplementary Table S30) were identified and clustered by OrthoFinder (v2.3.11) [72]. Single-copy orthologous genes (1:1:1) were aligned using MAFFT [73] (v7.310), a widely utilized tool for multiple sequence alignment. Subsequently, a maximum-likelihood phylogenetic tree was generated using PhyML (v3.3) [74], a commonly employed software tool for phylogenetic analysis. The HKY85 model, known for its capacity to accommodate nucleotide substitution patterns, was applied in the tree construction procedure. To evaluate the reliability of the generated tree, 1000 bootstrap replications were performed, providing statistical evidence for the branching structures. Species divergence time was calculated using MCMCTREE in PAML (v4.9) [75]. Four divergence time points from TimeTree (http://timetree.org.cn) were used

to calibrate the divergence times: (a) *Ornithorhynchus anatinus* (Platypus) and *Monodelphis domestica* (Opossum) (163.7–185.9 Ma), (b) *Homo sapiens* (Human) and *Mus musculus* (Mouse) (81.3–91.0 MYA), (c) *Balaena mysticetus* and *Balaenoptera acutorostrata* (21.3–28.8 Ma) and (d) *Sousa chinensis* and *Tursiops truncatus* (2.0–3.8 Ma). The core-orthologous gene sets were identified by BLAST (v2.0.14) [76] with an E-value threshold of $1\times E^{-10}$ (at least 10 syntenic genes allowed), and syntenic blocks were defined using MCscanX v1.5.2 [77]. Circos was used to plot the synteny results.

Gene family expansion and contraction analysis

Protein sequences of YFP, EFP and 24 published mammals were used to search homologs. Based on the gene families clustered by OrthoFinder, the CAFÉ (v4.0) [78] software was used to perform expansion and contraction analyses in the clade of finless porpoises. Random birth and death models were employed to study gains and losses of gene families in a user-specified phylogeny. The global parameter λ, which describes both the gene birth (λ) and death ($\mu = -\lambda$) rate for gene families in all branches of the tree, was estimated using maximum likelihood. Then the P-value was calculated for each gene family, and P-value ≤ 0.01 was defined as a "significantly expanded or contracted gene family". KEGG and GO enrichment analyses were conducted among these significantly expanded and contracted gene families.

Gene positive selection analysis

Protein sequences of two finless porpoise and another eight cetaceans were used to identify single copy orthologs with OrthoFinder. Then Ka/Ks ratios for these single copy orthologs were calculated by following steps. Initially, the single-copy orthologs underwent global alignment using PRANK. Subsequently, alignment refinement via Gblocks was utilized to remove inadequately aligned positions and divergent regions, thereby isolating conserved blocks from the multiple alignment. Codeml from the PAML package [75] was ultimately employed to compute Ka/Ks ratios across various branches, utilizing the free-ratio model. Genes that showed values of Ka/Ks higher than 1 along the branch leading to finless porpoise were reanalyzed using the codon-based branch site tests implemented in PAML (PAML, RRID:SCR_014932). The branch site model allowed ω to vary both among sites in the protein and across branches, and it was used to detect episodic positive selection.

Gene expression analysis

The raw RNA-seq reads underwent quality control using SOAPnuke (v2.0). Reads were filtered out if they had an N rate ≥ 0.01, low quality ≤ 20, low quality rate ≥ 0.1, or contained index sequences. Subsequently, the clean reads were aligned to the EFS v2.0 genome utilizing the Hisat2 high sensitivity model, excluding discordant pairs and mixed alignments. The alignment process involved setting a minimum insert size of 1 bp and a maximum insert size of 1000 bp. We utilized featureCounts [79] and transcripts per million (TPM) method to generate an estimated mapped read count matrix and calculate the gene expression level, respectively.

Conclusion

The availability of reliable chromosome-level genome assemblies provides a remarkable improvement in identifying genes, characterizing genomic regions and performing comparative genomic analyses. In the present study, we assembled telomere-to-telomere and gap-free Yangtze finless porpoise and the East Asian finless porpoise genomes by combining PacBio long reads, Hi-C and short-read sequencing technologies. The new assemblies have higher contiguity and completeness, as well as more complete single-copy BUSCO genes with fewer fragmented or missing genes than the first drafts. The reconstructed phylogeny determined that YFP and EFP diverged approximately 0.5-1.1 million years ago (Ma). Gene family expansion analysis revealed significantly enriched pathways and GO terms associated with the regulation of immune resistance and hypoxic tolerance. Selection pressure analysis identified genes associated with DNA damage repair in the YFP and high salt tolerance in the EFP, respectively. Our results provide evidence of the gradual adaptation of EFP to a marine environment and the potential sensitivity of YFP to genome damage. Identification of the centromere, telomere, and associated genes can serve as valuable resources for a comprehensive understanding of chromosome stability, recombination, repair mechanisms, and evolutionary processes. Overall, these are the most continuous cetacean genome assemblies to date, with chromosome-scale contigs and no gaps. This study will lay a foundation for population genomics studies at the whole genome level, and deepen the scientific understanding of issues related to population conservation and adaptation mechanisms.

DATA AVAILABILITY

Raw sequencing data and genome assemblies in this study have been deposited in the NCBI database (BioProject ID PRJNA915046 and PRJNA859258). Furthermore, results of repeat annotation, gene structure annotation and gene functional annotation had been deposited in the *figshare* https://figshare.com/s/1fc632fd4f3cab36b776.

COMPETING INTERESTS

The authors declare that they have no competing interests.

AUTHORS' CONTRIBUTIONS

K.L., J.J. and P.X. designed and conceived the study. D.Y., C.Y. and J.Z. collected and prepared the samples. C.C. and C.Z. performed the data analysis. D.Y., C.C. and J.J. wrote the manuscript with significant contributions from Y.L., Z.C., H.Z., C.W. and L.L. K.L., Z.H. and D.L. provided the financial support. All authors read and approved the final version of the manuscript.

ACKNOWLEDGMENTS

This work was funded by the National Key R&D Program of China (2021YFD1200304), the Central Public-interest Scientific Institution Basal Research Fund, Freshwater Fisheries Research Center, CAFS (2021JBFM15) and Project of Implementation of Yangtze Finless Porpoise Protection in the Middle and Lower Reaches of Yangtze River (2021).

REFERENCES

1. Gao, A and Zhou, K. Growth and reproduction of three populations of finless porpoise, *Neophocaena phocaenoides*, in Chinese waters. *Aquat Mamm* 1993;**19** (1):3-12.
2. Jefferson, T. Preliminary analysis of geographic variation in cranial morphometrics of the finless porpoise (*Neophocaena phocaenoides*). *Raffles Bull Zool* 2002;**10**:3-14.
3. Wang, P. The morphological characters and the problem of subspecies identifications of the finless porpoise. *Fish Sci* 1992;**11**:4-8.
4. Gao, A and Zhou, K. Geographical variation of external measurements and three subspecies of *Neophocaena phocaenoides* in Chinese waters. *Acta Theriol Sin* 1995;**15**(2):81-92.
5. Wang, J, Frasier, T, Yang, S, *et al*. Detecting recent speciation events: the case of the finless porpoise (genus *Neophocaena*). *Heredity* 2008;**101**(2):145-55.
6. Jefferson, T and Wang, J. Revision of the taxonomy of finless porpoises (genus *Neophocaena*): The existence of two species. *J Mar Anim Ecol* 2011;**4**(1):3-16.
7. Yang, G, Ren, W, Zhou, K, *et al*. Population genetic structure of finless porpoises, *Neophocaena phocaenoides*, in Chinese waters, inferred from mitochondrial control region sequences. *Marine mammal science* 2002;**18**(2):336-47.
8. Xu, S, Sun, P, Zhou, K, *et al*. Sequence variability at three MHC loci of finless porpoises (*Neophocaena phocaenoides*). *Immunogenetics* 2007;**59**(7):581-92.
9. Chen, M, Zheng, J, Wu, M, *et al*. Genetic diversity and population structure of the critically endangered Yangtze finless porpoise (*Neophocaena asiaeorientalis asiaeorientalis*) as revealed by mitochondrial and microsatellite DNA. *Int J Mol Sci* 2014;**15**(7):11307-23.
10. Chen, M, Fontaine, M, Chehida Y, *et al*. Genetic footprint of population fragmentation and contemporary collapse in a freshwater cetacean. *Sci Rep* 2017;**7**(1):14449.
11. Lin, W, Frère, C, Karczmarski, L, *et al*. Phylogeography of the finless porpoise (genus *Neophocaena*): testing the stepwise divergence hypothesis in the northwestern Pacific. *Sci Rep* 2014;**4**:6572.
12. Li, S, Xu, S, Wan, H, *et al*. Genome-wide SNP and population divergence of finless porpoises. *Genome Biol Evol* 2013;**5**(4):758-68.
13. Zheng, J, Xia, J, He, S, *et al*. Population genetic structure of the Yangtze finless porpoise (*Neophocaena phocaenoides asiaeorientalis*): implications for management and conservation. *Biochem Genet* 2005;**43**(5-6):307-20.
14. Zhou, X, Guang, X, Sun, D, *et al*. Population genomics of finless porpoises reveal an incipient cetacean species adapted to freshwater. *Nat Commun* 2018;**9**(1):1276.
15. Yin, D, Lin, D, Guo, H, *et al*. Integrated analysis of blood mRNAs and microRNAs reveals immune changes with age in the Yangtze finless porpoise (*Neophocaena asiaeorientalis*). Comp *Biochem Physiol B Biochem Mol Biol* 2021;**256**:110635.
16. Liu, W, Yin, D, Lin, D, *et al*. Blood Transcriptome Analysis Reveals Gene Expression Differences between Yangtze Finless Porpoises from Two Habitats: Natural and Ex Situ Protected Waters. *fishes* 2022;**7**:96.
17. Nurk, S, Koren, S, Rhie, A, *et al*. The complete sequence of a human genome. *Science* 2022;**376**(6588):44-53.
18. Rhie A, Nurk S, Cechova M, *et al*. The complete sequence of a human Y chromosome. *Nature* 2023;**621**(7978):344-54.
19. Huang, Z, Xu, Z, Bai, H, *et al*. Evolutionary analysis of a complete chicken genome. *Proc Natl*

Acad Sci U S A 2023;**120**(8):e2216641120.

20. Xue, L, Gao, Y, Wu, M *et al*. Telomere-to-telomere assembly of a fish Y chromosome reveals the origin of a young sex chromosome pair. *Genome Biol* 2021;**22**(1):203.
21. Zhang, L, Lan, T, Lin, C, *et al*. Chromosome-scale genomes reveal genomic consequences of inbreeding in the South China tiger: A comparative study with the Amur tiger. *Mol Ecol Resour* 2023;**23**(2):330-47.
22. Shukla, H, Suryamohan, K, Khan, A, *et al*. Near-chromosomal de novo assembly of Bengal tiger genome reveals genetic hallmarks of apex predation. *Gigascience* 2022;**12**:giac112.
23. Yin, D, Chen, C, Lin, D, *et al*. Gapless genome assembly of East Asian finless porpoise. *Sci Data* 2022;**9**(1):765.
24. Rhie, A, Walenz, B, Koren, S, *et al*. Merqury: reference-free quality, completeness, and phasing assessment for genome assemblies. *Genome Biol* 2020;**21**(1):245.
25. Zhang, A, Kong, T, Sun, B, *et al*. A telomere-to-telomere genome assembly of Zhonghuang 13, a widely-grown soybean variety from the original center of Glycine max. *The Crop Journal* 2023. doi:https://doi.org/10.1016/j.cj.2023.10.003.
26. Slade, R and McCallum, H. Overdominant vs. frequency-dependent selection at MHC loci. *Genetics* 1992;**132**(3):861–64.
27. Tian, R, Chen, M, Chai, S, *et al*. Divergent Selection of Pattern Recognition Receptors in Mammals with Different Ecological Characteristics. *J Mol Evol* 2018;**86**(2):138-49.
28. Lokesh, J and Kiron, V. Transition from freshwater to seawater reshapes the skin-associated microbiota of Atlantic salmon. *Sci Rep* 2016;**25**(6):19707.
29. Yuan, Y, Zhang, Y, Zhang, P, *et al*. Comparative genomics provides insights into the aquatic adaptations of mammals. *Proc Natl Acad Sci U S A* 2021;**118**(37):e2106080118.
30. Guo, B, Sun, Y, Wang, Y, *et al*. Evolutionary genetics of pulmonary anatomical adaptations in deep-diving cetaceans. *BMC Genomics* 2024;**25**(1):339.
31. Zhou, X, Sun, F, Xu, S, *et al*. Baiji genomes reveal low genetic variability and new insights into secondary aquatic adaptations. *Nat Commun* 2013;**4**:2708.
32. Li, L, Rong, X, Li, G, *et al*. Genomic organization and adaptive evolution of IGHC genes in marine mammals. *Mol Immunol* 2018;**99**:75-81.
33. Ming, Y, Jian, J, Yu, F, *et al*. Molecular footprints of inshore aquatic adaptation in Indo-Pacific humpback dolphin (*Sousa chinensis*). *Genomics* 2019;**111**(5):1034-42.
34. Chatterjee, N and Walker, G. Mechanisms of DNA damage, repair, and mutagenesis. *Environ Mol Mutagen* 2017;**58**(5):235-63.
35. Puschhof, J and Sears, C. Microbial metabolites damage DNA. *Science* 2022;**378**(6618):358-59.
36. Lv, W, Gu, H, He, D, *et al*. Polystyrene nanospheres-induced hepatotoxicity in swamp eel (*Monopterus albus*): From biochemical, pathological and transcriptomic perspectives. *Sci Total Environ* 2023;**893**:164844.
37. Zhang, K, Qian, Z, Ruan, Y, *et al*. First evaluation of legacy persistent organic pollutant contamination status of stranded Yangtze finless porpoises along the Yangtze River Basin, China. *Sci Total Environ* 2020;**710**:136446.
38. Xiong, X, Qian, Z, Mei, Z, *et al*. Trace elements accumulation in the Yangtze finless porpoise (*Neophocaena asiaeorientalis asiaeorientalis*)-A threat to the endangered freshwater cetacean. *Sci Total Environ* 2019;**686**:797-804.

39. Shoemaker, V and Nagy, K. Osmoregulation in amphibians and reptiles. *Annu Rev Physiol*. 1977;**39**: 449-71.
40. Ruan, R, Guo, A, Hao, Y, *et al*. De novo assembly and characterization of narrow-ridged finless porpoise renal transcriptome and identification of candidate genes involved in osmoregulation. *Int J Mol Sci* 2015;**16**(1):2220-38.
41. Yan, M, Wei, G, Pan, X *et al*. A method suitable for extracting genomic DNA from animal and plant-modified CTAB method. *Agric Sci Technol*, 2008, **9**(2):39-41.
42. Chen Y, Chen Y, Shi C, *et al*. SOAPnuke: a MapReduce acceleration-supported software for integrated quality control and preprocessing of high-throughput sequencing data. *Gigascience* 2018;**7**(1):1-6.
43. Chen Y, Nie F, Xie S, *et al*. Efficient assembly of nanopore reads via highly accurate and intact error correction. *Nat Commun* 2021;**12**(1):60.
44. Cheng, H, Concepcion, G, Feng, X, *et al*. Haplotype-resolved de novo assembly using phased assembly graphs with hifiasm. *Nat Methods* 2021;**18**(2):170-5.
45. Roach, M, Schmidt, S and Borneman, A. Purge Haplotigs: allelic contig reassignment for third-gen diploid genome assemblies. *BMC bioinformatics* 2018;**19**(1):460.
46. Durand, N, Shamim, M, Machol, I, *et al*. Juicer Provides a One-Click System for Analyzing Loop-Resolution Hi-C Experiments. *Cell systems* 2016;**3**(1):95-8.
47. Dudchenko, O, Batra, S, Omer, A, *et al*. De novo assembly of the Aedes aegypti genome using Hi-C yields chromosome-length scaffolds. *Science* 2017;**356**(6333):92-5.
48. Xu, G, Xu, T, Zhu, R, *et al*. LR_Gapcloser: a tiling path-based gap closer that uses long reads to complete genome assembly. *Gigascience* 2019;**8**(1):giy157.
49. Xu M, Guo L, Gu S, *et al*. TGS-GapCloser: A fast and accurate gap closer for large genomes with low coverage of error-prone long reads. *Gigascience* 2020;**9**(9):giaa094.
50. Waterhouse, R, Seppey, M, Simão, F, *et al*. BUSCO Applications from Quality Assessments to Gene Prediction and Phylogenomics. *Mol Biol Evol* 2018;**35**(3):543-8.
51. Li, H. Minimap2: pairwise alignment for nucleotide sequences. *Bioinformatics* 2018;**34** (18):3094-100.
52. Kim, D, Paggi, J, Park, C, *et al*. Graph-based genome alignment and genotyping with HISAT2 and HISAT-genotype. *Nat Biotechnol* 2019;**37**(8):907-15.
53. Lin, Y, Ye, C, Li, X, *et al*. quarTeT: a telomere-to-telomere toolkit for gap-free genome assembly and centromeric repeat identification. *Hortic Res* 2023;**10**(8):uhad127.
54. Chen N. Using RepeatMasker to identify repetitive elements in genomic sequences. *Curr Protoc Bioinformatics* 2004;**Chapter 4**:Unit 4.10.
55. Xu, Z and Wang, H. LTR_FINDER: an efficient tool for the prediction of full-length LTR retrotransposons. *Nucleic Acids Res* 2007;**35**:W265-8.
56. Price, A, Jones, N and Pevzner, P. De novo identification of repeat families in large genomes. *Bioinformatics* 2005;**21**(Suppl 1):i351-8.
57. Benson, G. Tandem repeats finder: a program to analyze DNA sequences. *Nucleic Acids Res* 1999;**27**(2):573-80.
58. Kim, D, Langmead, B and Salzberg, S. HISAT: a fast spliced aligner with low memory requirements. *Nat Methods* 2015;**12**(4):357-60.
59. Kovaka, S, Zimin, A, Pertea, G, *et al*. Transcriptome assembly from long-read RNA-seq alignments with StringTie2. *Genome Biol* 2019;**20**(1):278.

60. Haas, B, Salzberg, S, Zhu W, *et al*. Automated eukaryotic gene structure annotation using EVidenceModeler and the Program to Assemble Spliced Alignments. *Genome Biol* 2008;**9**(1):R7.
61. Keilwagen, J, Hartung, F and Grau, J. GeMoMa: Homology-Based Gene Prediction Utilizing Intron Position Conservation and RNA-seq Data. *Methods Mol Biol* 2019;**1962**:161-77.
62. Stanke, M and Waack, S. Gene prediction with a hidden Markov model and a new intron submodel. *Bioinformatics* 2003;**19**(Suppl 2):ii215-25.
63. Bairoch, A and Apweiler, R. The SWISS-PROT protein sequence data bank and its supplement TrEMBL. *Nucleic Acids Res* 1997;**25**(1):31-6.
64. Kanehisa, M, Sato, Y, Kawashima, M, *et al*. KEGG as a reference resource for gene and protein annotation. *Nucleic Acids Res* 2016;**44**(D1):D457-62.
65. Jones, P, Binns, D, Chang, H, *et al*. InterProScan 5: genome-scale protein function classification. *Bioinformatics* 2014;**30**(9):1236-40.
66. Ashburner, M, Ball, CA, Blake, J, *et al*. Gene ontology: tool for the unification of biology. The Gene Ontology Consortium. *Nat Genet* 2000;**25**(1):25-9.
67. Li, T, Xu, X, Zhao, J, *et al*. Genome assembly of KA105, a new resource for maize molecular breeding and genomic research. *The Crop Journal* 2023;**11**(6):1793-1804.
68. Marçais, G, Delcher, A, Phillippy, A, *et al*. MUMmer4: A fast and versatile genome alignment system. *PLoS Comput Biol* 2018;**14**(1):e1005944.
69. Goel, M, Sun, H, Jiao, W, *et al*. SyRI: finding genomic rearrangements and local sequence differences from whole-genome assemblies. *Genome Biol* 2019;**20**(1):277.
70. Wang, K, Li, M and Hakonarson, H. ANNOVAR: functional annotation of genetic variants from high-throughput sequencing data. *Nucleic Acids Res* 2010;**38**(16):e164.
71. Yin, L, Zhang, H, Tang, Z, *et al*. rMVP: A Memory-efficient, Visualization-enhanced, and Parallel-accelerated tool for Genome-Wide Association Study. *Genomics, Proteomics & Bioinformatics* 2021;**19**(4):619-28.
72. Emms, D and Kelly, S. OrthoFinder: phylogenetic orthology inference for comparative genomics. *Genome Biol* 2019;**20**(1):238.
73. Nakamura, T, Yamada, K, Tomii, K, *et al*. Parallelization of MAFFT for large-scale multiple sequence alignments. *Bioinformatics* 2018;**34**(14):2490-2.
74. Guindon, S, Delsuc, F, Dufayard, J, *et al*. Estimating maximum likelihood phylogenies with PhyML. *Methods Mol Biol* 2009;**537**:113-37.
75. Yang, Z. PAML 4: phylogenetic analysis by maximum likelihood. *Mol Biol Evol* 2007;**24**(8):1586-91.
76. Altschul, S, Gish, W, Miller, W, *et al*. Basic local alignment search tool. *J Mol Biol* 1990;**215**(3):403-10.
77. Wang, Y, Tang, H, Debarry, J, *et al*. MCScanX: a toolkit for detection and evolutionary analysis of gene synteny and collinearity. *Nucleic Acids Res* 2012;**40**(7):e49.
78. Bie, T, Cristianini, N, Demuth, J, *et al*. CAFE: a computational tool for the study of gene family evolution. *Bioinformatics* 2006;**22**(10):1269-71.
79. Liao, Y, Smyth, G and Shi, W. featureCounts: an efficient general purpose program for assigning sequence reads to genomic features. *Bioinformatics* 2014;**30**(7):923-30.

Tables

Table 1. Genome assembly statistics of YFP and EFP.

	**YFP v2.0**	**YFP v1.0**	**EFP v2.0**	**EFP v1.0**
Total size of assembled genome (Gb)	2.48	2.27	2.50	2.50
Contig N50 (Mb)	125.12	0.09	128.00	84.69
Contig N90 (Mb)	83.62	0.02	80.21	29.54
Number of contigs	23	66,345	24	52
Scaffold N50 (Mb)	125.12	6.34	128.00	122.40
Scaffold N90 (Mb)	83.62	1.13	80.21	80.21
Scaffolds number	22	13,698	23	23
Number of base chromosomes	22	22	23	23
Number of gap-free chromosomes	22	0	23	7
Number of gaps	0	52,647	0	28
Number of telomeres (pairs/single)	20/2	0/0	21/2	21/2
Number of estimated centromeres	22	0	23	23
TE size	42.54%	NA	42.80%	42.23%
GC content	41.70%	41.00%	41.70%	41.70%
BUSCO* (Genome)	95.2%	94.0%	95.3%	95.4%
Gene Number	23,139	18,479	23,101	22,814
New-found gene number	5,480	NA	1,453	NA
Functional proteins	96.21%	NA	96.20%	97.31%
BUSCO* (Protein)	97.5%	94.6%	97.6%	97.9%
Data source	This study (PRJNA915046)	GCF_003031525.2	This study (PRJNA859258)	GCA_026225855.1

Note: The term "v2.0" denotes the updated genome assembly and annotation produced in the present study, while "v1.0" refers to the initial draft of the genome assembly and annotation that was previously published. “BUSCO*” indicated the percentage of complete BUSCO evaluation. “pairs/single”: “pairs” indicated that the telomeres were found at both ends of the chromosomes; “single” indicated that the telomeres were found only at the one end of chromosomes. “New-found genes” indicated that the genes were predicted in the extra sequence segments from the current assembly and were annotated in the current assembly.

Figure legends

Figure 1. Sample site and Genome assessment of YFP v2.0 and EFP v2.0.

A: Location distribution and sampling site of the YFP and EFP.

B: Proportions of genes that could be functionally annotated and transcriptionally detected in YFP v2.0 and EFP v2.0.

Figure 2. T2T-resolved assembly of YPF v2.0 and EFP v2.0 and functional enrichment of genes in the centromere region.

Structure of T2T and gap-free chromosomes in A) YFP v2.0, and B) EFP v2.0. All 21+X/Y chromosomes are drawn to scale and the ruler indicates chromosome length. Triangles indicate the presence of telomere sequence repeats. Circles represent the locations of centromeric regions. The gap positions in the v1.0 genome assemblies are marked with squares to the right of the chromosome in the v2.0 genome assemblies.

Figure 3. Synteny analysis of YFP v2.0 and EFP v2.0 genomes: A) chromosomes scale, Unit length is Mb; B) syntenic blocks between YFP v2.0 and EFP v2.0.

Figure 4. Structure variant between YFP v2.0 and EFP v2.0 genome assembly with YFP v2.0 genome assembly for reference.

The density plot of A) SNPs and B) InDels between YFP v2.0 and EFP v2.0 genome assembly. The numerical values adjacent to the color legend indicate the count of SNPs/InDels per megabase (MB) window, where the gray legend representing zero. Each color corresponds to a specific range of values. For instance, in Figure 4A, the initial blue legend represents the range from 1 to 4440.

Figure 5. Genome evolution of YFP v2.0 and EFP v2.0.

A: Divergence time between YFP v2.0 and EFP v2.0, and number of expanded and contracted gene families. Green and red numbers indicate gene family expansions and contractions, respectively. MRCA: Most Recent Common Ancestor. Ma: Million years ago.

B: A comparison of gene families associated with orthologs and paralogs in YFP v2.0 and EFP v2.0, and other 24 mammal species.

Figure 6. Functional Enrichment of genes.

Significant A) KEGG and B) GO enrichment of expanded gene families in YFP and EFP lineage.

KEGG enrichment analysis of positively selected genes in C) YFP and D) EFP, respectively.

Revised Manuscript(marked) Click here to access/download;Manuscript;Revised Manuscript(marked).docx

Telomere-to-telomere gap-free genome assembly of the endangered Yangtze finless porpoise and East Asian finless porpoise

Denghua Yin[1†], Chunhai Chen[2†], Danqing Lin[1†], Zhong Hua[1], Congping Ying[3], Jialu Zhang[1], Chenxi Zhao[2], Yan Liu[1], Zhichen Cao[4], Han Zhang[4], Chenhe Wang[2], Liping Liang[2], Pao Xu[1,3], Jianbo Jian[2,*] and Kai Liu[1,3,4,*]

[1]Key Laboratory of Freshwater Fisheries and Germplasm Resources Utilization, Ministry of Agriculture and Rural Affairs, Freshwater Fisheries Research Center, Chinese Academy of Fishery Sciences, Wuxi 214081, China.

[2]BGI Genomics, BGI-Shenzhen, Shenzhen 518083, China.

[3]Wuxi Fisheries College, Nanjing Agricultural University, Wuxi 214081, China.

[4]National Demonstration Center for Experimental Fisheries Science Education, Shanghai Ocean University, Shanghai 201306, China.

[†]Authors contributed equally to this work.

[*]Correspondence address: E-mail: jianjianbo@bgi.com, liuk@ffrc.cn.

Abstract

The Yangtze finless porpoise (*Neophocaena asiaeorientalis asiaeorientalis*, YFP) and the East Asian finless porpoise (*Neophocaena asiaeorientalis sunameri*, EFP) are two subspecies of the narrow-ridged finless porpoise that live in fresh and salt water, respectively. They serve as ideal models for the study of freshwater adaptation and evolution. The main objective of this study was to provide contiguous chromosome-level genome assemblies for YFP and EFP. Here, we generated and upgraded the genomes of YFP and EFP at the telomere-to-telomere level ~~by combining~~through the integration of PacBio HiFi long reads, ultra-long ONT reads and Hi-C sequencing data~~,~~. ~~with a~~The total sizes of the genomes are 2.48 Gb and 2.50 Gb, respectively. The scaffold N50 of two genomes was 125.12 Mb (YFP) and 128 Mb (EFP) with one contig for one chromosome. The telomere repeat and centromere position were clearly identified in both YFP and EFP genomes. Telomeric repeat units were detected at 85% and 90% of the chromosome at both ends in YFP and EFP genomes, respectively. In total, 5,480 new-found genes were detected in the YFP genome, including 56 genes located in the newly identified centromere regions. Additionally, synteny blocks, comparative genomes, phylogenetic relationships, gene family expansion and inference of selection ~~pressure~~ were studied in connection with the genomes of other related mammals. Significant improvements were observed in genome contiguity, the number of scaffolds and gaps, and annotation compared to the first drafts of the YFP (GCA_003031525.2~~GCF_000442215~~) and EFP (GCA_026225855.1) genome assemblies. The improvement of ~~Telomere~~telomere-to-telomere gap-free reference genome~~s~~ resources ~~will~~ supports conservation genetics and population management for finless porpoises.

Keywords: telomere-to-telomere; genome assembly; Yangtze finless porpoise; gap-free; HiFi sequencing; Hi-C sequencing

Introduction

Finless porpoises (*Neophocaena* spp.) are ~~uniquely~~ small toothed whales capable of inhabiting freshwater (Yangtze River) and saltwater (coastal waters of southern and eastern Asia) environments [1, 2]. They are characterized by a blunt, rounded head, an equal width upper and lower jaw, and lack of an ~~clearly~~ obvious dorsal fin [3, 4]. Based on morphological characteristics, geographic distribution and molecular genetic evidence, it is generally believed that the finless porpoise can be divided into two species, namely the Indo-Pacific finless porpoise (*N. phocaenoides*) and the narrow-ridged finless porpoise (*N. asiaeorientalis*) [5, 6]. In China, there exist two subspecies of the narrow-ridged finless porpoise: one is the freshwater ~~population (~~Yangtze finless porpoise~~,~~ (*N. a. asiaeorientalis*, YFP), which exclusively inhabits the middle and lower reaches of the Yangtze River and adjacent Dongting and Poyang lakes~~;~~. ~~while t~~The other subspecies is the marine ~~population (~~East Asian finless porpoise~~,~~ (*N. a. sunameri*, EFP), which ~~occurs~~ can be found in the coastal waters of the Yellow Sea and Bohai Sea, ~~as well as~~and in the northern waters of the East China Sea [5, 6] (Figure 1A).

The investigation of the evolutionary origins and conservation genetics of finless porpoises is ~~an urgent priority~~a pressing concern for scientists. Yang *et al*. identified significant genetic structure between either the Yangtze River population or the Yellow Sea population and the South China Sea population~~between the Indo-Pacific finless porpoise and the other two populations~~ by analyzing the sequences of mtDNA control region of finless porpoises in Chinese waters [7]. This result was supported by subsequent mtDNA sequences, nuclear DNA microsatellites, single nucleotide polymorphisms (SNPs) and MHC loci [8-12]. The genetic diversity of East Asian finless porpoises surpasses that of the other two populations, indicating it as the likely center of origin for this species [7]. Zheng *et al*. analyzed the sequences of the mtDNA control region of seven local populations of Yangtze finless porpoises in the middle and lower reaches of the Yangtze River, and found that the overall level of genetic diversity was low. Notably, the downstream population showed richer genetic variation than the midstream population. Such a genetic pattern reflects, to some extent, the marine origin and evolutionary history of the Yangtze population [13]. Based on genomic analysis of finless porpoise populations, significant genetic structure was identified among the three populations, indicating local adaptive evolution and emphasizing the evolutionary distinctiveness

and conservation significance of the Yangtze finless porpoise [14].

Availability of a high-quality genome assembly is not only critical for the genomic studies of finless porpoises, but also would be a valuable resource for comparative genomics and evolutionary studies of cetaceans. The first draft of the YFP genome assembly (GCF_003031525.2) was published in 2018 with a size of 2.3 Gb~~, which was~~ generated by short-read sequencing on the Illumina HiSeq 2000 platform [14]. ~~However, this draft was highly fragmented and consisted of 104 scaffolds with an N50 of 6.3 Mb.~~ The assembly comprised 13,698 scaffolds, with a scaffold N50 of 6.3 Mb, excluding the minimum sequence length (100 bp) consideration. Although progress had been made in genome-wide studies of YFP through the availability of this draft, such as immune changes with age and gene expression profiles in different habitats [15, 16], the lack of chromosomal information limits the potential application of the data.~~had led to some limitations in genomic studies of YFP.~~ Recent advances in ultra-long ONT and PacBio HiFi sequencing technologies, as well as assembly algorithms, have facilitated the development of ~~Telomere~~telomere-to-telomere (T2T) genome assemblies. The completion of the T2T human genome sequence and the full Y chromosome sequence represents a significant milestone in the field of human genomics research~~, offering great potential for comprehensive genomic analysis in evolutionary studies~~ [17, 18]. The T2T genome has emerged as a hotspot genomic research~~ fields now~~, demonstrating extensive applications to other animal species ~~such as~~like chicken~~,~~ and fish [19, 20]. T2T genome assemblies can serve as a benchmark with enhanced accuracy and comprehensive genomic references for future studies, facilitating the confident identification and annotation of genes, regulatory elements, and other functional components.

A ~~H~~high-quality genome can support finer genetic analyses, such as the length, number, and distribution of key indicators of inbreeding, such as ROH and IBD [21, 22], and these analyses are more urgent than ever for the conservation of endangered species. In this study, we utilize~~d~~ PacBio HiFi, Nanopore and Hi-C data to generate two improved telomere-to-telomere gap-free genomes of Yangtze and East Asian finless porpoises. We compare~~d~~ the quality of newly drafted assemblies with previously available versions and explore~~d~~ the synteny blocks, comparative genomes, phylogenetic relationships, gene family expansion and inference of selection ~~pressure~~ in relation to the several mammals. Finless porpoises serve as a representative example for comprehending speciation, evolution, and population genetics. The high-quality chromosomal-level references help

elucidate ecological and aquatic adaptation mechanisms in cetaceans~~facilitate the elucidation of adaptation mechanisms in aquatic mammals~~.

Results and Discussion

Genome sequencing and gap-free assembly

We integrated PacBio HiFi long reads, ultra-long ONT reads and Hi-C sequencing data to generate chromosome-level genome assemblies for YFP ~~-~~and EFP. Henceforth, we refer to this new T2T genome as 2.0 and the original genome as 1.0. We generated approximately 123 Gb (49 ×) PacBio HiFi reads, 279 Gb (111 ×) Hi-C reads and 225 Gb (90 ×) ONT reads for YFP v2.0 (Supplementary Table S1). ~~Based on~~In this study, we supplemented the existing dataset comprising ~~the previously sequenced~~ 62 × PacBio HiFi and 85 × Hi-C reads of the EFP ~~[15],~~ [15] ~~we generated~~with an additional 215 Gb (86 ×) of ONT reads ~~in this study~~ (Supplementary Table S1). The YFP v2.0 genome assembly ~~of the YFP~~ comprised 23 scaffolds, with both contig N50 and scaffold N50 measuring 125.12 Mb (Table 1). These scaffolds were assembled into 21 autosomal chromosomes, one X chromosome, and one mitochondrial chromosome, resulting in a final assembly size of 2.48 Gb (Supplementary Table S2). Similarly, the EFP v2.0 genome assembly ~~for EFP~~ consisted of 24 contigs or scaffolds, with both contig N50 and scaffold N50 measuring 128.00 Mb (Table 1). These scaffolds were also assembled into 21 autosomal chromosomes, one X+Y chromosome, and one mitochondrial chromosome, with a final assembly size of 2.50 Gb (Supplementary Table S2).

We have significantly enhanced the contiguity, accuracy and completeness of ~~Compared to~~ the YFP v1.0 (GCA_003031525.2~~GCF_000442215~~) and EFP v1.0 (GCA_026225855.1) assemblies~~, we have significantly enhanced the contiguity, accuracy and completeness of these two genome assemblies~~. The contig N50 values of the two genomes were consistent with their respective chromosome lengths, and a single contig represented a complete chromosome, which is notably superior to the recently published finless porpoise genomes (e.g., 125.12 Mb vs. 0.09 Mb for YFP and 128.00 Mb vs 84.69 Mb for EFP) (Table 1). The YFP v1.0 and EFP v1.0 genome assemblies had 52,647 and 28 gaps, respectively, whereas ~~in~~ the new assembly process produced ~~we filled all the gaps and obtained~~ a gap-free genome, ~~greatly~~ obviously improving ~~the~~ contiguity ~~of the assembled sequences~~ (Figure 2A and Figure 2B). The quality values obtained from Merqury's k-

mer analysis [23] for YFP v2.0 and EFP v2.0 were calculated as 60.18 and 64.38, respectively. ~~The Merqury estimated quality values of 60.18 and 64.38 based on k-mer analysis of YFP and EFP, respectively, which indicated that our assemblies were of high quality~~ (Supplementary Table S3). These values indicate a foundational accuracy level of 99.999%, confirming the high quality of our assembly for each component. Moreover, the mapping rates of RNA reads to the two genome assemblies were 95.47% and 95.42%, respectively, whereas they were 70.01% and 92.34%~~, correspondingly,~~ for previously published assemblies (Supplementary Table S4). Moreover, the mapping results of ONT long reads with a mapping quality >20 indicate a read coverage of 99.99% for YFP v2.0 and EFP v2.0 whole genome (Supplementary Figure S1). Using Benchmarking Universal Single-Copy Orthologs (BUSCO) evaluation, it is suggested our results represent~~we achieved~~ 95.20% completeness of YFP and 95.30% completeness of EFP (Table 1 and Supplementary Figure ~~1E~~S2). Additionally, we have identified/predicted both the ~~identified~~ telomeric repeat ~~units~~ and centromere candidate region in YFP v2.0 and EFP v2.0 (Figure 2A, Figure 2B and Supplementary Table S5-S6). Telomeric repeat units of YFP v2.0 genome were detected at both ends of 18 chromosomes and at one end of 3 chromosomes. Similarly, telomeric repeat units of EFP v2.0 genome were detected at both ends of 20 chromosomes and at one end of 2 chromosomes. Telomeric repeat units were detected at 85% and 90% of the chromosome at both ends in YFP v2.0 and EFP v2.0 genomes, respectively. Our estimations of the centromere region are novel and were not present in~~Notably, the centromere regions of each chromosome were predicted in the newly assembled YFP and EFP genomes, whereas the associated centromere sequences were not predicted in the first draft of~~ YFP v1.0. ~~The new genome assemblies of YFP and EFP were well-assembled without any gaps, achieving nearly telomere-to-telomere (T2T) completeness.~~ Finally, we also utilized Hi-C data for chromosome sequencing and orientation~~.~~, resulting in a comprehensive Hi-C matrix that effectively illustrates genome-wide interactions (Supplementary Figure S3~~Figure 1C and Figure 1D~~). These findings suggest that the new genome assemblies of YFP and EFP represent a significant improvement over the previously published genome assembly, achieving near telomere-to-telomere (T2T) completeness.

Gene prediction and annotation

Two strategies including *de novo* and homolog-base methods were applied to annotate repeat elements. The genomes of YFP v2.0 and EFP v2.0 contained 1,058.09 Mb (42.54%) and 1,069.10

Mb (42.80%) of repetitive sequences, respectively (Supplementary Figure S1S4 S2 _and Table S7). Long interspersed nuclear elements (LINEs) were the most abundant type of annotated transposable elements, constitutingaccounting for 38.88% and 39.10% of the genomes of YFP v2.0 and EFP v2.0, respectively (Supplementary Table S8). For gene content assessment, 8 homologous proteins and 24 RNA-seq data were used (Supplementary Table S9-S10). In total, the number of predicted protein-coding genes was 23,139 in the YFP v2.0 genome and 23,101 in the EFP v2.0 genomewe predicted 23,139 and 23,101 protein-coding genes in the YFP and EFP genomes, respectively (Table 1),). where tThe comprehensive roughly comparable number of predicted protein-coding genes for both T2T genomes is further evidence supporting the gene models he average length of coding sequence (CDS) was 1,507 bp and 1,510 bp, respectively. The average length of exon was both 175 bp, and the average length of intron was 6,082 and 6,107 bp, respectively (Supplementary Table S11-S12). The protein-coding genes in the YFP and EFP genomes were supported by at least one evidence with a CDS overlap ratio greater than 80% at a level of 99.96% and 99.95%, respectively (Supplementary Table S13S9-S14S12). It was is worth noting that the length distribution of gene models at the levels of genes, CDS, exons and introns showed a similar trend when compared to those of YFP v1.0 (GCA_003031525.2GCF_000442215), EFP v1.0 (GCA_026225855.1) and Bottlenose Dolphin (GCF_011762595) (Supplementary Figure S3S5). In the predicted gene models of YFP v2.0 and EFP v2.0, the BUSCO analysis identified 97.5 % and 97.6% complete conserved single copy mammalian genes (odb10), respectively (Table 1 and Supplementary Figure 1ES2). In total, 22,263 (96.21%) gene models in the YFP v2.0 genome and 22,224 (96.20%) gene models in the EFP v2.0 genome were annotated in at least one database (NR, SwissProt, KEGG, KOG, TrEMBL, Gene Ontology and InterPro) (Table 1, Figure 1F1B), whereas 71.22% (16,480) of YFP v2.0 genes and 71.24% (16,457) of EFP v2.0 genes are annotated in five functional databases (NR, SwissProt, KEGG, KOG and InterPro) (Supplementary Table S15 S13 and Figure S4S6 S5). Finally, 20,–589 (88.98%) and 20,–613 (89.23%) genes from the YFP v2.0 and EFP v2.0 genomes, respectively, were determined to be transcriptionally active based on the analysis of could be transcriptionally detected by the 24 RNA-seq datasets (Figure 1F1B). In conclusion, our annotation of two gene sets of reliably high quality has established a robust foundation for further research.

Analysis of centromere related genes

The centromere is an important functional structure of eukaryotic chromosomes, and plays an

~~important~~ necessary role in ~~ensuring the correct segregation of chromosomes during~~ cell division for eukaryotes. The intrinsic mechanisms underlying~~mystery of~~ the evolution of centromere structure among different species has not been fully revealed due to the challenge of assembling highly repetitive sequences [~~23~~24]. In this study, we conducted predictions on~~detected~~ repeat monomers within the YFP v2.0 and EFP v2.0 genomes~~.~~, potentially constituting~~that may constitute~~ the centromere (Figure 2A, Figure 2B and Supplementary Table ~~S16~~S14~~-S17~~S15). The monomeric sequences vary in length from 99 to 201 bp, with the 144-bp, 150-bp, and 138-bp monomers being the most abundant. Centromeres are composed of more than one repeat monomer and are located within TE- and TR-enriched regions, which are areas with relatively lower gene density (Supplementary Figure S7). A total of 235 and 237 genes were identified in the candidate centromere regions for YFP v2.0 and EFP v2.0, respectively, through predictions generated by centromere-finding software. Moreover, the newly discovered centromere regions for YFP v2.0 hosted approximately 56 genes, while only 20 genes were found in analogous regions for EFP v2.0. The "newly identified centromere regions" refer to specific areas identified in the genome that have been recently assembled but were not included in the previously published version. This discovery may suggest the presence of novel, previously uncharacterized centromere sites. ~~In total, 235 and 237 genes were identified in the YFP and EFP candidate centromere region, respectively, while 56 and 20 genes of YFP and EFP were discovered in the newly identified centromere regions.~~ We further compared the genes located in the centromere and non-centromere regions in the genomes of YFP v2.0 and EFP v2.0, respectively, and found that there were no significant differences in the expression patterns of these genes in different transcripts (Supplementary Figure ~~3A~~S8~~, Figure 3B~~ and Supplementary Table ~~S18~~S16~~-S19~~S17). Additionally, we analyzed the functional enrichment of genes located in the centromere regions of the two genomes~~ (Supplementary Figure S6-S7)~~. ~~These genes were mainly involved in localization, locomotion, transcription receptor activity and cytoskeletal motor activity in GO enrichment (Figure 3C, Figure 3D and Supplementary Table S20-S23).~~These genes were mainly associated with Keratin filament (GO:0045095), Intermediate filament (GO:0005882), Gamete generation (GO:0007276) and Channel activity (GO:0015267), as indicated by GO enrichment (Supplementary Table S18). These findings align with those produced by analysis using the KEGG database, and highlight the essential role of centromeres in chromosome segregation and positioning (Supplementary Figure S9 and Table S19-S20).

Centromeres provide structural support to the nucleus, ensuring chromosome integrity, and are crucial in mitosis and meiosis. Moreover, centromeric regions may interact with cell cycle regulation, cell division signaling pathways, and channel activity, impacting cell division progression.

Variations between YFP and EFP genomes

Synteny analysis of the gene order between YFP v2.0 and EFP v2.0 revealed 907 large shared syntenic blocks, encompassing 89.59% (41,428) genes, and 17 chromosomal rearrangements (Figure 3). Further comparison of the genomic sequencesComparative analysis of the YFP v2.0 and EFP v2.0 genomes, however, yielded numerous variations between the two, including 3,887,060 single-nucleotide polymorphisms (SNPs) (Figure 4A) and 704,944 short insertions/deletions (IndelsInDels) (Figure 4B). The variations in SNPs were primarily located in the intergenic regions (65.28%) and intronic regions (32.66%), and rarely in the exon regions (0.64%). Similarly, variations in Indels InDels were less concentrated in exon regions (0.23%) (Supplementary Table S24S21). Of the exon region variations, 12,203 SNPs (11,987 non-synonymous SNPs, 189 stop-gain SNPs, and 27 stop-loss SNPs) and 953 Indels InDels (464 frameshift deletion, 467 frameshift insertion, 20 stop-gain IndelsInDels, and 2 stop-loss IndelsInDels) were identified and functionally associated with 6,158 and 582 genes, respectively. KEGG enrichment analysis revealed that these genes were significantly (P value < =0.05) enriched in "calcium signaling pathway", "NF-kappa B signaling pathway", "complement and coagulation cascades", "antigen processing and presentation" and "Intestinal immune network for IgA productionglycerolipid metabolism" (Supplementary Figure S10 and Figure 4C and Supplementary Table S25S22).

Here, tThe genes coding for the mutated regions of the YFP and EFP are widely enriched in immune-related pathways, . This association may be intricately linked to the distinct pathogenic microorganisms unique to freshwater and seawater environments. Marine mammals exhibit a diminished histocompatibility complex (MHC) diversity attributed to decreased encounters with microparasitic diversity in their marine habitat relative to their terrestrial origin. This phenomenon implies that mammals encounter distinct pathogenic pressures in varied ecological settings, potentially influencing the evolution of immune-related genes [25]. Evolutionary analyses of the innate immune pattern recognition receptor (TLRs) in the YFP and the marine finless porpoise indicate that the YFP has undergone specific adaptive changes [26].which may be closely related to their different habitats in freshwater and seawater, respectively. The microbial diversitycategories

and pathogenicity of ~~of~~ freshwater and seawater environments vary~~, differ greatly~~, leading to distinct ~~and the~~ effects of pathogenic microorganisms on the organisms in these two types of environments[27]~~are also different~~. Therefore, the YFP and EFP would be expected to ~~will~~ undergo adaptive evolution ~~in order~~ to adapt to the pathogen stresses specific to their respective ecological environments, freshwater and seawater~~of the two ecological environments, respectively, in freshwater and seawater~~. ~~Alterations in a considerable number of immune-related genes may be important to facilitate the adaptation of YFP and EFP to freshwater and seawater habitats, respectively.~~

~~Specifically, b~~By comparing the assembly results of the YFP and EFP versions, it was found that the YFP v2.0 ~~assembled~~ assembly included 5,480 new genes, while the EFP v2.0 ~~assembled~~ assembly included 1,453 new genes compared to the previous version of the assembly (Table 1 and Supplementary Table ~~S26~~S23). These genes were expressed in all 24 samples (Supplementary Figure ~~S8~~S11~~-S9~~ and Table ~~S27~~S24~~-S28~~S25). GO and KEGG functional enrichment analysis indicated that these genes are primarily enriched in immunity and iron binding, among other functions. These biological processes encompass a wide range of aspects, including cellular structure and function, protein synthesis, signal transduction, immune response, and energy metabolism (Supplementary Table S26-S29).~~GO functional enrichment analysis discovered that these genes are mainly enriched in cellular process, metabolic process, cellular anatomical entity, binding and catalytic activity (Figure 4D, Figure 4E, Supplementary Table S29-S32 and Figure S10-S11).~~

Phylogeny ~~and synteny~~ analysis

Gene family analysis was performed on 506,098 protein coding sequences from ten cetaceans and sixteen terrestrial species of mamal~~mammals~~, and clustered into 22,196 gene families, which ~~including~~ include 594 species-specific genes in YFP v2.0 and EFP v2.0 (Supplementary ~~Table S7 and~~ Table ~~S33~~S30). Among the dataset, a total of 2,161 single-copy gene families were identified. Subsequently, multiple sequence alignments were conducted, which were then followed by the reconstruction of evolutionary trees. The resulting phylogenetic tree produced a topology consistent with previous studies [28], highlighting its reliability and alignment with established scientific knowledge. This analysis notably revealed the topological arrangement within branches of mammals. The emergence of the EFP branch alongside its YFP counterpart was particularly

Formatted: Not Highlight

Formatted: Not Highlight

noteworthy.~~A total of 2,161 single-copy orthologous genes were aligned using MAFFT~~ (Supplementary Figure ~~S12~~S12). Our analysis had revealed that the divergence time between the YFP and EFP ranges from 0.5 to 1.1 million years ago (Figure 5A, Figure 5B and Supplementary Figure ~~S13~~S13), which is the first estimate at the molecular level since their classification as two distinct subspecies.

~~Synteny analysis demonstrated that YFP displayed a greater level of conservation than EFP (Figure 1B). We observed similar patterns in the distribution of gene frequency, gene density, TE density, and GC density between the two genomes, with most of their chromosomes being aligned with each other. Notably, certain chromosomes in the YFP genome were found to match multiple chromosomes in the EFP genome, indicating that chromosomal rearrangements occurred in both genomes after their speciation.~~

Gene family and positive selection analysis

We used CAFÉv4.0 to analyze the evolution of gene families based on orthologous clusters of protein coding sequences from twenty-six mammals. ~~When~~ Upon comparing the genomes of YFP v2.0 and EFP v2.0 with their most ~~last~~recent common ancestor, it was ~~found~~ observed that 843 gene families underwent expansion ~~expanded~~ while 98 gene families experienced contraction~~contracted~~ (Figure 5A and Figure 5B). ~~Out of~~Among the 215 expanded gene families identified in the YFP v2.0 and EFP v2.0 lineage, a total of 2,674 genes were ~~found~~ determined to be ~~involved with statistical significance~~ significantly associated (P<0.05) (Supplementary Table ~~S34~~S31). We observed an expansion for genes significantly enriched in several KEGG pathways, including “antigen processing and presentation”, “intestinal immune network for IgA production”, “oxidative phosphorylation”, and the “calcium signaling pathway” (Figure ~~5C~~6A). The significantly enriched GO terms, ~~including~~ included “ferric iron binding”, “iron ion transport”, “riboflavin biosynthetic process”, “tetrahydrofolate biosynthetic process” and “cytochrome-c oxidase activity”, ~~were~~ also expanded (Figure ~~5D~~6B). Gene families associated with “oxidative phosphorylation”, “cytochrome-c oxidase activity”, “riboflavin biosynthetic process”, “ferric iron binding” and “iron ion transport” exhibited expansion in finless porpoises. Iron ions play a crucial role in numerous essential physiological functions within living organisms, such as oxygen transportation, electron transport chains, and various metabolic pathways. Several sets of redox reactions are necessary to maintain effective gas exchange in water for cetaceans, and iron is often used in these reactions as

an electron acceptor [29]. The expansion of redox reaction and iron ion gene families in cetaceans potentially improved the efficacy of oxygen utilization and facilitated adaptation to hypoxic conditions in aquatic habitats. An increase in the number of oxidation-reduction and iron-binding gene families was also observed in *L. vexillifer*, a species that encounters hypoxic conditions during dives [30]. The changes in pathogenic microorganisms that occur during the reintroduction of cetaceans from land to sea present a significant challenge to their survival and may have influenced the evolution and adaptation of immune genes [31]. The expansion of immune-related gene families in finless porpoises in this study enhanced the process of antigen presentation and conferred resistance against various pathogenic microorganisms amidst changes in their habitat. Genomic studies of the *S. chinensis* have revealed that cetaceans have developed various species-specific gene families related to immunity and DNA repair, which are linked to potential adaptive mechanisms [32]. We ~~postulated~~ suggest ~~that~~ these genes play~~ed~~ a crucial role in facilitating hypoxic tolerance and ~~regulating osmotic pressure,~~ enhancing immune resistance ~~and facilitating hypoxic tolerance~~ in finless porpoises, thereby reflecting potential mechanisms of adaptation to the aquatic environment. Further investigations are required to elucidate the specific functions of these gene families and their potential significance in the biology of finless porpoises.

The Codeml program in PAML with a branch-site model was employed to test for ~~for~~ selective pressure ~~analyses~~ based on orthologous clusters of 10 cetaceans, including *Neophocaena asiaeorientalis asiaeorientalis* (Yangtze finless porpoise), *Neophocaena asiaeorientalis sunameri* (East Asian finless porpoise), *Tursiops truncatus* (Bottlenose dolphin), *Orcinus orca* (Killer whale), *Lipotes vexillifer* (Yangtze River dolphin), *Physeter catodon* (Sperm whale), *Balaenoptera acutorostrata* (Minke whale), *Balaena mysticetus* (Bowhead whale), *Delphinapterus leucas* (Beluga whale), *Sousa chinensis* (Indo-Pacific humpback dolphin) ~~Yangtze finless porpoise, East Asian finless porpoise, Bottlenose dolphin, Killer whale, Yangtze River dolphin, Sperm whale, Minke whale, Bowhead whale, Beluga whale, Chinese white dolphin~~. We identified 41 positively selected genes (PSGs) in the YFP lineage, which were functionally enriched in “RNA degradation”, “nucleotide excision repair”, “DNA replication”, “mismatch repair”, and “homologous recombination” pathways (*P*<0.05) (Figure ~~5E~~ 6C and Supplementary Table ~~S35~~S32). The selective pressure to evolve DNA damage repair pathways implied that the Yangtze finless porpoise might be experiencing increased threats to genome stability. The mechanism of DNA damage repair plays a

crucial role in preserving genome integrity by enabling cells to identify and repair DNA damage, thereby averting the accumulation of harmful mutations [33]. In a comparative genomic analysis between the South China tiger and the Amur tiger, it was noted that genes related to DNA repair underwent positive selection in the South China tiger [21]. The observed phenomenon could be explained by the higher probability of genome instability in the temperate and subtropical habitats of the South China tiger. This may be linked to metabolites generated by intestinal microflora, which possess the ability to trigger DNA damage [34]. The stability of the genome or gene expression system in the Yangtze finless porpoise across different organs and life stages remains uncertain. However, one potential interpretation of this data is the suggestion that the Yangtze finless porpoise could be vulnerable to genomic instability triggers in the Yangtze River, such as water pollutants, which may increase the likelihood of DNA damage [35]. Pollutants found in the Yangtze River possess the capacity to accumulate within the food chain, leading to cellular DNA damage and impacting the genome stability of the Yangtze finless porpoise [36, 37]. This makes a compelling case for the improved conservation of the species and the development of more rigorous water pollution mitigation practices.

~~The evolution of DNA damage repair pathways implied the existence of additional triggers for genomic instability in the Yangtze River, including human activities such as wading projects, dredging and quarrying.~~ Interestingly, a total of 44 PSGs within the EFP lineage were involved in “sodium-dependent phosphate transport”, “sodium symporter activity”, “aldosterone-regulated sodium reabsorption”, and “calcium signaling pathway” (Figure ~~5E~~ 6D and Supplementary Table ~~S36~~S33). These metabolic pathways play an important role in regulating sodium levels in the body [38]. Among these PSGs, six were potentially associated with the adaptation of EFP to high osmolarity environment, including the Na(+)/H(+) exchange regulatory cofactor ~~(~~*NHE-RF2*~~)~~ and sodium-dependent phosphate cotransporter ~~(~~*SLC34*~~)~~. These genes identified are likely to have significant implications in the control of urine formation and the preservation of water-salt metabolic balance [38], suggesting that the East Asian finless porpoise may have a different urine formation process. Previous genome-selective sweep analyses in the finless porpoises revealed that the *SLC14A* in the East Asian finless porpoise underwent positive selection [14]. Comparative analyses conducted at the transcriptome level between the Yangtze finless porpoise and the East Asian finless porpoise revealed a notable upregulation of the *NHE3* in the kidneys of the East Asian

finless porpoise. These results imply some adaptive enhanced osmoregulatory capability in the East Asian finless porpoise [39]. The aforementioned studies suggest that the East Asian finless porpoise has evolved a complex and efficient osmoregulatory mechanism as it acclimatized to the hypertonic marine environment, demonstrating adaptations at both molecular and transcriptional levels.~~These indicated that Yangtze and East Asian finless porpoises may possess distinct adaptation strategies to their aquatic environment, which warrant further investigation.~~

Methods

Sample collection, DNA extraction, and sequencing

We collected an adult dead female YFP sample from Lianzhou Lake, Anqing City, Anhui Province, China (N30°15′32″, E116°54′38″) in 2021 and a dead juvenile male EFP sample from the Yellow Sea near Lianyungang City, Jiangsu Province, China (N34°55′27″, E119°11′37″) in 2019 for sequencing (Figure 1A). The Office of Fishery Supervision and Management in the Yangtze River Basin, Ministry of Agriculture and Rural Affairs of the People's Republic of China has designated our research institution to perform post-mortem analysis and genetic preservation on deceased porpoises. No ethical considerations were taken into account in this study. DNA were extracted from muscle tissues following the phenol/chloroform DNA extraction method. DNA extracted from the YFP was utilized to construct PacBio HiFi, Hi-C, and Oxford Nanopore Technologies (ONT) libraries. DNA extracted from an EFP was utilized to construct an ONT library. ~~According to the manufacturer's instructions (QIAGEN, Germany), a~~The PacBio HiFi library was constructed using SMRTbell Prep Kit 3.0 (Pacific Biosciences, USA)~~a QIAGEN Blood & Cell Culture DNA Midi Kit~~ and subsequently sequenced on the PacBio Sequel II system in circular consensus sequence (CCS) mode. To collect data for the Hi-C library, the muscle tissues were first fixed in 1% formaldehyde (Sigma) for cross-linking and resuspended in lysis buffer. Then, MboI (NEB) restriction endonucleases were used to fragment the chromatin in the muscle to fragment DNA. The DNA fragments were captured by utilizing Streptavidin-coated magnetic beads (Thermo Fisher SCIENTIFIC) following biotin labeling and crosslinking using T4 DNA Ligase (ENZYMATICS). The Hi-C library was finally sequenced on a BGI MGISEQ platform. ~~A Hi-C library was generated using the *Mbo* I restriction enzyme and subsequently sequenced on BGI MGISEQ platform.~~ To generate and sequence ONT libraries, we isolated genomic DNA using the CTAB[40] method,

selected fragments exceeding 5 kb in size with the SageHLS HMW library system (Sage Science), processed the DNA with the Ligation sequencing 1D kit (SQK-LSK109, Oxford Nanopore Technologies, Oxford, UK), and subsequently sequenced the ONT libraries on a PromethION platform (Oxford Nanopore Technologies) at the BGI (Wuhan, China).

Gap-free genome assembly and quality assessment

We ~~utilized~~ utilized SMRTLink v11.0.0 (https://www.pacb.com/support/software-downloads) ~~to~~ to filter PacBio HiFi reads, applying the following criteria: a minimum requirement of three full-length subreads for generating CCS, a draft length threshold of 500 bp before polishing, and aiming for a predicted accuracy of 0.99.~~polish the PacBio HiFi reads,~~ To filter Hi-C reads, index reads were removed and reads were filtered using the following SOAPNUKE v2.0 [~~24~~41] parameters: N rate >= 0.01, low quality <= 20, low quality rate >= 0.1.~~to filter the Hi-C reads,~~ Subsequently, ONT reads were filtered based on a length < 5 kb and a quality value < 7.~~and self-designed Perl programs to refine the ONT reads. The errors in the cleaned ONT reads were subsequently corrected using~~T ~~t~~he Necat pipeline~~.~~ (v 20200119) [~~25~~42] was utilized for the enhancement of ONT reads. This was achieved through the application of error correction algorithms to evaluate quality scores, k-mer frequencies, and alignment methods. This process enhanced the accuracy of reading and generated refined results suitable for further analyses. To achieve gap-free chromosome-level assemblies, we employed both the Hifiasm (v0.15.1) [~~26~~43] and Necat pipeline (v20200119) [~~25~~42] to separately assemble PacBio HiFi reads and ONT corrected reads into the initial contigs. The Purge-Haplotigs program [~~27~~44] ~~program~~ was utilized to eliminate redundant contigs that exhibited similar sequences but distinct haplotypes, specifically targeting those with aligned coverage below 30%. This strategic approach significantly optimizes the assembly process by removing redundant information, thus improving the accuracy of genome assembly.~~was applied to remove redundant contigs with parameters "-j 80 -s 80 -a 30".~~ We utilized Hi-C data to cluster, order, and orient the contigs into pseudo-chromosomes though the implementation of the Juicer (v1.5) [~~28~~45] and 3D-DNA (v180922) [~~29~~46] pipelines~~ for clustering, ordering, and orienting the contigs into pseudo-chromosomes using Hi-C data~~. ~~Additionally,~~ Ultra-long ONT reads and contigs were used to generate gapless scaffolds ~~we utilized ultra-long ONT reads and contigs to bridge the gaps in the gapless PacBio assembly~~ through the LR_Gapcloser (v1.0) [~~30~~47] and TGSgapcloser (v 1.0.1)[~~31~~48] pipelines.

Various metrics~~methods~~ were ~~employed~~ used to evaluate the quality of the gap-free genome assemblies, including contiguity, accuracy~~correctness~~, and completeness. First, we calculated the length metrics of genomic sequences to evaluate contiguity and subsequently used Merqury (v1.3) [~~32~~23] with k-mer set to 21 to assess the accuracy. Second, Benchmarking Universal Single-Copy Orthologs (BUSCO) [~~33~~49] evaluation ~~were~~ was conducted to assess the completeness. Third, we also mapped PacBio HiFi, ONT and RNA-seq data into the genome assemblies using Minimap2 [~~34~~50] and Hisat2 (v2.1.0) [~~35~~51] to assess the completeness. In addition, we utilized the quartet pipeline [~~36~~52] to search for telomere repeat sequences and centromere regions in YFP and EFP.

~~Gene structure~~Genome annotation

Repetitive sequence~~s~~ annotation was identified ~~by~~ using both *de novo* and homology-base approaches~~prediction~~. For the *de novo* strategy~~prediction~~, RepeatModeler (v1.0.4) [~~37~~53] was employed to identify repetitive elements, whereas ~~and~~ LTR-FINDER (v1.0.7) [~~38~~54] was used for the ~~were~~ specific annotation of~~conducted to identify repetitive elements and annotate~~ long terminal repeats~~, respectively~~. For homolog-based prediction, ~~DNA and protein transposable elements (TEs) were detected by~~ RepeatMasker (v4.0.7) [~~39~~55] was utilized to detect DNA transposable elements (TEs), while~~and~~ RepeatProteinMasker (v4.0.7) was employed to identify protein-based TEs, both based on the Repbase database.~~, respectively.~~ Additionally, Tandem Repeat Finder (v4.10.0) [~~40~~56] was used to identify Tandem repeats. The utilization of these tools facilitated a comprehensive annotation of repetitive sequences, leading to a substantial improvement in the accuracy and detail of the analysis.

A combination of RNA-seq, homology-based and *de novo* prediction strategies was utilized to identify protein-coding genes in the genomes of YFP and EFP. RNA-seq data [15, 16] were mapped to genome ~~assembly~~ assemblies with Hisat2 v2.1.0 [~~41~~57] with the following parameters: --sensitive --no-discordant --no-mixed -I 1 -X 1000 --max-intronlen 1000000. The produced BAM alignments were further assembled into gene models with StringTie v1.3.5 [~~42~~58] with the following parameters: -f 0.3 -j 3 -c 5 -g 100 -s 10000 and validated using PASA v2.5.2 [~~43~~59]. The coding sequences were identified by TransDecoder (v5.5.0) (https://github.com/TransDecoder/TransDecoder) with default parameters. Utilizing 149,956 genes from 8 closely related cetacean species and transcriptomic sequencing data from 24 YFPs as input files, the ~~For gene content assessment, 8 homologous proteins and 24 RNA-seq data were used~~

~~(Supplementary Table S9-S10).Combing RNA-seq data with homolog sequences of eight cetaceans (Supplementary Table S7), the homology-like coding sequences were predicted using~~ GeMoMa v1.9 [~~44~~60] software was employed to conduct homology-based prediction analysis (Supplementary Table S7, S34-S35). A set of ~~O~~one thousand high quality genes, which were predicted by the GeMoMa software and validated by OrthoDB for mammals, were randomly selected for training~~filtered out to train~~ the predictors ~~by~~ in Augustus v3.2.1 [~~45~~61]. The Augustus v3.2.1 program was used to perform *de novo* prediction. We used GeMoMa software to integrate all predicted protein-coding genes, and annotated them with NR, Swissprot [~~46~~62], KEGG [~~47~~63], KOG, TrEMBL, InterPro [~~48~~64] and GO [~~49~~65] databases.

Genome comparison and Identification of newly assembled genes

Single nucleotide polymorphisms (SNPs) and insertions/deletions (InDels) were identified using methods following those previously described. Genome alignment was conducted utilizing the NUCmer program integrated within MUMmer4 (v4.0.0) [66] to compare the v2.0 assembly with the v1.0 assembly, as well as the YFP assembly with the EFP assembly. Utilizing the Maximum Unique Matches (MUM) mode involved setting parameters such as a minimum MUM length of 1000 bp, a minimum similarity threshold of 90%, and the exclusion of matches below 40 bp. Alignment blocks were identified using the delta-filter program, while SNPs and InDels were detected using the show-snps program and Syri (v1.6.3) [67], respectively. Functional annotation of SNPs and InDels is conducted using ANNOVAR [**Error! Reference source not found.**] to assess their impacts on gene structure and function. Additionally, we utilize the CMplot R package [**Error! Reference source not found.**] to visually depict the density distribution of these genetic variations. The objective of these analyses was to reveal structural variations between the v1.0 and v2.0 genome assemblies, as well as between the YFP and EFP genome assemblies. Variants were meticulously annotated utilizing the ANNOVAR package (v 2013-06-21) [68].

~~With the v2.0 genome assembly as a reference, we utilized the software MUMmer [50] and Syri (v1.6.3) [51] to explore the structural variations between the v1.0 and v2.0 genome assemblies. Genes were categorized as newly assembled if the gene region of first draft genome assembly exhibited a deletion of at least 50 bp and a minimum overlap of 30% within that region. The package ANNOVAR (v 2013-06-21) [52] was utilized to annotate the variations.~~Genes were categorized as newly assembled when~~if~~ the gene region in the initial ~~of first~~ draft genome assembly

~~exhibited~~showed a deletion of at least 50 bp and had a minimum overlap of 30% within that specific region.

~~**Identification of new assembled genes**~~

~~The software Syri (v1.6.3) was employed to detect structural variations between the v2.0 genome assembly and the previously published genome assembly of finless porpoises. A gene was classified as newly assembled if the previously published genome assembly exhibited a deletion of at least 50 bp and the gene region had a minimum overlap of 30% with that region.~~

Gene family and phylogenomic analysis

Gene families of 26 species (Supplementary Table ~~S7~~S30) were identified and clustered by OrthoFinder (v2.3.11) [~~53~~69]. Single-copy orthologous genes (1:1:1) were aligned using MAFFT [~~54~~70] (v7.310), a widely utilized tool for multiple sequence alignment. Subsequently, ~~and~~ a maximum-likelihood phylogenetic tree was ~~constructed with~~generated using PhyML (v3.3) [~~55~~71], a commonly employed software tool for phylogenetic analysis. ~~using~~ The HKY85 model, known for its capacity to accommodate nucleotide substitution patterns, was applied in the tree construction procedure. To evaluate the reliability of the generated tree, 1000 bootstrap replications were performed, providing statistical evidence for the branching structures. ~~Consistent phylogeny with previous study was demonstrated by all branches, as evidenced by 100/100 bootstrap support. [56].~~ Species divergence time was calculated using MCMCTREE in PAML (v4.9) [~~57~~72]. Four divergence time points from TimeTree (http://timetree.org.cn) were used to calibrate the divergence times: (a) *Ornithorhynchus anatinus* (Platypus) and *Monodelphis domestica* (Opossum) (163.7–185.9 Ma), (b) *Homo sapiens* (Human) and *Mus musculus* (Mouse) (81.3–91.0 MYA), (c) *Balaena mysticetus* and *Balaenoptera acutorostrata* (21.3–28.8 Ma) and (d) *Sousa chinensis* and *Tursiops truncatus* (2.0–3.8 Ma). The core-orthologous gene sets were identified by ~~Blast~~ BLAST (v2.0.14) [~~58~~73] with an E-value threshold of $1\times E^{-10}$ (at least 10 syntenic genes allowed), and ~~defined~~ syntenic blocks were defined ~~by~~ using MCscanX v1.5.2 [~~59~~74]. Circos was used to plot the synteny results.

Gene family expansion and contraction analysis

Protein sequences of YFP, EFP and 24 published mammals were used to search homologs. Based on the gene families clustered by OrthoFinder, the CAFÉ (v4.0) [~~60~~75] software was used to perform expansion and contraction analyses in ~~branch of~~the finless porpoises clade. Random birth

and death models were employed to study gains and losses of gene families in a user-specified phylogeny. The global parameter λ, which describes both the gene birth (λ) and death (μ = −λ) rate for gene families in all branches of the tree, was estimated using maximum likelihood. Then the P-value was calculated for each gene family, and P-value ≤0.01 was defined as a "significantly expanded ~~and~~ or contracted gene family". KEGG and GO enrichment analyses were conducted among these significantly expanded and contracted gene ~~family~~families.

Gene positive selection analysis

Protein sequences of two finless porpoise and ~~other 8 published~~another eight cetaceans were used to identify single copy orthologs with OrthoFinder. Then Ka/Ks ratios for these single copy orthologs were calculated by following steps. Initially, the single-copy orthologs underwent global alignment using PRANK. Subsequently, alignment refinement via Gblocks was utilized to remove inadequately aligned positions and divergent regions, thereby isolating conserved blocks from the multiple alignment. Codeml from the PAML package ~~Firstly, global alignment among these single copy orthologs was executed by PRANK and then filtered the alignment with Gblocks. Finally, Ka/Ks ratios on different branches was calculated by Codeml in the PAML package~~ [~~57~~72] was ultimately employed to compute Ka/Ks ratios across various branches, utilizing the free-ratio model. ~~with the free-ratio model.~~ Genes that showed values of Ka/Ks higher than 1 along the branch~~,~~ leading to finless porpoise were reanalyzed using the codon-based branch site tests implemented in PAML (PAML, RRID:SCR_014932). The branch site model allowed ω to vary both among sites in the protein and across branches, and it was used to detect episodic positive selection.

Gene expression analysis

The raw RNA-seq reads underwent quality control using SOAPnuke (v2.0). Reads were filtered out if they had an N rate ≥ 0.01, low quality ≤ 20, low quality rate ≥ 0.1, or contained index sequences. Subsequently, the clean reads were aligned to the EFS v2.0 genome utilizing the Hisat2 high sensitivity model, excluding discordant pairs and mixed alignments. The alignment process involved setting a minimum insert size of 1 bp and a maximum insert size of 1000 bp.~~The raw RNA-seq reads were quality controlled by SOAPnuke (v2.0), and the clean reads were subsequently aligned to the EFS v2.0 genome using Hisat2 (v2.1.0), with the following parameters: '--phred33 -p 5 --sensitive --no-discordant --no-mixed -I 1 -X 1000'.~~ We utilized featureCounts [~~61~~76] and transcripts per million (TPM) method to generate an estimated mapped read count matrix and

calculate the gene expression level, respectively.

Conclusion

The availability of reliable chromosome-level genome ~~assembly~~ assemblies provide~~s~~ remarkable improvements in identifying genes, characterizing genomic regions and performing comparative genomic analyses. In the present study, we assembled ~~two~~ telomere-to-telomere and gap-free Yangtze finless porpoise and the East Asian finless porpoise genomes by combining PacBio long reads, Hi-C and short-read sequencing technologies. The new assemblies have higher contiguity and completeness, as well as more complete single-copy BUSCO genes with fewer fragmented or missing genes than the first drafts. ~~Genome synteny analysis revealed a robust collinear relationship between the Yangtze finless porpoise and the East Asian finless porpoise. Reconstructing ancestral chromosomes enabled the identification of chromosomal rearrangement events in the finless porpoise.~~ The reconstructed phylogeny determined that ~~the~~ YFP and EFP ~~constitute a clade and~~ diverged approximately 0.5-1.1 million years ago (Ma). Gene family expansion analysis revealed significantly enriched pathways and GO terms associated with the regulation of immune resistance and hypoxic tolerance. Selection pressure analysis identified genes associated with DNA damage repair in the YFP and high salt tolerance in the EFP, respectively. Our results provide evidence of the gradual adaptation of EFP to a marine environment and the potential sensitivity of YFP to genome damage. Identification ~~The acquisition~~ of the centromere, telomere, and associated genes can serve as valuable resources for a comprehensive~~ly~~ understanding of chromosome stability, ~~undesired~~ recombination, repair mechanisms, and evolutionary processes. Overall, ~~this is~~these are the most continuous cetacean genome ~~assembly~~ assemblies to date, with chromosome-scale contigs and no gaps. This study will lay a foundation for population genomics studies at the whole genome level, and deepen the scientific understanding of issues related to population conservation and adaptation mechanisms.

DATA AVAILABILITY

Raw sequencing data and genome assemblies in this study have been deposited in the NCBI database (BioProject ID PRJNA915046 and PRJNA859258). Furthermore, results of repeat annotation, gene structure annotation and gene functional annotation had been deposited in the *figshare* https://figshare.com/s/1fc632fd4f3cab36b776.

COMPETING INTERESTS

The authors declare that they have no competing interests.

AUTHORS' CONTRIBUTIONS

K.L., J.~~B.~~J. and P.X. designed and conceived the study. D.~~H.~~Y., C.~~P.~~Y. and J.~~L.~~Z. collected and prepared the samples. C.~~H.~~C. and C.~~X.~~Z. performed the data analysis. D.~~H.~~Y., C.~~H.~~C. and J.~~B.~~J. wrote the manuscript with significant contributions from Y.L., Z.~~C.~~C., H.Z., C.~~H.~~W. and L.~~P.~~L. K.L., Z.H. and D.~~Q.~~L. provided the financial support. All authors read and approved the final version of the manuscript.

ACKNOWLEDGMENTS

This work was funded by the National Key R&D Program of China (2021YFD1200304), the Central Public-interest Scientific Institution Basal Research Fund, Freshwater Fisheries Research Center, CAFS (2021JBFM15) and Project of Implementation of Yangtze Finless Porpoise Protection in the Middle and Lower Reaches of Yangtze River (2021).

REFERENCES

1. Gao, A and Zhou, K. Growth and reproduction of three populations of finless porpoise, *Neophocaena phocaenoides*, in Chinese waters. *Aquat Mamm* 1993;**19** (1):3-12.
2. Jefferson, T. Preliminary analysis of geographic variation in cranial morphometrics of the finless porpoise (*Neophocaena phocaenoides*). *Raffles Bull Zool* 2002;**10**:3-14.
3. Wang, P. The morphological characters and the problem of subspecies identifications of the finless porpoise. *Fish Sci* 1992;**11**:4-8.
4. Gao, A and Zhou, K. Geographical variation of external measurements and three subspecies of *Neophocaena phocaenoides* in Chinese waters. *Acta Theriol Sin* 1995;**15**(2):81-92.
5. Wang, J, Frasier, T, Yang, S, *et al*. Detecting recent speciation events: the case of the finless porpoise (genus *Neophocaena*). *Heredity* 2008;**101**(2):145-55.
6. Jefferson, T and Wang, J. Revision of the taxonomy of finless porpoises (genus *Neophocaena*): The existence of two species. *J Mar Anim Ecol* 2011;**4**(1):3-16.
7. Yang, G, Ren, W, Zhou, K, *et al*. Population genetic structure of finless porpoises, *Neophocaena phocaenoides*, in Chinese waters, inferred from mitochondrial control region sequences. *Marine mammal science* 2002;**18**(2):336-47.
8. Xu, S, Sun, P, Zhou, K, *et al*. Sequence variability at three MHC loci of finless porpoises (*Neophocaena phocaenoides*). *Immunogenetics* 2007;**59**(7):581-92.
9. Chen, M, Zheng, J, Wu, M, *et al*. Genetic diversity and population structure of the critically endangered Yangtze finless porpoise (*Neophocaena asiaeorientalis asiaeorientalis*) as revealed by mitochondrial and microsatellite DNA. *Int J Mol Sci* 2014;**15**(7):11307-23.
10. Chen, M, Fontaine, M, Chehida Y, *et al*. Genetic footprint of population fragmentation and contemporary collapse in a freshwater cetacean. *Sci Rep* 2017;**7**(1):14449.
11. Lin, W, Frère, C, Karczmarski, L, *et al*. Phylogeography of the finless porpoise (genus *Neophocaena*): testing the stepwise divergence hypothesis in the northwestern Pacific. *Sci Rep* 2014;**4**:6572.
12. Li, S, Xu, S, Wan, H, *et al*. Genome-wide SNP and population divergence of finless porpoises. *Genome Biol Evol* 2013;**5**(4):758-68.
13. Zheng, J, Xia, J, He, S, *et al*. Population genetic structure of the Yangtze finless porpoise (*Neophocaena phocaenoides asiaeorientalis*): implications for management and conservation. *Biochem Genet* 2005;**43**(5-6):307-20.
14. Zhou, X, Guang, X, Sun, D, *et al*. Population genomics of finless porpoises reveal an incipient cetacean species adapted to freshwater. *Nat Commun* 2018;**9**(1):1276.
15. Yin, D, Lin, D, Guo, H, *et al*. Integrated analysis of blood mRNAs and microRNAs reveals immune changes with age in the Yangtze finless porpoise (*Neophocaena asiaeorientalis*). Comp *Biochem Physiol B Biochem Mol Biol* 2021;**256**:110635.
16. Liu, W, Yin, D, Lin, D, *et al*. Blood Transcriptome Analysis Reveals Gene Expression Differences between Yangtze Finless Porpoises from Two Habitats: Natural and Ex Situ Protected Waters. *fishes* 2022;**7**:96.
17. Nurk, S, Koren, S, Rhie, A, *et al*. The complete sequence of a human genome. *Science* 2022;**376**(6588):44-53.
18. Rhie A, Nurk S, Cechova M, *et al*. The complete sequence of a human Y chromosome. *Nature* 2023;**621**(7978):344-54.
19. Huang, Z, Xu, Z, Bai, H, *et al*. Evolutionary analysis of a complete chicken genome. *Proc*

Natl Acad Sci U S A 2023;**120**(8):e2216641120.
20. Xue, L, Gao, Y, Wu, M *et al*. Telomere-to-telomere assembly of a fish Y chromosome reveals the origin of a young sex chromosome pair. *Genome Biol* 2021;**22**(1):203.
21. Zhang, L, Lan, T, Lin, C, *et al*. Chromosome-scale genomes reveal genomic consequences of inbreeding in the South China tiger: A comparative study with the Amur tiger. *Mol Ecol Resour* 2023;**23**(2):330-47.
22. Shukla, H, Suryamohan, K, Khan, A, *et al*. Near-chromosomal de novo assembly of Bengal tiger genome reveals genetic hallmarks of apex predation. *Gigascience* 2022;**12**:giac112.
23. Rhie, A, Walenz, B, Koren, S, *et al*. Merqury: reference-free quality, completeness, and phasing assessment for genome assemblies. *Genome Biol* 2020;**21**(1):245.
24. Zhang, A, Kong, T, Sun, B, *et al*. A telomere-to-telomere genome assembly of Zhonghuang 13, a widely-grown soybean variety from the original center of Glycine max. *The Crop Journal* 2023. doi:https://doi.org/10.1016/j.cj.2023.10.003.
25. Slade, R and McCallum, H. Overdominant vs. frequency-dependent selection at MHC loci. *Genetics* 1992;**132**(3):861–64.
26. Tian, R, Chen, M, Chai, S, *et al*. Divergent Selection of Pattern Recognition Receptors in Mammals with Different Ecological Characteristics. *J Mol Evol* 2018;**86**(2):138-49.
27. Lokesh, J and Kiron, V. Transition from freshwater to seawater reshapes the skin-associated microbiota of Atlantic salmon. *Sci Rep* 2016;**25**(6):19707.
28. Yuan, Y, Zhang, Y, Zhang, P, *et al*. Comparative genomics provides insights into the aquatic adaptations of mammals. *Proc Natl Acad Sci U S A* 2021;**118**(37):e2106080118.
29. Guo, B, Sun, Y, Wang, Y, *et al*. Evolutionary genetics of pulmonary anatomical adaptations in deep-diving cetaceans. *BMC Genomics* 2024;**25**(1):339.
30. Zhou, X, Sun, F, Xu, S, *et al*. Baiji genomes reveal low genetic variability and new insights into secondary aquatic adaptations. *Nat Commun* 2013;**4**:2708.
31. Li, L, Rong, X, Li, G, *et al*. Genomic organization and adaptive evolution of IGHC genes in marine mammals. *Mol Immunol* 2018;**99**:75-81.
32. Ming, Y, Jian, J, Yu, F, *et al*. Molecular footprints of inshore aquatic adaptation in Indo-Pacific humpback dolphin (*Sousa chinensis*). *Genomics* 2019;**111**(5):1034-42.
33. Chatterjee, N and Walker, G. Mechanisms of DNA damage, repair, and mutagenesis. *Environ Mol Mutagen* 2017;**58**(5):235-63.
34. Puschhof, J and Sears, C. Microbial metabolites damage DNA. *Science* 2022;**378**(6618):358-59.
35. Lv, W, Gu, H, He, D, *et al*. Polystyrene nanospheres-induced hepatotoxicity in swamp eel (*Monopterus albus*): From biochemical, pathological and transcriptomic perspectives. *Sci Total Environ* 2023;**893**:164844.
36. Zhang, K, Qian, Z, Ruan, Y, *et al*. First evaluation of legacy persistent organic pollutant contamination status of stranded Yangtze finless porpoises along the Yangtze River Basin, China. *Sci Total Environ* 2020;**710**:136446.
37. Xiong, X, Qian, Z, Mei, Z, *et al*. Trace elements accumulation in the Yangtze finless porpoise (*Neophocaena asiaeorientalis asiaeorientalis*)-A threat to the endangered freshwater cetacean. *Sci Total Environ* 2019;**686**:797-804.
38. Shoemaker, V and Nagy, K. Osmoregulation in amphibians and reptiles. *Annu Rev Physiol*. 1977;**39**: 449-71.

39. Ruan, R, Guo, A, Hao, Y, *et al*. De novo assembly and characterization of narrow-ridged finless porpoise renal transcriptome and identification of candidate genes involved in osmoregulation. *Int J Mol Sci* 2015;**16**(1):2220-38.
40. Winnepenninckx, B, Backeljau, T, Wachter R. Extraction of high molecular weight DNA from molluscs. *Trends Genet* 1993;**9**(12): 407.
41. Chen Y, Chen Y, Shi C, *et al*. SOAPnuke: a MapReduce acceleration-supported software for integrated quality control and preprocessing of high-throughput sequencing data. *Gigascience* 2018;**7**(1):1-6.
42. Chen Y, Nie F, Xie S, *et al*. Efficient assembly of nanopore reads via highly accurate and intact error correction. *Nat Commun* 2021;**12**(1):60.
43. Cheng, H, Concepcion, G, Feng, X, *et al*. Haplotype-resolved de novo assembly using phased assembly graphs with hifiasm. *Nat Methods* 2021;**18**(2):170-5.
44. Roach, M, Schmidt, S and Borneman, A. Purge Haplotigs: allelic contig reassignment for third-gen diploid genome assemblies. *BMC bioinformatics* 2018;**19**(1):460.
45. Durand, N, Shamim, M, Machol, I, *et al*. Juicer Provides a One-Click System for Analyzing Loop-Resolution Hi-C Experiments. *Cell systems* 2016;**3**(1):95-8.
46. Dudchenko, O, Batra, S, Omer, A, *et al*. De novo assembly of the Aedes aegypti genome using Hi-C yields chromosome-length scaffolds. *Science* 2017;**356**(6333):92-5.
47. Xu, G, Xu, T, Zhu, R, *et al*. LR_Gapcloser: a tiling path-based gap closer that uses long reads to complete genome assembly. *Gigascience* 2019;**8**(1):giy157.
48. Xu M, Guo L, Gu S, *et al*. TGS-GapCloser: A fast and accurate gap closer for large genomes with low coverage of error-prone long reads. *Gigascience* 2020;**9**(9):giaa094.
49. Waterhouse, R, Seppey, M, Simão, F, *et al*. BUSCO Applications from Quality Assessments to Gene Prediction and Phylogenomics. *Mol Biol Evol* 2018;**35**(3):543-8.
50. Li, H. Minimap2: pairwise alignment for nucleotide sequences. *Bioinformatics* 2018;**34**(18):3094-100.
51. Kim, D, Paggi, J, Park, C, *et al*. Graph-based genome alignment and genotyping with HISAT2 and HISAT-genotype. *Nat Biotechnol* 2019;**37**(8):907-15.
52. Lin, Y, Ye, C, Li, X, *et al*. quarTeT: a telomere-to-telomere toolkit for gap-free genome assembly and centromeric repeat identification. *Hortic Res* 2023;**10**(8):uhad127.
53. Chen N. Using RepeatMasker to identify repetitive elements in genomic sequences. *Curr Protoc Bioinformatics* 2004;**Chapter 4**:Unit 4.10.
54. Xu, Z and Wang, H. LTR_FINDER: an efficient tool for the prediction of full-length LTR retrotransposons. *Nucleic Acids Res* 2007;**35**:W265-8.
55. Price, A, Jones, N and Pevzner, P. De novo identification of repeat families in large genomes. *Bioinformatics* 2005;**21**(Suppl 1):i351-8.
56. Benson, G. Tandem repeats finder: a program to analyze DNA sequences. *Nucleic Acids Res* 1999;**27**(2):573-80.
57. Kim, D, Langmead, B and Salzberg, S. HISAT: a fast spliced aligner with low memory requirements. *Nat Methods* 2015;**12**(4):357-60.
58. Kovaka, S, Zimin, A, Pertea, G, *et al*. Transcriptome assembly from long-read RNA-seq alignments with StringTie2. *Genome Biol* 2019;**20**(1):278.
59. Haas, B, Salzberg, S, Zhu W, *et al*. Automated eukaryotic gene structure annotation using EVidenceModeler and the Program to Assemble Spliced Alignments. *Genome Biol*

2008;**9**(1):R7.

60. Keilwagen, J, Hartung, F and Grau, J. GeMoMa: Homology-Based Gene Prediction Utilizing Intron Position Conservation and RNA-seq Data. *Methods Mol Biol* 2019;**1962**:161-77.

61. Stanke, M and Waack, S. Gene prediction with a hidden Markov model and a new intron submodel. *Bioinformatics* 2003;**19**(Suppl 2):ii215-25.

62. Bairoch, A and Apweiler, R. The SWISS-PROT protein sequence data bank and its supplement TrEMBL. *Nucleic Acids Res* 1997;**25**(1):31-6.

63. Kanehisa, M, Sato, Y, Kawashima, M, *et al*. KEGG as a reference resource for gene and protein annotation. *Nucleic Acids Res* 2016;**44**(D1):D457-62.

64. Jones, P, Binns, D, Chang, H, *et al*. InterProScan 5: genome-scale protein function classification. *Bioinformatics* 2014;**30**(9):1236-40.

65. Ashburner, M, Ball, CA, Blake, J, *et al*. Gene ontology: tool for the unification of biology. The Gene Ontology Consortium. *Nat Genet* 2000;**25**(1):25-9.

66. Marçais, G, Delcher, A, Phillippy, A, *et al*. MUMmer4: A fast and versatile genome alignment system. *PLoS Comput Biol* 2018;**14**(1):e1005944.

67. Goel, M, Sun, H, Jiao, W, *et al*. SyRI: finding genomic rearrangements and local sequence differences from whole-genome assemblies. *Genome Biol* 2019;**20**(1):277.

68. Wang, K, Li, M and Hakonarson, H. ANNOVAR: functional annotation of genetic variants from high-throughput sequencing data. *Nucleic Acids Res* 2010;**38**(16):e164.

69. Emms, D and Kelly, S. OrthoFinder: phylogenetic orthology inference for comparative genomics. *Genome Biol* 2019;**20**(1):238.

70. Nakamura, T, Yamada, K, Tomii, K, *et al*. Parallelization of MAFFT for large-scale multiple sequence alignments. *Bioinformatics* 2018;**34**(14):2490-2.

71. Guindon, S, Delsuc, F, Dufayard, J, *et al*. Estimating maximum likelihood phylogenies with PhyML. *Methods Mol Biol* 2009;**537**:113-37.

72. Yang, Z. PAML 4: phylogenetic analysis by maximum likelihood. *Mol Biol Evol* 2007;**24**(8):1586-91.

73. Altschul, S, Gish, W, Miller, W, *et al*. Basic local alignment search tool. *J Mol Biol* 1990;**215**(3):403-10.

74. Wang, Y, Tang, H, Debarry, J, *et al*. MCScanX: a toolkit for detection and evolutionary analysis of gene synteny and collinearity. *Nucleic Acids Res* 2012;**40**(7):e49.

75. Bie, T, Cristianini, N, Demuth, J, *et al*. CAFE: a computational tool for the study of gene family evolution. *Bioinformatics* 2006;**22**(10):1269-71.

76. Liao, Y, Smyth, G and Shi, W. featureCounts: an efficient general purpose program for assigning sequence reads to genomic features. *Bioinformatics* 2014;**30**(7):923-30.

1. Gao, A and Zhou, K. Growth and reproduction of three populations of finless porpoise, *Neophocaena phocaenoides*, in Chinese waters. *Aquat Mamm* 1993;**19** (1):3-12.

2. Jefferson, T. Preliminary analysis of geographic variation in cranial morphometrics of the finless porpoise (*Neophocaena phocaenoides*). *Raffles Bull Zool* 2002;**10**:3-14.

3. Wang, P. The morphological characters and the problem of subspecies identifications of the finless porpoise. *Fish Sci* 1992;**11**:4-8.

4. Gao, A and Zhou, K. Geographical variation of external measurements and three subspecies of *Neophocaena phocaenoides* in Chinese waters. *Acta Theriol Sin* 1995;**15**(2):81-92.

5. Wang, J, Frasier, T, Yang, S, *et al*. Detecting recent speciation events: the case of the finless porpoise (genus *Neophocaena*). *Heredity* 2008;**101**(2):145-55.
6. Jefferson, T and Wang, J. Revision of the taxonomy of finless porpoises (genus *Neophocaena*): The existence of two species. *J Mar Anim Ecol* 2011;**4**(1):3-16.
7. Yang, G, Ren, W, Zhou, K, *et al*. Population genetic structure of finless porpoises, *Neophocaena phocaenoides*, in Chinese waters, inferred from mitochondrial control region sequences. *Marine mammal science* 2002;**18**(2):336-47.
8. Xu, S, Sun, P, Zhou, K, *et al*. Sequence variability at three MHC loci of finless porpoises (*Neophocaena phocaenoides*). *Immunogenetics* 2007;**59**(7):581-92.
9. Chen, M, Zheng, J, Wu, M, *et al*. Genetic diversity and population structure of the critically endangered Yangtze finless porpoise (*Neophocaena asiaeorientalis asiaeorientalis*) as revealed by mitochondrial and microsatellite DNA. *Int J Mol Sci* 2014;**15**(7):11307-23.
10. Chen, M, Fontaine, M, Chehida Y, *et al*. Genetic footprint of population fragmentation and contemporary collapse in a freshwater cetacean. *Sci Rep* 2017;**7**(1):14449.
11. Lin, W, Frère, C, Karczmarski, L, *et al*. Phylogeography of the finless porpoise (genus *Neophocaena*): testing the stepwise divergence hypothesis in the northwestern Pacific. *Sci Rep* 2014;**4**:6572.
12. Li, S, Xu, S, Wan, H, *et al*. Genome-wide SNP and population divergence of finless porpoises. *Genome Biol Evol* 2013;**5**(4):758-68.
13. Zheng, J, Xia, J, He, S, *et al*. Population genetic structure of the Yangtze finless porpoise (*Neophocaena phocaenoides asiaeorientalis*): implications for management and conservation. *Biochem Genet* 2005;**43**(5-6):307-20.
14. Zhou, X, Guang, X, Sun, D, *et al*. Population genomics of finless porpoises reveal an incipient cetacean species adapted to freshwater. *Nat Commun* 2018;**9**(1):1276.
15. Yin, D, Lin, D, Guo, H, *et al*. Integrated analysis of blood mRNAs and microRNAs reveals immune changes with age in the Yangtze finless porpoise (*Neophocaena asiaeorientalis*). Comp *Biochem Physiol B Biochem Mol Biol* 2021;**256**:110635.
16. Liu, W, Yin, D, Lin, D, *et al*. Blood Transcriptome Analysis Reveals Gene Expression Differences between Yangtze Finless Porpoises from Two Habitats: Natural and Ex Situ Protected Waters. *fishes* 2022;**7**:96.
17. Nurk, S, Koren, S, Rhie, A, *et al*. The complete sequence of a human genome. *Science* 2022;**376**(6588):44-53.
18. Rhie A, Nurk S, Cechova M, *et al*. The complete sequence of a human Y chromosome. *Nature* 2023;**621**(7978):344-54.
19. Huang, Z, Xu, Z, Bai, H, *et al*. Evolutionary analysis of a complete chicken genome. *Proc Natl Acad Sci U S A* 2023;**120**(8):e2216641120.
20. Xue, L, Gao, Y, Wu, M *et al*. Telomere-to-telomere assembly of a fish Y chromosome reveals the origin of a young sex chromosome pair. *Genome Biol* 2021;**22**(1):203.
21. Zhang, L, Lan, T, Lin, C, *et al*. Chromosome-scale genomes reveal genomic consequences of inbreeding in the South China tiger: A comparative study with the Amur tiger. *Mol Ecol Resour* 2023;**23**(2):330-47.
22. Shukla, H, Suryamohan, K, Khan, A, *et al*. Near-chromosomal de novo assembly of Bengal tiger genome reveals genetic hallmarks of apex predation. *Gigascience* 2022;**12**:giac112.
23. Zhang, A, Kong, T, Sun, B, *et al*. A telomere-to-telomere genome assembly of Zhonghuang

13, a widely-grown soybean variety from the original center of Glycine max. *The Crop Journal* 2023. doi:https://doi.org/10.1016/j.cj.2023.10.003.

24. Chen Y, Chen Y, Shi C, *et al*. SOAPnuke: a MapReduce acceleration-supported software for integrated quality control and preprocessing of high-throughput sequencing data. *Gigascience* 2018;**7**(1):1-6.
25. Chen Y, Nie F, Xie S, *et al*. Efficient assembly of nanopore reads via highly accurate and intact error correction. *Nat Commun* 2021;**12**(1):60.
26. Cheng, H, Concepcion, G, Feng, X, *et al*. Haplotype-resolved de novo assembly using phased assembly graphs with hifiasm. *Nat Methods* 2021;**18**(2):170-5.
27. Roach, M, Schmidt, S and Borneman, A. Purge Haplotigs: allelic contig reassignment for third-gen diploid genome assemblies. *BMC bioinformatics* 2018;**19**(1):460.
28. Durand, N, Shamim, M, Machol, I, *et al*. Juicer Provides a One-Click System for Analyzing Loop-Resolution Hi-C Experiments. *Cell systems* 2016;**3**(1):95-8.
29. Dudchenko, O, Batra, S, Omer, A, *et al*. De novo assembly of the Aedes aegypti genome using Hi-C yields chromosome-length scaffolds. *Science* 2017;**356**(6333):92-5.
30. Xu, G, Xu, T, Zhu, R, *et al*. LR_Gapcloser: a tiling path-based gap closer that uses long reads to complete genome assembly. *Gigascience* 2019;**8**(1):giy157.
31. Xu M, Guo L, Gu S, *et al*. TGS-GapCloser: A fast and accurate gap closer for large genomes with low coverage of error-prone long reads. *Gigascience* 2020;**9**(9):giaa094.
32. Rhie, A, Walenz, B, Koren, S, *et al*. Merqury: reference-free quality, completeness, and phasing assessment for genome assemblies. *Genome Biol* 2020;**21**(1):245.
33. Waterhouse, R, Seppey, M, Simão, F, *et al*. BUSCO Applications from Quality Assessments to Gene Prediction and Phylogenomics. *Mol Biol Evol* 2018;**35**(3):543-8.
34. Li, H. Minimap2: pairwise alignment for nucleotide sequences. *Bioinformatics* 2018;**34** (18):3094-100.
35. Kim, D, Paggi, J, Park, C, *et al*. Graph-based genome alignment and genotyping with HISAT2 and HISAT-genotype. *Nat Biotechnol* 2019;**37**(8):907-15.
36. Lin, Y, Ye, C, Li, X, *et al*. quarTeT: a telomere-to-telomere toolkit for gap-free genome assembly and centromeric repeat identification. *Hortic Res* 2023;**10**(8):uhad127.
37. Chen N. Using RepeatMasker to identify repetitive elements in genomic sequences. *Curr Protoc Bioinformatics* 2004;**Chapter 4**:Unit 4.10.
38. Xu, Z and Wang, H. LTR_FINDER: an efficient tool for the prediction of full-length LTR retrotransposons. *Nucleic Acids Res* 2007;**35**:W265-8.
39. Price, A, Jones, N and Pevzner, P. De novo identification of repeat families in large genomes. *Bioinformatics* 2005;**21**(Suppl 1):i351-8.
40. Benson, G. Tandem repeats finder: a program to analyze DNA sequences. *Nucleic Acids Res* 1999;**27**(2):573-80.
41. Kim, D, Langmead, B and Salzberg, S. HISAT: a fast spliced aligner with low memory requirements. *Nat Methods* 2015;**12**(4):357-60.
42. Kovaka, S, Zimin, A, Pertea, G, *et al*. Transcriptome assembly from long-read RNA-seq alignments with StringTie2. *Genome Biol* 2019;**20**(1):278.
43. Haas, B, Salzberg, S, Zhu W, *et al*. Automated eukaryotic gene structure annotation using EVidenceModeler and the Program to Assemble Spliced Alignments. *Genome Biol* 2008;**9**(1):R7.

44. Keilwagen, J, Hartung, F and Grau, J. GeMoMa: Homology-Based Gene Prediction Utilizing Intron Position Conservation and RNA-seq Data. *Methods Mol Biol* 2019;**1962**:161-77.
45. Stanke, M and Waack, S. Gene prediction with a hidden Markov model and a new intron submodel. *Bioinformatics* 2003;**19**(Suppl 2):ii215-25.
46. Bairoch, A and Apweiler, R. The SWISS-PROT protein sequence data bank and its supplement TrEMBL. *Nucleic Acids Res* 1997;**25**(1):31-6.
47. Kanehisa, M, Sato, Y, Kawashima, M, *et al*. KEGG as a reference resource for gene and protein annotation. *Nucleic Acids Res* 2016;**44**(D1):D457-62.
48. Jones, P, Binns, D, Chang, H, *et al*. InterProScan 5: genome-scale protein function classification. *Bioinformatics* 2014;**30**(9):1236-40.
49. Ashburner, M, Ball, CA, Blake, J, *et al*. Gene ontology: tool for the unification of biology. The Gene Ontology Consortium. *Nat Genet* 2000;**25**(1):25-9.
50. Marçais, G, Delcher, A, Phillippy, A, *et al*. MUMmer4: A fast and versatile genome alignment system. *PLoS Comput Biol* 2018;**14**(1):e1005944.
51. Goel, M, Sun, H, Jiao, W, *et al*. SyRI: finding genomic rearrangements and local sequence differences from whole-genome assemblies. *Genome Biol* 2019;**20**(1):277.
52. Wang, K, Li, M and Hakonarson, H. ANNOVAR: functional annotation of genetic variants from high-throughput sequencing data. *Nucleic Acids Res* 2010;**38**(16):e164.
53. Emms, D and Kelly, S. OrthoFinder: phylogenetic orthology inference for comparative genomics. *Genome Biol* 2019;**20**(1):238.
54. Nakamura, T, Yamada, K, Tomii, K, *et al*. Parallelization of MAFFT for large-scale multiple sequence alignments. *Bioinformatics* 2018;**34**(14):2490-2.
55. Guindon, S, Delsuc, F, Dufayard, J, *et al*. Estimating maximum likelihood phylogenies with PhyML. *Methods Mol Biol* 2009;**537**:113-37.
56. Yuan, Y, Zhang, Y, Zhang, P, *et al*. Comparative genomics provides insights into the aquatic adaptations of mammals. *Proc Natl Acad Sci U S A* 2021;**118**(37):e2106080118.
57. Yang, Z. PAML 4: phylogenetic analysis by maximum likelihood. *Mol Biol Evol* 2007;**24**(8):1586-91.
58. Altschul, S, Gish, W, Miller, W, *et al*. Basic local alignment search tool. *J Mol Biol* 1990;**215**(3):403-10.
59. Wang, Y, Tang, H, Debarry, J, *et al*. MCScanX: a toolkit for detection and evolutionary analysis of gene synteny and collinearity. *Nucleic Acids Res* 2012;**40**(7):e49.
60. Bie, T, Cristianini, N, Demuth, J, *et al*. CAFE: a computational tool for the study of gene family evolution. *Bioinformatics* 2006;**22**(10):1269-71.
61. Liao, Y, Smyth, G and Shi, W. featureCounts: an efficient general purpose program for assigning sequence reads to genomic features. *Bioinformatics* 2014;**30**(7):923-30.

Tables

Table 1. Genome assembly statistics of YFP and EFP~~Yangtze and East Asian finless porpoises~~

	~~Yangtze finless porpoise~~YFP v2.0	~~Yangtze finless porpoise~~YFP v1.0	~~East Asian finless porpoise~~EFP v2.0	~~East Asian finless porpoise~~EFP v1.0
Total size of assembled genome (Gb)	2.48	2.27	2.50	2.50
Contig N50 (Mb)	125.12	0.09	128.00	84.69
Contig N90 (Mb)	83.62	0.02	80.21	29.54
Number of contigs	23	~~47,942~~66,345	24	52
Scaffold N50 (Mb)	125.12	6.34	128.00	122.40
Scaffold N90 (Mb)	83.62	1.13	80.21	80.21
Scaffolds number	22	~~13,695~~13,698	23	23
Number of base chromosomes	22	22	23	23
Number of gap-free chromosomes	22	0	23	7
Number of gaps	0	52,647	0	28
Number of telomeres (pairs/single)	20/2	0/0	21/2	21/2
Number of estimated centromeres	22	0	23	23
TE size	42.54%	NA	42.80%	42.23%
GC content	41.70%	41.00%	41.70%	41.70%
BUSCO*~~-~~ (Genome)	~~C:~~95.2%	~~C:~~94.0%	~~C:~~95.3%	~~C:~~95.4%
Gene Number	23,139	18,479	23,101	22,814
New-found gene number	5,480	NA	1,453	NA
Functional proteins	96.21%	NA	96.20%	97.31%
BUSCO*~~-~~ (Protein)	~~C:~~97.5%	~~C:~~94.6%	~~C:~~97.6%	~~C:~~97.9%
Data source	This study(PRJNA915046)	GCF_003031525.2~~GCF_000442215~~	This study(PRJNA859258)	GCA_026225855.1

Note: “v2.0” indicated the new genome assembly and annotation generated in this study; “v1.0” indicated previously published first draft of genome assembly and annotation. “BUSCO*~~C~~” indicated the percentage of complete BUSCO evaluation. “pairs/single”: “pairs” indicated that the telomeres were found at both ends of the chromosomes; “single” indicated that the telomeres were found only at the one end of chromosomes. “New-found genes” indicated that the genes were predicted in the extra sequence segments from the current assembly and were annotated in the current assembly.

Figure legends

~~**Figure 1. Genome analysis and quality assessment of Yangtze and East Asian finless porpoises v2.0.**~~

~~A: Location distribution and sampling site of the Yangtze and East Asian finless porpoises.~~

~~B: **Synteny analysis of Yangtze finless porpoise and East Asian finless porpoise v2.0 genomes:** a) chromosomes length; b) frequency of genes; c) density of genes; d) repeat density; e) GC density and f) syntenic blocks between Yangtze and East Asian finless porpoises.~~

~~C: Heat map displaying Hi-C interactions of Yangtze finless porpoises v2.0.~~

~~D: Heat map displaying Hi-C interactions of East Asian finless porpoises v2.0.~~

~~E: BUSCO assessments exhibiting proportions classified as Complete and single-copy (S, blue), Complete and duplicated (D, green), Fragmented (F, yellow), and Missing (M, red) categories.~~

~~F: Proportions of genes that could be functionally annotated and transcriptionally detected in Yangtze and East Asian finless porpoises v2.0.~~

Figure 1. Sample site and Genome assessment of YFP v2.0 and EFP v2.0.

A: Location distribution and sampling site of the YFP and EFP.

B：Proportions of genes that could be functionally annotated and transcriptionally detected in YFP v2.0 and EFP v2.0.

~~**Figure 2. T2T-resolved assembly of Yangtze and East Asian finless porpoises v2.0.**~~

~~A: Structure of T2T and gap-free chromosomes in Yangtze finless porpoises v2.0. All 21+X chromosomes of Yangtze finless porpoises v2.0 are drawn to scale and the ruler indicates chromosome length. Triangles indicate the presence of telomere sequence repeats. Circles represent the locations of centromeric regions. The gap positions in the Yangtze finless porpoises v1.0 genome assembly are marked with squares corresponding to the right side of the chromosome in the Yangtze finless porpoises v2.0 genome assembly.~~

~~B: Structure of T2T and gap-free chromosomes in East Asian finless porpoises v2.0. All 21+X/Y chromosomes of East Asian finless porpoises v2.0 are drawn to scale and the ruler indicates chromosome length. Triangles indicate the presence of telomere sequence repeats. Circles represent the locations of centromeric regions. The gap positions in the East Asian finless porpoises v1.0~~

~~genome assembly are marked with squares corresponding to the right side of the chromosome in the East Asian finless porpoises v2.0 genome assembly.~~

Figure 2. T2T-resolved assembly of YPF v2.0 and EFP v2.0 and functional enrichment of genes in the centromere region.

Structure of T2T and gap-free chromosomes in A) YFP v2.0, and B) EFP v2.0. All 21+X/Y chromosomes are drawn to scale and the ruler indicates chromosome length. Triangles indicate the presence of telomere sequence repeats. Circles represent the locations of centromeric regions. The gap positions in the v1.0 genome assemblies are marked with squares to the right of the chromosome in the v2.0 genome assemblies.

Figure 3. Synteny analysis of YFP v2.0 and EFP v2.0 genomes: A) chromosomes scale, Unit length is Mb; B) syntenic blocks between YFP v2.0 and EFP v2.0.

~~**Figure 3. Expression patterns and functional enrichment of genes in the centromere region.**~~

~~A: Heatmaps of the gene expression levels in centromere region and non-centromere region of Yangtze finless porpoises v2.0.~~

~~B: Heatmaps of the gene expression levels in centromere region and non-centromere region of East Asian finless porpoises v2.0.~~

~~C: GO enrichment analysis of genes in centromere region of Yangtze finless porpoises v2.0.~~

~~D: GO enrichment analysis of genes in centromere region of East Asian finless porpoises v2.0.~~

Figure 4. Structure variant between YFP v2.0 and EFP~~Yangtze and East Asian finless porpoises~~ v2.0 genome assembly with YFP~~Yangtze finless porpoises~~ v2.0 genome assembly for reference.

~~A:~~ The density plot of A) SNPs and B) InDels between YFP v2.0 and EFP ~~Yangtze and East Asian finless porpoises~~ v2.0 genome assembly. The numerical values adjacent to the color legend indicate the count of SNPs/InDels per megabase (MB) window, where the gray legend representing zero. Each color corresponds to a specific range of values. For instance, in Figure 4A, the initial blue legend represents the range from 1 to 4440.

~~B: The density plot of Indels between Yangtze and East Asian finless porpoises v2.0 genome assembly.~~

~~C: KEGG enrichment analysis of genes located in SNP and Indel region of Yangtze finless porpoises v2.0 genome assembly. Blue bar charts indicate the genes located in Indel region; while red bar charts indicate the genes located in the SNP region.~~

~~D: GO enrichment of new-found genes of Yangtze finless porpoise v2.0.~~

~~E: GO enrichment of new-found genes of East Asian finless porpoise v2.0.~~

Figure 5. Genome evolution of YFP v2.0 and EFP v2.0~~Yangtze and East Asian finless porpoises~~.

A: Divergence time between YFP v2.0 and EFP v2.0~~Yangtze finless porpoises and East Asian finless porpoises~~, and number of expanded and contracted gene families. ~~green~~ Green and red numbers indicate gene family expansions and contractions, respectively. MRCA: Most Recent Common Ancestor. Ma: Million years ago.

B: A comparison of gene families associated with orthologs and paralogs in YFP v2.0 and EFP v2.0~~Yangtze finless porpoises and East Asian finless porpoises~~, and other 24 mammal species.

Figure 6. Functional Enrichment of genes.

~~C:~~ Significant A) KEGG and B) GO enrichment of expanded gene families in ~~Yangtze~~ YFP and ~~East Asian finless porpoise~~EFP lineage.

~~D:~~ KEGG enrichment analysis of positively selected genes in C) YFP~~Yangtze finless porpoises (Neaa)~~ and D) EFP~~East Asian finless porpoise (Neas)~~, respectively.

Response to reviewers Click here to access/download;Manuscript;Response to reviewers.docx

Dear Dr. Hans Zauner,

Thanks for your kind consideration and comments regarding to our manuscript entitled "Telomere-to-telomere gap-free genome assembly of the endangered Yangtze finless porpoise and East Asian finless porpoise" (GIGA-D-23-00359). We have revised our manuscript (details are provided below) in accordance with all comments and suggestions from the reviewers. We hope the revised manuscript have been improved and meet the standard requirement. We look forward to hearing from *GigaScience* soon.

Sincerely yours,

Correspondence: Kai Liu

Freshwater Fisheries Research Center, Chinese Academy of Fishery Sciences, Wuxi 214081, China.

E-mail: liuk@ffrc.cn

Response to reviewer 1's comments:

Reviewer #1: The authors have assembled new telomer-to-telomer reference genomes for two finless porpoise species, the fresh-water Yangtze finless porpoise and the oceanic East Asian finless porpoise. These genome assemblies appear to be ultra-high quality, with substantial description of the genomic features (repeats, genes, assembly characteristics), improvements over previous assemblies for the same species, and comparison of the genomes for some features including genomic structure, variation, novel genes and genes under selection. These represent substantial contributions to cetacean genomics and to understanding the evolution of these two sister species in their unique habitats.

The manuscript requires substantial revision to provide adequate methods for the reader to understand how the genomes were assembled and analyzed, and the figures need to be modified substantially to be useful. As they are, the legends do not adequately describe what's in many of the figures, the figures often try to present too much information in a single plot or set of plots, and the text is often too small to read. In my detailed comments below, I suggest ways to modify the figures and legends to make them more informative and useful.

The manuscript is generally well written, but requires some editing for standard English, and several sections repeat information presented in tables and figures, and could be edited to reduce length and focus on interpretation rather than just repetition. In particular, although the improvements in the new genome assemblies are substantial compared to previous assemblies, these improvements are a normal process in genome assembly, and not of great biological importance. I suggest reducing description and discussion of the upgrades to the genomes, and focus on the interesting characteristics of, and comparisons between, the two new genomes.

Response: Thanks for your valuable suggestions on our work. The comments are providing important guiding significance to our research. Based on the comments we received, detailed modifications are provided below. According to your advice, this manuscript was edited for proper English language, grammar, punctuation, spelling, and overall style by one native English speaker.

Specific comments (by line number)

Comment 1:28. change 'the chromosome both ends' to 'the chromosomes at both ends'

Response: We are sorry for the mistake. We have changed 'the chromosome both ends' to 'the chromosomes at both ends' in the revised manuscript.

Comment 2:31. 'selection pressure' was not studied, only inference of selection.

Response: Thank you very much for your suggestion. We have revised 'selection pressure' to 'inference of selection'.

Comment 3:33. The accession ID given for YFP, GCF000442215, is for Lipotes vexillifer, not for the YFP genome.

Response: We are sorry for the mistake. We have changed 'GCF000442215' to 'GCA_003031525.2'

Comment 4:43. is there a word missing between 'clearly' and 'dorsal fin? Or, change from 'a clearly dorsal fin' to 'an obvious dorsal fin'.

Response: Thank you very much for your suggestion. We have revised 'a clearly dorsal fin' to 'an obvious dorsal fin'.

Comment 5:54. It's not clear what you mean by 'populations', since the comparisons are among species and subspecies. The term 'population' means something different from species and subspecies, so this section should be revised to use the appropriate terms.

Response: Thank you very much for your suggestion. We have revised the sentence 'Yang *et al*. identified significant genetic structure between the Indo-Pacific finless porpoise and the other two populations…' to 'Yang *et al*. identified significant genetic structure between either the Yangtze River population or the Yellow Sea population and the South China Sea population…' in the revised manuscript.

Comment 6:67. Insert 'a' before 'high-quality genome'

Response: We have inserted 'a' before 'high-quality genome' in the revised manuscript.

Comment 7:69. change 'cetacean' to 'cetaceans'

Response: We have change 'cetacean' to 'cetaceans' in the revised manuscript.

Comment 8:70. delete 'which was'

Response: We have deleted 'which was' in the revised manuscript.

Comment 9:71. The number of scaffolds for the previous version of the genome is misleading. The number given, 104, is based only scaffolds that are larger than the N50 length. According to the supplemental materials for Zhou et al. 2018, there were actually 97,387 scaffolds >100bp, 2179 scaffolds >2kb, and 423 scaffolds larger than the N90 size. If not all scaffolds are going to be counted, then it's important to specify which scaffolds were counted.

Response: Thank you for bringing this to our attention. We have made revisions in the description of the initial genome assembly of YFP. As reported on the NCBI website (https://www.ncbi.nlm.nih.gov/datasets/genome/GCF_003031525.1/), the assembly comprised 13,698 scaffolds, with a scaffold N50 of 6.3 Mb, excluding the minimum sequence length (100 bp) consideration.

Comment 10:80. change to 'a hotspot of genomic research, with extensive applications…'

Response: We have changed '…a hotspot genomic research fields now, extensive applications…' to '…a hotspot of genomic research, demonstrating extensive applications…'

Comment 11:81. insert 'and' between 'chicken' and 'fish'.

Response: We have inserted 'and' between 'chicken' and 'fish'.

Comment 12:99. change 'Based on' to 'In addition to'

Response: We have changed 'Based on the previously sequenced 62x PacBio HiFi and 85x Hi-C reads of the EFP, we generated 215 Gb (86x) ONT reads in this study' to 'In this study, we supplemented the existing dataset comprising 62 × PacBio HiFi and 85 × Hi-C reads of the EFP with an additional 215 Gb (86 ×) of ONT reads'.

Comment 13:116. What do the Merqury quality values mean? Are these from a range (e.g., 1-100), or do they represent a log value? The reader can't interpret this without some context, and you can't assume they are familiar with every program.

Response: The quality values obtained from Merqury's k-mer analysis for YFP v2.0 and EFP v2.0 were calculated as 60.18 and 64.38, respectively. These values indicate a foundational accuracy level of 99.999%, confirming the high quality of our assembly for each component. It is essential to emphasize that Merqury quality values span from 0 to 255, with higher values denoting superior quality.

Ref:

Rhie, A, Walenz, B, Koren, S, *et al*. Merqury: reference-free quality, completeness, and phasing assessment for genome assemblies. *Genome Biol* 2020;**21**(1):245.

Comment 14:133-156. The whole section on gene prediction and annotation results could be reduced so that it repeats less of what is already presented in Table 1. Simple summary values such as average length of coding sequences don't need to be presented in the results, especially if they are not discussed in the discussion section as being important.

Response: Thank you for your suggestion concerning 'Gene prediction and annotation' section. We have revised the text with simple summary values at lines 163-172 in the revised MS to minimize redundancy and emphasize the main discoveries. The revised section now offers a succinct overview of the predicted protein-coding genes in the YFP and EFP genomes, supported by evidence and a comparison of length distributions. Supplementary Tables 9-12 contain further information, such as the average lengths of coding sequences, exons, and introns, as recommended.

We have changed 'In total, we predicted 23,139 and 23,101 protein-coding genes in the

YFP and EFP genomes, respectively (Table 1), where the average length of coding sequence (CDS) was 1,507 bp and 1,510 bp, respectively. The average length of exon was both 175 bp, and the average length of intron was 6,082 and 6,107 bp, respectively (Supplementary Table S11-S12). The protein-coding genes in the YFP and EFP genomes were supported by at least one evidence with a CDS overlap ratio greater than 80% at a level of 99.96% and 99.95%, respectively (Supplementary Table S13-S14). ' to 'In total, the number of predicted protein-coding genes was 23,139 in the YFP v2.0 genome and 23,101 in the EFP v2.0 genome (Table 1). The roughly comparable number of predicted protein-coding genes for both T2T genomes is further evidence supporting the gene models (Supplementary Table S9-S12).'

Comment 15:138. The sentence starting 'For gene content assessment…' should be in the methods, not results. It's also not clear what you mean by '8 homologous proteins' (proteins, or databases, or ???). Also, change 'RNAseq data' to 'RNAseq datasets' (as in line 156).

Response: We apologize for any inconvenience caused. What I meant to convey is: "Utilizing 149,956 genes from 8 closely related cetacean species and transcriptomic sequencing data from 24 Yangtze finless porpoises as input files, the GeMoMa software was employed to conduct homology-based prediction analysis."

Comment 16:143-145. The sentence starting with 'The protein-coding genes in…' is difficult to understand, and I'm not sure it's important. Does it belong in the methods, or does it say something about the quality of the gene identification?

Response: We have revised the text with simple summary values at lines 163-172 in the revised MS to minimize redundancy and emphasize the main discoveries.

We have changed 'In total, we predicted 23,139 and 23,101 protein-coding genes in the YFP and EFP genomes, respectively (Table 1), where the average length of coding sequence (CDS) was 1,507 bp and 1,510 bp, respectively. The average length of exon was both 175 bp, and the average length of intron was 6,082 and 6,107 bp, respectively (Supplementary Table S11-S12). The protein-coding genes in the YFP and EFP

genomes were supported by at least one evidence with a CDS overlap ratio greater than 80% at a level of 99.96% and 99.95%, respectively (Supplementary Table S13-S14). ' to 'In total, the number of predicted protein-coding genes was 23,139 in the YFP v2.0 genome and 23,101 in the EFP v2.0 genome (Table 1). The roughly comparable number of predicted protein-coding genes for both T2T genomes is further evidence supporting the gene models (Supplementary Table S9-S12).'

Comment 17:147. The wrong accession ID is given for the YFP, and I don't understand why the accession numbers are being presented here at all, since this whole section is about the new YFP and EFP genomes.

Response: We are sorry for the mistake. We have corrected the accession ID and changed the sentence to 'It is worth noting that the length distribution of gene models at the levels of genes, CDS, exons and introns showed a similar trend when compared to those of YFP v1.0 (GCA_003031525.2), EFP v1.0 (GCA_026225855.1) and Bottlenose Dolphin (GCF_011762595).'

Comment 18:155. 'Finally, 20, 589 (88.98%) and 20, 613 (89.23%)' of what? Genes? It's unclear what you are referring to for these numbers.

Response: We are sorry for the unclear description. We have changed 'Finally, 20,589 (88.98%) and 20,613 (89.23%) could be transcriptionally detected by the 24 RNA-seq datasets.' to 'Finally, 20,589 (88.98%) and 20,613 (89.23%) genes from the YFP v2.0 and EFP v2.0 genomes, respectively, were determined to be transcriptionally active based on the analysis of 24 RNA-seq datasets.'

Comment 19:162. What are 'repeat monomers'? is this a particular sequence that is unique to centromere repeats? One more sentence to describe what you are using to infer centromeres would be useful.

Response: We are sorry for the unclear description. We have added the description: 'In this study, we conducted predictions on repeat monomers within the YFP v2.0 and EFP v2.0 genomes, potentially constituting the centromere. The monomeric sequences vary

in length from 99 to 201 bp, with the 144-bp, 150-bp, and 138-bp monomers being the most abundant. Centromeres are composed of more than one repeat monomer and are located within TE- and TR-enriched regions, which are areas with relatively lower gene density.'

Comment 20:165. Does 'newly identified centromere regions' refer to regions that are newly discovered in centromeres, or newly discovered in the new genome assemblies (compared to the old ones)? The sentence is a little confusing.

Response: We are sorry for the unclear description. We have changed 'In total, 235 and 237 genes were identified in the YFP and EFP candidate centromere region, respectively, while 56 and 20 genes of YFP and EFP were discovered in the newly identified centromere regions.' to 'A total of 235 and 237 genes were identified in the candidate centromere regions for YFP v2.0 and EFP v2.0, respectively, through predictions generated by centromere-finding software. Moreover, the newly discovered centromere regions for YFP v2.0 hosted approximately 56 genes, while only 20 genes were found in analogous regions for EFP v2.0. The "newly identified centromere regions" refer to specific areas identified in the genome that have been recently assembled but were not included in the previously published version. This discovery may suggest the presence of novel, previously uncharacterized centromere sites.'

Comment 21:179-180. please explain what 'stop-gain SNPs' and 'stop-loss SNPs' are. I think this means SNPs that result in the gain or loss of a stop codon, but I don't know the specific term (jargon).

Response: I apologize for any inconvenience caused. In genetics, these terms mean:

'stop-gain SNPs' (nonsense): A mutation that results in the premature termination of protein synthesis by introducing a premature stop codon, leading to the production of a truncated and often non-functional protein.

'stop-loss SNPs': A mutation where a stop codon is lost, allowing the translation machinery to continue synthesizing the protein beyond its intended termination point.

Comment 22:183. Why did you identify these 5 pathways from Figure 4C? Were they the only ones with functional variants, or are they just random examples from all of the ones in Figure 4C?

Response: We are sorry for the unclear description. We have modified the description of these pathways: 'KEGG enrichment analysis revealed that these genes were significantly (P value < =0.05) enriched in “NF-kappa B signaling pathway”, “complement and coagulation cascades”, “antigen processing and presentation” and “Intestinal immune network for IgA production”'.

In addition, we have enriched the discussion on these pathways: "The genes coding for the mutated regions of the YFP and EFP are widely enriched in immune-related pathways. This association may be intricately linked to the distinct pathogenic microorganisms unique to freshwater and seawater environments. Marine mammals exhibit a diminished histocompatibility complex (MHC) diversity attributed to decreased encounters with microparasitic diversity in their marine habitat relative to their terrestrial origin. This phenomenon implies that mammals encounter distinct pathogenic pressures in varied ecological settings, potentially influencing the evolution of immune-related genes. [1]. Evolutionary analyses of the innate immune pattern recognition receptor (TLRs) in the YFP and the marine finless porpoise indicate that the YFP has undergone specific adaptive changes [2]. The microbial diversity and pathogenicity of freshwater and seawater environments vary, leading to distinct effects of pathogenic microorganisms on the organisms in these two types of environments [3]. Therefore, the YFP and EFP would be expected to undergo adaptive evolution to adapt to the pathogen stresses specific to their respective ecological environments, freshwater and seawater."

Ref

[1] Slade, R and McCallum, H. Overdominant vs. frequency-dependent selection at MHC loci. *Genetics* 1992;**132**(3):861–64.

[2] Tian, R, Chen, M, Chai, S, *et al*. Divergent Selection of Pattern Recognition Receptors in Mammals with Different Ecological Characteristics. *J Mol Evol* 2018;**86**(2):138-49.

[3] Lokesh, J and Kiron, V. Transition from freshwater to seawater reshapes the skin-associated

microbiota of Atlantic salmon. *Sci Rep* 2016;**25**(6):19707.

Comment 23:186. Delete 'Here', and start the sentence with 'The genes coding…'

Response: Thank you very much for your suggestion. We have deleted 'Here' in the revised manuscript.

Comment 24:190. change 'will' to 'would be expected to'

Response: Thank you very much for your suggestion. We have changed 'will' to 'would be expected to'.

Comment 25:192-193. Delete the last sentence of the paragraph, as it is just repeating what is said above.

Response: Thank you very much for your suggestion. We have deleted the repeated sentence.

Comment 26:194. Delete 'Specifically,' and start the sentence with 'By comparing…' .

Response: Thank you very much for your suggestion. We have deleted 'Specifically,' and start the sentence with 'By comparing…' in the revised manuscript.

Comment 27:195. change 'YFP assembled 5480 new genes, while EFP assembled 1453…' to 'The YFP assembly included 5480 new genes, while the EFP assembly included 1453…'

Response: Thank you very much for your suggestion. We have corrected it in the revised manuscript.

Comment 28:198. Change 'discovered' to 'indicated'

Response: Thank you very much for your suggestion. We have changed 'discovered' to 'indicated' in the revised manuscript.

Comment 29:203. Change 'including' to 'include'

Response: Thank you very much for your suggestion. We have changed 'including' to 'include' in the revised manuscript.

Comment 30:209. Insert 'structural' or 'chromosomal' before 'conservation'

Response: Thank you very much for your suggestion. We have changed 'Synteny analysis demonstrated that YFP displayed a greater level of <u>conservation</u> than EFP' to 'Synteny analysis of the gene order between YFP v2.0 and EFP v2.0 revealed 907 large shared syntenic blocks, encompassing 89.59% (41428) genes, and 17 chromosomal rearrangements' in the revised manuscript.

Comment 31:209-214. Figure 1B should be its own figure, and changed to include only the synteny information. It's too small and too dense to be useful as it is. The full figure can be presented in supplemental materials. The text describing the figure could be expanded to describe what was found rather than just general observations of 'similar patterns' gene frequency, density, etc., or it could be reduced to just focus on what was different (specific chromosome rearrangements).

Response: Thank you for the valuable suggestions provided. We have separated subfigures A and F from Figure 1B into individual new Figure 3. The remaining subfigures have been consolidated to create a new figure, now referred to as Supplemental Figure S7.

Comment 32:218. What do you mean by the 'last common ancestor'? This 'ancestor' doesn't exist, so is it a reconstructed ancestral genome?

Response: We apologize for the error in the description. We have changed 'When comparing the genomes of YFP and EFP with their <u>last common ancestor</u>, it was found that 843 gene families expanded while 98 contracted. 'to 'Upon comparing the genomes of YFP v2.0 and EFP v2.0 with their <u>most recent</u> common ancestor, it was observed that 843 gene families underwent expansion, while 98 gene families experienced contraction.'

When studying the evolution of gene families, the root of the phylogenetic tree is

typically described using the term "Most Recent Common Ancestor" (MRCA), indicating the point of origin for these gene families during the evolutionary process, where they initially diversified into different genes. This term emphasizes the shared origin among different members of the gene family and their relationship to the common ancestor.

Comment 33:220. What does 'involved with statistical significance' mean?

Response: We are sorry for the unclear description. We have changed 'Out of the 215 expanded gene families identified in the YFP and EFP lineage, a total of 2,674 genes were found to be involved with statistical significance ($P<0.05$)' to 'Among the 215 expanded gene families identified in the YFP v2.0 and EFP v2.0 lineage, a total of 2,674 genes were determined to be significantly associated ($P<0.05$)'.

Comment 34:232-234. The first time these species are mentioned, the Latin name needs to be included. In the methods, only the Latin names are used, so the reader can't easily know what species the common names refer to (e.g. the Chinese white dolphin is called the Indo-Pacific humpback dolphin in the Society for Marine Mammalogy's taxonomy list).

Response: We are sorry for the unclear description. We have supplemented the Latin names in the sentence '*Neophocaena asiaeorientalis asiaeorientalis* (Yangtze finless porpoise), *Neophocaena asiaeorientalis sunameri* (East Asian finless porpoise), *Tursiops truncatus* (Bottlenose dolphin), *Orcinus orca* (Killer whale), *Lipotes vexillifer* (Yangtze River dolphin), *Physeter catodon* (Sperm whale), *Balaenoptera acutorostrata* (Minke whale), *Balaena mysticetus* (Bowhead whale), *Delphinapterus leucas* (Beluga whale), *Sousa chinensis* (Indo-Pacific humpback dolphin)'.

In addition, we have added the common names in the methods '(a) *Ornithorhynchus anatinus* (Platypus) and *Monodelphis domestica* (Opossum) (163.7–185.9 Ma), (b) *Homo sapiens* (Human) and *Mus musculus* (Mouse) (81.3–91.0 MYA), (c) *Balaena mysticetus* and *Balaenoptera acutorostrata* (21.3–28.8 Ma) and (d) *Sousa chinensis* and *Tursiops truncatus* (2.0–3.8 Ma).'

Comment 35:237-239. What evidence is there for the statement that 'The evolution of DNA damage repair pathways implied the existence of additional triggers for genomic instability in the Yangtze River, including human activities such as wading projects, dredging and quarrying'? It seems odd to invoke pressure from the last century or less for evolutionary changes at the gene pathway level.

Response: We are sorry for the mistake. We have revised and expanded upon this paragraph: ' The selective pressure to evolve DNA damage repair pathways implied that the Yangtze finless porpoise might be experiencing increased threats to genome stability. The mechanism of DNA damage repair plays a crucial role in preserving genome integrity by enabling cells to identify and repair DNA damage, thereby averting the accumulation of harmful mutations [1]. In a comparative genomic analysis between the South China tiger and the Amur tiger, it was noted that genes related to DNA repair underwent positive selection in the South China tiger [2]. The observed phenomenon could be explained by the higher probability of genome instability in the temperate and subtropical habitats of the South China tiger. This may be linked to metabolites generated by intestinal microflora, which possess the ability to trigger DNA damage [3]. The stability of the genome or gene expression system in the Yangtze finless porpoise across different organs and life stages remains uncertain. However, one potential interpretation of this data is the suggestion that the Yangtze finless porpoise could be vulnerable to genomic instability triggers in the Yangtze River, such as water pollutants, which may increase the likelihood of DNA damage [4]. Pollutants found in the Yangtze River possess the capacity to accumulate within the food chain, leading to cellular DNA damage and impacting the genome stability of the Yangtze finless porpoise [5-6]. This makes a compelling case for the improved conservation of the species and the development of more rigorous water pollution mitigation practices.'

Ref

[1] Chatterjee, N and Walker, G. Mechanisms of DNA damage, repair, and mutagenesis. *Environ Mol Mutagen* 2017;**58**(5):235-63.

[2] Zhang, L, Lan, T, Lin, C, *et al*. Chromosome-scale genomes reveal genomic consequences of inbreeding in the South China tiger: A comparative study with the Amur tiger. *Mol Ecol Resour*

2023;**23**(2):330-47.
[3] Puschhof, J and Sears, C. Microbial metabolites damage DNA. *Science* 2022;**378**(6618):358-59.
[4] Lv, W, Gu, H, He, D, *et al*. Polystyrene nanospheres-induced hepatotoxicity in swamp eel (*Monopterus albus*): From biochemical, pathological and transcriptomic perspectives. *Sci Total Environ* 2023;**893**:164844.
[5] Zhang, K, Qian, Z, Ruan, Y, *et al*. First evaluation of legacy persistent organic pollutant contamination status of stranded Yangtze finless porpoises along the Yangtze River Basin, China. *Sci Total Environ* 2020;**710**:136446.
[6] Xiong, X, Qian, Z, Mei, Z, *et al*. Trace elements accumulation in the Yangtze finless porpoise (*Neophocaena asiaeorientalis asiaeorientalis*)-A threat to the endangered freshwater cetacean. *Sci Total Environ* 2019;**686**:797-804.

Comment 36:256-257. The 'QIAGEN Blood & Cell Culture DNA Midi Kit' appears to be a DNA extraction kit, not a library preparation kit, so it cannot have been used to construct PacBio HiFi libraries. This section needs more detail to specify exactly what kits or methods were used to construct libraries, as the library methods impact downstream DNA sequence processing.

Response: Thanks for the suggestion. We have modified and enriched the methods: 'The PacBio HiFi library was constructed using SMRTbell Prep Kit 3.0 (Pacific Biosciences, USA) and subsequently sequenced on the PacBio Sequel II system in circular consensus sequence (CCS) mode. To collect data for the Hi-C library, the muscle tissues were first fixed in 1% formaldehyde (Sigma) for cross-linking and resuspended in lysis buffer. Then, MboI (NEB) restriction endonucleases were used to fragment the chromatin in the muscle to fragment DNA. The DNA fragments were captured by utilizing Streptavidin-coated magnetic beads (Thermo Fisher SCIENTIFIC) following biotin labeling and crosslinking using T4 DNA Ligase (ENZYMATICS). The Hi-C library was finally sequenced on a BGI MGISEQ platform.'

Comment 37:261. Cite a reference for the 'CTAB method'

Response: Thanks for bringing this to our attention. We have cited a reference for the 'CTAB method'.

Ref:

Yan, M, Wei, G, Pan, X *et al*. A method suitable for extracting genomic DNA from animal and

plant-modified CTAB method. *Agric Sci Technol*, 2008, **9**(2):39-41.

Comment 38:267. What filters were used to 'filter the Hi-C reads? Are there specific quality filters, or read trimming that are needed?

Response: Thank you for drawing our attention to this. To filter Hi-C reads, index reads were removed and reads were filtered using the following SOAPNUKE v2.0 parameters: N rate >= 0.01, low quality <= 20, low quality rate >= 0.1. Correspondingly, we have amended the relevant descriptions in the revised MS at line 406-408.

Comment 39:268. What do you mean by 'refine' the ONT reads? This is a meaningless term, and since the Perl scripts are described as 'self-designed', this provides no information about what was done. Likewise, the statement that 'ONT reads were subsequently corrected' is also too vague. What was actually done to 'correct' the reads?

Response: Thank you for your valuable suggestions. ONT reads were filtered based on a length < 5 kb and a quality value < 7. The Necat pipeline (v 20200119) was utilized for the enhancement of ONT reads. This was achieved through the application of error correction algorithms to evaluate quality scores, k-mer frequencies, and alignment methods. This process enhanced the accuracy of reading and generated refined results suitable for further analyses. Correspondingly, we have amended the relevant descriptions in the revised MS at line 408-414.

Comment 40:272. There are a lot of programs used to process data, and while it's good that the actual parameters are given for some of them, it would be useful to also state whether the parameters were the default parameters or modified for these data, and describe what the parameter do (e.g., what does '-j 80 -s 80 -a 30' do?)

Response: Thank you for your valuable suggestions. The Purge-Haplotigs program was utilized to eliminate redundant contigs that exhibited similar sequences but distinct haplotypes, specifically targeting those with aligned coverage below 30%. This strategic approach significantly optimizes the assembly process by removing redundant information, thus improving the accuracy of genome assembly. Correspondingly, we

have amended the relevant descriptions in the revised MS at line 416-420.

Comment 41:275. What do you mean by 'bridge the gaps in the gapless PacBio assembly? Do you mean that ONT reads and contigs were used to bridge gaps in the contig assembly in order to generate the gapless scaffolds?

Response: We are sorry for the unclear description. We have changed the sentence to ' Ultra-long ONT reads and contigs were used to generate gapless scaffolds through the LR_Gapcloser (v1.0) and TGSgapcloser (v 1.0.1) pipelines.'

Comment 42:281. Change 'were' to 'was'

Response: We have changed 'were' to 'was' in the revised manuscript.

Comment 43:283-4. insert 'the' before 'quartet pipeline' and change 'region' to 'regions'.

Response: We have inserted 'the' before 'quartet pipeline' and changed 'region' to 'regions'.

Comment 44:287. Change 'conducted' to 'used'

Response: We have changed '…RepeatModeler (v1.0.4) and LTR-FINDER (v1.0.7) were conducted to identify repetitive elements and annotate long terminal repeats, respectively.' to '…RepeatModeler (v1.0.4) was employed to identify repetitive elements, whereas LTR-FINDER (v1.0.7) was used for the specific annotation of long terminal repeats.' in the revised manuscript.

Comment 45:294. If RNAseq data were mapped to both assemblies, then change 'assembly' to 'assemblies'.

Response: We have changed 'assembly' to 'assemblies' in the revised manuscript.

Comment 46:299. I think 'combing' should be changed to 'combining'

Response: We have changed 'combing' to 'combining'.

Comment 47:320. Change 'Consistent phylogeny with previous study' to 'A phylogeny consistent with a previous study'

Response: We have changed 'Consistent phylogeny with previous study' to 'A phylogeny consistent with a previous study'.

Comment 48:327. Change 'Blast' to 'BLAST'

Response: We have changed 'Blast' to 'BLAST'.

Comment 49:328. It's unclear what you mean by 'defined syntenic blocks by MCscanX'. Do mean that 'syntenic blocks were defined using MCscanX'?

Response: We have changed 'defined syntenic blocks by MCscanX' to 'syntenic blocks were defined using MCscanX'

Comment 50:332. delete 'branch of', or change to 'analysis in the finless porpoise clade'.

Response: We have changed 'analysis in branch of finless porpoise' to 'analysis in the finless porpoise clade'

Comment 51:336. should the meaning of P-value≤0.01 be 'significantly expanded and contracted gene families', or is it either expanded OR contracted 'significantly expanded or contracted gene families'?

Response: We have changed 'significantly expanded and contracted gene families' to 'significantly expanded or contracted gene families'.

Comment 52:338. Change 'family' to 'families'

Response: We have changed 'family' to 'families'.

Comment 53:339. Change the section title to 'Gene positive selection analysis'

Response: We have changed the section title to 'Gene positive selection analysis'.

Comment 54:343. What filtering of alignments was done with Gblocks?

Response: Thank you for drawing our attention to this. Alignment refinement via Gblocks was utilized to remove inadequately aligned positions and divergent regions, thereby isolating conserved blocks from the multiple alignment. Correspondingly, we have amended the relevant descriptions in the revised MS at line 528-530.

Comment 55:345. delete the comma after 'branch'

Response: We have deleted the comma after 'branch'.

Comment 56:350. 'quality controlled' is vague. What was done to the reads to make them 'clean'? Please include the parameters or steps, e.g., reads were trimmed to remove adapter sequences and base calls with quality <30 (or whatever your parameters were).

Response: Thank you for your valuable suggestions. The raw RNA-seq reads underwent quality control using SOAPnuke (v2.0). Reads were filtered out if they had an N rate ≥ 0.01, low quality ≤ 20, low quality rate ≥ 0.1, or contained index sequences. Subsequently, the clean reads were aligned to the EFS v2.0 genome utilizing the Hisat2 high sensitivity model, excluding discordant pairs and mixed alignments. The alignment process involved setting a minimum insert size of 1 bp and a maximum insert size of 1000 bp. Correspondingly, we have amended the relevant descriptions in the manuscript at line 539-543.

Comment 57:357. change 'assembly' to 'assemblies'

Response: We have change 'assembly' to 'assemblies'.

Comment 58:359. delete 'two' (not needed because you name the species genomes in the same sentence)

Response: We have deleted 'two'.

Comment 69:372. What is 'undesired recombination'? Undesired by whom? I think you can just delete that word, and say the genome data can serve as a valuable resources for understanding… recombination, etc.

Response: We have deleted 'undesired '.

Comment 60:373. change 'Overall, this is the most continuous genome assembly to date' to 'Overall, these are the most continuous cetacean genome assemblies to date'

Response: We have changed 'Overall, this is the most continuous genome assembly to date' to 'Overall, these are the most continuous cetacean genome assemblies to date'.

Comment 61:375. change 'deepen the scientific issues' to 'deepen the scientific understanding of issues'

Response: We have changed 'deepen the scientific issues' to 'deepen the scientific understanding of issues'.

Comment 62:387. Authors' contributions: The initials vary between 2 and 3 initials to represent authors' names, but the names in the authors list are all only two names, so there should be only 2 initials for each.

Response: We have corrected the initials of each name to 2. ' K.L., J.J. and P.X. designed and conceived the study. D.Y., C.Y. and J.Z. collected and prepared the samples. C.C. and C.Z. performed the data analysis. D.Y., C.C. and J.J. wrote the manuscript with significant contributions from Y.L., Z.C., H.Z., C.W. and L.L. K.L., Z.H. and D.L. provided the financial support. All authors read and approved the final version of the manuscript.'

Comment 63: Table 1. In the legend, provide the accession ID's for each genome version in the note below the table.

It's not clear why there is "C:" in front of each percentage for the two BUSCO lines. I think it would be clearer to remove "C:" from each number, put an asterisk (*) after BUSCO, and in the note, write "* percentage of complete BUSCO evaluation".

Response: Thank you for your valuable suggestions. We have provided the accession IDs for each genome except for the finless porpoise v2.0 genome assembly, as it is under control by NCBI but not yet public. Additionally, we have replaced "C:" with

"BUSCO*" in the legend of Table 1, and mentioned "BUSCO*" as the percentage of complete BUSCO evaluation.

Figures.

Comment 64: Figure 1 contains too many unrelated figures, and too dense information, some of which is never discussed in the paper. I suggest that Figure 1 should include only A and F, B should be its own figure, and C,D,E should be moved to supplemental materials.

For the new figure (B), only the rings A (chromosome length) and F (syntenic blocks) should be included, as the others are too tiny and dense to be useful to the reader. Those can be put into supplemental materials with better descriptions to allow the reader to see the detail and to explain the colors and scale. All colors within the figures need to be explained or include a color legend.

Response: Thank you very much for your suggestion. We have separated subfigures A and F from Figure 1B into individual new Figure 3. The remaining subfigures have been consolidated to create a new figure, now referred to as Supplemental Figure S7.

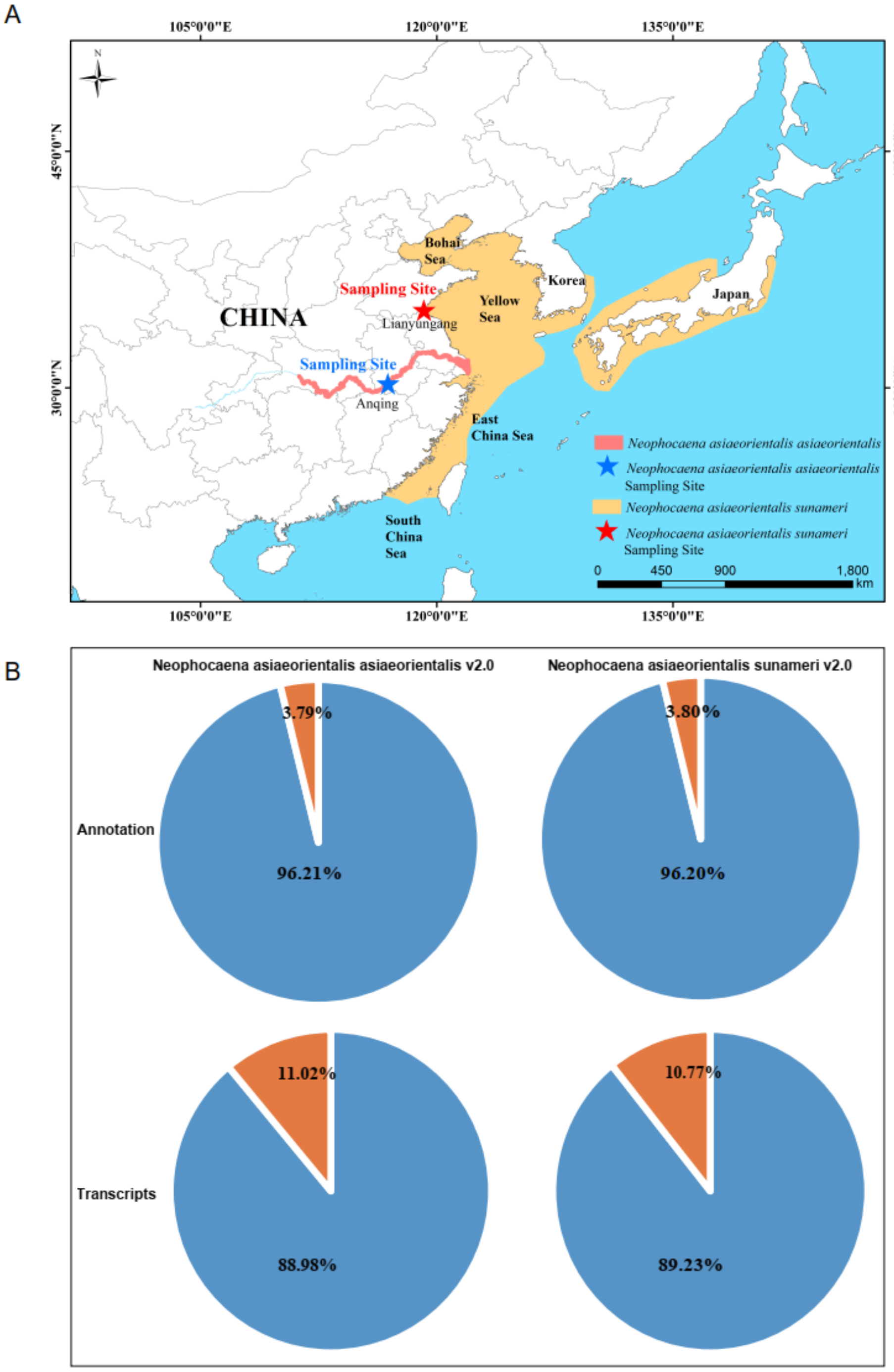

Figure 1. Sample site and Genome assessment of YFP v2.0 and EFP v2.0.

A: Location distribution and sampling site of the YFP and EFP.

B：Proportions of genes that could be functionally annotated and transcriptionally detected in YFP v2.0 and EFP v2.0.

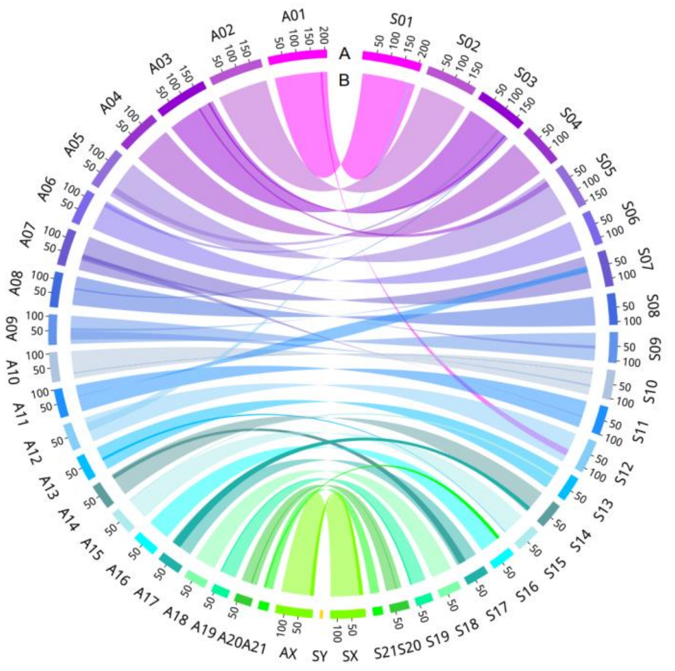

Figure 3. **Synteny analysis of YFP v2.0 and EFP v2.0 genomes**: A) chromosomes scale, Unit length is Mb; B) syntenic blocks between YFP v2.0 and EFP v2.0.

Comment 65: Figure 2. There is no visible "ruler" in the figure (but described in the legend).

The legends for A and B repeat most of the information. They can be combined to reduce repetition, e.g.,

Structure of T2T and gap-free chromosomes in A) Yangtze finless porpoises v2.0, and B) East Asian finless porpoises (v2.0). All 21+Xchromosomes are drawn to scale and the ruler indicates chromosome length. Triangles indicate the presence of telomere sequence repeats. Circles represent the locations of centromeric regions. The gap positions in the v1.0 genome assemblies are marked with squares to the right of the chromosome in the v2.0 genome assemblies.

Response: We have added "ruler" to the figure and combined the legends of A and B according to your helpful suggestion: 'Structure of T2T and gap-free chromosomes in A) YFP v2.0, and B) EFP v2.0. All 21+X/Y chromosomes are drawn to scale and the ruler indicates chromosome length. Triangles indicate the presence of telomere sequence repeats. Circles represent the locations of centromeric regions. The gap

positions in the v1.0 genome assemblies are marked with squares to the right of the chromosome in the v2.0 genome assemblies'.

Comment 66: Figure 3. A and B are described as 'Heatmaps', but they appear to be box plots, not heatmaps. They are only described in one sentence in the text, so I suggest that they could be moved to supplemental materials, and include only C and D in the figure.

In C and D, the legend says "GO enrichment analysis of genes", but the figure is not showing analysis, but results. Please describe the plot and results, e.g., number of genes in centromere regions associated with GO pathways" (that may not be correct, but I'm not sure what the figure is showing, so it needs to be described better).

Figure 4 D and E could be added to Figure 3 to show all of the same types of plots in one figure instead of splitting data into different figures.

Response: Thank you for your suggestion. Inspired by your guidance, We have made the following adjustments to Figure 3：

A. Figures 3A and 3B have been relocated to the supplemental materials and re-titled as follows: Supplementary Figure S8 Box plots illustrating the gene expression levels in the centromere region and non-centromere region of YFP v2.0 and EFP v2.0. Corresponding gene expression data are presented in Supplementary Tables S16 and S17.

B. We sincerely apologize for the previous error in presenting the enrichment analysis in Figures 3C and 3D. In the revised manuscript, we have corrected this and now display the revised enrichment analysis results in the supplemental materials as: Supplementary Figure S9 KEGG enrichment of centromere genes of YFP v2.0 and EFP v2.0. Additionally, the KEGG enrichment analysis are presented in Supplementary Tables S19 and S20.

C. We extend our sincere apologies for the earlier error regarding the presentation of the enrichment analysis in Figure 4D and 4E. In the revised manuscript, we have made the necessary corrections by removing Figure 4D and 4E. The updated enrichment analysis results are now appropriately presented in the supplemental material,

specifically in Supplementary Tables S26 to S29.

Comment 67: Figure 4.

A. what do the numbers next to the color legend represent? Is that the maximum number of SNPs per MB window? Is each color a range of values?

C. I don't understand what you mean by genes located in the Indel region or in the SNP region. What are the Indel and SNP 'regions'? These terms have not been used anywhere else or defined.

D-E. Consider moving to Figure 3. Are these 'new-found' genes relative to the old (V1) versions of the genomes, or genes that are unique to each species?

Response: I apologize for any inconvenience caused.

A. To improve the clarity of the presentation, we have made the following revisions to Figure 4 in the revised manuscript: the new Figure 4 now only retains sub-figures A and B; sub-figures C-E have been removed and replaced with the results of the KEGG enrichment analysis, which are now presented in Supplementary Figure S10 and Table S22.

B. The numerical values adjacent to the color legend indicate the count of SNPs/InDels per megabase (MB) window, where the gray legend representing zero. Each color corresponds to a specific range of values. For instance, in Figure 4A, the initial blue legend represents the range from 1 to 4440.

C. To address your inquiries regarding the terms "genes located in the Indel region" and "genes located in the SNP region," allow me to provide clarification:

Following the detection of SNPs (single nucleotide polymorphisms) and InDels (insertions or deletions), we employed Annovar for functional annotation. This process evaluates how these genetic variations might impact gene structure. The phrases in question describe the spatial relationship between genes and the aforementioned genetic alterations:

Indel Region: An Indel refers to a segment of DNA that has been inserted or deleted within the genome. When we mention a gene located in an Indel region, it indicates that the gene is situated at the site of such an insertion or deletion event.

SNP Region: A SNP represents a variation involving a single nucleotide within the genome. If a gene is described as being in a SNP region, it suggests that the gene contains one or more SNPs.

In summary, these terms denote the proximity of genes to genetic variations that may have functional implications.

D. The 'new-found' genes mentioned in this manuscript are relative to the old (V1) versions of the genomes, and the associated gene functional enrichment results are presented in Supplementary Tables S26 to S29.

Ref:

Wang, K, Li, M and Hakonarson, H. ANNOVAR: functional annotation of genetic variants from high-throughput sequencing data. *Nucleic Acids Res* 2010;**38**(16):e164.

Comment 68: Figure 5.

A. "Ma: Million years ago" can be deleted, as "Ma" is not used in the figure.

B. what are the units for the scale? Is this a count of gene families?

C-D. define what "Rich Factor" means. What do the colors mean?

E. There was no description of E, and it is too small to read. I suggest moving it to supplemental materials to allow it to be described in detail and large enough for the reader to see if interested.

Response: We have retained A and B of Figure 5 and treated C, D, E as a separate Figure 6.

A."Ma" is used in the Figure 5A.

B. The units of the scale are "millions of years ago," which are used to represent the divergence times between species.

C-D. In the context of KEGG enrichment analysis, the "Rich Factor" is a measure that represents the ratio of the number of candidate genes annotated to a specific pathway to the total number of genes annotated to that pathway. It is an indicator of how significantly the differentially expressed genes are enriched within a particular KEGG pathway compared to the whole genome background. A higher Rich Factor implies a greater degree of enrichment, suggesting that the pathway is more relevant to the

biological changes observed in the study.

The colors in a KEGG enrichment plot typically represent the significance of the enrichment. They are often associated with the q-value, which is a corrected p-value that adjusts for multiple hypothesis testing. The color intensity or shade—commonly ranging from blue to red—indicates the level of significance, with darker shades or redder colors signifying more significant enrichment (lower q-values). This visual representation helps researchers quickly identify pathways that are potentially important in the biological context of their study.

To summarize, the "Rich Factor" is a measure of enrichment significance, and the colors in a KEGG enrichment plot are a visual representation of the statistical significance of the enrichment, with redder colors indicating more significant enrichment.

E. To present the information more clearly, we have replaced Figure 5E with Figure 6C and 6D.

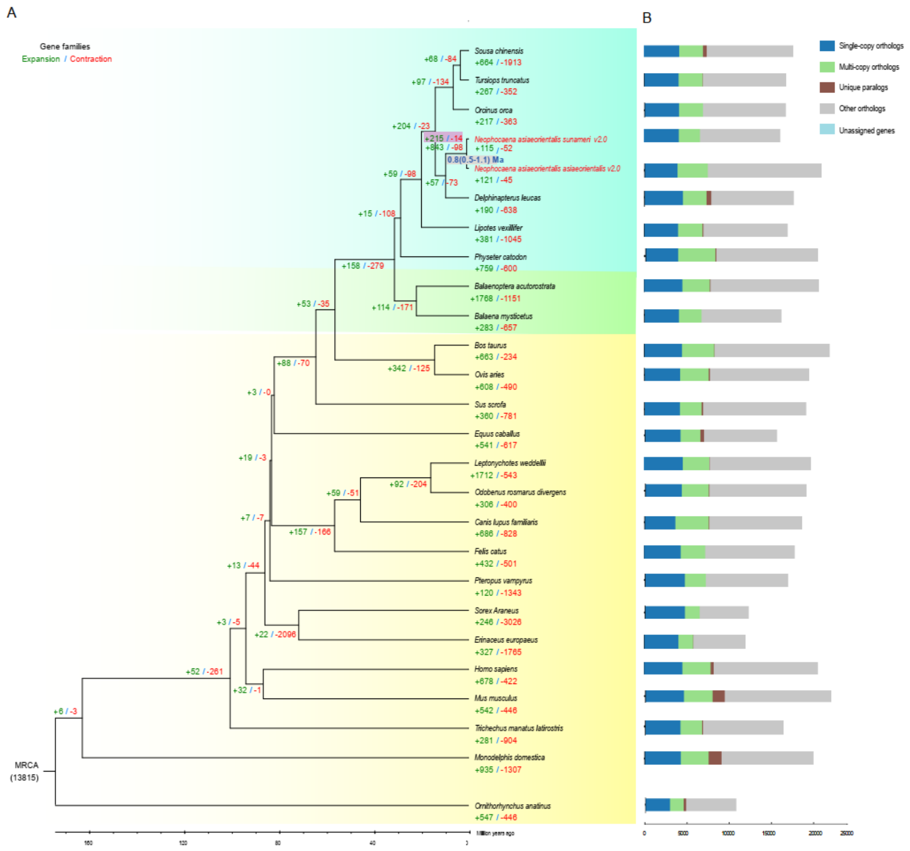

Figure 5. Genome evolution of YFP v2.0 and EFP v2.0.

A: Divergence time between YFP v2.0 and EFP v2.0, and number of expanded and contracted gene families. green and red numbers indicate gene family expansions and contractions, respectively.

MRCA: Most Recent Common Ancestor. Ma: Million years ago.

B: A comparison of gene families associated with orthologs and paralogs in YFP v2.0 and EFP v2.0, and other 24 mammal species.

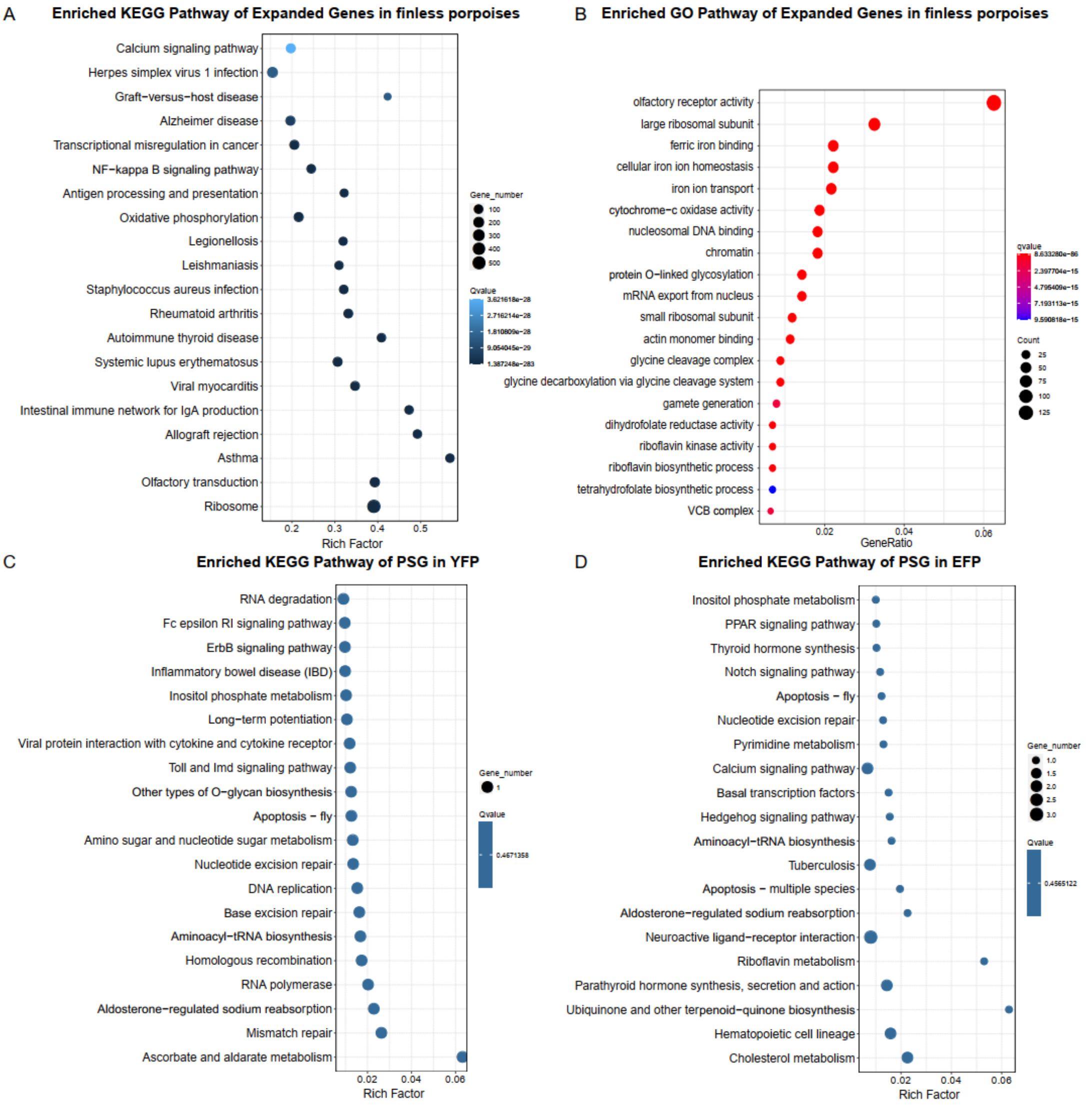

Figure 6. Functional Enrichment of genes.

Significant A) KEGG and B) GO enrichment of expanded gene families in YFP and EFP lineage. KEGG enrichment analysis of positively selected genes in C) YFP and D) EFP, respectively.

Reviewer #2: In this work, Yin *et al*. present two newly assembled genomes of two narrow-ridged finless porpoise (*Neophocaena asiaeorientalis*) subspecies (Yangtze, YFP and East Asia, EFP) as well as a comprehensive report on its quality and differences in terms of genotype changes related to their different habitats. I overall enjoyed reading this manuscript, I did not find too many downfalls in their methods, and I think these two genomes are of the highest quality and one of if not the most continuous reference genomes for cetaceans published thus far. Apart of this, however, I found several downfalls of the manuscript text itself which leads me to conclude that a major revision is needed for this manuscript to be considered for publication in GigaScience. I will first talk about general issues, point out the few downfalls within their methods and then provide a more detailed list of text issues which may help the authors to revise the manuscript.

Response: Thank you very much for your comments on our manuscript. The comments are all valuable and very helpful for revising and improving our paper. Based on the comments we received, detailed modifications are provided below.

General issues:

Comment 1: The text of the manuscript has multiple grammatical errors and I invite the authors to revise the language of the manuscript critically and carefully. I will list the errors I found down below but will not guarantee that I found everything. Furthermore, many statements made by the authors come without a proper citation which I will also highlight down below.

Response: We value your correction of language and citation errors. In the revised version, we have double-checked the paper for grammar, spelling, formatting, and citation issues and corrected them.

Comment 2: The "Results and Discussion" part is missing a discussion. The authors invest the vast majority of this part describing their results and do not attempt to contextualize their results with recent literature. For example, the authors could have compared the quality of these reference genomes and annotations to other cetacean

genomes to emphasize their superior quality. Furthermore, the authors did find, through various analyses, genes that might have played a role in their adaptions to different habitats but do not describe what is known about these genes and gene categories and do not describe how these gene might have help in their adaption. Also, the few sentences that do mention these possibilities come without proper citations, again.

Response: Thank you very much for your suggestion. We have added detailed description about genes with proper citations, which can be found in comment 23,29,30 and 31.

Comment 3: The material and method section is missing details. I assumed to find them in some sort of supplementary methods or data repository but failed to find them. If these details are indeed missing, I strongly suggest to provide more details in one of both ways (supplementary methods or within a data repository). I'll list the missing parts down below. Furthermore, the data repository made by the authors on figshare does lack a proper commentary README file and interested scientists are left to figure out the content of these files by themselves. I would strongly recommend providing an extensive README file for this repository to assure reproducibility.

Response: We have made revisions based on your suggestions provided. An enhanced version of the documentation file has been revised and is now accessible on the Figshare database. The documentation file contains the following contents:

Submitting organization: Freshwater Fisheries Research Center, Chinese Academy of Fishery Sciences
Department: Key Laboratory of Freshwater Fisheries and Germplasm Resources Utilization
Street: Binhu
City: Wuxi
Postal code: 214081
Country: China

1)Neaa v1 :
BioProject ID: PRJNA915046
BioSample ID: SAMN32369490
Assembly methods: Hifiasm 0.16.1-r375, LR_gapcloser, TGS_gapclosr, 3d_dna
Genome coverage: 49.0X
Sequencing technologies: PacBio Sequell, Oxford Nanopore PromethION, DNBSEQ (Hi-C)
Organism name : Neophocaena asiaeorientalis ssp. asiaeorientalis
Sex : Female
Date: 2023-02-24
Files:

Neaa.Chrplus.Genome.v1.fasta.gz : Genome assembly in FASTA format, contains the nucleotide composition information for each chromosome.
Neaa.Chrplus.Gene.v1.cds.gz : CDS file (Coding Sequence File), contains the coding sequences (CDS) in the form of DNA sequences, typically describing the coding regions of genes.
Neaa.Chrplus.Gene.v1.gff.gz : GFF file (General Feature Format), a text file format used to describe genomic features such as genes, exons, promoters, UTRs, etc. It is commonly used for storing genome annotation information.
Neaa.Chrplus.Gene.v1.pep.gz : PEP file (Protein Sequence File), contains protein sequences in the form of amino acid sequences, typically describing the translated products (proteins) of genes.
Neaa.Chrplus.Repeat.v1.gff.gz : GFF file (General Feature Format), a text file format used to describe various repeat types, such as LTRs (Long Terminal Repeats) and Tandem Repeats.
Neaa.Chrplus.Function.v1.xls.gz: Table file (Table Format), functional annotation file: Used to describe the annotation of each protein sequence to different functional databases, including KEGG, GO, NR, SwissProt, Trembl, and other functional [illegible]

2)Neas v1:
BioProject ID: PRJNA859258
BioSample ID: SAMN29774552
Assembly methods: Hifiasm 0.16.1-r375, LR_gapcloser, TGS_gapclosr, 3d_dna
Genome coverage: 62.0 X
Sequencing technologies: PacBio Sequell, Oxford Nanopore PromethION, DNBSEQ (Hi-C)
Organism name : Neophocaena asiaeorientalis ssp. sunameri
Sex : Male
Date: 2023-02-24

Files:
Neas.Chrplus.Genome.v1.fasta.gz : Genome assembly in FASTA format, contains the nucleotide composition information for each chromosome.
Neas.Chrplus.Gene.v1.cds.gz : CDS file (Coding Sequence File), contains the coding sequences (CDS) in the form of DNA sequences, typically describing the coding regions of genes.
Neas.Chrplus.Gene.v1.gff.gz : GFF file (General Feature Format), a text file format used to describe genomic features such as genes, exons, promoters, UTRs, etc. It is commonly used for storing genome annotation information.
Neas.Chrplus.Gene.v1.pep.gz : PEP file (Protein Sequence File), contains protein sequences in the form of amino acid sequences, typically describing the translated products (proteins) of genes.
Neas.Chrplus.Repeat.v1.gff.gz : GFF file (General Feature Format), a text file format used to describe various repeat types, such as LTRs (Long Terminal Repeats) and Tandem Repeats.
Neas.Chrplus.Function.v1.xls.gz: Table file (Table Format), functional annotation file: Used to describe the annotation of each protein sequence to different functional databases, including KEGG, GO, NR, SwissProt, Trembl, and other functional [illegible]
md5.txt: MD5 value of each listed file.

For more details, please visit the Figshare website: https://figshare.com/s/1fc632fd4f3cab36b776.

Methods:

Comment 4: One part that is entirely missing in the Methods is the description of how SNPs were generated. Hence, I cannot evaluate the quality of their calls. I highly recommend adding the respective description because otherwise, these steps are not reproducible.

Response: Thank you for your insightful recommendations. Single nucleotide polymorphisms (SNPs) and insertions/deletions (InDels) were identified using methods similar to those previously described [1]. Genome alignment was conducted utilizing the NUCmer program integrated within MUMmer4 (v4.0.0) [2] to compare the v2.0 assembly with the v1.0 assembly, as well as the YFP assembly with the EFP assembly. Utilizing the Maximum Unique Matches (MUM) mode involved setting parameters such as a minimum MUM length of 1000 bp, a minimum similarity threshold of 90%, and the exclusion of matches below 40 bp. Alignment blocks were identified using the delta-filter program, while SNPs and InDels were detected using the show-snps program and Syri (v1.6.3) [3], respectively. Functional annotation of SNPs and InDels is conducted using ANNOVAR [4] to assess their impacts on gene structure and function. Additionally, we utilize the CMplot R package [5] to visually depict the density distribution of these genetic variations. The objective of these analyses was to reveal structural variations between the v1.0 and v2.0 genome assemblies, as well as between the YFP and EFP genome assemblies. Variants were meticulously annotated utilizing the ANNOVAR package (v 2013-06-21) [4]. Correspondingly, we have amended the relevant descriptions in the revised MS at line 468-480.

Ref:

[1] Li, T, Xu, X, Zhao, J, *et al*. Genome assembly of KA105, a new resource for maize molecular breeding and genomic research. *The Crop Journal* 2023;**11**(6):1793-1804.

[2] Marçais, G, Delcher, A, Phillippy, A, *et al*. MUMmer4: A fast and versatile genome alignment system. *PLoS Comput Biol* 2018;**14**(1):e1005944.

[3] Goel, M, Sun, H, Jiao, W, *et al*. SyRI: finding genomic rearrangements and local sequence differences from whole-genome assemblies. *Genome Biol* 2019;**20**(1):277.
[4] Wang, K, Li, M and Hakonarson, H. ANNOVAR: functional annotation of genetic variants from high-throughput sequencing data. *Nucleic Acids Res* 2010;**38**(16):e164.
[5] Yin, L, Zhang, H, Tang, Z, *et al*. rMVP: A Memory-efficient, Visualization-enhanced, and Parallel-accelerated tool for Genome-Wide Association Study. *Genomics, Proteomics & Bioinformatics* 2021;**19**(4):619-28.

Comment 5: The authors use gene enrichment analyses multiple times but do not mention any false discovery rate correction. If this was not done, I highly recommend repeating these analyses with corrected p-values. If it was done, this information needs to be added to the manuscript. I further failed to find what was used as a reference gene set to compare the enrichment to. This is another information that needs to be provided to ensure reproducibility.

Response: We apologize for the oversight; the gene enrichment analysis mentioned in our previous Figures 3 and 4 was indeed incorrect. In the revised manuscript, we have corrected the gene enrichment analysis and have applied the q value to adjust the p values. The updated results are now presented in Figure 6, Supplementary Tables 19-20, 22, 26-29. Specifically, the reference gene set used for enrichment comparison in Figure 6 C, Tables S19, 22, 26, 27 is from YFP v2.0; the reference gene set used for enrichment comparison in Figure 6D, Tables 20, 21, 28, 29 is from EFP v2.0; and the reference gene sets used for enrichment comparison in Figure 6A and B are both from YFP v2.0 and EFP v2.0.

List:

In the following, I'll list the more specific issues I found within the manuscript which may help the authors to revise their manuscript:

Introduction:

Comment 6: Line 43-46: This sentence implies the results of a study, but no citation is provided.

Response: We are sorry for the mistake. We have added the following citations to the sentence: "Based on morphological characteristics, geographic distribution and

molecular genetic evidence, it is generally believed that the finless porpoise can be divided into two species, namely the Indo-Pacific finless porpoise (*N. phocaenoides*) and the narrow-ridged finless porpoise (*N. asiaeorientalis*) [1-2]."

Ref

[1] Wang, J, Frasier, T, Yang, S, *et al*. Detecting recent speciation events: the case of the finless porpoise (genus *Neophocaena*). *Heredity* 2008;**101**(2):145-55.

[2] Jefferson, T and Wang, J. Revision of the taxonomy of finless porpoises (genus *Neophocaena*): The existence of two species. *J Mar Anim Ecol* 2011;**4**(1):3-16.

Comment 7: Line 53: I think the "urgent priority for scientists" needs to be more justified. Consider toning this down or being more specific.

Response: Thank you very much for your suggestion. We have changed "urgent priority for scientists" to "a pressing concern for scientists".

Comment 8: Line 57-58: This sentence implies the results of a study, but no citation is provided.

Response: We are sorry for the mistake. We have added the following citation to the sentence: "The genetic diversity of East Asian finless porpoises surpasses that of the other two populations, indicating it as the likely center of origin for this species [1]."

Ref

[1] Yang, G, Ren, W, Zhou, K, *et al*. Population genetic structure of finless porpoises, *Neophocaena phocaenoides*, in Chinese waters, inferred from mitochondrial control region sequences. *Marine mammal science* 2002;**18**(2):336-47.

Comment 9: Line 67: "Availability of a high-quality genome assembly."

Response: We are sorry for the mistake. We have changed "Availability of high-quality genome assembly…" to "Availability of a high-quality genome assembly…"

Comment 10: Line 79-81: This is not a sentence.

Response: We are sorry for the mistake. We have changed the sentence to "The T2T

genome has emerged as a hotspot genomic research, demonstrating extensive applications to other animal species like chicken and fish."

Comment 11: Line 84: I would suggest to either make the word "genome" plural or add an "a" at the beginning of the sentence.

Response: We are sorry for the mistake. We have added an "a" at the beginning of the sentence.

Comment 12: Line 86-91: Consider changing these sentences to present.

Response: Thank you very much for your suggestion. We have changed these sentences to present.

Comment 13: Line 92-93: This statement is extremely vague. Consider being more specific about what we can learn from their genomes.

Response: Thank you very much for your suggestion. We have changed this statement to "The high-quality chromosomal-level references help elucidate ecological and aquatic adaptation mechanisms in cetaceans."

Results and Discussion:

Comment 14: Line 99: "Based on" doesn't make sense when speaking about additional sequencing efforts.

Response: Thank you very much for your suggestion. We have changed "Based on the previously sequenced 62x PacBio HiFi and 85x Hi-C reads of the EFP, we generated 215 Gb (86x) ONT reads in this study" to "In this study, we supplemented the existing dataset comprising 62x PacBio HiFi and 85x Hi-C reads of the EFP with an additional 215 Gb (86x) of ONT reads."

Comment 15: Line 104-105: How can scaffold and contig statistics be completely identical?

Response: Thank you for drawing our attention to this. The genomic assembly in this

study initially progressed at the contig level by assembling PacBio data using the hifiasm software. Subsequently, redundant contigs were removed through alignment of the assembly with the PacBio data using the purge_haplotigs software, which utilized alignment coverage depth and other relevant metrics. Furthermore, the improved assembly was compared to the complete genome of *Neophocaena asiaeorientalis* mitochondrion (accession number: NC_026456.1) using the minimap2 software to detect possible mitochondrial sequences. The contig with the highest alignment score (temporarily designated as: ptg000094l) was selected as the mitochondrial genome sequence for the species under study, while other potential sequences were excluded. Next, the ultimate contig-level genomic assembly was precisely anchored to the chromosome level utilizing Hi-C data. Finally, gap filling was performed using Nanopore and PacBio data to achieve a chromosome-level assembly that is free of gaps. Upon completion, the scaffold and contig statistics produced identical results.

Comment 16: Line 116-118: In this sentence is a verb missing. Also, consider explaining the mercury score in one sentence since this is something not known by the generally informed reader.

Response: We are sorry for the mistake. We have changed "The Merqury estimated quality values of 60.18 and 64.38 based on k-mer analysis of YFP and EFP, respectively, which indicated that our assemblies were of high quality" to "The quality values obtained from Merqury's k-mer analysis for YFP and EFP were calculated as 60.18 and 64.38, respectively. These values indicated the superior quality of our assemblies. It is essential to emphasize that Merqury quality values span from 0 to 255, with higher values denoting superior quality."

Comment 17: Line 137: You may want to replace "constituting" with "reaching" or "accounting for"

Response: We have replaced "constituting" with "accounting for".

Comment 18: Line 139-143: You have an excessive use of the word "respectively" in

this paragraph.

Response: Thank you very much for your suggestion. We have changed " In total, we predicted 23,139 and 23,101 protein-coding genes in the YFP and EFP genomes, respectively…" to "In total, the number of predicted protein-coding genes was 23,139 in the YFP genome and 23,101 in the EFP genome…"

In addition, we have revised the text with simple summary values at lines 163-172 in the revised MS to minimize redundancy and emphasize the main discoveries. The revised section now offers a succinct overview of the predicted protein-coding genes in the YFP and EFP genomes, supported by evidence and a comparison of length distributions. Supplementary Tables 9-12 contain further information, such as the average lengths of coding sequences, exons, and introns, as recommended.

Comment 19: Line 141-142: Consider maxing the word "exon" and "intron" plural.

Response: Thank you very much for your suggestion. We have removed the average lengths of coding sequences, exons, and introns to supplementary Tables.

Comment 20: Line 145: Consider turning the "was" into present.

Response: We have changed the word "was" to "is".

Comment 21: Line 159: Consider rephrasing "mystery" to something more objective.

Response: Thank you very much for your suggestion. We have changed "The mystery of the evolution of centromere structure…" to "The intrinsic mechanisms underlying the evolution of centromere structure…"

Comment 22: Line 163-165: It is not directly clear what the difference between "candidate centromere region" and "newly identified centromere regions" is. Consider adding a sentence to explain these differences.

Response: We are sorry for the unclear description. The term "candidate centromere region" refers to regions identified by centromere-finding software quartet pipeline as potential centromeres. "Newly identified centromere regions" indicate areas predicted

by the software, which are present in the freshly assembled assembly of this study but absent in the previously published v1.0 version of the *Neophocaena asiaeorientalis* genome.

The method for obtaining these newly assembled regions in this study can be found in the "Genome comparison and Identification of newly assembled genes" section. We have changed the sentence to "A total of 235 and 237 genes were identified in the candidate centromere regions for YFP and EFP, respectively, through predictions generated by centromere-finding software. Moreover, 56 genes were identified in the newly discovered centromere regions for YFP, while 20 genes were found in analogous regions for EFP. The "newly identified centromere regions" refer to specific areas identified in the genome that have been recently assembled but were not included in the previously published version. This discovery may suggest the presence of previously unknown centromere sites."

Comment 23: Line 188: It is not clear what the authors meant with "microbial category". Please add further explanation to this.

Response: We are sorry for the mistake. We have changed "microbial category" to "microbial diversity", and expanded upon this paragraph. Please refer to the following question (**Comment 24**) for further details.

Comment 24: Line: 188-193: These sentences imply the results of a study, but a citation is missing. The paragraph is also very vague. Is there something known about different microbial stressors in sea- and freshwater?

Response: We are sorry for the unclear description. We have revised and expanded upon this paragraph: "The genes coding for the mutated regions of the YFP and EFP are widely enriched in immune-related pathways. This association may be intricately linked to the distinct pathogenic microorganisms unique to freshwater and seawater environments. Marine mammals exhibit a diminished histocompatibility complex (MHC) diversity attributed to decreased encounters with microparasitic diversity in their marine habitat relative to their terrestrial origin. This phenomenon implies that

mammals encounter distinct pathogenic pressures in varied ecological settings, potentially influencing the evolution of immune-related genes. [1]. Evolutionary analyses of the innate immune pattern recognition receptor (TLRs) in the YFP and the marine finless porpoise indicate that the YFP has undergone specific adaptive changes [2]. The microbial diversity and pathogenicity of freshwater and seawater environments vary, leading to distinct effects of pathogenic microorganisms on the organisms in these two types of environments [3]. Therefore, the YFP and EFP would be expected to undergo adaptive evolution to adapt to the pathogen stresses specific to their respective ecological environments, freshwater and seawater."

Ref

[1] Slade, R and McCallum, H. Overdominant vs. frequency-dependent selection at MHC loci. *Genetics* 1992;**132**(3):861–64.

[2] Tian, R, Chen, M, Chai, S, *et al*. Divergent Selection of Pattern Recognition Receptors in Mammals with Different Ecological Characteristics. *J Mol Evol* 2018;**86**(2):138-49.

[3] Lokesh, J and Kiron, V. Transition from freshwater to seawater reshapes the skin-associated microbiota of Atlantic salmon. *Sci Rep* 2016;**25**(6):19707.

Comment 25: Line 195: I don't think it is correct to say that a subspecies "assembled" new genes.

Response: We are sorry for the mistake. We have changed "… the YFP assembled 5480 new genes, while EFP assembled 1453 new genes..." to "…the YFP assembly included 5480 new genes, while the EFP assembly included 1453 new genes..."

Comment 26: Line 203: Consider changing "including" to: "which include 594 species-specific genes"

Response: We are sorry for the mistake. We have changed "including" to "which include 594 species-specific genes".

Comment 27: Line 205-206: You jump from the alignment of genes to the phylogenetic dating without mentioning the phylogenetic tree itself. I would add a sentence between

these two to briefly describe the tree you made before talking about the specific dates you received for the emergence of the two subspecies.

Response: Thank you for your insightful recommendations. We have modified line 273-279 of the article with the following sentences: “Among the dataset, a total of 2,161 single-copy gene families were identified. Subsequently, multiple sequence alignments were conducted, which were then followed by the reconstruction of evolutionary trees. The resulting phylogenetic tree exhibited a consistent topology with previous studies, highlighting its reliability and alignment with established scientific knowledge. This analysis notably revealed the topological arrangement within branches of mammals. The emergence of the EFP branch alongside its YFP counterpart was particularly noteworthy.”

Comment 28: Line 209: I do not think it is correct to call one of both subspecies more conserved than the other. Figure 1B is a synteny comparison between both genomes and without an outgroup it is not possible to infer the ancestral state of both possible rearrangements. If a comparison between other more distantly related genomes happened, I would mention them here as well, otherwise, I would remove this statement and directly talk about the possible rearrangements.

Response: Thank you for your suggestion; our previous description was indeed lacking in rigor. Following your advice, we have discussed the genomic rearrangements of the two subspecies in line 223-225 of the revised manuscript.

Comment 29: Line 212-214: My first suspicion reading this sentence was that the chromosomes simply weren't fully assembled in the EFP genome. But comparing this sentence with Figure 1B, I think the authors intended to say that parts of YFP chromosomes matched parts of multiple chromosomes within the EFP genome. If so, please consider rephrasing this sentence to avoid this confusion.

Response: Thank you very much for your suggestion. We have changed the sentence to " Synteny analysis of the gene order between YFP v2.0 and EFP v2.0 revealed 907

large shared syntenic blocks, encompassing 89.59% (41,428) genes, and 17 chromosomal rearrangements (Figure 3)."

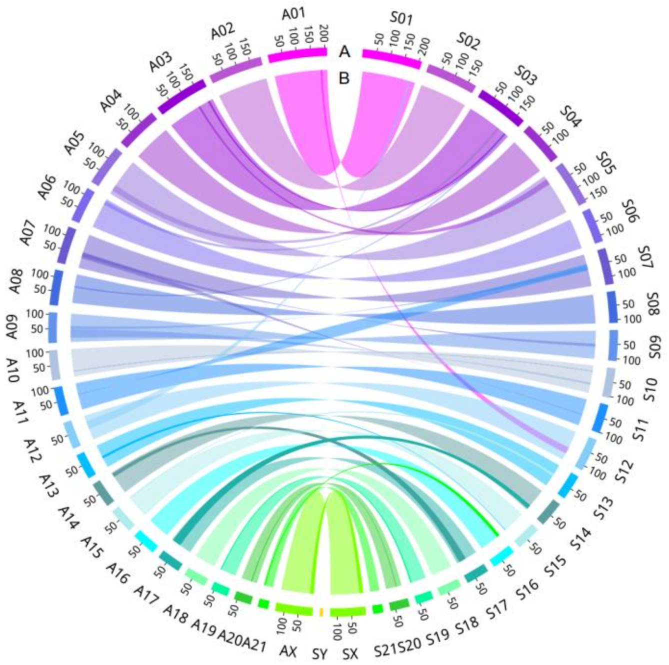

Figure 3. Synteny analysis of YFP v2.0 and EFP v2.0 genomes: A) chromosomes scale, Unit length is Mb; B) syntenic blocks between YFP v2.0 and EFP v2.0.

Comment 30: Line 229-230: Here is a point where the authors should definitely try to contextualize their findings with recent literature. Are these functions that are convergently evolved by other species adapting to freshwater?

Response: Thank you very much for your suggestion. We have revised and expanded upon this paragraph: 'Gene families associated with "oxidative phosphorylation", "cytochrome-c oxidase activity", "riboflavin biosynthetic process", "ferric iron binding" and "iron ion transport" exhibited expansion in finless porpoises. Iron ions play a crucial role in numerous essential physiological functions within living organisms, such as oxygen transportation, electron transport chains, and various metabolic pathways. Several sets of redox reactions are necessary to maintain effective gas exchange in water for cetaceans, and iron is often used in these reactions as an electron acceptor [1]. The expansion of redox reaction and iron ion gene families in cetaceans potentially

improved the efficacy of oxygen utilization and facilitated adaptation to hypoxic conditions in aquatic habitats. An increase in the number of oxidation-reduction and iron-binding gene families was also observed in *L.vexillifer*, a species that encounters hypoxic conditions during dives [2]. The changes in pathogenic microorganisms that occur during the reintroduction of cetaceans from land to sea present a significant challenge to their survival and may have influenced the evolution and adaptation of immune genes [3]. The expansion of immune-related gene families in finless porpoises in this study enhanced the process of antigen presentation and conferred resistance against various pathogenic microorganisms amidst changes in their habitat. Genomic studies of the *S. chinensis* have revealed that cetaceans have developed various species-specific gene families related to immunity and DNA repair, which are linked to potential adaptive mechanisms [4]. We suggest these genes play a crucial role in facilitating hypoxic tolerance and enhancing immune resistance in finless porpoises, thereby reflecting potential mechanisms of adaptation to the aquatic environment. Further investigations are required to elucidate the specific functions of these gene families and their potential significance in the biology of finless porpoises.'

Ref:
[1] Guo, B, Sun, Y, Wang, Y, *et al*. Evolutionary genetics of pulmonary anatomical adaptations in deep-diving cetaceans. *BMC Genomics* 2024;**25**(1):339.
[2] Zhou, X, Sun, F, Xu, S, *et al*. Baiji genomes reveal low genetic variability and new insights into secondary aquatic adaptations. *Nat Commun* 2013;**4**:2708.
[3] Li, L, Rong, X, Li, G, *et al*. Genomic organization and adaptive evolution of IGHC genes in marine mammals. *Mol Immunol* 2018;**99**:75-81.
[4] Ming, Y, Jian, J, Yu, F, *et al*. Molecular footprints of inshore aquatic adaptation in Indo-Pacific humpback dolphin (*Sousa chinensis*). *Genomics* 2019;**111**(5):1034-42.

Comment 31: Line 239: The authors definitely need to elaborate what might specifically cause these genomic instabilities. Is their something known about specific components and how they function as mutagens?

Response: We are sorry for the mistake. We have revised and expanded upon this paragraph: 'The selective pressure to evolve DNA damage repair pathways implied that the Yangtze finless porpoise might be experiencing increased threats to genome stability. The mechanism of DNA damage repair plays a crucial role in preserving genome

integrity by enabling cells to identify and repair DNA damage, thereby averting the accumulation of harmful mutations [1]. In a comparative genomic analysis between the South China tiger and the Amur tiger, it was noted that genes related to DNA repair underwent positive selection in the South China tiger [2]. The observed phenomenon could be explained by the higher probability of genome instability in the temperate and subtropical habitats of the South China tiger. This may be linked to metabolites generated by intestinal microflora, which possess the ability to trigger DNA damage [3]. The stability of the genome or gene expression system in the Yangtze finless porpoise across different organs and life stages remains uncertain. However, one potential interpretation of this data is the suggestion that the Yangtze finless porpoise could be vulnerable to genomic instability triggers in the Yangtze River, such as water pollutants, which may increase the likelihood of DNA damage [4]. Pollutants found in the Yangtze River possess the capacity to accumulate within the food chain, leading to cellular DNA damage and impacting the genome stability of the Yangtze finless porpoise [5-6]. This makes a compelling case for the improved conservation of the species and the development of more rigorous water pollution mitigation practices.'

Ref

[1] Chatterjee, N and Walker, G. Mechanisms of DNA damage, repair, and mutagenesis. *Environ Mol Mutagen* 2017;**58**(5):235-63.

[2] Zhang, L, Lan, T, Lin, C, *et al*. Chromosome-scale genomes reveal genomic consequences of inbreeding in the South China tiger: A comparative study with the Amur tiger. *Mol Ecol Resour* 2023;**23**(2):330-47.

[3] Puschhof, J and Sears, C. Microbial metabolites damage DNA. *Science* 2022;**378**(6618):358-59.

[4] Lv, W, Gu, H, He, D, *et al*. Polystyrene nanospheres-induced hepatotoxicity in swamp eel (*Monopterus albus*): From biochemical, pathological and transcriptomic perspectives. *Sci Total Environ* 2023;**893**:164844.

[5] Zhang, K, Qian, Z, Ruan, Y, *et al*. First evaluation of legacy persistent organic pollutant contamination status of stranded Yangtze finless porpoises along the Yangtze River Basin, China. *Sci Total Environ* 2020;**710**:136446.

[6] Xiong, X, Qian, Z, Mei, Z, *et al*. Trace elements accumulation in the Yangtze finless porpoise (*Neophocaena asiaeorientalis asiaeorientalis*)-A threat to the endangered freshwater cetacean. *Sci Total Environ* 2019;**686**:797-804.

Comment 32: Line 240: Why are these PSGs "interesting"? Again, no context is given.

Response: Thank you very much for your suggestion. We have revised and expanded upon this paragraph: Interestingly, a total of 44 PSGs within the EFP lineage were involved in “sodium-dependent phosphate transport”, “sodium symporter activity”, “aldosterone-regulated sodium reabsorption”, and “calcium signaling pathway”. These metabolic pathways play an important role in regulating sodium levels in the body [1]. Among these PSGs, six were potentially associated with the adaptation of EFP to high osmolarity environment, including the Na(+)/H(+) exchange regulatory cofactor *NHE-RF2* and sodium-dependent phosphate cotransporter *SLC34*. These genes identified are likely to have significant implications in the control of urine formation and the preservation of water-salt metabolic balance [1], suggesting that the East Asian finless porpoise may have a different urine formation process. Previous genome-selective sweep analyses in the finless porpoises revealed that the *SLC14A* in the East Asian finless porpoise underwent positive selection [2]. Comparative analyses conducted at the transcriptome level between the Yangtze finless porpoise and the East Asian finless porpoise revealed a notable upregulation of the *NHE3* in the kidneys of the East Asian finless porpoise. These results imply some adaptive enhanced osmoregulatory capability in the East Asian finless porpoise [3]. The aforementioned studies suggest that the East Asian finless porpoise has evolved a complex and efficient osmoregulatory mechanism as it acclimatized to the hypertonic marine environment, demonstrating adaptations at both molecular and transcriptional levels.

Ref:

[1] Shoemaker, V and Nagy, K. Osmoregulation in amphibians and reptiles. *Annu Rev Physiol*. 1977;**39**: 449-71.

[2] Zhou, X, Guang, X, Sun, D, *et al*. Population genomics of finless porpoises reveal an incipient cetacean species adapted to freshwater. *Nat Commun* 2018;**9**(1):1276.

[3] Ruan, R, Guo, A, Hao, Y, *et al*. De novo assembly and characterization of narrow-ridged finless porpoise renal transcriptome and identification of candidate genes involved in osmoregulation. *Int J Mol Sci* 2015;**16**(1):2220-38.

Methods:

Comment 33: Line 252: I would invite the authors to provide more details of why no ethical considerations were taken into account. I'm also not sure if this is in conflict

with GigaScience's own ethical guidelines. But I would leave this decision to the Editor and the Journal.

Response: The specimens of the YFP and EFP utilized for genome sequencing were obtained from dead individuals found in their natural habitat. The Office of Fishery Supervision and Management in the Yangtze River Basin, Ministry of Agriculture and Rural Affairs of the People's Republic of China has designated our research institution to perform post-mortem analysis and genetic preservation on deceased porpoises.

Comment 34: Line 259: I cannot find which library kit was used for the Hi-C library.

Response: Thanks for the suggestion. We have modified and enriched the methods: 'The PacBio HiFi library was constructed using SMRTbell Prep Kit 3.0 (Pacific Biosciences, USA) and subsequently sequenced on the PacBio Sequel II system in circular consensus sequence (CCS) mode. To collect data for the Hi-C library, the muscle tissues were first fixed in 1% formaldehyde (Sigma) for cross-linking and resuspended in lysis buffer. Then, MboI (NEB) restriction endonucleases were used to fragment the chromatin in the muscle to fragment DNA. The DNA fragments were captured by utilizing Streptavidin-coated magnetic beads (Thermo Fisher SCIENTIFIC) following biotin labeling and crosslinking using T4 DNA Ligase (ENZYMATICS). The Hi-C library was finally sequenced on a BGI MGISEQ platform.'

Comment 35: Line 268: The code for the self-designed Perl program is not provided in any repository and it is not described how the ONT reads were refined in this step.

Response: Thank you for your valuable suggestion. We have revised the text in Line 409: "ONT reads were filtered based on a length < 5 kb and a quality value < 7."

Comment 36: Line 272-274: This is not a sentence. A verb is missing.

Response: We are sorry for the mistake. We have changed "Juicer (v1.5) and 3D-DNA (v180922) pipeline for clustering, ordering, and orienting the contigs into pseudo-chromosomes using Hi-C data." to "We utilized the Hi-C data to cluster, order, and orient the contigs into pseudo-chromosomes through the implementation of the Juicer

(v1.5) and 3D-DNA (v180922) pipeline."

Comment 37: Line 281: The authors chose the OrthoDB for mammals (not stated here but in the results) and I wonder why they did not use the Cetacea specific one. Just a minor comment.

Response: Thank you for your valuable comment. Given that the majority of research on cetaceans has relied on the OrthoDB for mammals for assessments, we chose to ensure consistency in data comparison by employing the same OrthoDB for mammals database in our analysis. For example, Gao, H., *et al*. (2023) employed the OrthoDB database specifically designed for mammals in their research.

Ref:

Gao, H, Kang, H, Zhang, Y, *et al*. Chromosome-Level Genome Assembly of the Rough-Toothed Dolphin (*Steno bredanensis*). *Journal of Marine Science and Engineering* 2023;**11**(2): 418.

Comment 38: Line 286-291: The homology-based repeat annotation was not based on any databases like RepBase? What homologous information was used? Please specify.

Response: Thank you for your reminder. Our previous description was incomplete, as we failed to mention the utilization of the Repbase database in the Repeat analysis process. Consequently, the description in line 441-444 has been revised as follows: "For homolog-based prediction, RepeatMasker (v4.0.7) was utilized to detect DNA transposable elements (TEs), while RepeatProteinMasker (v4.0.7) was employed to identify protein-based TEs, both based on the Repbase database."

Comment 39: Line 301: How is "high quality genes" defined? No information is given.

Response: Thank you for your reminder. "high-quality genes" refers to those genes predicted by the GeMoMa software and validated by OrthoDB for mammals. We have made modifications in the manuscript at line 461-463: "A set of one thousand high-quality genes, which were predicted by the GeMoMa software and validated by OrthoDB for mammals, was randomly selected for training the predictors in Augustus

v3.2.1."

Comment 40: Line 305-315: The paragraph "Genome comparison" and "Identification of new assembled genes" is extremely similarly phrased. Would it be possible to merge them into one, shorter paragraph to avoid redundancy?

Response: Thank you very much for your suggestion. We have merged "Genome comparison" and "Identification of new assembled genes" into one paragraph: "**Genome comparison and Identification of newly assembled genes**

Comment 41: Line 320: Was there no model testing done? Please specify. Also, there is no information given of how many bootstrap replications were conducted. I guess 1000 but this information is not given.

Response: Thank you for your reminder. In this study, model testing was omitted, and the default HKY85 model was used for the phylogenetic analysis. A phylogenetic tree was constructed using the maximum-likelihood method in PhyML, and the reliability of the results was assessed through 1000 bootstrap replications. The phylogenetic relationship among the three lineages of marine mammals is consistent with previous research findings.

Consequently, the description in line 482-488 has been revised as follows: "Single-copy orthologous genes (1:1:1) were aligned using MAFFT (v7.310), a widely utilized tool for multiple sequence alignment. Subsequently, a maximum-likelihood phylogenetic tree was generated using PhyML (v3.3), a commonly employed software tool for phylogenetic analysis. The HKY85 model, known for its capacity to accommodate nucleotide substitution patterns, was applied in the tree construction procedure. To evaluate the reliability of the generated tree, 1000 bootstrap replications were performed, providing statistical evidence for the branching structures."

Ref:

Yuan Y, Zhang Y, Zhang P, *et al*. Comparative genomics provides insights into the aquatic adaptations of mammals[J]. Proceedings of the National Academy of Sciences, 2021, 118(37): e2106080118.

M. R. McGowen, J. Gatesy, D. E. Wildman, Molecular evolution tracks macroevolutionary transitions in Cetacea. Trends Ecol. Evol. 29, 336–346 (2014).

Z. Chen, S. Xu, K. Zhou, G. Yang, Whale phylogeny and rapid radiation events revealed using novel retroposed elements and their flanking sequences. BMC Evol. Biol. 11, 314 (2011).

M. R. McGowen, Toward the resolution of an explosive radiation—Amultilocusphylogeny of oceanic dolphins (Delphinidae). Mol. Phylogenet. Evol. 60,345–357 (2011). 17. M. R. McGowen et al., Phylogenomic resolution of the cetacean tree of life using target sequence capture. Syst. Biol. 69, 479–501 (2020).

Comment 42: Line 337-338: Gene enrichment analysis usually requires some sort of correction for false discovery rates. However, nothing like this was described in the manuscript. If this was done, I would mention it here. Otherwise, I would definitely recommend correcting for this!

Response: We apologize for the oversight; the gene enrichment analysis mentioned in our previous Figures 3 and 4 was indeed incorrect. In the revised manuscript, we have corrected the gene enrichment analysis and have applied the q value to adjust the p values. The updated results are now presented in Figure 6, Supplementary Tables 19-20, 22, 26-29.

Comment 43: Line 339: I guess the word "selection" is missing in this paragraph headline.

Response: We are sorry for the mistake. We have changed the section title to 'Gene positive selection analysis'.

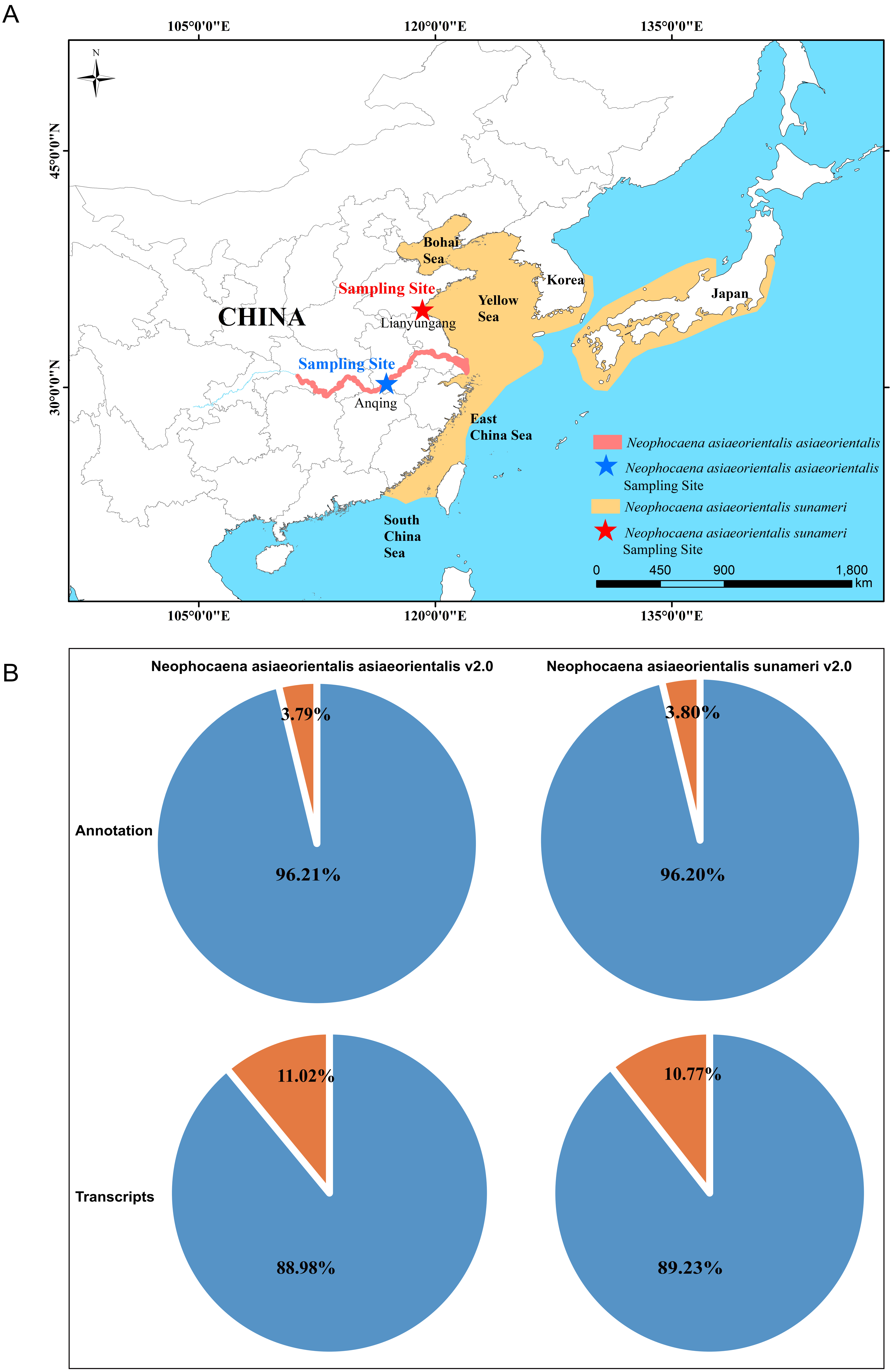
A
105°0'0"E
120°0'0"E
135°0'0"E
45°0'0"N
30°0'0"N
N
CHINA
Bohai Sea
Yellow Sea
Korea
Japan
Sampling Site
Lianyungang
Sampling Site
Anqing
East China Sea
South China Sea
Neophocaena asiaeorientalis asiaeorientalis
Neophocaena asiaeorientalis asiaeorientalis Sampling Site
Neophocaena asiaeorientalis sunameri
Neophocaena asiaeorientalis sunameri Sampling Site
0 450 900 1,800 km
B
Neophocaena asiaeorientalis asiaeorientalis v2.0
Neophocaena asiaeorientalis sunameri v2.0
Annotation
3.79%
96.21%
3.80%
96.20%
Transcripts
11.02%
88.98%
10.77%
89.23%

Figure 2

Click here to access/download;Figure;Figure 2.pdf

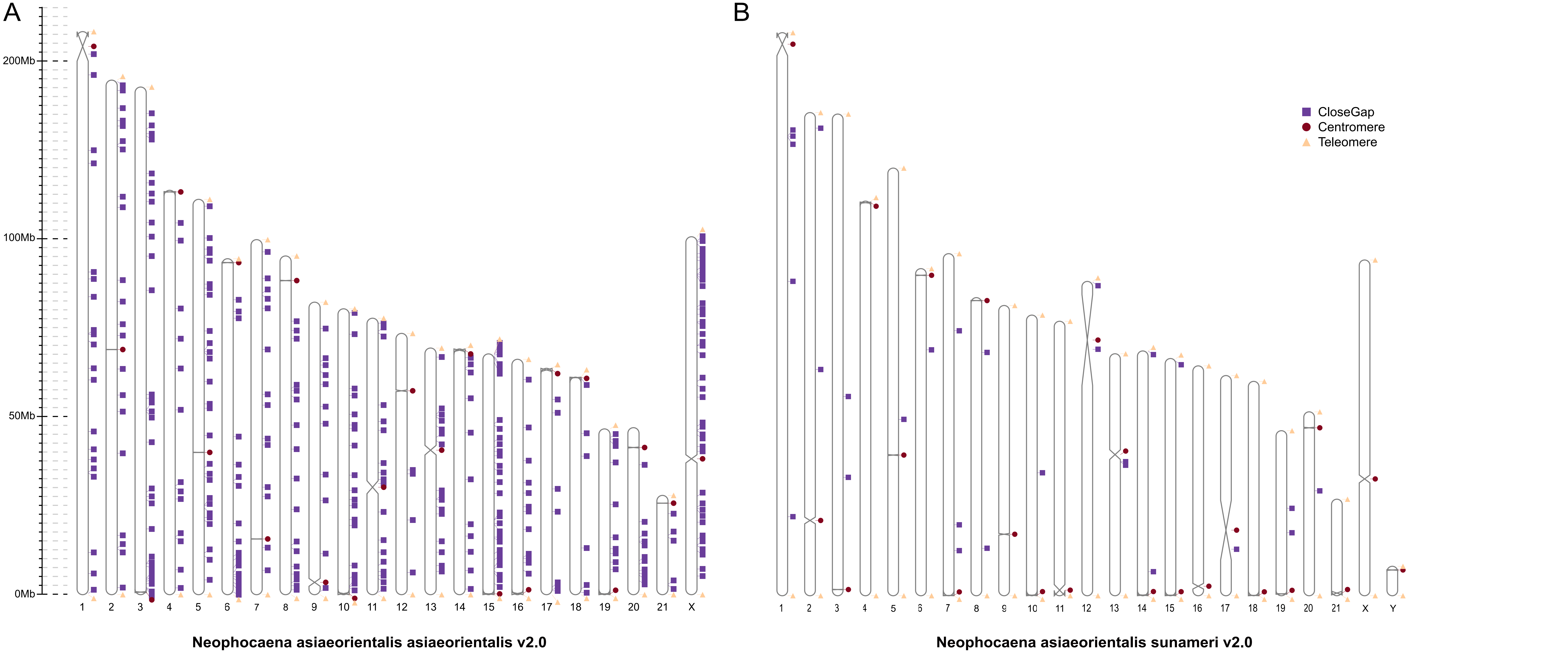

Figure 3

Click here to access/download;Figure;Figure 3.pdf

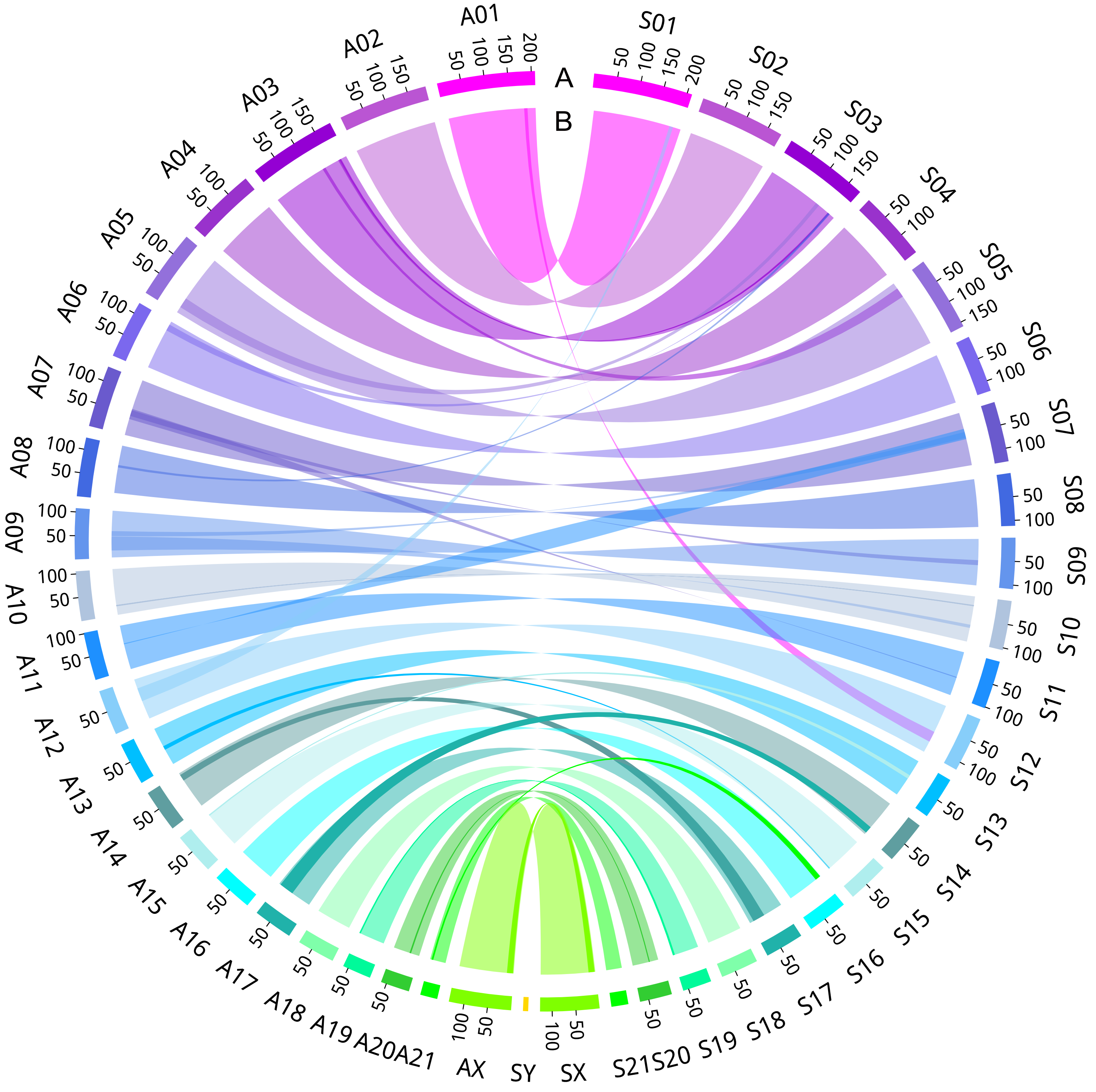

Figure 4

Click here to access/download;Figure;Figure 4.pdf

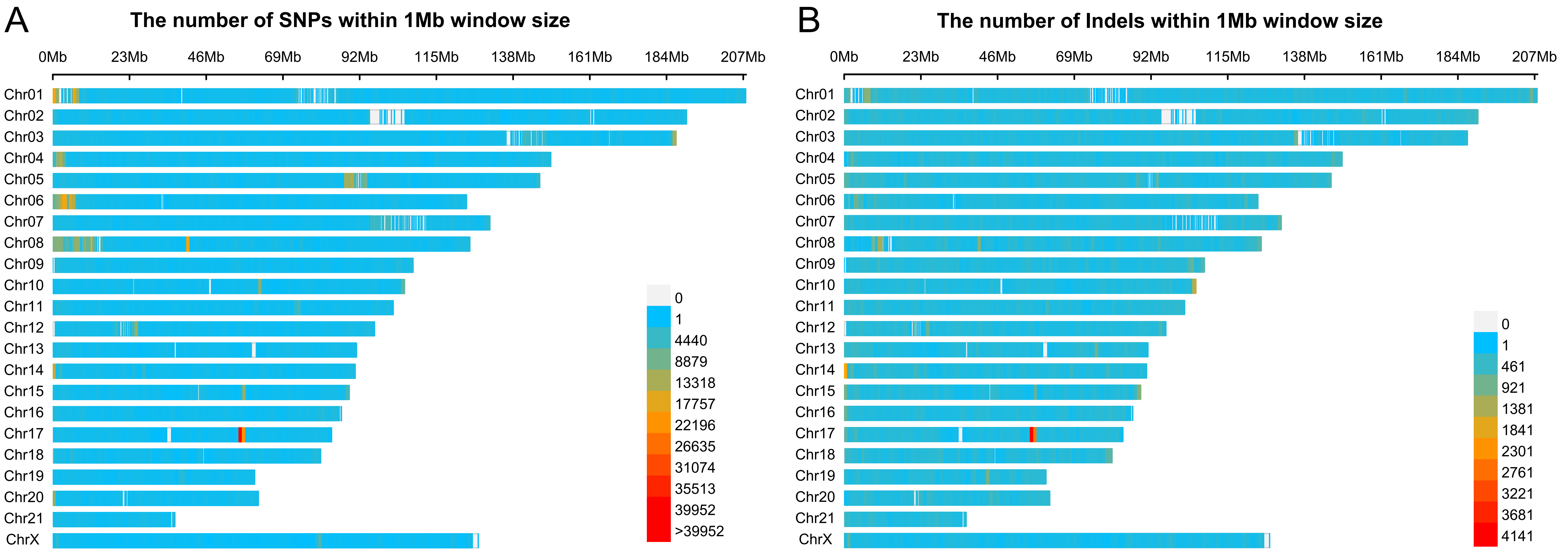

Click here to access/download;Figure;Figure 5.pdf

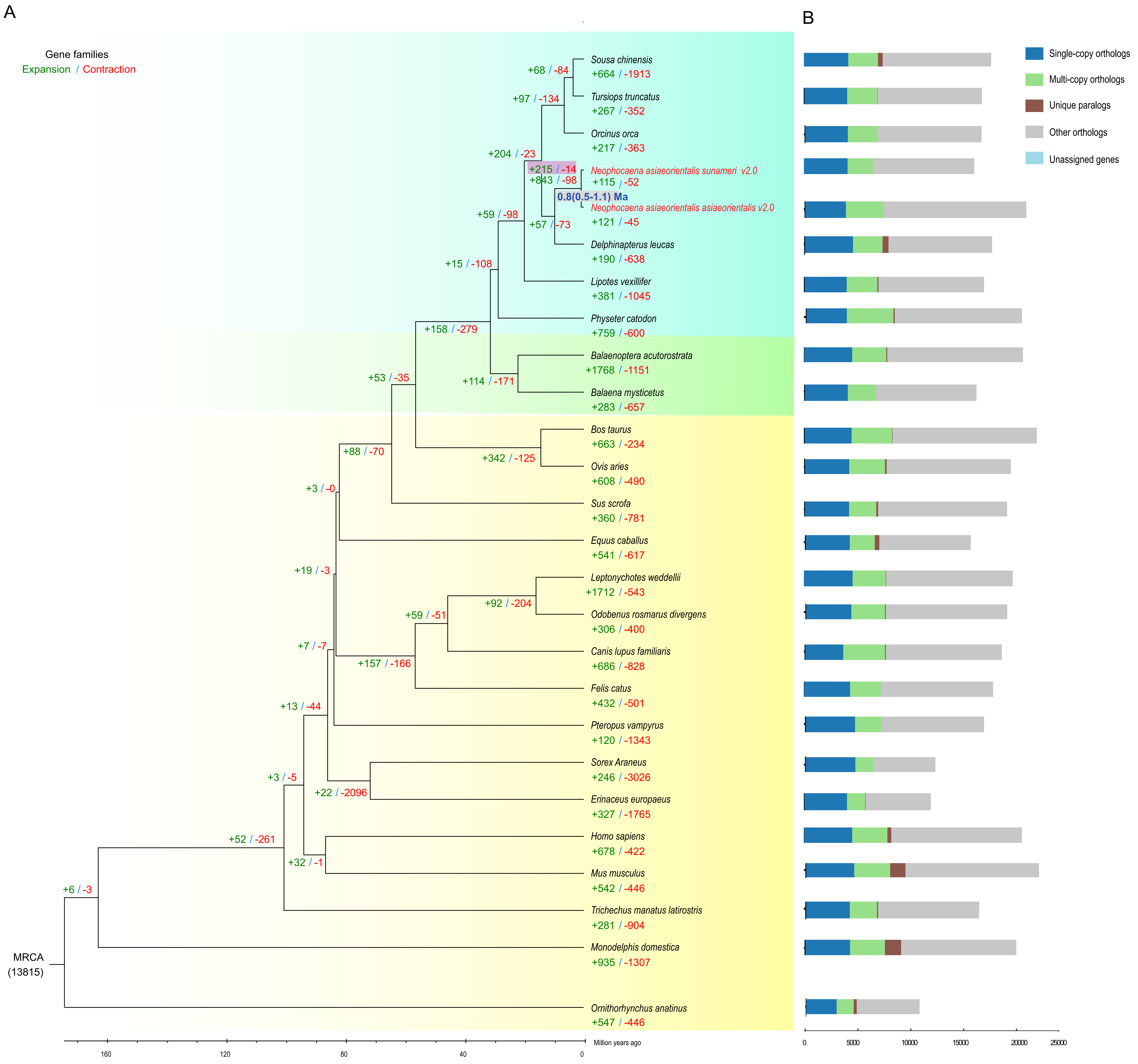

Figure 6

Click here to access/download;Figure;Figure 6.pdf

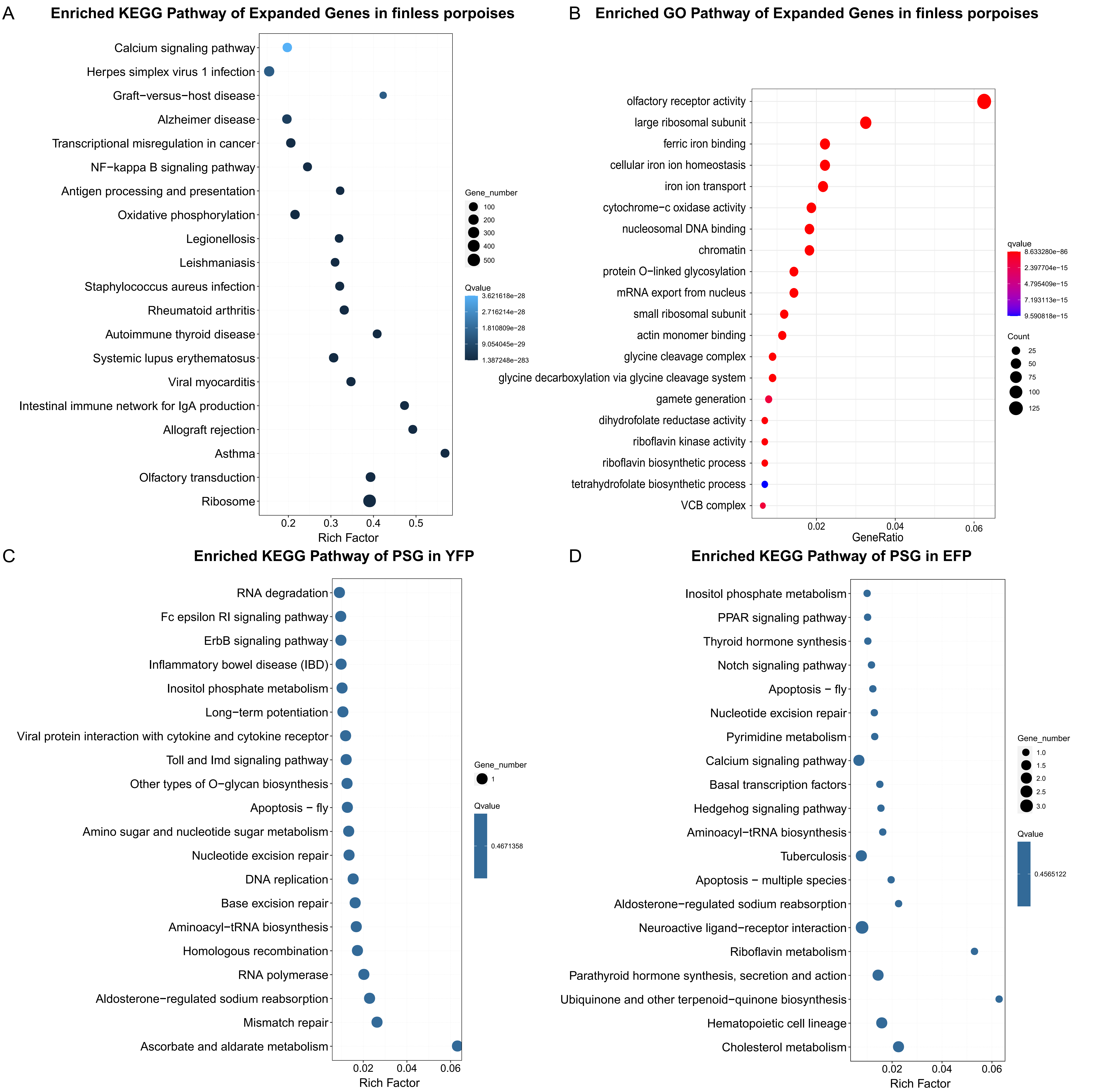

Click here to access/download
Supplementary Material
Supplementary Materials.docx

Supplementary Table S16

Click here to access/download
Supplementary Material
Supplementary Table S16.xls

Supplementary Table S17

Click here to access/download
Supplementary Material
Supplementary Table S17.xls

Supplementary Table S18

Click here to access/download
Supplementary Material
Supplementary Table S18.xls

Click here to access/download
Supplementary Material
Supplementary Table S19.xls

Supplementary Table S20

Click here to access/download

Supplementary Material

Supplementary Table S20.xls

Supplementary Table S22

Click here to access/download
Supplementary Material
Supplementary Table S22.xls

Supplementary Table S24

Click here to access/download
Supplementary Material
Supplementary Table S24.xls

Supplementary Table S25

Click here to access/download
Supplementary Material
Supplementary Table S25.xls

Supplementary Table S26

Click here to access/download
Supplementary Material
Supplementary Table S26.xls

Supplementary Table S27

Click here to access/download
Supplementary Material
Supplementary Table S27.xls

Supplementary Table S28

Click here to access/download
Supplementary Material
Supplementary Table S28.xls

Supplementary Table S29

Click here to access/download
Supplementary Material
Supplementary Table S29.xls
