# Supplementary material for: Telomere-to-telomere gap-free genome assembly of the endangered Yangtze finless porpoise and East Asian finless porpoise
Source: Gigascience. 2024 Sep 16;13:giae067. doi: 10.1093/gigascience/giae067 (PMC11403816; doi:10.1093/gigascience/giae067)
Supplement: giae067_GIGA-D-23-00359_Revision_2 [file giae067_giga-d-23-00359_revision_2.pdf]

GigaScience

Telomere-to-telomere gap-free genome assembly of the endangered Yangtze finless porpoise and East Asian finless porpoise

--Manuscript Draft--

**Manuscript Number:**	GIGA-D-23-00359R2
**Full Title:**	Telomere-to-telomere gap-free genome assembly of the endangered Yangtze finless porpoise and East Asian finless porpoise
**Article Type:**	Research
**Funding Information:**	the National Key R&D Program of China (2021YFD1200304) — Doctor Danqing Lin
**Abstract:**	The Yangtze finless porpoise (Neophocaena asiaeorientalis asiaeorientalis, YFP) and the East Asian finless porpoise (Neophocaena asiaeorientalis sunameri, EFP) are two subspecies of the narrow-ridged finless porpoise that live in fresh and salt water, respectively. They serve as ideal models for the study of freshwater adaptation and evolution. The main objective of this study was to provide contiguous chromosome-level genome assemblies for YFP and EFP. Here, we generated and upgraded the genomes of YFP and EFP at the telomere-to-telomere level through the integration of PacBio HiFi long reads, ultra-long ONT reads and Hi-C sequencing data. The total sizes of the genomes are 2.48 Gb and 2.50 Gb, respectively. The scaffold N50 of two genomes was 125.12 Mb (YFP) and 128 Mb (EFP) with one contig for one chromosome. The telomere repeat and centromere position were clearly identified in both YFP and EFP genomes. Telomeric repeat units were detected at 85% and 90% of the chromosome at both ends in YFP and EFP genomes, respectively. In total, 5,480 new-found genes were detected in the YFP genome, including 56 genes located in the newly identified centromere regions. Additionally, synteny blocks, structural similarities, phylogenetic relationships, gene family expansion and inference of selection were studied in connection with the genomes of other related mammals. Significant improvements were observed in genome contiguity, the number of scaffolds and gaps, and annotation compared to the first drafts of the YFP (GCA_003031525.2) and EFP (GCA_026225855.1) genome assemblies. The improvement of telomere-to-telomere gap-free reference genome resources support conservation genetics and population management for finless porpoises.
**Corresponding Author:**	Kai Liu, Ph.D<br>CAFS FFRC: Chinese Academy of Fishery Sciences Freshwater Fisheries Research Center<br>Wuxi, CHINA
**Corresponding Author Secondary Information:**	
**Corresponding Author's Institution:**	CAFS FFRC: Chinese Academy of Fishery Sciences Freshwater Fisheries Research Center
**Corresponding Author's Secondary Institution:**	
**First Author:**	Denghua Yin
**First Author Secondary Information:**	
**Order of Authors:**	Denghua Yin
	Chunhai Chen
	Danqing Lin
	Zhong Hua
	Congping Ying
	Jialu Zhang
	Chenxi Zhao

Powered by Editorial Manager® and ProduXion Manager® from Aries Systems Corporation

	Yan Liu
	Zhichen Cao
	Han Zhang
	Chenhe Wang
	Liping Liang
	Pao Xu
	Jianbo Jian
	Kai Liu, Ph.D
**Order of Authors Secondary Information:**	
**Response to Reviewers:**	Dear Dr. Hans Zauner,<br>Thanks for your kind consideration and comments regarding to our manuscript entitled "Telomere-to-telomere gap-free genome assembly of the endangered Yangtze finless porpoise and East Asian finless porpoise" (GIGA-D-23-00359R1). We have revised our manuscript (details are provided below) in accordance with all comments and suggestions from the reviewer 2. We hope the revised manuscript have been improved and meet the standard requirement. We look forward to hearing from GigaScience soon.<br><br>Sincerely yours,<br><br><br>Correspondence: Kai Liu<br>Freshwater Fisheries Research Center, Chinese Academy of Fishery Sciences, Wuxi 214081, China.<br>E-mail: liuk@ffrc.cn<br><br>Response to reviewer 2's comments:<br>Reviewer #2: In this manuscript, Denghua Yin and colleagues present two new, high-quality, de novo genome assemblies of two narrow-ridged finless porpoise (Neophocaena asiaeorientalis) subspecies (Yangtze, YFP and East Asia, EFP) as well as a comprehensive report on its quality and analyses that identify molecular differences related to their different habitats (fresh and sea water). This is the second time I'm reading this manuscript and I think the manuscript was substantially improved by the changes made in response to the last review. I still regard these genomes to be of the highest quality and a valuable resource for future studies. The methodological shortcomings which I highlighted last time were completely addressed and the language problems were amended in most cases. The only general issue that wasn't covered enough since last time is in the discussion where the authors fail to provide more comparisons to assemblies from the literature. The authors mention in the conclusion that these assemblies are potentially the "most continuous" genomes of cetacean today and I tend to believe them. However, the manuscript is lacking a clear comparison to other assemblies to make this statement. I already suggested this in my last review, and I consider this to be crucial, although I'm aware that this conflicts to some extent with the suggestions made by reviewer 1. Hence, I suggest to the authors to add just a brief comparison (2-3 sentences) to the discussion to validate this claim, to provide some contextual statistics and to give the reader an idea about the improved quality of these genomes. In the following, I'll list some additional suggestions that may improve the manuscript. Given that these suggestions require only minor changes to the text, I recommend a minor revision to the editor.<br>Response: Thank you very much for your comments on our manuscript. The comments provided are all valuable and very helpful for revising and improving our paper. We have addressed the only general issue raised in comment 1. Based on the comments we received, detailed modifications are provided below.<br><br>General issues:<br>Comment 1: As mentioned already, I suggest adding a small comparative paragraph to the discussion to compare the quality of these genomes to previous genomes of Cetacea.<br>Response: According to the reviewer's suggestion, we have compared the genome

Powered by Editorial Manager® and ProduXion Manager® from Aries Systems Corporation

assemblies presented in this study with 34 cetacean genomes available from public repositories such as NCBI and CNGB. The comprehensive analysis confirms that our assemblies are the most continuous among cetaceans known to date. Notably, our contig N50 and scaffold N50 values significantly surpass those of other genomes, while the number of contigs is considerably lower, highlighting the improved continuity and quality of our assemblies. In addition, a table summarizing the statistical comparison of the genomes has been included. This table is now cited in the main text and is presented as Supplementary Table S3. This table provides a concise overview of the data that underpins our assertions and enables a quick assessment of the comparative genomic consistency among cetacean species.
We have added a comparative paragraph at lines 120-129 in the revised MS: "As of May 2024, the killer whale genome assembly (GCF_937001465.1) stands out among the 34 publicly available cetacean genomes sourced from the NCBI (National Center for Biotechnology Information) and CNGBdb (China National GeneBank DataBase) databases. It features an N50 length of 45.6 Mb and is composed of 570 contigs. In our study, the two newly assembled genomes exhibit a marked improvement in contiguity. The contig N50 length achieved in our assemblies is threefold that of the killer whale genome, with the number of contigs comprising only 4.03% of the latter. Moreover, the analysis of the blue whale genome (GCF_009873245.2) shows a contig N50 value that is only 4.0% of our assemblies, while its contig count is 42 times higher [1] (Supplementary Table S3). The comparative metrics highlight the superior continuity of the YFP and EFP genome assemblies in this study."
Ref:
[1] Bukhman, Y, Morin, P, Meyer, S, et al. A high-quality blue whale genome, segmental duplications, and historical demography. Mol Biol Evol 2024; 41(3): msae036.

Supplementary Table S3 Assembly statistics for 36 species of cetaceans.
SpeciesCommon nameGenome Size/GbContig N50/MbContig NumbersSuper-scaffold N50/MbScaffold NumberData Source
Neophocaena asiaeorientalis sunameriEast Asian finless porpoise (EFP v2.0)2.50 128.00 23 128.00 23 This study (PRJNA859258)
Neophocaena asiaeorientalis asiaeorientalisYangtze finless porpoise (YEP v2.0)2.48 125.12 22 125.12 22 This study (PRJNA915046)
Neophocaena asiaeorientalis asiaeorientalisEast Asian finless porpoise (EFP v1.0)2.50 84.69 52 122.40 23 GCA_026225855.1
Orcinus orcaKiller whale2.645.6570 114.20 447 GCF_937001465.1
Kogia brevicepsPygmy sperm whale2.60 42.80 723 122.40 580 GCF_026419965.1
Inia geoffrensisBoutu2.70 40.90 1,186 117.40 902 GCA_036417435.1
Eschrichtius robustusGrey whale3.00 39.2982 126.50 703 GCA_028021215.1
Eubalaena glacialisNorth Atlantic right whale2.80 37.50 1,155 129.60 788 GCF_028564815.1
Balaenoptera ricei
Rice's whale2.70 36.50 1,108 122.10 774 GCF_028023285.1
Phocoena sinusvaquita2.40 20.20 272 115.50 64 GCF_008692025.1
Tursiops truncatusBottlenosed dolphins2.37 9.73 1,035 108.43 361 GCF_011762595.1
Balaenoptera musculusBlue whale2.40 6.30 972 110.30 105 GCF_009873245.2
Phocoena phocoenaHarbor porpoise2.50 3.71,405 110.2 439 GCA_963924725.1
Delphinus delphissaddleback dolphin2.70 3.60 1,598 107.1 630 GCF_949987515.1
Stenella coeruleoalbaStriped dolphin2.70 3.60 1,630 105.30 637 GCA_951394435.1
Lagenorhynchus albirostrisWhite-beaked dolphin2.53.40 1,479 110.7 409 GCF_949774975.1
Globicephala melasLong-finned pilot whale2.70 3.30 2,076 105.10 992 GCF_963455315.1
Hyperoodon ampullatusNorthern bottlenose whale2.80 3.10 1,854 117.4 756 GCA_949752795.1
Balaenoptera acutorostrataMinke whale2.80 3.00 2,551 116.50 1,374 GCF_949987535.1
Lagenorhynchus obliquidensPacific white-sided dolphin2.46 0.28 29,411 30.04 13,174 CNA0007259
Delphinapterus leucasBeluga whale2.32 0.20 29,443 31.18 5,904 GCF_002288925.2
Sousa chinensisIndo-Pacific humpback dolphin2.34 0.18 46,900 9.01 20,903 GCA_003521335.2
Pseudorca crassidensFalse killer whale2.43 0.15 60,880 32.50 32,690 CNA0007265

Tursiops aduncusIndo-Pacific bottlenose dolphin2.41 0.11 160,669 104.71 118,124 CNA0007267
Neophocaena asiaeorientalis asiaeorientalisYangtze finless porpoise (YEP v1.0)2.27 0.09 66,342 6.34 13,695 GCF_003031525.2
Balaenoptera musculusBlue whale2.37 0.08 79,155 8.28 28,500 CNA0007254
Balaenoptera brydeBryde’s whale2.37 0.07 140,815 103.91 74,190 CNA0007253
Grampus griseusRisso's dolphin2.50 0.06 813,233 93.20 773,381 GCA_028646425.1
Kogia simaDwarf sperm whale2.59 0.06 503,547 26.52 428,024 CNA0007257
Steno bredanensisRough-toothed dolphin2.30 0.06 266,806 94.90 221,678 GCA_028646385.1
Lagenorhynchus australisPeale’s dolphin2.39 0.05 225,716 0.38 151,990 CNA0007258
Balaena mysticetusBowhead whale2.31 0.04 118,156 0.88 7,227 bowhead-whale.org
Physeter catodonSperm whale2.51 0.04 133,554 122.18 12,168 GCF_002837175.3
Lipotes vexilliferYangtze River dolphin2.37 0.03 148,597 2.44 24,809 GCF_000442215.2
Mesoplodon densirostrisBlainville’s beaked whale2.47 0.03 1,071,210 0.80 944,262 CNA0007260
Neophocaena phocaenoidesIndo-Pacific finless porpoise2.62 0.01 2,048,710 0.43 1,806,060 CNA0007261

Abstract:
Comment 2: Line 31: I do not think you can "study comparative genomes". Consider "study structural similarities" instead.
Response: Thank you very much for your suggestion. We have changed "comparative genomes" to "structural similarities".

Introduction:
Comment 3: Line 95-98: Same as in line 30. Furthermore, the article "the" is used in a confusing way here. Consider changing the sentence to: "We compare the quality of the newly drafted assemblies with previously available versions and explore synteny blocks, structural similarities, phylogenetic relationships, gene family expansion and inference of selection in relation to several other mammals."
Response: We are sorry for the mistake. We have changed the sentence to: "We compare the quality of the newly drafted assemblies with previously available versions and explore synteny blocks, structural similarities, phylogenetic relationships, gene family expansion and inference of selection in relation to several other mammals."

Results and Discussion:
Comment 4: Line 127: Consider changing "a gap-free genome" to a plural form.
Response: Thank you very much for your suggestion. We have changed "a gap-free genome" to "gap-free genomes".

Comment 5: Line 132: Thank you for choosing to explain the Merqury scores. However, I'm missing the sentences: "These values indicated the superior quality of our assemblies. It is essential to emphasize that Merqury quality values span from 0 to 255, with higher values denoting superior quality." They were mentioned in the response to the reviewer but not in the manuscript. Is there a reason for this?
Response: We apologize for the mistake. The sentences "These values indicated the superior quality of our assemblies. It is essential to emphasize that Merqury quality values span from 0 to 255, with higher values denoting superior quality." have been included at lines 131-133 in the revised MS.

Comment 6: Line 220-221: A citation is missing for this statement.
Response: We are sorry for the mistake. We have added a citation for this statement.
Ref:
Brar, G, Amon, A. Emerging roles for centromeres in meiosis I chromosome segregation. Nat Rev Genet 2008;9(12):899-910.

Comment 7: Line 283: Although the authors probably mentioned the paper somewhere already, I would suggest adding a citation for this first subspecies classification here.
Response: Thank you for your suggestion. We have added a citation for this first

subspecies classification here.
Ref:
Zhou, X, Guang, X, Sun, D, et al. Population genomics of finless porpoises reveal an incipient cetacean species adapted to freshwater. Nat Commun 2018;9(1):1276.

Comment 8: Line 312: The genus name of Lipotes vexillifer is already shortened here to a "L." although its not written out beforehand in the manuscript. I would suggest writing out the name here and shorten it at line 329.
Response: Thank you very much for your suggestion. We have written out "Lipotes vexillifer" at line 283, and shorten it as "L. vexillifer" at line 300 in the new revised MS. In addition, we have abbreviated "N. a. asiaeorientalis" and "N. a. sunameri" at line 296-298, as well as "B. mysticetus", "B. acutorostrata", "S. chinensis" and "T. truncates" at line 453-454 since they were previously mentioned in the manuscript.

Methods:
Comment 9: Line 444: I appreciate that the authors clarified the RepBase usage. However, for this workflow here to be reproduceable, you should usually provide the version number of RepBase and/or the date when the database was retrieved. Consider adding this information to ensure reproducibility.
Response: Thank you very much for your suggestion. We have provided the version number of RepBase database (v21.12) at line 401 in the revised MS.

Comment 10: Line 467: Consider changing the capital to a lower case "I" in "Information".
Response: We are sorry for the mistake. We have changed "Genome comparison and Identification of newly assembled genes" to "Genome comparison and identification of newly assembled genes".

Comment 11: Line 469: described where? Please add details to this statement.
Response: We are sorry for the unclear description. We have added a citation for this statement.
Ref:
Li, T, Xu, X, Zhao, J, et al. Genome assembly of KA105, a new resource for maize molecular breeding and genomic research. The Crop Journal 2023;11(6):1793-1804.

Comment 12: Line 499+501: I'm not sure if the phrases "a widely utilized tool for multiple sequence alignment" and "a commonly employed software tool for phylogenetic analysis" are the result of some corrections meant to answer suggestions made by reviewer 1, but for me, these phrases sound unnecessary and strange.
Response: We are sorry for the unclear description. We have changed "Single-copy orthologous genes (1:1:1) were aligned using MAFFT (v7.310), a widely utilized tool for multiple sequence alignment. Subsequently, a maximum-likelihood phylogenetic tree was generated using PhyML (v3.3), a commonly employed software tool for phylogenetic analysis." to "We utilized MAFFT (v7.310) and PhyML (v3.3) software tools to align single-copy orthologous genes (1:1:1) and construct a maximum-likelihood phylogenetic tree, respectively."

Conclusion:
Comment 13: Line 569: Here the authors clearly state that these genomes are probably the "most continuous" genomes of cetacean thus far. Again, for this statement to be justified, I strongly recommend adding numbers and citations to this claim somewhere in the discussion.
Response: Thank you very much for your suggestion. We have addressed the only general issue raised in comment 1. In addition, we have added the sentence in the Conclusion section: Compared to the 34 cetacean genomes sourced from public databases, the two genomes newly assembled in our study exhibit the longest contig N50 and scaffold N50, along with the lowest number of contigs, thus achieving superior continuity.

Figures:
Comment 14: Figure 5 (Phylogeny): Although beautiful, I'm very sure that the time axis in the bottom of the graph won't be readable when printed out or implemented within a

	PDF version of the article. This axis is just too small.<br>Response: Thank you for your feedback. We have increased the size of the time axis at the bottom of the graph to ensure it is readable when printed or viewed in the PDF version of the article.
**Additional Information:**	
**Question**	**Response**
Are you submitting this manuscript to a special series or article collection?	No
**Experimental design and statistics**<br><br>Full details of the experimental design and statistical methods used should be given in the Methods section, as detailed in our Minimum Standards Reporting Checklist. Information essential to interpreting the data presented should be made available in the figure legends.<br><br>Have you included all the information requested in your manuscript?	Yes
**Resources**<br><br>A description of all resources used, including antibodies, cell lines, animals and software tools, with enough information to allow them to be uniquely identified, should be included in the Methods section. Authors are strongly encouraged to cite Research Resource Identifiers (RRIDs) for antibodies, model organisms and tools, where possible.<br><br>Have you included the information requested as detailed in our Minimum Standards Reporting Checklist?	Yes
**Availability of data and materials**<br><br>All datasets and code on which the conclusions of the paper rely must be either included in your submission or deposited in publicly available repositories (where available and ethically appropriate), referencing such data using	Yes

a unique identifier in the references and in the “Availability of Data and Materials” section of your manuscript.<br><br>Have you have met the above requirement as detailed in our Minimum Standards Reporting Checklist?	

Manuscript Click here to access/download;Manuscript;Manuscript.docx

Telomere-to-telomere gap-free genome assembly of the endangered Yangtze finless porpoise and East Asian finless porpoise

Denghua Yin[1†], Chunhai Chen[2†], Danqing Lin[1†], Zhong Hua[1], Congping Ying[3], Jialu Zhang[1], Chenxi Zhao[2], Yan Liu[1], Zhichen Cao[4], Han Zhang[4], Chenhe Wang[2], Liping Liang[2], Pao Xu[1,3], Jianbo Jian[2,5,*] and Kai Liu[1,3,4,*]

[1]Key Laboratory of Freshwater Fisheries and Germplasm Resources Utilization, Ministry of Agriculture and Rural Affairs, Freshwater Fisheries Research Center, Chinese Academy of Fishery Sciences, Wuxi 214081, China.

[2]BGI Genomics, Shenzhen 518083, China.

[3]Wuxi Fisheries College, Nanjing Agricultural University, Wuxi 214081, China.

[4]National Demonstration Center for Experimental Fisheries Science Education, Shanghai Ocean University, Shanghai 201306, China.

[5]Guangdong Provincial Key Laboratory of Marine Biotechnology, Shantou University, Shantou 515063, China.

[†]Authors contributed equally to this work.

[*]Correspondence address: E-mail: jianjianbo@bgi.com, liuk@ffrc.cn.

Abstract

The Yangtze finless porpoise (*Neophocaena asiaeorientalis asiaeorientalis*, YFP) and the East Asian finless porpoise (*Neophocaena asiaeorientalis sunameri*, EFP) are two subspecies of the narrow-ridged finless porpoise that live in fresh and salt water, respectively. They serve as ideal models for the study of freshwater adaptation and evolution. The main objective of this study was to provide contiguous chromosome-level genome assemblies for YFP and EFP. Here, we generated and upgraded the genomes of YFP and EFP at the telomere-to-telomere level through the integration of PacBio HiFi long reads, ultra-long ONT reads and Hi-C sequencing data. The total sizes of the genomes are 2.48 Gb and 2.50 Gb, respectively. The scaffold N50 of two genomes was 125.12 Mb (YFP) and 128 Mb (EFP) with one contig for one chromosome. The telomere repeat and centromere position were clearly identified in both YFP and EFP genomes. Telomeric repeat units were detected at 85% and 90% of the chromosome at both ends in YFP and EFP genomes, respectively. In total, 5,480 new-found genes were detected in the YFP genome, including 56 genes located in the newly identified centromere regions. Additionally, synteny blocks, structural similarities, phylogenetic relationships, gene family expansion and inference of selection were studied in connection with the genomes of other related mammals. Significant improvements were observed in genome contiguity, the number of scaffolds and gaps, and annotation compared to the first drafts of the YFP (GCA_003031525.2) and EFP (GCA_026225855.1) genome assemblies. The improvement of telomere-to-telomere gap-free reference genome resources support conservation genetics and population management for finless porpoises.

Keywords: telomere-to-telomere; genome assembly; Yangtze finless porpoise; gap-free; HiFi sequencing; Hi-C sequencing

Introduction

Finless porpoises (*Neophocaena* spp.) are small toothed whales capable of inhabiting freshwater (Yangtze River) and saltwater (coastal waters of southern and eastern Asia) environments [1, 2]. They are characterized by a blunt, rounded head, an equal width upper and lower jaw, and lack of an obvious dorsal fin [3, 4]. Based on morphological characteristics, geographic distribution and molecular genetic evidence, it is generally believed that the finless porpoise can be divided into two species, namely the Indo-Pacific finless porpoise (*N. phocaenoides*) and the narrow-ridged finless porpoise (*N. asiaeorientalis*) [5, 6]. In China, there exist two subspecies of the narrow-ridged finless porpoise: one is the freshwater Yangtze finless porpoise (*N. a. asiaeorientalis*, YFP), which exclusively inhabits the middle and lower reaches of the Yangtze River and adjacent Dongting and Poyang lakes. The other subspecies is the marine East Asian finless porpoise (*N. a. sunameri*, EFP), which can be found in the coastal waters of the Yellow Sea and Bohai Sea, and in the northern waters of the East China Sea [5, 6] (Figure 1A).

The investigation of the evolutionary origins and conservation genetics of finless porpoises is a pressing concern for scientists. Yang *et al.* identified significant genetic structure between either the Yangtze River population or the Yellow Sea population and the South China Sea population by analyzing the sequences of mtDNA control region of finless porpoises in Chinese waters [7]. This result was supported by subsequent mtDNA sequences, nuclear DNA microsatellites, single nucleotide polymorphisms (SNPs) and MHC loci [8-12]. The genetic diversity of East Asian finless porpoises surpasses that of the other two populations, indicating it as the likely center of origin for this species [7]. Zheng *et al.* analyzed the sequences of the mtDNA control region of seven local populations of Yangtze finless porpoises in the middle and lower reaches of the Yangtze River, and found that the overall level of genetic diversity was low. Notably, the downstream population showed richer genetic variation than the midstream population. Such a genetic pattern reflects, to some extent, the marine origin and evolutionary history of the Yangtze population [13]. Based on genomic analysis of finless porpoise populations, significant genetic structure was identified among the three populations, indicating local adaptive evolution and emphasizing the evolutionary distinctiveness and conservation significance of the Yangtze finless porpoise [14].

Availability of a high-quality genome assembly is not only critical for the genomic studies of

finless porpoises, but also would be a valuable resource for comparative genomics and evolutionary studies of cetaceans. The first draft of the YFP genome assembly (GCF_003031525.2) was published in 2018 with a size of 2.3 Gb generated by short-read sequencing on the Illumina HiSeq 2000 platform [14]. The assembly comprised 13,698 scaffolds, with a scaffold N50 of 6.3 Mb, excluding the minimum sequence length (100 bp) consideration. Although progress had been made in genome-wide studies of YFP through the availability of this draft, such as immune changes with age and gene expression profiles in different habitats [15, 16], the lack of chromosomal information limits the potential application of the data. Recent advances in ultra-long ONT and PacBio HiFi sequencing technologies, as well as assembly algorithms, have facilitated the development of telomere-to-telomere (T2T) genome assemblies. The completion of the T2T human genome sequence and the full Y chromosome sequence represents a significant milestone in the field of human genomics research [17, 18]. The T2T genome has emerged as a hotspot genomic research, demonstrating extensive applications to other animal species like chicken and fish [19, 20]. T2T genome assemblies can serve as a benchmark with enhanced accuracy and comprehensive genomic references for future studies, facilitating the confident identification and annotation of genes, regulatory elements, and other functional components.

A high-quality genome can support finer genetic analyses, such as the length, number, and distribution of key indicators of inbreeding, such as ROH and IBD [21, 22], and these analyses are more urgent than ever for the conservation of endangered species. In this study, we utilize PacBio HiFi, Nanopore and Hi-C data to generate improved telomere-to-telomere gap-free genomes of Yangtze and East Asian finless porpoises. We compare the quality of the newly drafted assemblies with previously available versions and explore synteny blocks, structural similarities, phylogenetic relationships, gene family expansion and inference of selection in relation to several other mammals. Finless porpoises serve as a representative example for comprehending speciation, evolution, and population genetics. The high-quality chromosomal-level references help elucidate ecological and aquatic adaptation mechanisms in cetaceans.

Results and Discussion

Genome sequencing and gap-free assembly

We integrated PacBio HiFi long reads, ultra-long ONT reads and Hi-C sequencing data to

generate chromosome-level genome assemblies for YFP and EFP. Henceforth, we refer to this new T2T genome as 2.0 and the original genome as 1.0. We generated approximately 123 Gb (49 ×) PacBio HiFi reads, 279 Gb (111 ×) Hi-C reads and 225 Gb (90 ×) ONT reads for YFP v2.0 (Supplementary Table S1). In this study, we supplemented the existing dataset comprising 62 × PacBio HiFi and 85 × Hi-C reads of the EFP [23] with an additional 215 Gb (86 ×) of ONT reads (Supplementary Table S1). The YFP v2.0 genome assembly comprised 23 scaffolds, with both contig N50 and scaffold N50 measuring 125.12 Mb (Table 1). These scaffolds were assembled into 21 autosomal chromosomes, one X chromosome, and one mitochondrial chromosome, resulting in a final assembly size of 2.48 Gb (Supplementary Table S2). Similarly, the EFP v2.0 genome assembly consisted of 24 scaffolds, with both contig N50 and scaffold N50 measuring 128.00 Mb (Table 1). These scaffolds were also assembled into 21 autosomal chromosomes, one X+Y chromosome, and one mitochondrial chromosome, with a final assembly size of 2.50 Gb (Supplementary Table S2).

We have significantly enhanced the contiguity, accuracy and completeness of the YFP v1.0 (GCA_003031525.2) and EFP v1.0 (GCA_026225855.1) assemblies. The contig N50 values of the two new genomes were consistent with their respective chromosome lengths, and a single contig represented a complete chromosome, which is notably superior to the recently published finless porpoise genomes (e.g., 125.12 Mb vs. 0.09 Mb for YFP and 128.00 Mb vs 84.69 Mb for EFP) (Table 1). The YFP v1.0 and EFP v1.0 genome assemblies had 52,647 and 28 gaps, respectively, whereas the new assembly process produced gap-free genomes, obviously improving contiguity (Figure 2A and Figure 2B). As of May 2024, the killer whale genome assembly (GCF_937001465.1) stands out among the 34 publicly available cetacean genomes sourced from the NCBI (National Center for Biotechnology Information) and CNGBdb (China National GeneBank DataBase) databases. It features an N50 length of 45.6 Mb and is composed of 570 contigs. In our study, the two newly assembled genomes exhibit a marked improvement in contiguity. The contig N50 length achieved in our assemblies is threefold that of the killer whale genome, with the number of contigs comprising only 4.03% of the latter. Moreover, the analysis of the blue whale genome (GCF_009873245.2) shows a contig N50 value that is only 4.0% of our assemblies, while its contig count is 42 times higher [24] (Supplementary Table S3). The comparative metrics highlight the superior continuity of the YFP and EFP genome assemblies in this study.

The quality values obtained from Merqury's k-mer analysis [25] for YFP v2.0 and EFP v2.0 were calculated as 60.18 and 64.38, respectively (Supplementary Table S4). These values indicated the superior quality of our assemblies. It is essential to emphasize that Merqury quality values span from 0 to 255, with higher values denoting superior quality. These values indicate a foundational accuracy level of 99.999%, confirming the high quality of our assembly for each component. Moreover, the mapping rates of RNA reads to the two genome assemblies were 95.47% and 95.42%, respectively, whereas they were 70.01% and 92.34% for previously published assemblies (Supplementary Table S5). Moreover, the mapping results of ONT long reads with a mapping quality >20 indicate a read coverage of 99.99% for YFP v2.0 and EFP v2.0 whole genomes (Supplementary Figure S1). Using the Benchmarking Universal Single-Copy Orthologs (BUSCO) evaluation, our results represent a completeness of 95.20% for YFP and 95.30% for EFP (Table 1 and Supplementary Figure S2). Additionally, we have identified/predicted both the telomeric repeat and centromere candidate region in YFP v2.0 and EFP v2.0 (Figure 2A, Figure 2B and Supplementary Table S6-S7). Telomeric repeat units of YFP v2.0 genome were detected at both ends of 18 chromosomes and at one end of 3 chromosomes. Similarly, telomeric repeat units of EFP v2.0 genome were detected at both ends of 20 chromosomes and at one end of 2 chromosomes. Telomeric repeat units were detected at 85% and 90% of the chromosome at both ends in YFP v2.0 and EFP v2.0 genomes, respectively. Our estimations of the centromere region are novel and were not present in YFP v1.0. Finally, we also utilized Hi-C data for chromosome sequencing and orientation, resulting in a comprehensive Hi-C matrix that effectively illustrates genome-wide interactions (Supplementary Figure S3). These findings suggest that the new genome assemblies of YFP and EFP represent a significant improvement over the previously published genome assembly, achieving near telomere-to-telomere (T2T) completeness.

Gene prediction and annotation

Two strategies including *de novo* and homolog-base methods were applied to annotate repeat elements. The genomes of YFP v2.0 and EFP v2.0 contained 1,058.09 Mb (42.54%) and 1,069.10 Mb (42.80%) of repetitive sequences, respectively (Supplementary Figure S4 and Table S8). Long interspersed nuclear elements (LINEs) were the most abundant type of annotated transposable elements, accounting for 38.88% and 39.10% of the genomes of YFP v2.0 and EFP v2.0, respectively (Supplementary Table S9). In total, the number of predicted protein-coding genes was

23,139 in the YFP v2.0 genome and 23,101 in the EFP v2.0 genome (Table 1). The roughly comparable number of predicted protein-coding genes for both T2T genomes is further evidence supporting the gene models (Supplementary Table S10-S13). It is worth noting that the length distribution of gene models at the levels of genes, CDS, exons and introns showed a similar trend when compared to those of YFP v1.0 (GCA_003031525.2), EFP v1.0 (GCA_026225855.1) and Bottlenose Dolphin (GCF_011762595) (Supplementary Figure S5). In the predicted gene models of YFP v2.0 and EFP v2.0, the BUSCO analysis identified 97.5 % and 97.6% complete conserved single copy mammalian genes (odb10), respectively (Table 1 and Supplementary Figure S2). A total of 22,263 (96.21%) gene models in the YFP v2.0 genome and 22,224 (96.20%) gene models in the EFP v2.0 genome were annotated in at least one database, including NR, SwissProt, KEGG, KOG, TrEMBL, Gene Ontology, and InterPro (Figure 1B and Table 1). Furthermore, 71.22% (16,480) of YFP v2.0 genes and 71.24% (16,457) of EFP v2.0 genes were annotated in five functional databases, namely NR, SwissProt, KEGG, KOG, and InterPro (Supplementary Figure S6 and Table S14). Finally, 20,589 (88.98%) and 20,613 (89.23%) genes from the YFP v2.0 and EFP v2.0 genomes, respectively, were determined to be transcriptionally active based on the analysis of 24 RNA-seq datasets (Figure 1B). In conclusion, our annotation of two gene sets of reliably high quality has established a robust foundation for further research.

Analysis of centromere related genes

The centromere plays a necessary role in cell division for eukaryotes. The intrinsic mechanisms underlying the evolution of centromere structure among different species has not been fully revealed due to the challenge of assembling highly repetitive sequences [26]. In this study, we conducted predictions on repeat monomers within the YFP v2.0 and EFP v2.0 genomes, potentially constituting the centromeres (Figure 2A, Figure 2B and Supplementary Table S15-S16). The monomeric sequences vary in length from 99 to 201 bp, with the 144 bp, 150 bp, and 138 bp monomers being the most abundant. Centromeres are composed of more than one repeat monomer and are located within TE- and TR-enriched regions, which are areas with relatively lower gene density (Supplementary Figure S7). A total of 235 and 237 genes were identified in the candidate centromere regions for YFP v2.0 and EFP v2.0, respectively, through predictions generated by centromere-finding software. Moreover, the newly discovered centromere regions for YFP v2.0 hosted approximately 56 genes, while only 20 genes were found in analogous regions for EFP v2.0.

The "newly identified centromere regions" refer to specific areas identified in the genome that have been recently assembled but were not included in the previously published version. This discovery may suggest the presence of novel, previously uncharacterized centromere sites. We further compared the genes located in the centromere and non-centromere regions in the genomes of YFP v2.0 and EFP v2.0, respectively, and found that there were no significant differences in the expression patterns of these genes in different transcripts (Supplementary Figure S8 and Supplementary Table S17-S18). Additionally, we analyzed the functional enrichment of genes located in the centromere regions of the two genomes. These genes were mainly associated with Keratin filament (GO:0045095), Intermediate filament (GO:0005882), Gamete generation (GO:0007276) and Channel activity (GO:0015267), as indicated by GO enrichment (Supplementary Table S19). These findings are consistent with results obtained through analysis using the KEGG database, highlighting the essential role of centromeres in the segregation and positioning of chromosomes. (Supplementary Figure S9 and Table S20-S21). Centromeres play a vital role in providing structural support to the nucleus, thereby ensuring the integrity of chromosomes, and are essential for the processes of mitosis and meiosis. Moreover, centromeric regions have the potential to engage with mechanisms related to cell cycle regulation, cell division signaling pathways, and channel activity, thereby impacting the progression of cell division [27].

Variations between YFP and EFP genomes

Synteny analysis comparing the gene order between YFP v2.0 and EFP v2.0 identified 907 significant shared syntenic blocks, covering 89.59% (41,428) of the genes, and revealed 17 chromosomal rearrangements (Figure 3). Further comparison of the genomic sequences of the YFP v2.0 and EFP v2.0 genomes, however, yielded numerous variations between the two, including 3,887,060 single-nucleotide polymorphisms (SNPs) (Figure 4A) and 704,944 short insertions/deletions (InDels) (Figure 4B). The SNPs exhibited predominant occurrences in intergenic regions (65.28%) and intronic regions (32.66%), with minimal presence in exon regions (0.64%). Similarly, the distribution of variations in InDels was less concentrated in exon regions, accounting for only 0.23% (Supplementary Table S22). Of the exon region variations, 12,203 SNPs (11,987 non-synonymous SNPs, 189 stop-gain SNPs, and 27 stop-loss SNPs) and 953 InDels (464 frameshift deletion, 467 frameshift insertion, 20 stop-gain InDels, and 2 stop-loss InDels) were identified and functionally associated with 6,158 and 582 genes, respectively. KEGG enrichment

analysis revealed that these genes were significantly (P value < =0.05) enriched in "NF-kappa B signaling pathway", "complement and coagulation cascades", "antigen processing and presentation" and "Intestinal immune network for IgA production" (Supplementary Figure S10 and Table S23).

The genes coding for the mutated regions of the YFP and EFP are widely enriched in immune-related pathways. This association may be intricately linked to the distinct pathogenic microorganisms unique to freshwater and seawater environments. Marine mammals exhibit a diminished histocompatibility complex (MHC) diversity attributed to decreased encounters with microparasitic diversity in their marine habitat relative to their terrestrial origin. This phenomenon implies that mammals encounter distinct pathogenic pressures in varied ecological settings, potentially influencing the evolution of immune-related genes [28]. Evolutionary analyses of the innate immune pattern recognition receptor (TLRs) in the YFP and the marine finless porpoise indicate that the YFP has undergone specific adaptive changes [29]. The microbial diversity and pathogenicity of freshwater and seawater environments vary, leading to distinct effects of pathogenic microorganisms on the organisms in these two types of environments [30]. Therefore, the YFP and EFP would be expected to undergo adaptive evolution to adapt to the pathogen stresses specific to their respective ecological environments, freshwater and seawater.

By comparing the assembly results of the YFP and EFP versions, it was found that the YFP v2.0 assembly included 5,480 new genes, while the EFP v2.0 assembly included 1,453 new genes compared to the previous version of the assembly (Table 1 and Supplementary Table S24). These genes were expressed in all 24 samples (Supplementary Figure S11 and Table S25-S26). GO and KEGG functional enrichment analysis indicated that these genes are primarily enriched in immunity and iron binding, among other functions. These biological processes encompass a wide range of aspects, including cellular structure and function, protein synthesis, signal transduction, immune response, and energy metabolism (Supplementary Table S27-S30).

Phylogeny analysis

Gene family analysis was performed on 506,098 protein-coding sequences derived from ten cetaceans and sixteen terrestrial mammalian species. The sequences were clustered into 22,196 gene families, which include 594 species-specific genes identified in YFP v2.0 and EFP v2.0 (Supplementary Table S31). Among the dataset, a total of 2,161 single-copy gene families were identified. Subsequently, multiple sequence alignments were conducted, which were then followed

by the reconstruction of evolutionary trees. The resulting phylogenetic tree produced a topology consistent with previous studies [31], highlighting its reliability and alignment with established scientific knowledge. This analysis notably revealed the topological arrangement within branches of mammals. The emergence of the EFP branch alongside its YFP counterpart was particularly noteworthy. (Supplementary Figure S12). Our analysis had revealed that the divergence time between the YFP and EFP ranges from 0.5 to 1.1 million years ago (Figure 5A, Figure 5B and Supplementary Figure S13), which is the first estimate at the molecular level since their classification as two distinct subspecies [14].

Gene family and positive selection analysis

We used CAFÉv4.0 to analyze the evolution of gene families based on orthologous clusters of protein coding sequences from twenty-six mammals. Upon comparing the genomes of YFP v2.0 and EFP v2.0 with their most recent common ancestor, it was observed that 843 gene families underwent expansion while 98 gene families experienced contraction (Figure 5A and Figure 5B). Among the 215 expanded gene families identified in the YFP v2.0 and EFP v2.0 lineage, a total of 2,674 genes were determined to be significantly associated ($P<0.05$) (Supplementary Table S32). We observed an expansion for genes significantly enriched in several KEGG pathways, including "antigen processing and presentation", "intestinal immune network for IgA production", "oxidative phosphorylation", and the "calcium signaling pathway" (Figure 6A). The significantly enriched GO terms, included "ferric iron binding", "iron ion transport", "riboflavin biosynthetic process", "tetrahydrofolate biosynthetic process" and "cytochrome-c oxidase activity", also expanded (Figure 6B).

Gene families associated with "oxidative phosphorylation", "cytochrome-c oxidase activity", "riboflavin biosynthetic process", "ferric iron binding" and "iron ion transport" exhibited expansion in finless porpoises. Iron ions play a crucial role in numerous essential physiological functions within living organisms, such as oxygen transportation, electron transport chains, and various metabolic pathways. Several sets of redox reactions are necessary to maintain effective gas exchange in water for cetaceans, and iron is often used in these reactions as an electron acceptor [32]. The expansion of redox reaction and iron ion gene families in cetaceans potentially improved the efficacy of oxygen utilization and facilitated adaptation to hypoxic conditions in aquatic habitats. An increase in the number of oxidation-reduction and iron-binding gene families was also observed

in *Lipotes vexillifer*, a species that encounters hypoxic conditions during dives [33]. The changes in pathogenic microorganisms that occur during the reintroduction of cetaceans from land to sea present a significant challenge to their survival and may have influenced the evolution and adaptation of immune genes [34]. The expansion of immune-related gene families in finless porpoises in this study enhanced the process of antigen presentation and conferred resistance against various pathogenic microorganisms amidst changes in their habitat. Genomic studies of the *S. chinensis* have revealed that cetaceans have developed various species-specific gene families related to immunity and DNA repair, which are linked to potential adaptive mechanisms [35]. We suggest these genes play a crucial role in facilitating hypoxic tolerance and enhancing immune resistance in finless porpoises, thereby reflecting potential mechanisms of adaptation to the aquatic environment. Further investigations are required to elucidate the specific functions of these gene families and their potential significance in the biology of finless porpoises.

The Codeml program in PAML with a branch-site model was employed to test for selective pressure based on orthologous clusters of 10 cetaceans, including *N. a. asiaeorientalis* (Yangtze finless porpoise; NCBI taxon ID:1706337), *N. a. sunameri* (East Asian finless porpoise; NCBI taxon ID:861190), *Tursiops truncatus* (Bottlenose dolphin; NCBI taxon ID:9739), *Orcinus orca* (Killer whale; NCBI taxon ID:9733), *L. vexillifer* (Yangtze River dolphin; NCBI taxon ID:118797), *Physeter catodon* (Sperm whale; NCBI taxon ID:9755), *Balaenoptera acutorostrata* (Minke whale; NCBI taxon ID:9767), *Balaena mysticetus* (Bowhead whale; NCBI taxon ID:27602), *Delphinapterus leucas* (Beluga whale; NCBI taxon ID:9749), *Sousa chinensis* (Indo-Pacific humpback dolphin; NCBI taxon ID:103600). We identified 41 positively selected genes (PSGs) in the YFP lineage, which were functionally enriched in “RNA degradation”, “nucleotide excision repair”, “DNA replication”, “mismatch repair”, and “homologous recombination” pathways (P<0.05) (Figure 6C and Supplementary Table S33). The selective pressure to evolve DNA damage repair pathways implied that the Yangtze finless porpoise might be experiencing increased threats to genome stability. The mechanism of DNA damage repair plays a crucial role in preserving genome integrity by enabling cells to identify and repair DNA damage, thereby averting the accumulation of harmful mutations [36]. In a comparative genomic analysis between the South China tiger and the Amur tiger, it was noted that genes related to DNA repair underwent positive selection in the South China tiger [21]. The observed phenomenon could be explained by the higher

probability of genome instability in the temperate and subtropical habitats of the South China tiger. This may be linked to metabolites generated by intestinal microflora, which possess the ability to trigger DNA damage [37]. The stability of the genome or gene expression system in the Yangtze finless porpoise across different organs and life stages remains uncertain. However, one potential interpretation of this data is the suggestion that the Yangtze finless porpoise could be vulnerable to genomic instability triggers in the Yangtze River, such as water pollutants, which may increase the likelihood of DNA damage [38]. Pollutants found in the Yangtze River possess the capacity to accumulate within the food chain, leading to cellular DNA damage and impacting the genome stability of the Yangtze finless porpoise [39, 40]. This makes a compelling case for the improved conservation of the species and the development of more rigorous water pollution mitigation practices.

Interestingly, a total of 44 PSGs within the EFP lineage were involved in "sodium-dependent phosphate transport", "sodium symporter activity", "aldosterone-regulated sodium reabsorption", and "calcium signaling pathway" (Figure 6D and Supplementary Table S34). These metabolic pathways play an important role in regulating sodium levels in the body [41]. Among these PSGs, six were potentially associated with the adaptation of EFP to high osmolarity environment, including the Na(+)/H(+) exchange regulatory cofactor *NHE-RF2* and sodium-dependent phosphate cotransporter *SLC34*. These genes identified are likely to have significant implications in the control of urine formation and the preservation of water-salt metabolic balance [41], suggesting that the East Asian finless porpoise may have a different urine formation process. Previous genome-selective sweep analyses in the finless porpoises revealed that the *SLC14A* in the East Asian finless porpoise underwent positive selection [14]. Comparative analyses conducted at the transcriptome level between the Yangtze finless porpoise and the East Asian finless porpoise revealed a notable upregulation of the *NHE3* in the kidneys of the East Asian finless porpoise. These results imply some adaptive enhanced osmoregulatory capability in the East Asian finless porpoise [42]. The aforementioned studies suggest that the East Asian finless porpoise has evolved a complex and efficient osmoregulatory mechanism as it acclimatized to the hypertonic marine environment, demonstrating adaptations at both molecular and transcriptional levels.

Methods

Sample collection, DNA extraction, and sequencing

We collected an adult dead female YFP sample from Lianzhou Lake, Anqing City, Anhui Province, China (N30°15′32″, E116°54′38″) in 2021 and a dead juvenile male EFP sample from the Yellow Sea near Lianyungang City, Jiangsu Province, China (N34°55′27″, E119°11′37″) in 2019 for sequencing (Figure 1A). The Office of Fishery Supervision and Management in the Yangtze River Basin, Ministry of Agriculture and Rural Affairs of the People's Republic of China has designated our research institution to perform post-mortem analysis and genetic preservation on deceased porpoises. No ethical considerations were taken into account in this study. DNA were extracted from muscle tissues following the phenol/chloroform DNA extraction method. DNA extracted from the YFP was utilized to construct PacBio HiFi, Hi-C, and Oxford Nanopore Technologies (ONT) libraries. DNA extracted from an EFP was utilized to construct an ONT library. The PacBio HiFi library was constructed using SMRTbell Prep Kit 3.0 (Pacific Biosciences, USA) and subsequently sequenced on the PacBio Sequel II system in circular consensus sequence (CCS) mode. To collect data for the Hi-C library, the muscle tissues were first fixed in 1% formaldehyde (Sigma) for cross-linking and resuspended in lysis buffer. Then, MboI (NEB) restriction endonucleases were used to fragment the chromatin in the muscle to fragment DNA. The DNA fragments were captured by utilizing Streptavidin-coated magnetic beads (Thermo Fisher SCIENTIFIC) following biotin labeling and crosslinking using T4 DNA Ligase (ENZYMATICS). The Hi-C library was finally sequenced on a BGI MGISEQ platform. To generate and sequence ONT libraries, we isolated genomic DNA using the CTAB [43] method, selected fragments exceeding 5 kb in size with the SageHLS HMW library system (Sage Science), processed the DNA with the Ligation sequencing 1D kit (SQK-LSK109, Oxford Nanopore Technologies, Oxford, UK), and subsequently sequenced the ONT libraries on a PromethION platform (Oxford Nanopore Technologies) at the BGI (Wuhan, China).

Gap-free genome assembly and quality assessment

We utilized SMRTLink v11.0.0 (https://www.pacb.com/support/software-downloads) to filter PacBio HiFi reads, applying the following criteria: a minimum requirement of three full-length subreads for generating CCS, a draft length threshold of 500 bp before polishing, and aiming for a predicted accuracy of 0.99. To filter Hi-C reads, index reads were removed and reads were filtered using the following SOAPNUKE v2.0 [44] parameters: N rate >= 0.01, low quality <= 20, low

quality rate >= 0.1. Subsequently, ONT reads were filtered based on a length < 5 kb and a quality value < 7. The Necat pipeline (v 20200119) [45] was utilized for the enhancement of ONT reads. This was achieved through the application of error correction algorithms to evaluate quality scores, k-mer frequencies, and alignment methods. This process enhanced the accuracy of reading and generated refined results suitable for further analyses. To achieve gap-free chromosome-level assemblies, we employed both the Hifiasm (v0.15.1) [46] and Necat pipeline (v20200119) [45] to separately assemble PacBio HiFi reads and ONT corrected reads into the initial contigs. The Purge-Haplotigs program [47] was utilized to eliminate redundant contigs that exhibited similar sequences but distinct haplotypes, specifically targeting those with aligned coverage below 30%. This strategic approach significantly optimizes the assembly process by removing redundant information, thus improving the accuracy of genome assembly. We utilized Hi-C data to cluster, order, and orient the contigs into pseudo-chromosomes though the implementation of the Juicer (v1.5) [48] and 3D-DNA (v180922) [49] pipelines. Ultra-long ONT reads and contigs were used to generate gapless scaffolds through the LR_Gapcloser (v1.0) [50] and TGSgapcloser (v 1.0.1) [51] pipelines.

Various metrics were used to evaluate the quality of the gap-free genome assemblies, including contiguity, accuracy, and completeness. First, we calculated the length metrics of genomic sequences to evaluate contiguity and subsequently used Merqury (v1.3) [25] with k-mer set to 21 to assess the accuracy. Second, Benchmarking Universal Single-Copy Orthologs (BUSCO) [52] evaluation was conducted to assess the completeness. Third, we also mapped PacBio HiFi, ONT and RNA-seq data into the genome assemblies using Minimap2 [53] and Hisat2 (v2.1.0) [54] to assess the completeness. In addition, we utilized the quartet pipeline [55] to search for telomere repeat sequences and centromere regions in YFP and EFP.

Genome annotation

Repetitive sequence annotation was identified using both *de novo* and homology-base approaches. For the *de novo* strategy, RepeatModeler (v1.0.4) [56] was employed to identify repetitive elements, whereas LTR-FINDER (v1.0.7) [57] was used for the specific annotation of long terminal repeats. For homolog-based prediction, RepeatMasker (v4.0.7) [58] was utilized to detect DNA transposable elements (TEs), while RepeatProteinMasker (v4.0.7) was employed to identify protein-based TEs, both based on the Repbase database (v21.12). Additionally, Tandem Repeat Finder (v4.10.0) [59] was used to identify Tandem repeats. The utilization of these tools facilitated a comprehensive

annotation of repetitive sequences, leading to a substantial improvement in the accuracy and detail of the analysis.

A combination of RNA-seq, homology-based and *de novo* prediction strategies was utilized to identify protein-coding genes in the genomes of YFP and EFP. RNA-seq data [15, 16] were mapped to genome assemblies with Hisat2 v2.1.0 [60] with the following parameters: --sensitive --no-discordant --no-mixed -I 1 -X 1000 --max-intronlen 1000000. The produced BAM alignments were further assembled into gene models with StringTie v1.3.5 [61] with the following parameters: -f 0.3 -j 3 -c 5 -g 100 -s 10000 and validated using PASA v2.5.2 [62]. The coding sequences were identified by TransDecoder (v5.5.0) (https://github.com/TransDecoder/TransDecoder) with default parameters. Utilizing 149,956 genes from 8 closely related cetacean species and transcriptomic sequencing data from 24 YFPs as input files, the GeMoMa v1.9 [63] software was employed to conduct homology-based prediction analysis (Supplementary Table S8, S35-S36). A set of one thousand high quality genes, which were predicted by the GeMoMa software and validated by OrthoDB for mammals, were randomly selected for training the predictors in Augustus v3.2.1 [64]. The Augustus v3.2.1 program was used to perform *de novo* prediction. We used GeMoMa software to integrate all predicted protein-coding genes, and annotated them with NR, Swissprot [65], KEGG [66], KOG, TrEMBL, InterPro [67] and GO [68] databases.

Genome comparison and identification of newly assembled genes

Single nucleotide polymorphisms (SNPs) and insertions/deletions (InDels) were identified using methods following those previously described [69]. Genome alignment was conducted utilizing the NUCmer program integrated within MUMmer4 (v4.0.0) [70] to compare the v2.0 assembly with the v1.0 assembly, as well as the YFP assembly with the EFP assembly. Utilizing the Maximum Unique Matches (MUM) mode involved setting parameters such as a minimum MUM length of 1000 bp, a minimum similarity threshold of 90%, and the exclusion of matches below 40 bp. Alignment blocks were identified using the delta-filter program, while SNPs and InDels were detected using the show-snps program and Syri (v1.6.3) [71], respectively. Functional annotation of SNPs and InDels is conducted using ANNOVAR [72] to assess their impacts on gene structure and function. Additionally, we utilize the CMplot R package [73] to visually depict the density distribution of these genetic variations. The objective of these analyses was to reveal structural variations between the v1.0 and v2.0 genome assemblies, as well as between the YFP and EFP

genome assemblies. Variants were meticulously annotated utilizing the ANNOVAR package (v 2013-06-21) [72].

Genes were categorized as newly assembled when the gene region in the initial draft genome assembly showed a deletion of at least 50 bp and had a minimum overlap of 30% within that specific region.

Gene family and phylogenomic analysis

Gene families of 26 species (Supplementary Table S31) were identified and clustered by OrthoFinder (v2.3.11) [74]. We utilized MAFFT (v7.310) and PhyML (v3.3) software tools to align single-copy orthologous genes (1:1:1) and construct a maximum-likelihood phylogenetic tree, respectively [75, 76]. The HKY85 model, known for its capacity to accommodate nucleotide substitution patterns, was applied in the tree construction procedure. To evaluate the reliability of the generated tree, 1000 bootstrap replications were performed, providing statistical evidence for the branching structures. Species divergence time was calculated using MCMCTREE in PAML (v4.9) [77]. Four divergence time points from TimeTree (http://timetree.org.cn) were used to calibrate the divergence times: (a) *Ornithorhynchus anatinus* (Platypus; NCBI taxon ID:9258) and *Monodelphis domestica* (Opossum; NCBI taxon ID:13616) (163.7–185.9 Ma), (b) *Homo sapiens* (Human; NCBI taxon ID:9606) and *Mus musculus* (Mouse; NCBI taxon ID:10090) (81.3–91.0 MYA), (c) *B. mysticetus* and *B. acutorostrata* (21.3–28.8 Ma) and (d) *S. chinensis* and *T. truncatus* (2.0–3.8 Ma). The core-orthologous gene sets were identified by BLAST (v2.0.14) [78] with an E-value threshold of $1{\times}E^{-10}$ (at least 10 syntenic genes allowed), and syntenic blocks were defined using MCscanX v1.5.2 [79]. Circos was used to plot the synteny results.

Gene family expansion and contraction analysis

Protein sequences of YFP, EFP and 24 published mammals were used to search homologs. Based on the gene families clustered by OrthoFinder, the CAFÉ (v4.0) [80] software was used to perform expansion and contraction analyses in the clade of finless porpoises. Random birth and death models were employed to study gains and losses of gene families in a user-specified phylogeny. The global parameter λ, which describes both the gene birth (λ) and death ($\mu = -\lambda$) rate for gene families in all branches of the tree, was estimated using maximum likelihood. Then the P-value was calculated for each gene family, and P-value ≤ 0.01 was defined as a “significantly expanded or contracted gene family”. KEGG and GO enrichment analyses were conducted among

these significantly expanded and contracted gene families.

Gene positive selection analysis

Protein sequences of two finless porpoise and another eight cetaceans were used to identify single copy orthologs with OrthoFinder. Then Ka/Ks ratios for these single copy orthologs were calculated by following steps. Initially, the single-copy orthologs underwent global alignment using PRANK. Subsequently, alignment refinement via Gblocks was utilized to remove inadequately aligned positions and divergent regions, thereby isolating conserved blocks from the multiple alignment. Codeml from the PAML package [77] was ultimately employed to compute Ka/Ks ratios across various branches, utilizing the free-ratio model. Genes that showed values of Ka/Ks higher than 1 along the branch leading to finless porpoise were reanalyzed using the codon-based branch site tests implemented in PAML (PAML, RRID:SCR_014932). The branch site model allowed ω to vary both among sites in the protein and across branches, and it was used to detect episodic positive selection.

Gene expression analysis

The raw RNA-seq reads underwent quality control using SOAPnuke (v2.0). Reads were filtered out if they had an N rate ≥ 0.01, low quality ≤ 20, low quality rate ≥ 0.1, or contained index sequences. Subsequently, the clean reads were aligned to the EFS v2.0 genome utilizing the Hisat2 high sensitivity model, excluding discordant pairs and mixed alignments. The alignment process involved setting a minimum insert size of 1 bp and a maximum insert size of 1000 bp. We utilized featureCounts [81] and transcripts per million (TPM) method to generate an estimated mapped read count matrix and calculate the gene expression level, respectively.

Conclusion

The availability of reliable chromosome-level genome assemblies provides a remarkable improvement in identifying genes, characterizing genomic regions and performing comparative genomic analyses. In the present study, we assembled telomere-to-telomere and gap-free Yangtze finless porpoise and the East Asian finless porpoise genomes by combining PacBio long reads, Hi-C and short-read sequencing technologies. The new assemblies have higher contiguity and completeness, as well as more complete single-copy BUSCO genes with fewer fragmented or missing genes than the first drafts. Compared to the 34 cetacean genomes sourced from public databases, the two genomes newly assembled in our study exhibit the longest contig N50 and scaffold N50, along with the lowest number of contigs, thus achieving superior continuity. The reconstructed phylogeny determined that YFP and EFP diverged approximately 0.5-1.1 million years ago (Ma). Gene family expansion analysis revealed significantly enriched pathways and GO terms associated with the regulation of immune resistance and hypoxic tolerance. Selection pressure analysis identified genes associated with DNA damage repair in the YFP and high salt tolerance in the EFP, respectively. Our results provide evidence of the gradual adaptation of EFP to a marine environment and the potential sensitivity of YFP to genome damage. Identification of the centromere, telomere, and associated genes can serve as valuable resources for a comprehensive understanding of chromosome stability, recombination, repair mechanisms, and evolutionary processes. Overall, these are the most continuous cetacean genome assemblies to date, with chromosome-scale contigs and no gaps. This study will lay a foundation for population genomics studies at the whole genome level, and deepen the scientific understanding of issues related to population conservation and adaptation mechanisms.

DATA AVAILABILITY

Raw sequencing data and genome assemblies in this study have been deposited in the NCBI database (BioProject ID PRJNA915046 and PRJNA859258). Furthermore, results of repeat annotation, gene structure annotation and gene functional annotation had been deposited in the *figshare* https://figshare.com/s/1fc632fd4f3cab36b776.

COMPETING INTERESTS

The authors declare that they have no competing interests.

AUTHORS' CONTRIBUTIONS

K.L., J.J. and P.X. designed and conceived the study. D.Y., C.Y. and J.Z. collected and prepared the samples. C.C. and C.Z. performed the data analysis. D.Y., C.C. and J.J. wrote the manuscript with significant contributions from Y.L., Z.C., H.Z., C.W. and L.L. K.L., Z.H. and D.L. provided the financial support. All authors read and approved the final version of the manuscript.

ACKNOWLEDGMENTS

This work was funded by the National Key R&D Program of China (2021YFD1200304), the Central Public-interest Scientific Institution Basal Research Fund, Freshwater Fisheries Research Center, CAFS (2021JBFM15) and Project of Implementation of Yangtze Finless Porpoise Protection in the Middle and Lower Reaches of Yangtze River (2021).

REFERENCES

1. Gao, A and Zhou, K. Growth and reproduction of three populations of finless porpoise, *Neophocaena phocaenoides*, in Chinese waters. *Aquat Mamm* 1993;**19** (1):3-12.
2. Jefferson, T. Preliminary analysis of geographic variation in cranial morphometrics of the finless porpoise (*Neophocaena phocaenoides*). *Raffles Bull Zool* 2002;**10**:3-14.
3. Wang, P. The morphological characters and the problem of subspecies identifications of the finless porpoise. *Fish Sci* 1992;**11**:4-8.
4. Gao, A and Zhou, K. Geographical variation of external measurements and three subspecies of *Neophocaena phocaenoides* in Chinese waters. *Acta Theriol Sin* 1995;**15**(2):81-92.
5. Wang, J, Frasier, T, Yang, S, *et al*. Detecting recent speciation events: the case of the finless porpoise (genus *Neophocaena*). *Heredity* 2008;**101**(2):145-55.
6. Jefferson, T and Wang, J. Revision of the taxonomy of finless porpoises (genus *Neophocaena*): The existence of two species. *J Mar Anim Ecol* 2011;**4**(1):3-16.
7. Yang, G, Ren, W, Zhou, K, *et al*. Population genetic structure of finless porpoises, *Neophocaena phocaenoides*, in Chinese waters, inferred from mitochondrial control region sequences. *Marine mammal science* 2002;**18**(2):336-47.
8. Xu, S, Sun, P, Zhou, K, *et al*. Sequence variability at three MHC loci of finless porpoises (*Neophocaena phocaenoides*). *Immunogenetics* 2007;**59**(7):581-92.
9. Chen, M, Zheng, J, Wu, M, *et al*. Genetic diversity and population structure of the critically endangered Yangtze finless porpoise (*Neophocaena asiaeorientalis asiaeorientalis*) as revealed by mitochondrial and microsatellite DNA. *Int J Mol Sci* 2014;**15**(7):11307-23.
10. Chen, M, Fontaine, M, Chehida Y, *et al*. Genetic footprint of population fragmentation and contemporary collapse in a freshwater cetacean. *Sci Rep* 2017;**7**(1):14449.
11. Lin, W, Frère, C, Karczmarski, L, *et al*. Phylogeography of the finless porpoise (genus *Neophocaena*): testing the stepwise divergence hypothesis in the northwestern Pacific. *Sci Rep* 2014;**4**:6572.
12. Li, S, Xu, S, Wan, H, *et al*. Genome-wide SNP and population divergence of finless porpoises. *Genome Biol Evol* 2013;**5**(4):758-68.
13. Zheng, J, Xia, J, He, S, *et al*. Population genetic structure of the Yangtze finless porpoise (*Neophocaena phocaenoides asiaeorientalis*): implications for management and conservation. *Biochem Genet* 2005;**43**(5-6):307-20.
14. Zhou, X, Guang, X, Sun, D, *et al*. Population genomics of finless porpoises reveal an incipient cetacean species adapted to freshwater. *Nat Commun* 2018;**9**(1):1276.
15. Yin, D, Lin, D, Guo, H, *et al*. Integrated analysis of blood mRNAs and microRNAs reveals immune changes with age in the Yangtze finless porpoise (*Neophocaena asiaeorientalis*). Comp *Biochem Physiol B Biochem Mol Biol* 2021;**256**:110635.
16. Liu, W, Yin, D, Lin, D, *et al*. Blood Transcriptome Analysis Reveals Gene Expression Differences between Yangtze Finless Porpoises from Two Habitats: Natural and Ex Situ Protected Waters. *fishes* 2022;**7**:96.
17. Nurk, S, Koren, S, Rhie, A, *et al*. The complete sequence of a human genome. *Science* 2022;**376**(6588):44-53.
18. Rhie A, Nurk S, Cechova M, *et al*. The complete sequence of a human Y chromosome. *Nature* 2023;**621**(7978):344-54.
19. Huang, Z, Xu, Z, Bai, H, *et al*. Evolutionary analysis of a complete chicken genome. *Proc Natl*

Acad Sci U S A 2023;**120**(8):e2216641120.
20. Xue, L, Gao, Y, Wu, M *et al*. Telomere-to-telomere assembly of a fish Y chromosome reveals the origin of a young sex chromosome pair. *Genome Biol* 2021;**22**(1):203.
21. Zhang, L, Lan, T, Lin, C, *et al*. Chromosome-scale genomes reveal genomic consequences of inbreeding in the South China tiger: A comparative study with the Amur tiger. *Mol Ecol Resour* 2023;**23**(2):330-47.
22. Shukla, H, Suryamohan, K, Khan, A, *et al*. Near-chromosomal de novo assembly of Bengal tiger genome reveals genetic hallmarks of apex predation. *Gigascience* 2022;**12**:giac112.
23. Yin, D, Chen, C, Lin, D, *et al*. Gapless genome assembly of East Asian finless porpoise. *Sci Data* 2022;**9**(1):765.
24. Bukhman, Y, Morin, P, Meyer, S, *et al*. A high-quality blue whale genome, segmental duplications, and historical demography. *Mol Biol Evol* 2024; **41**(3): msae036.
25. Rhie, A, Walenz, B, Koren, S, *et al*. Merqury: reference-free quality, completeness, and phasing assessment for genome assemblies. *Genome Biol* 2020;**21**(1):245.
26. Zhang, A, Kong, T, Sun, B, *et al*. A telomere-to-telomere genome assembly of Zhonghuang 13, a widely-grown soybean variety from the original center of Glycine max. *The Crop Journal* 2023. doi:https://doi.org/10.1016/j.cj.2023.10.003.
27. Brar, G, Amon, A. Emerging roles for centromeres in meiosis I chromosome segregation. *Nat Rev Genet* 2008;**9**(12):899-910.
28. Slade, R and McCallum, H. Overdominant vs. frequency-dependent selection at MHC loci. *Genetics* 1992;**132**(3):861–64.
29. Tian, R, Chen, M, Chai, S, *et al*. Divergent Selection of Pattern Recognition Receptors in Mammals with Different Ecological Characteristics. *J Mol Evol* 2018;**86**(2):138-49.
30. Lokesh, J and Kiron, V. Transition from freshwater to seawater reshapes the skin-associated microbiota of Atlantic salmon. *Sci Rep* 2016;**25**(6):19707.
31. Yuan, Y, Zhang, Y, Zhang, P, *et al*. Comparative genomics provides insights into the aquatic adaptations of mammals. *Proc Natl Acad Sci U S A* 2021;**118**(37):e2106080118.
32. Guo, B, Sun, Y, Wang, Y, *et al*. Evolutionary genetics of pulmonary anatomical adaptations in deep-diving cetaceans. *BMC Genomics* 2024;**25**(1):339.
33. Zhou, X, Sun, F, Xu, S, *et al*. Baiji genomes reveal low genetic variability and new insights into secondary aquatic adaptations. *Nat Commun* 2013;**4**:2708.
34. Li, L, Rong, X, Li, G, *et al*. Genomic organization and adaptive evolution of IGHC genes in marine mammals. *Mol Immunol* 2018;**99**:75-81.
35. Ming, Y, Jian, J, Yu, F, *et al*. Molecular footprints of inshore aquatic adaptation in Indo-Pacific humpback dolphin (*Sousa chinensis*). *Genomics* 2019;**111**(5):1034-42.
36. Chatterjee, N and Walker, G. Mechanisms of DNA damage, repair, and mutagenesis. *Environ Mol Mutagen* 2017;**58**(5):235-63.
37. Puschhof, J and Sears, C. Microbial metabolites damage DNA. *Science* 2022;**378**(6618):358-59.
38. Lv, W, Gu, H, He, D, *et al*. Polystyrene nanospheres-induced hepatotoxicity in swamp eel (*Monopterus albus*): From biochemical, pathological and transcriptomic perspectives. *Sci Total Environ* 2023;**893**:164844.
39. Zhang, K, Qian, Z, Ruan, Y, *et al*. First evaluation of legacy persistent organic pollutant contamination status of stranded Yangtze finless porpoises along the Yangtze River Basin,

China. *Sci Total Environ* 2020;**710**:136446.

40. Xiong, X, Qian, Z, Mei, Z, *et al*. Trace elements accumulation in the Yangtze finless porpoise (*Neophocaena asiaeorientalis asiaeorientalis*)-A threat to the endangered freshwater cetacean. *Sci Total Environ* 2019;**686**:797-804.
41. Shoemaker, V and Nagy, K. Osmoregulation in amphibians and reptiles. *Annu Rev Physiol*. 1977;**39**: 449-71.
42. Ruan, R, Guo, A, Hao, Y, *et al*. De novo assembly and characterization of narrow-ridged finless porpoise renal transcriptome and identification of candidate genes involved in osmoregulation. *Int J Mol Sci* 2015;**16**(1):2220-38.
43. Yan, M, Wei, G, Pan, X *et al*. A method suitable for extracting genomic DNA from animal and plant-modified CTAB method. *Agric Sci Technol*, 2008, **9**(2):39-41.
44. Chen Y, Chen Y, Shi C, *et al*. SOAPnuke: a MapReduce acceleration-supported software for integrated quality control and preprocessing of high-throughput sequencing data. *Gigascience* 2018;**7**(1):1-6.
45. Chen Y, Nie F, Xie S, *et al*. Efficient assembly of nanopore reads via highly accurate and intact error correction. *Nat Commun* 2021;**12**(1):60.
46. Cheng, H, Concepcion, G, Feng, X, *et al*. Haplotype-resolved de novo assembly using phased assembly graphs with hifiasm. *Nat Methods* 2021;**18**(2):170-5.
47. Roach, M, Schmidt, S and Borneman, A. Purge Haplotigs: allelic contig reassignment for third-gen diploid genome assemblies. *BMC bioinformatics* 2018;**19**(1):460.
48. Durand, N, Shamim, M, Machol, I, *et al*. Juicer Provides a One-Click System for Analyzing Loop-Resolution Hi-C Experiments. *Cell systems* 2016;**3**(1):95-8.
49. Dudchenko, O, Batra, S, Omer, A, *et al*. De novo assembly of the Aedes aegypti genome using Hi-C yields chromosome-length scaffolds. *Science* 2017;**356**(6333):92-5.
50. Xu, G, Xu, T, Zhu, R, *et al*. LR_Gapcloser: a tiling path-based gap closer that uses long reads to complete genome assembly. *Gigascience* 2019;**8**(1):giy157.
51. Xu M, Guo L, Gu S, *et al*. TGS-GapCloser: A fast and accurate gap closer for large genomes with low coverage of error-prone long reads. *Gigascience* 2020;**9**(9):giaa094.
52. Waterhouse, R, Seppey, M, Simão, F, *et al*. BUSCO Applications from Quality Assessments to Gene Prediction and Phylogenomics. *Mol Biol Evol* 2018;**35**(3):543-8.
53. Li, H. Minimap2: pairwise alignment for nucleotide sequences. *Bioinformatics* 2018;**34** (18):3094-100.
54. Kim, D, Paggi, J, Park, C, *et al*. Graph-based genome alignment and genotyping with HISAT2 and HISAT-genotype. *Nat Biotechnol* 2019;**37**(8):907-15.
55. Lin, Y, Ye, C, Li, X, *et al*. quarTeT: a telomere-to-telomere toolkit for gap-free genome assembly and centromeric repeat identification. *Hortic Res* 2023;**10**(8):uhad127.
56. Chen N. Using RepeatMasker to identify repetitive elements in genomic sequences. *Curr Protoc Bioinformatics* 2004;**Chapter 4**:Unit 4.10.
57. Xu, Z and Wang, H. LTR_FINDER: an efficient tool for the prediction of full-length LTR retrotransposons. *Nucleic Acids Res* 2007;**35**:W265-8.
58. Price, A, Jones, N and Pevzner, P. De novo identification of repeat families in large genomes. *Bioinformatics* 2005;**21**(Suppl 1):i351-8.
59. Benson, G. Tandem repeats finder: a program to analyze DNA sequences. *Nucleic Acids Res* 1999;**27**(2):573-80.

60. Kim, D, Langmead, B and Salzberg, S. HISAT: a fast spliced aligner with low memory requirements. *Nat Methods* 2015;**12**(4):357-60.
61. Kovaka, S, Zimin, A, Pertea, G, *et al*. Transcriptome assembly from long-read RNA-seq alignments with StringTie2. *Genome Biol* 2019;**20**(1):278.
62. Haas, B, Salzberg, S, Zhu W, *et al*. Automated eukaryotic gene structure annotation using EVidenceModeler and the Program to Assemble Spliced Alignments. *Genome Biol* 2008;**9**(1):R7.
63. Keilwagen, J, Hartung, F and Grau, J. GeMoMa: Homology-Based Gene Prediction Utilizing Intron Position Conservation and RNA-seq Data. *Methods Mol Biol* 2019;**1962**:161-77.
64. Stanke, M and Waack, S. Gene prediction with a hidden Markov model and a new intron submodel. *Bioinformatics* 2003;**19**(Suppl 2):ii215-25.
65. Bairoch, A and Apweiler, R. The SWISS-PROT protein sequence data bank and its supplement TrEMBL. *Nucleic Acids Res* 1997;**25**(1):31-6.
66. Kanehisa, M, Sato, Y, Kawashima, M, *et al*. KEGG as a reference resource for gene and protein annotation. *Nucleic Acids Res* 2016;**44**(D1):D457-62.
67. Jones, P, Binns, D, Chang, H, *et al*. InterProScan 5: genome-scale protein function classification. *Bioinformatics* 2014;**30**(9):1236-40.
68. Ashburner, M, Ball, CA, Blake, J, *et al*. Gene ontology: tool for the unification of biology. The Gene Ontology Consortium. *Nat Genet* 2000;**25**(1):25-9.
69. Li, T, Xu, X, Zhao, J, *et al*. Genome assembly of KA105, a new resource for maize molecular breeding and genomic research. *The Crop Journal* 2023;**11**(6):1793-1804.
70. Marçais, G, Delcher, A, Phillippy, A, *et al*. MUMmer4: A fast and versatile genome alignment system. *PLoS Comput Biol* 2018;**14**(1):e1005944.
71. Goel, M, Sun, H, Jiao, W, *et al*. SyRI: finding genomic rearrangements and local sequence differences from whole-genome assemblies. *Genome Biol* 2019;**20**(1):277.
72. Wang, K, Li, M and Hakonarson, H. ANNOVAR: functional annotation of genetic variants from high-throughput sequencing data. *Nucleic Acids Res* 2010;**38**(16):e164.
73. Yin, L, Zhang, H, Tang, Z, *et al*. rMVP: A Memory-efficient, Visualization-enhanced, and Parallel-accelerated tool for Genome-Wide Association Study. *Genomics, Proteomics & Bioinformatics* 2021;**19**(4):619-28.
74. Emms, D and Kelly, S. OrthoFinder: phylogenetic orthology inference for comparative genomics. *Genome Biol* 2019;**20**(1):238.
75. Nakamura, T, Yamada, K, Tomii, K, *et al*. Parallelization of MAFFT for large-scale multiple sequence alignments. *Bioinformatics* 2018;**34**(14):2490-2.
76. Guindon, S, Delsuc, F, Dufayard, J, *et al*. Estimating maximum likelihood phylogenies with PhyML. *Methods Mol Biol* 2009;**537**:113-37.
77. Yang, Z. PAML 4: phylogenetic analysis by maximum likelihood. *Mol Biol Evol* 2007;**24** (8):1586-91.
78. Altschul, S, Gish, W, Miller, W, *et al*. Basic local alignment search tool. *J Mol Biol* 1990;**215**(3):403-10.
79. Wang, Y, Tang, H, Debarry, J, *et al*. MCScanX: a toolkit for detection and evolutionary analysis of gene synteny and collinearity. *Nucleic Acids Res* 2012;**40**(7):e49.
80. Bie, T, Cristianini, N, Demuth, J, *et al*. CAFE: a computational tool for the study of gene family evolution. *Bioinformatics* 2006;**22**(10):1269-71.

81. Liao, Y, Smyth, G and Shi, W. featureCounts: an efficient general purpose program for assigning sequence reads to genomic features. *Bioinformatics* 2014;**30**(7):923-30.

Tables

Table 1. Genome assembly statistics of YFP and EFP.

	**YFP v2.0**	**YFP v1.0**	**EFP v2.0**	**EFP v1.0**
Total size of assembled genome (Gb)	2.48	2.27	2.50	2.50
Contig N50 (Mb)	125.12	0.09	128.00	84.69
Contig N90 (Mb)	83.62	0.02	80.21	29.54
Number of contigs	22	66,345	23	51
Scaffold N50 (Mb)	125.12	6.34	128.00	122.40
Scaffold N90 (Mb)	83.62	1.13	80.21	80.21
Scaffolds number	22	13,698	23	23
Number of base chromosomes	22	22	23	23
Number of gap-free chromosomes	22	0	23	7
Number of gaps	0	52,647	0	28
Number of telomeres (pairs/single)	20/2	0/0	21/2	21/2
Number of estimated centromeres	22	0	23	23
TE size	42.54%	NA	42.80%	42.23%
GC content	41.70%	41.00%	41.70%	41.70%
BUSCO* (Genome)	95.2%	94.0%	95.3%	95.4%
Gene Number	23,139	18,479	23,101	22,814
New-found gene number	5,480	NA	1,453	NA
Functional proteins	96.21%	NA	96.20%	97.31%
BUSCO* (Protein)	97.5%	94.6%	97.6%	97.9%
Data source	This study (PRJNA915046)	GCF_003031525.2	This study (PRJNA859258)	GCA_026225855.1

Note: The term "v2.0" denotes the updated genome assembly and annotation produced in the present study, while "v1.0" refers to the initial draft of the genome assembly and annotation that was previously published. "BUSCO*" indicated the percentage of complete BUSCO evaluation. "pairs/single": "pairs" indicated that the telomeres were found at both ends of the chromosomes; "single" indicated that the telomeres were found only at the one end of chromosomes. "New-found genes" indicated that the genes were predicted in the extra sequence segments from the current assembly and were annotated in the current assembly.

Figure legends

Figure 1. Sample site and Genome assessment of YFP v2.0 and EFP v2.0.

A: Location distribution and sampling site of the YFP and EFP.

B: Proportions of genes that could be functionally annotated and transcriptionally detected in YFP v2.0 and EFP v2.0.

Figure 2. T2T-resolved assembly of YPF v2.0 and EFP v2.0 and functional enrichment of genes in the centromere region.

Structure of T2T and gap-free chromosomes in A) YFP v2.0, and B) EFP v2.0. All 21+X/Y chromosomes are drawn to scale and the ruler indicates chromosome length. Triangles indicate the presence of telomere sequence repeats. Circles represent the locations of centromeric regions. The gap positions in the v1.0 genome assemblies are marked with squares to the right of the chromosome in the v2.0 genome assemblies.

Figure 3. Synteny analysis of YFP v2.0 and EFP v2.0 genomes: A) chromosomes scale, Unit length is Mb; B) syntenic blocks between YFP v2.0 and EFP v2.0.

Figure 4. Structure variant between YFP v2.0 and EFP v2.0 genome assembly with YFP v2.0 genome assembly for reference.

The density plot of A) SNPs and B) InDels between YFP v2.0 and EFP v2.0 genome assembly. The numerical values adjacent to the color legend indicate the count of SNPs/InDels per megabase (MB) window, where the gray legend representing zero. Each color corresponds to a specific range of values. For instance, in Figure 4A, the initial blue legend represents the range from 1 to 4440.

Figure 5. Genome evolution of YFP v2.0 and EFP v2.0.

A: Divergence time between YFP v2.0 and EFP v2.0, and number of expanded and contracted gene families. Green and red numbers indicate gene family expansions and contractions, respectively. MRCA: Most Recent Common Ancestor. Ma: Million years ago.

B: A comparison of gene families associated with orthologs and paralogs in YFP v2.0 and EFP v2.0, and other 24 mammal species.

Figure 6. Functional Enrichment of genes.

Significant A) KEGG and B) GO enrichment of expanded gene families in YFP and EFP lineage.

KEGG enrichment analysis of positively selected genes in C) YFP and D) EFP, respectively.

Revised manuscript (marked)

Click here to access/download;Manuscript;Revised manuscript (marked).docx

Formatted: Numbering: Continuous

Telomere-to-telomere gap-free genome assembly of the endangered Yangtze finless porpoise and East Asian finless porpoise

Denghua Yin[1†], Chunhai Chen[2†], Danqing Lin[1†], Zhong Hua[1], Congping Ying[3], Jialu Zhang[1], Chenxi Zhao[2], Yan Liu[1], Zhichen Cao[4], Han Zhang[4], Chenhe Wang[2], Liping Liang[2], Pao Xu[1,3], Jianbo Jian[2,5,*] and Kai Liu[1,3,4,*]

[1]Key Laboratory of Freshwater Fisheries and Germplasm Resources Utilization, Ministry of Agriculture and Rural Affairs, Freshwater Fisheries Research Center, Chinese Academy of Fishery Sciences, Wuxi 214081, China.

[2]BGI Genomics, ~~BGI Shenzhen,~~ Shenzhen 518083, China.

[3]Wuxi Fisheries College, Nanjing Agricultural University, Wuxi 214081, China.

[4]National Demonstration Center for Experimental Fisheries Science Education, Shanghai Ocean University, Shanghai 201306, China.

[5]Guangdong Provincial Key Laboratory of Marine Biotechnology, Shantou University, Shantou 515063, China.

[†]Authors contributed equally to this work.

[*]Correspondence address: E-mail: jianjianbo@bgi.com, liuk@ffrc.cn.

Abstract

The Yangtze finless porpoise (*Neophocaena asiaeorientalis asiaeorientalis*, YFP) and the East Asian finless porpoise (*Neophocaena asiaeorientalis sunameri*, EFP) are two subspecies of the narrow-ridged finless porpoise that live in fresh and salt water, respectively. They serve as ideal models for the study of freshwater adaptation and evolution. The main objective of this study was to provide contiguous chromosome-level genome assemblies for YFP and EFP. Here, we generated and upgraded the genomes of YFP and EFP at the telomere-to-telomere level through the integration of PacBio HiFi long reads, ultra-long ONT reads and Hi-C sequencing data. The total sizes of the genomes are 2.48 Gb and 2.50 Gb, respectively. The scaffold N50 of two genomes was 125.12 Mb (YFP) and 128 Mb (EFP) with one contig for one chromosome. The telomere repeat and centromere position were clearly identified in both YFP and EFP genomes. Telomeric repeat units were detected at 85% and 90% of the chromosome at both ends in YFP and EFP genomes, respectively. In total, 5,480 new-found genes were detected in the YFP genome, including 56 genes located in the newly identified centromere regions. Additionally, synteny blocks, ~~comparative genomes~~structural similarities, phylogenetic relationships, gene family expansion and inference of selection were studied in connection with the genomes of other related mammals. Significant improvements were observed in genome contiguity, the number of scaffolds and gaps, and annotation compared to the first drafts of the YFP (GCA_003031525.2) and EFP (GCA_026225855.1) genome assemblies. The improvement of telomere-to-telomere gap-free reference genome resources support conservation genetics and population management for finless porpoises.

Keywords: telomere-to-telomere; genome assembly; Yangtze finless porpoise; gap-free; HiFi sequencing; Hi-C sequencing

Introduction

Finless porpoises (*Neophocaena* spp.) are small toothed whales capable of inhabiting freshwater (Yangtze River) and saltwater (coastal waters of southern and eastern Asia) environments [1, 2]. They are characterized by a blunt, rounded head, an equal width upper and lower jaw, and lack of an obvious dorsal fin [3, 4]. Based on morphological characteristics, geographic distribution and molecular genetic evidence, it is generally believed that the finless porpoise can be divided into two species, namely the Indo-Pacific finless porpoise (*N. phocaenoides*) and the narrow-ridged finless porpoise (*N. asiaeorientalis*) [5, 6]. In China, there exist two subspecies of the narrow-ridged finless porpoise: one is the freshwater Yangtze finless porpoise (*N. a. asiaeorientalis*, YFP), which exclusively inhabits the middle and lower reaches of the Yangtze River and adjacent Dongting and Poyang lakes. The other subspecies is the marine East Asian finless porpoise (*N. a. sunameri*, EFP), which can be found in the coastal waters of the Yellow Sea and Bohai Sea, and in the northern waters of the East China Sea [5, 6] (Figure 1A).

The investigation of the evolutionary origins and conservation genetics of finless porpoises is a pressing concern for scientists. Yang *et al*. identified significant genetic structure between either the Yangtze River population or the Yellow Sea population and the South China Sea population by analyzing the sequences of mtDNA control region of finless porpoises in Chinese waters [7]. This result was supported by subsequent mtDNA sequences, nuclear DNA microsatellites, single nucleotide polymorphisms (SNPs) and MHC loci [8-12]. The genetic diversity of East Asian finless porpoises surpasses that of the other two populations, indicating it as the likely center of origin for this species [7]. Zheng *et al*. analyzed the sequences of the mtDNA control region of seven local populations of Yangtze finless porpoises in the middle and lower reaches of the Yangtze River, and found that the overall level of genetic diversity was low. Notably, the downstream population showed richer genetic variation than the midstream population. Such a genetic pattern reflects, to some extent, the marine origin and evolutionary history of the Yangtze population [13]. Based on genomic analysis of finless porpoise populations, significant genetic structure was identified among the three populations, indicating local adaptive evolution and emphasizing the evolutionary distinctiveness and conservation significance of the Yangtze finless porpoise [14].

Availability of a high-quality genome assembly is not only critical for the genomic studies of

finless porpoises, but also would be a valuable resource for comparative genomics and evolutionary studies of cetaceans. The first draft of the YFP genome assembly (GCF_003031525.2) was published in 2018 with a size of 2.3 Gb generated by short-read sequencing on the Illumina HiSeq 2000 platform [14]. The assembly comprised 13,698 scaffolds, with a scaffold N50 of 6.3 Mb, excluding the minimum sequence length (100 bp) consideration. Although progress had been made in genome-wide studies of YFP through the availability of this draft, such as immune changes with age and gene expression profiles in different habitats [15, 16], the lack of chromosomal information limits the potential application of the data. Recent advances in ultra-long ONT and PacBio HiFi sequencing technologies, as well as assembly algorithms, have facilitated the development of telomere-to-telomere (T2T) genome assemblies. The completion of the T2T human genome sequence and the full Y chromosome sequence represents a significant milestone in the field of human genomics research [17, 18]. The T2T genome has emerged as a hotspot genomic research, demonstrating extensive applications to other animal species like chicken and fish [19, 20]. T2T genome assemblies can serve as a benchmark with enhanced accuracy and comprehensive genomic references for future studies, facilitating the confident identification and annotation of genes, regulatory elements, and other functional components.

A high-quality genome can support finer genetic analyses, such as the length, number, and distribution of key indicators of inbreeding, such as ROH and IBD [21, 22], and these analyses are more urgent than ever for the conservation of endangered species. In this study, we utilize PacBio HiFi, Nanopore and Hi-C data to generate improved telomere-to-telomere gap-free genomes of Yangtze and East Asian finless porpoises. We compare the quality of the newly drafted assemblies with previously available versions and explore ~~the~~ synteny blocks, ~~comparative genomes~~structural similarities, phylogenetic relationships, gene family expansion and inference of selection in relation to ~~the~~ several other mammals. Finless porpoises serve as a representative example for comprehending speciation, evolution, and population genetics. The high-quality chromosomal-level references help elucidate ecological and aquatic adaptation mechanisms in cetaceans.

Results and Discussion

Genome sequencing and gap-free assembly

We integrated PacBio HiFi long reads, ultra-long ONT reads and Hi-C sequencing data to

generate chromosome-level genome assemblies for YFP and EFP. Henceforth, we refer to this new T2T genome as 2.0 and the original genome as 1.0. We generated approximately 123 Gb (49 ×) PacBio HiFi reads, 279 Gb (111 ×) Hi-C reads and 225 Gb (90 ×) ONT reads for YFP v2.0 (Supplementary Table S1). In this study, we supplemented the existing dataset comprising 62 × PacBio HiFi and 85 × Hi-C reads of the EFP [23] with an additional 215 Gb (86 ×) of ONT reads (Supplementary Table S1). The YFP v2.0 genome assembly comprised 23 scaffolds, with both contig N50 and scaffold N50 measuring 125.12 Mb (Table 1). These scaffolds were assembled into 21 autosomal chromosomes, one X chromosome, and one mitochondrial chromosome, resulting in a final assembly size of 2.48 Gb (Supplementary Table S2). Similarly, the EFP v2.0 genome assembly consisted of 24 ~~contigs or~~ scaffolds, with both contig N50 and scaffold N50 measuring 128.00 Mb (Table 1). These scaffolds were also assembled into 21 autosomal chromosomes, one X+Y chromosome, and one mitochondrial chromosome, with a final assembly size of 2.50 Gb (Supplementary Table S2).

We have significantly enhanced the contiguity, accuracy and completeness of the YFP v1.0 (GCA_003031525.2) and EFP v1.0 (GCA_026225855.1) assemblies. The contig N50 values of the two new genomes were consistent with their respective chromosome lengths, and a single contig represented a complete chromosome, which is notably superior to the recently published finless porpoise genomes (e.g., 125.12 Mb vs. 0.09 Mb for YFP and 128.00 Mb vs 84.69 Mb for EFP) (Table 1). The YFP v1.0 and EFP v1.0 genome assemblies had 52,647 and 28 gaps, respectively, whereas the new assembly process produced ~~a~~ gap-free genomes, obviously improving contiguity (Figure 2A and Figure 2B). As of May 2024, the killer whale genome assembly (GCF_937001465.1) stands out among the 34 publicly available cetacean genomes sourced from the NCBI (National Center for Biotechnology Information) and CNGBdb (China National GeneBank DataBase) databases. It features an N50 length of 45.6 Mb and is composed of 570 contigs. In our study, the two newly assembled genomes exhibit a marked improvement in contiguity. The contig N50 length achieved in our assemblies is threefold that of the killer whale genome, with the number of contigs comprising only 4.03% of the latter. Moreover, the analysis of the blue whale genome (GCF_009873245.2) shows a contig N50 value that is only 4.0% of our assemblies, while its contig count is 42 times higher [24] (Supplementary Table S3). The comparative metrics highlight the superior continuity of the YFP and EFP genome assemblies in this study.

The quality values obtained from Merqury's k-mer analysis [25~~24~~] for YFP v2.0 and EFP v2.0 were calculated as 60.18 and 64.38, respectively~~.~~ (Supplementary Table S~~3~~4). These values indicated the superior quality of our assemblies. It is essential to emphasize that Merqury quality values span from 0 to 255, with higher values denoting superior quality. These values indicate a foundational accuracy level of 99.999%, confirming the high quality of our assembly for each component. Moreover, the mapping rates of RNA reads to the two genome assemblies were 95.47% and 95.42%, respectively, whereas they were 70.01% and 92.34% for previously published assemblies (Supplementary Table ~~S4~~S5). Moreover, the mapping results of ONT long reads with a mapping quality >20 indicate a read coverage of 99.99% for YFP v2.0 and EFP v2.0 whole genomes (Supplementary Figure S1). Using the Benchmarking Universal Single-Copy Orthologs (BUSCO) evaluation, ~~it is suggested~~ our results represent a completeness of 95.20% ~~completeness of~~ for YFP and 95.30% ~~completeness of~~for EFP (Table 1 and Supplementary Figure S2). Additionally, we have identified/predicted both the telomeric repeat and centromere candidate region in YFP v2.0 and EFP v2.0 (Figure 2A, Figure 2B and Supplementary Table ~~S5~~S6-~~S6~~S7). Telomeric repeat units of YFP v2.0 genome were detected at both ends of 18 chromosomes and at one end of 3 chromosomes. Similarly, telomeric repeat units of EFP v2.0 genome were detected at both ends of 20 chromosomes and at one end of 2 chromosomes. Telomeric repeat units were detected at 85% and 90% of the chromosome at both ends in YFP v2.0 and EFP v2.0 genomes, respectively. Our estimations of the centromere region are novel and were not present in YFP v1.0. Finally, we also utilized Hi-C data for chromosome sequencing and orientation, resulting in a comprehensive Hi-C matrix that effectively illustrates genome-wide interactions (Supplementary Figure S3). These findings suggest that the new genome assemblies of YFP and EFP represent a significant improvement over the previously published genome assembly, achieving near telomere-to-telomere (T2T) completeness.

Gene prediction and annotation

Two strategies including *de novo* and homolog-base methods were applied to annotate repeat elements. The genomes of YFP v2.0 and EFP v2.0 contained 1,058.09 Mb (42.54%) and 1,069.10 Mb (42.80%) of repetitive sequences, respectively (Supplementary Figure S4 and Table ~~S7~~S8). Long interspersed nuclear elements (LINEs) were the most abundant type of annotated transposable elements, accounting for 38.88% and 39.10% of the genomes of YFP v2.0 and EFP v2.0, respectively (Supplementary Table ~~S8~~S9). In total, the number of predicted protein-coding genes

was 23,139 in the YFP v2.0 genome and 23,101 in the EFP v2.0 genome (Table 1). The roughly comparable number of predicted protein-coding genes for both T2T genomes is further evidence supporting the gene models (Supplementary Table ~~S9~~S10-~~S12~~S13). It is worth noting that the length distribution of gene models at the levels of genes, CDS, exons and introns showed a similar trend when compared to those of YFP v1.0 (GCA_003031525.2), EFP v1.0 (GCA_026225855.1) and Bottlenose Dolphin (GCF_011762595) (Supplementary Figure S5). In the predicted gene models of YFP v2.0 and EFP v2.0, the BUSCO analysis identified 97.5 % and 97.6% complete conserved single copy mammalian genes (odb10), respectively (Table 1 and Supplementary Figure S2). ~~In~~ A total~~,~~ of 22,263 (96.21%) gene models in the YFP v2.0 genome and 22,224 (96.20%) gene models in the EFP v2.0 genome were annotated in at least one database, ~~(~~including NR, SwissProt, KEGG, KOG, TrEMBL, Gene Ontology, and InterPro~~)~~ (Figure 1B and Table 1)~~,~~. ~~whereas~~ Furthermore, 71.22% (16,480) of YFP v2.0 genes and 71.24% (16,457) of EFP v2.0 genes ~~are~~ were annotated in five functional databases, ~~(~~namely NR, SwissProt, KEGG, KOG, and InterPro~~)~~ (Supplementary Figure S6 and Table ~~S13~~S14). Finally, 20,589 (88.98%) and 20,613 (89.23%) genes from the YFP v2.0 and EFP v2.0 genomes, respectively, were determined to be transcriptionally active based on the analysis of 24 RNA-seq datasets (Figure 1B). In conclusion, our annotation of two gene sets of reliably high quality has established a robust foundation for further research.

Analysis of centromere related genes

The centromere plays a necessary role in cell division for eukaryotes. The intrinsic mechanisms underlying the evolution of centromere structure among different species has not been fully revealed due to the challenge of assembling highly repetitive sequences [26~~25~~]. In this study, we conducted predictions on repeat monomers within the YFP v2.0 and EFP v2.0 genomes, potentially constituting the centromeres (Figure 2A, Figure 2B and Supplementary Table ~~S14~~S15-~~S15~~S16). The monomeric sequences vary in length from 99 to 201 bp, with the 144~~-~~ bp, 150~~-~~ bp, and 138~~-~~ bp monomers being the most abundant. Centromeres are composed of more than one repeat monomer and are located within TE- and TR-enriched regions, which are areas with relatively lower gene density (Supplementary Figure S7). A total of 235 and 237 genes were identified in the candidate centromere regions for YFP v2.0 and EFP v2.0, respectively, through predictions generated by centromere-finding software. Moreover, the newly discovered centromere regions for YFP v2.0 hosted approximately 56 genes, while only 20 genes were found in analogous regions for EFP v2.0.

The "newly identified centromere regions" refer to specific areas identified in the genome that have been recently assembled but were not included in the previously published version. This discovery may suggest the presence of novel, previously uncharacterized centromere sites. We further compared the genes located in the centromere and non-centromere regions in the genomes of YFP v2.0 and EFP v2.0, respectively, and found that there were no significant differences in the expression patterns of these genes in different transcripts (Supplementary Figure S8 and Supplementary Table ~~S16~~S17~~-S17~~S18). Additionally, we analyzed the functional enrichment of genes located in the centromere regions of the two genomes. These genes were mainly associated with Keratin filament (GO:0045095), Intermediate filament (GO:0005882), Gamete generation (GO:0007276) and Channel activity (GO:0015267), as indicated by GO enrichment (Supplementary Table ~~S18~~S19). These findings ~~align~~ are consistent with~~ those produced by~~ results obtained through analysis using the KEGG database, ~~and~~ highlighting the essential role of centromeres in the ~~chromosome~~ segregation and positioning of chromosomes (Supplementary Figure S9 and Table ~~S19~~S20~~-S20~~S21). Centromeres play a vital role in provid~~e~~ing structural support to the nucleus, thereby ensuring the integrity of chromosome~~s integrity~~, and are ~~crucial~~ essential for the~~in~~ processes of mitosis and meiosis. Moreover, centromeric regions ~~may interact with~~have the potential to engage with mechanisms related to cell cycle regulation, cell division signaling pathways, and channel activity, thereby impacting the progression of cell division ~~progression~~ [27].

Variations between YFP and EFP genomes

Synteny analysis ~~of~~ comparing the gene order between YFP v2.0 and EFP v2.0 ~~revealed~~ identified 907 ~~large~~ significant shared syntenic blocks, ~~encompassing~~ covering 89.59% (41,428) of the genes, and revealed 17 chromosomal rearrangements (Figure 3). Further comparison of the genomic sequences of the YFP v2.0 and EFP v2.0 genomes, however, yielded numerous variations between the two, including 3,887,060 single-nucleotide polymorphisms (SNPs) (Figure 4A) and 704,944 short insertions/deletions (InDels) (Figure 4B). The ~~variations in~~ SNPs ~~were primarily located in~~exhibited predominant occurrences in~~ the~~ intergenic regions (65.28%) and intronic regions (32.66%), ~~and rarely~~with minimal presence in ~~the~~ exon regions (0.64%). Similarly, the distribution of variations in InDels ~~were~~ was less concentrated in exon regions, accounting for only ~~(~~0.23%~~)~~ (Supplementary Table ~~S21~~S22). Of the exon region variations, 12,203 SNPs (11,987 non-synonymous SNPs, 189 stop-gain SNPs, and 27 stop-loss SNPs) and 953 InDels (464 frameshift

deletion, 467 frameshift insertion, 20 stop-gain InDels, and 2 stop-loss InDels) were identified and functionally associated with 6,158 and 582 genes, respectively. KEGG enrichment analysis revealed that these genes were significantly (P value < =0.05) enriched in "NF-kappa B signaling pathway", "complement and coagulation cascades", "antigen processing and presentation" and "Intestinal immune network for IgA production" (Supplementary Figure S10 and Table ~~S22~~S23).

The genes coding for the mutated regions of the YFP and EFP are widely enriched in immune-related pathways. This association may be intricately linked to the distinct pathogenic microorganisms unique to freshwater and seawater environments. Marine mammals exhibit a diminished histocompatibility complex (MHC) diversity attributed to decreased encounters with microparasitic diversity in their marine habitat relative to their terrestrial origin. This phenomenon implies that mammals encounter distinct pathogenic pressures in varied ecological settings, potentially influencing the evolution of immune-related genes [28~~26~~]. Evolutionary analyses of the innate immune pattern recognition receptor (TLRs) in the YFP and the marine finless porpoise indicate that the YFP has undergone specific adaptive changes [29~~27~~]. The microbial diversity and pathogenicity of freshwater and seawater environments vary, leading to distinct effects of pathogenic microorganisms on the organisms in these two types of environments [30~~28~~]. Therefore, the YFP and EFP would be expected to undergo adaptive evolution to adapt to the pathogen stresses specific to their respective ecological environments, freshwater and seawater.

By comparing the assembly results of the YFP and EFP versions, it was found that the YFP v2.0 assembly included 5,480 new genes, while the EFP v2.0 assembly included 1,453 new genes compared to the previous version of the assembly (Table 1 and Supplementary Table ~~S23~~S24). These genes were expressed in all 24 samples (Supplementary Figure S11 and Table ~~S24~~S25-~~S25~~S26). GO and KEGG functional enrichment analysis indicated that these genes are primarily enriched in immunity and iron binding, among other functions. These biological processes encompass a wide range of aspects, including cellular structure and function, protein synthesis, signal transduction, immune response, and energy metabolism (Supplementary Table ~~S26~~S27-~~S29~~S30).

Phylogeny analysis

Gene family analysis was performed on 506,098 protein-coding sequences derived from ten cetaceans and sixteen terrestrial mammalian species. The sequences were clustered into 22,196 gene

families, which include 594 species-specific genes identified in YFP v2.0 and EFP v2.0 (Supplementary Table S30S31). Among the dataset, a total of 2,161 single-copy gene families were identified. Subsequently, multiple sequence alignments were conducted, which were then followed by the reconstruction of evolutionary trees. The resulting phylogenetic tree produced a topology consistent with previous studies [3129], highlighting its reliability and alignment with established scientific knowledge. This analysis notably revealed the topological arrangement within branches of mammals. The emergence of the EFP branch alongside its YFP counterpart was particularly noteworthy. (Supplementary Figure S12). Our analysis had revealed that the divergence time between the YFP and EFP ranges from 0.5 to 1.1 million years ago (Figure 5A, Figure 5B and Supplementary Figure S13), which is the first estimate at the molecular level since their classification as two distinct subspecies [14].

Gene family and positive selection analysis

We used CAFÉv4.0 to analyze the evolution of gene families based on orthologous clusters of protein coding sequences from twenty-six mammals. Upon comparing the genomes of YFP v2.0 and EFP v2.0 with their most recent common ancestor, it was observed that 843 gene families underwent expansion while 98 gene families experienced contraction (Figure 5A and Figure 5B). Among the 215 expanded gene families identified in the YFP v2.0 and EFP v2.0 lineage, a total of 2,674 genes were determined to be significantly associated ($P<0.05$) (Supplementary Table S31S32). We observed an expansion for genes significantly enriched in several KEGG pathways, including “antigen processing and presentation”, “intestinal immune network for IgA production”, “oxidative phosphorylation”, and the “calcium signaling pathway” (Figure 6A). The significantly enriched GO terms, included “ferric iron binding”, “iron ion transport”, “riboflavin biosynthetic process”, “tetrahydrofolate biosynthetic process” and “cytochrome-c oxidase activity”, also expanded (Figure 6B).

Gene families associated with “oxidative phosphorylation”, “cytochrome-c oxidase activity”, “riboflavin biosynthetic process”, “ferric iron binding” and “iron ion transport” exhibited expansion in finless porpoises. Iron ions play a crucial role in numerous essential physiological functions within living organisms, such as oxygen transportation, electron transport chains, and various metabolic pathways. Several sets of redox reactions are necessary to maintain effective gas exchange in water for cetaceans, and iron is often used in these reactions as an electron acceptor

[32~~30~~]. The expansion of redox reaction and iron ion gene families in cetaceans potentially improved the efficacy of oxygen utilization and facilitated adaptation to hypoxic conditions in aquatic habitats. An increase in the number of oxidation-reduction and iron-binding gene families was also observed in *Lipotes vexillifer*~~*L. vexillifer*~~, a species that encounters hypoxic conditions during dives [33~~31~~]. The changes in pathogenic microorganisms that occur during the reintroduction of cetaceans from land to sea present a significant challenge to their survival and may have influenced the evolution and adaptation of immune genes [34~~32~~]. The expansion of immune-related gene families in finless porpoises in this study enhanced the process of antigen presentation and conferred resistance against various pathogenic microorganisms amidst changes in their habitat. Genomic studies of the *S. chinensis* have revealed that cetaceans have developed various species-specific gene families related to immunity and DNA repair, which are linked to potential adaptive mechanisms [35~~33~~]. We suggest these genes play a crucial role in facilitating hypoxic tolerance and enhancing immune resistance in finless porpoises, thereby reflecting potential mechanisms of adaptation to the aquatic environment. Further investigations are required to elucidate the specific functions of these gene families and their potential significance in the biology of finless porpoises.

The Codeml program in PAML with a branch-site model was employed to test for selective pressure based on orthologous clusters of 10 cetaceans, including ~~*Neophocaena*~~ *N.* ~~*asiaeorientalis*~~ *a. asiaeorientalis* (Yangtze finless porpoise; NCBI taxon ID:1706337), ~~*Neophocaena*~~ *N.* ~~*asiaeorientalis*~~ *a. sunameri* (East Asian finless porpoise; NCBI taxon ID:861190), *Tursiops truncatus* (Bottlenose dolphin; NCBI taxon ID:9739), *Orcinus orca* (Killer whale; NCBI taxon ID:9733), ~~*Lipotes*~~ *L. vexillifer* (Yangtze River dolphin; NCBI taxon ID:118797), *Physeter catodon* (Sperm whale; NCBI taxon ID:9755), *Balaenoptera acutorostrata* (Minke whale; NCBI taxon ID:9767), *Balaena mysticetus* (Bowhead whale; NCBI taxon ID:27602), *Delphinapterus leucas* (Beluga whale; NCBI taxon ID:9749), *Sousa chinensis* (Indo-Pacific humpback dolphin; NCBI taxon ID:103600)~~.~~. We identified 41 positively selected genes (PSGs) in the YFP lineage, which were functionally enriched in "RNA degradation", "nucleotide excision repair", "DNA replication", "mismatch repair", and "homologous recombination" pathways ($P<0.05$) (Figure 6C and Supplementary Table ~~S32~~S33). The selective pressure to evolve DNA damage repair pathways implied that the Yangtze finless porpoise might be experiencing increased threats to genome stability. The mechanism of DNA damage repair plays a crucial role in preserving genome integrity by

enabling cells to identify and repair DNA damage, thereby averting the accumulation of harmful mutations [36~~34~~]. In a comparative genomic analysis between the South China tiger and the Amur tiger, it was noted that genes related to DNA repair underwent positive selection in the South China tiger [21]. The observed phenomenon could be explained by the higher probability of genome instability in the temperate and subtropical habitats of the South China tiger. This may be linked to metabolites generated by intestinal microflora, which possess the ability to trigger DNA damage [37~~35~~]. The stability of the genome or gene expression system in the Yangtze finless porpoise across different organs and life stages remains uncertain. However, one potential interpretation of this data is the suggestion that the Yangtze finless porpoise could be vulnerable to genomic instability triggers in the Yangtze River, such as water pollutants, which may increase the likelihood of DNA damage [38~~36~~]. Pollutants found in the Yangtze River possess the capacity to accumulate within the food chain, leading to cellular DNA damage and impacting the genome stability of the Yangtze finless porpoise [39~~37~~, 40~~38~~]. This makes a compelling case for the improved conservation of the species and the development of more rigorous water pollution mitigation practices.

Interestingly, a total of 44 PSGs within the EFP lineage were involved in "sodium-dependent phosphate transport", "sodium symporter activity", "aldosterone-regulated sodium reabsorption", and "calcium signaling pathway" (Figure 6D and Supplementary Table ~~S33~~S34). These metabolic pathways play an important role in regulating sodium levels in the body [41~~39~~]. Among these PSGs, six were potentially associated with the adaptation of EFP to high osmolarity environment, including the Na(+)/H(+) exchange regulatory cofactor *NHE-RF2* and sodium-dependent phosphate cotransporter *SLC34*. These genes identified are likely to have significant implications in the control of urine formation and the preservation of water-salt metabolic balance [41~~39~~], suggesting that the East Asian finless porpoise may have a different urine formation process. Previous genome-selective sweep analyses in the finless porpoises revealed that the *SLC14A* in the East Asian finless porpoise underwent positive selection [14]. Comparative analyses conducted at the transcriptome level between the Yangtze finless porpoise and the East Asian finless porpoise revealed a notable upregulation of the *NHE3* in the kidneys of the East Asian finless porpoise. These results imply some adaptive enhanced osmoregulatory capability in the East Asian finless porpoise [42~~40~~]. The aforementioned studies suggest that the East Asian finless porpoise has evolved a complex and efficient osmoregulatory mechanism as it acclimatized to the hypertonic marine environment,

demonstrating adaptations at both molecular and transcriptional levels.

Methods

Sample collection, DNA extraction, and sequencing

We collected an adult dead female YFP sample from Lianzhou Lake, Anqing City, Anhui Province, China (N30°15′32″, E116°54′38″) in 2021 and a dead juvenile male EFP sample from the Yellow Sea near Lianyungang City, Jiangsu Province, China (N34°55′27″, E119°11′37″) in 2019 for sequencing (Figure 1A). The Office of Fishery Supervision and Management in the Yangtze River Basin, Ministry of Agriculture and Rural Affairs of the People's Republic of China has designated our research institution to perform post-mortem analysis and genetic preservation on deceased porpoises. No ethical considerations were taken into account in this study. DNA were extracted from muscle tissues following the phenol/chloroform DNA extraction method. DNA extracted from the YFP was utilized to construct PacBio HiFi, Hi-C, and Oxford Nanopore Technologies (ONT) libraries. DNA extracted from an EFP was utilized to construct an ONT library. The PacBio HiFi library was constructed using SMRTbell Prep Kit 3.0 (Pacific Biosciences, USA) and subsequently sequenced on the PacBio Sequel II system in circular consensus sequence (CCS) mode. To collect data for the Hi-C library, the muscle tissues were first fixed in 1% formaldehyde (Sigma) for cross-linking and resuspended in lysis buffer. Then, MboI (NEB) restriction endonucleases were used to fragment the chromatin in the muscle to fragment DNA. The DNA fragments were captured by utilizing Streptavidin-coated magnetic beads (Thermo Fisher SCIENTIFIC) following biotin labeling and crosslinking using T4 DNA Ligase (ENZYMATICS). The Hi-C library was finally sequenced on a BGI MGISEQ platform. To generate and sequence ONT libraries, we isolated genomic DNA using the CTAB [43~~41~~] method, selected fragments exceeding 5 kb in size with the SageHLS HMW library system (Sage Science), processed the DNA with the Ligation sequencing 1D kit (SQK-LSK109, Oxford Nanopore Technologies, Oxford, UK), and subsequently sequenced the ONT libraries on a PromethION platform (Oxford Nanopore Technologies) at the BGI (Wuhan, China).

Gap-free genome assembly and quality assessment

We utilized SMRTLink v11.0.0 (https://www.pacb.com/support/software-downloads) to filter PacBio HiFi reads, applying the following criteria: a minimum requirement of three full-length

subreads for generating CCS, a draft length threshold of 500 bp before polishing, and aiming for a predicted accuracy of 0.99. To filter Hi-C reads, index reads were removed and reads were filtered using the following SOAPNUKE v2.0 [44~~42~~] parameters: N rate >= 0.01, low quality <= 20, low quality rate >= 0.1. Subsequently, ONT reads were filtered based on a length < 5 kb and a quality value < 7. The Necat pipeline (v 20200119) [45~~43~~] was utilized for the enhancement of ONT reads. This was achieved through the application of error correction algorithms to evaluate quality scores, k-mer frequencies, and alignment methods. This process enhanced the accuracy of reading and generated refined results suitable for further analyses. To achieve gap-free chromosome-level assemblies, we employed both the Hifiasm (v0.15.1) [46~~44~~] and Necat pipeline (v20200119) [45~~43~~] to separately assemble PacBio HiFi reads and ONT corrected reads into the initial contigs. The Purge-Haplotigs program [47~~45~~] was utilized to eliminate redundant contigs that exhibited similar sequences but distinct haplotypes, specifically targeting those with aligned coverage below 30%. This strategic approach significantly optimizes the assembly process by removing redundant information, thus improving the accuracy of genome assembly. We utilized Hi-C data to cluster, order, and orient the contigs into pseudo-chromosomes though the implementation of the Juicer (v1.5) [48~~46~~] and 3D-DNA (v180922) [49~~47~~] pipelines. Ultra-long ONT reads and contigs were used to generate gapless scaffolds through the LR_Gapcloser (v1.0) [50~~48~~] and TGSgapcloser (v 1.0.1) [51~~49~~] pipelines.

Various metrics were used to evaluate the quality of the gap-free genome assemblies, including contiguity, accuracy, and completeness. First, we calculated the length metrics of genomic sequences to evaluate contiguity and subsequently used Merqury (v1.3) [25~~24~~] with k-mer set to 21 to assess the accuracy. Second, Benchmarking Universal Single-Copy Orthologs (BUSCO) [52~~50~~] evaluation was conducted to assess the completeness. Third, we also mapped PacBio HiFi, ONT and RNA-seq data into the genome assemblies using Minimap2 [53~~51~~] and Hisat2 (v2.1.0) [54~~52~~] to assess the completeness. In addition, we utilized the quartet pipeline [55~~53~~] to search for telomere repeat sequences and centromere regions in YFP and EFP.

Genome annotation

Repetitive sequence annotation was identified using both *de novo* and homology-base approaches. For the *de novo* strategy, RepeatModeler (v1.0.4) [56~~54~~] was employed to identify repetitive elements, whereas LTR-FINDER (v1.0.7) [57~~55~~] was used for the specific annotation of long

terminal repeats. For homolog-based prediction, RepeatMasker (v4.0.7) [58~~56~~] was utilized to detect DNA transposable elements (TEs), while RepeatProteinMasker (v4.0.7) was employed to identify protein-based TEs, both based on the Repbase database (v21.12). Additionally, Tandem Repeat Finder (v4.10.0) [59~~57~~] was used to identify Tandem repeats. The utilization of these tools facilitated a comprehensive annotation of repetitive sequences, leading to a substantial improvement in the accuracy and detail of the analysis.

A combination of RNA-seq, homology-based and *de novo* prediction strategies was utilized to identify protein-coding genes in the genomes of YFP and EFP. RNA-seq data [15, 16] were mapped to genome assemblies with Hisat2 v2.1.0 [60~~58~~] with the following parameters: --sensitive --no-discordant --no-mixed -I 1 -X 1000 --max-intronlen 1000000. The produced BAM alignments were further assembled into gene models with StringTie v1.3.5 [61~~59~~] with the following parameters: -f 0.3 -j 3 -c 5 -g 100 -s 10000 and validated using PASA v2.5.2 [62~~60~~]. The coding sequences were identified by TransDecoder (v5.5.0) (https://github.com/TransDecoder/TransDecoder) with default parameters. Utilizing 149,956 genes from 8 closely related cetacean species and transcriptomic sequencing data from 24 YFPs as input files, the GeMoMa v1.9 [63~~61~~] software was employed to conduct homology-based prediction analysis (Supplementary Table ~~S7~~S8, ~~S34~~S35~~-S35~~S36). A set of one thousand high quality genes, which were predicted by the GeMoMa software and validated by OrthoDB for mammals, were randomly selected for training the predictors in Augustus v3.2.1 [64~~62~~]. The Augustus v3.2.1 program was used to perform *de novo* prediction. We used GeMoMa software to integrate all predicted protein-coding genes, and annotated them with NR, Swissprot [65~~63~~], KEGG [66~~64~~], KOG, TrEMBL, InterPro [67~~65~~] and GO [68~~66~~] databases.

Genome comparison and ~~Identification~~ identification of newly assembled genes

Single nucleotide polymorphisms (SNPs) and insertions/deletions (InDels) were identified using methods following those previously described [69~~67~~]. Genome alignment was conducted utilizing the NUCmer program integrated within MUMmer4 (v4.0.0) [70~~68~~] to compare the v2.0 assembly with the v1.0 assembly, as well as the YFP assembly with the EFP assembly. Utilizing the Maximum Unique Matches (MUM) mode involved setting parameters such as a minimum MUM length of 1000 bp, a minimum similarity threshold of 90%, and the exclusion of matches below 40 bp. Alignment blocks were identified using the delta-filter program, while SNPs and InDels were detected using the show-snps program and Syri (v1.6.3) [71~~69~~], respectively. Functional annotation

of SNPs and InDels is conducted using ANNOVAR [72~~70~~] to assess their impacts on gene structure and function. Additionally, we utilize the CMplot R package [73~~71~~] to visually depict the density distribution of these genetic variations. The objective of these analyses was to reveal structural variations between the v1.0 and v2.0 genome assemblies, as well as between the YFP and EFP genome assemblies. Variants were meticulously annotated utilizing the ANNOVAR package (v 2013-06-21) [72~~70~~].

Genes were categorized as newly assembled when the gene region in the initial draft genome assembly showed a deletion of at least 50 bp and had a minimum overlap of 30% within that specific region.

Gene family and phylogenomic analysis

Gene families of 26 species (Supplementary Table ~~S30~~S31) were identified and clustered by OrthoFinder (v2.3.11) [74~~72~~]. We utilized MAFFT (v7.310) and PhyML (v3.3) software tools to align single-copy orthologous genes (1:1:1) and construct a maximum-likelihood phylogenetic tree, respectively~~Single-copy orthologous genes (1:1:1) were aligned using MAFFT~~ [75~~73~~, ~~] (v7.310), a widely utilized tool for multiple sequence alignment. Subsequently, a maximum-likelihood phylogenetic tree was generated using PhyML (v3.3) [~~76~~74~~]~~., a commonly employed software tool for phylogenetic analysis.~~ The HKY85 model, known for its capacity to accommodate nucleotide substitution patterns, was applied in the tree construction procedure. To evaluate the reliability of the generated tree, 1000 bootstrap replications were performed, providing statistical evidence for the branching structures. Species divergence time was calculated using MCMCTREE in PAML (v4.9) [77~~75~~]. Four divergence time points from TimeTree (http://timetree.org.cn) were used to calibrate the divergence times: (a) *Ornithorhynchus anatinus* (Platypus; NCBI taxon ID:9258) and *Monodelphis domestica* (Opossum; NCBI taxon ID:13616) (163.7–185.9 Ma), (b) *Homo sapiens* (Human; NCBI taxon ID:9606) and *Mus musculus* (Mouse; NCBI taxon ID:10090) (81.3–91.0 MYA), (c) ~~*Balaena*~~ *B. mysticetus* and ~~*Balaenoptera*~~ *B. acutorostrata* (21.3–28.8 Ma) and (d) ~~*Sousa*~~ *S. chinensis* and ~~*Tursiops*~~ *T. truncatus* (2.0–3.8 Ma). The core-orthologous gene sets were identified by BLAST (v2.0.14) [78~~76~~] with an E-value threshold of $1\times E^{-10}$ (at least 10 syntenic genes allowed), and syntenic blocks were defined using MCscanX v1.5.2 [79~~77~~]. Circos was used to plot the synteny results.

Gene family expansion and contraction analysis

Protein sequences of YFP, EFP and 24 published mammals were used to search homologs. Based on the gene families clustered by OrthoFinder, the CAFÉ (v4.0) [80~~78~~] software was used to perform expansion and contraction analyses in the clade of finless porpoises. Random birth and death models were employed to study gains and losses of gene families in a user-specified phylogeny. The global parameter λ, which describes both the gene birth (λ) and death (μ = −λ) rate for gene families in all branches of the tree, was estimated using maximum likelihood. Then the P-value was calculated for each gene family, and P-value ≤0.01 was defined as a "significantly expanded or contracted gene family". KEGG and GO enrichment analyses were conducted among these significantly expanded and contracted gene families.

Gene positive selection analysis

Protein sequences of two finless porpoise and another eight cetaceans were used to identify single copy orthologs with OrthoFinder. Then Ka/Ks ratios for these single copy orthologs were calculated by following steps. Initially, the single-copy orthologs underwent global alignment using PRANK. Subsequently, alignment refinement via Gblocks was utilized to remove inadequately aligned positions and divergent regions, thereby isolating conserved blocks from the multiple alignment. Codeml from the PAML package [77~~75~~] was ultimately employed to compute Ka/Ks ratios across various branches, utilizing the free-ratio model. Genes that showed values of Ka/Ks higher than 1 along the branch leading to finless porpoise were reanalyzed using the codon-based branch site tests implemented in PAML (PAML, RRID:SCR_014932). The branch site model allowed ω to vary both among sites in the protein and across branches, and it was used to detect episodic positive selection.

Gene expression analysis

The raw RNA-seq reads underwent quality control using SOAPnuke (v2.0). Reads were filtered out if they had an N rate ≥ 0.01, low quality ≤ 20, low quality rate ≥ 0.1, or contained index sequences. Subsequently, the clean reads were aligned to the EFS v2.0 genome utilizing the Hisat2 high sensitivity model, excluding discordant pairs and mixed alignments. The alignment process involved setting a minimum insert size of 1 bp and a maximum insert size of 1000 bp. We utilized featureCounts [81~~79~~] and transcripts per million (TPM) method to generate an estimated mapped read count matrix and calculate the gene expression level, respectively.

Conclusion

The availability of reliable chromosome-level genome assemblies provides a remarkable improvement in identifying genes, characterizing genomic regions and performing comparative genomic analyses. In the present study, we assembled telomere-to-telomere and gap-free Yangtze finless porpoise and the East Asian finless porpoise genomes by combining PacBio long reads, Hi-C and short-read sequencing technologies. The new assemblies have higher contiguity and completeness, as well as more complete single-copy BUSCO genes with fewer fragmented or missing genes than the first drafts. Compared to the 34 cetacean genomes sourced from public databases, the two genomes newly assembled in our study exhibit the longest contig N50 and scaffold N50, along with the lowest number of contigs, thus achieving superior continuity. The reconstructed phylogeny determined that YFP and EFP diverged approximately 0.5-1.1 million years ago (Ma). Gene family expansion analysis revealed significantly enriched pathways and GO terms associated with the regulation of immune resistance and hypoxic tolerance. Selection pressure analysis identified genes associated with DNA damage repair in the YFP and high salt tolerance in the EFP, respectively. Our results provide evidence of the gradual adaptation of EFP to a marine environment and the potential sensitivity of YFP to genome damage. Identification of the centromere, telomere, and associated genes can serve as valuable resources for a comprehensive understanding of chromosome stability, recombination, repair mechanisms, and evolutionary processes. Overall, these are the most continuous cetacean genome assemblies to date, with chromosome-scale contigs and no gaps. This study will lay a foundation for population genomics studies at the whole genome level, and deepen the scientific understanding of issues related to population conservation and adaptation mechanisms.

DATA AVAILABILITY

Raw sequencing data and genome assemblies in this study have been deposited in the NCBI database (BioProject ID PRJNA915046 and PRJNA859258). Furthermore, results of repeat annotation, gene structure annotation and gene functional annotation had been deposited in the *figshare* https://figshare.com/s/1fc632fd4f3cab36b776.

COMPETING INTERESTS

The authors declare that they have no competing interests.

AUTHORS' CONTRIBUTIONS

K.L., J.J. and P.X. designed and conceived the study. D.Y., C.Y. and J.Z. collected and prepared the samples. C.C. and C.Z. performed the data analysis. D.Y., C.C. and J.J. wrote the manuscript with significant contributions from Y.L., Z.C., H.Z., C.W. and L.L. K.L., Z.H. and D.L. provided the financial support. All authors read and approved the final version of the manuscript.

ACKNOWLEDGMENTS

This work was funded by the National Key R&D Program of China (2021YFD1200304), the Central Public-interest Scientific Institution Basal Research Fund, Freshwater Fisheries Research Center, CAFS (2021JBFM15) and Project of Implementation of Yangtze Finless Porpoise Protection in the Middle and Lower Reaches of Yangtze River (2021).

REFERENCES

1. Gao, A and Zhou, K. Growth and reproduction of three populations of finless porpoise, *Neophocaena phocaenoides*, in Chinese waters. *Aquat Mamm* 1993;**19** (1):3-12.
2. Jefferson, T. Preliminary analysis of geographic variation in cranial morphometrics of the finless porpoise (*Neophocaena phocaenoides*). *Raffles Bull Zool* 2002;**10**:3-14.
3. Wang, P. The morphological characters and the problem of subspecies identifications of the finless porpoise. *Fish Sci* 1992;**11**:4-8.
4. Gao, A and Zhou, K. Geographical variation of external measurements and three subspecies of *Neophocaena phocaenoides* in Chinese waters. *Acta Theriol Sin* 1995;**15**(2):81-92.
5. Wang, J, Frasier, T, Yang, S, *et al*. Detecting recent speciation events: the case of the finless porpoise (genus *Neophocaena*). *Heredity* 2008;**101**(2):145-55.
6. Jefferson, T and Wang, J. Revision of the taxonomy of finless porpoises (genus *Neophocaena*): The existence of two species. *J Mar Anim Ecol* 2011;**4**(1):3-16.
7. Yang, G, Ren, W, Zhou, K, *et al*. Population genetic structure of finless porpoises, *Neophocaena phocaenoides*, in Chinese waters, inferred from mitochondrial control region sequences. *Marine mammal science* 2002;**18**(2):336-47.
8. Xu, S, Sun, P, Zhou, K, *et al*. Sequence variability at three MHC loci of finless porpoises (*Neophocaena phocaenoides*). *Immunogenetics* 2007;**59**(7):581-92.
9. Chen, M, Zheng, J, Wu, M, *et al*. Genetic diversity and population structure of the critically endangered Yangtze finless porpoise (*Neophocaena asiaeorientalis asiaeorientalis*) as revealed by mitochondrial and microsatellite DNA. *Int J Mol Sci* 2014;**15**(7):11307-23.
10. Chen, M, Fontaine, M, Chehida Y, *et al*. Genetic footprint of population fragmentation and contemporary collapse in a freshwater cetacean. *Sci Rep* 2017;**7**(1):14449.
11. Lin, W, Frère, C, Karczmarski, L, *et al*. Phylogeography of the finless porpoise (genus *Neophocaena*): testing the stepwise divergence hypothesis in the northwestern Pacific. *Sci Rep* 2014;**4**:6572.
12. Li, S, Xu, S, Wan, H, *et al*. Genome-wide SNP and population divergence of finless porpoises. *Genome Biol Evol* 2013;**5**(4):758-68.
13. Zheng, J, Xia, J, He, S, *et al*. Population genetic structure of the Yangtze finless porpoise (*Neophocaena phocaenoides asiaeorientalis*): implications for management and conservation. *Biochem Genet* 2005;**43**(5-6):307-20.
14. Zhou, X, Guang, X, Sun, D, *et al*. Population genomics of finless porpoises reveal an incipient cetacean species adapted to freshwater. *Nat Commun* 2018;**9**(1):1276.
15. Yin, D, Lin, D, Guo, H, *et al*. Integrated analysis of blood mRNAs and microRNAs reveals immune changes with age in the Yangtze finless porpoise (*Neophocaena asiaeorientalis*). Comp *Biochem Physiol B Biochem Mol Biol* 2021;**256**:110635.
16. Liu, W, Yin, D, Lin, D, *et al*. Blood Transcriptome Analysis Reveals Gene Expression Differences between Yangtze Finless Porpoises from Two Habitats: Natural and Ex Situ Protected Waters. *fishes* 2022;**7**:96.
17. Nurk, S, Koren, S, Rhie, A, *et al*. The complete sequence of a human genome. *Science* 2022;**376**(6588):44-53.
18. Rhie A, Nurk S, Cechova M, *et al*. The complete sequence of a human Y chromosome. *Nature* 2023;**621**(7978):344-54.
19. Huang, Z, Xu, Z, Bai, H, *et al*. Evolutionary analysis of a complete chicken genome. *Proc Natl*

Acad Sci U S A 2023;**120**(8):e2216641120.

20. Xue, L, Gao, Y, Wu, M *et al*. Telomere-to-telomere assembly of a fish Y chromosome reveals the origin of a young sex chromosome pair. *Genome Biol* 2021;**22**(1):203.
21. Zhang, L, Lan, T, Lin, C, *et al*. Chromosome-scale genomes reveal genomic consequences of inbreeding in the South China tiger: A comparative study with the Amur tiger. *Mol Ecol Resour* 2023;**23**(2):330-47.
22. Shukla, H, Suryamohan, K, Khan, A, *et al*. Near-chromosomal de novo assembly of Bengal tiger genome reveals genetic hallmarks of apex predation. *Gigascience* 2022;**12**:giac112.
23. Yin, D, Chen, C, Lin, D, *et al*. Gapless genome assembly of East Asian finless porpoise. *Sci Data* 2022;**9**(1):765.
24. Bukhman, Y, Morin, P, Meyer, S, *et al*. A high-quality blue whale genome, segmental duplications, and historical demography. *Mol Biol Evol* 2024; **41**(3): msae036.
25. Rhie, A, Walenz, B, Koren, S, *et al*. Merqury: reference-free quality, completeness, and phasing assessment for genome assemblies. *Genome Biol* 2020;**21**(1):245.
26. Zhang, A, Kong, T, Sun, B, *et al*. A telomere-to-telomere genome assembly of Zhonghuang 13, a widely-grown soybean variety from the original center of Glycine max. *The Crop Journal* 2023. doi:https://doi.org/10.1016/j.cj.2023.10.003.
27. Brar, G, Amon, A. Emerging roles for centromeres in meiosis I chromosome segregation. *Nat Rev Genet* 2008;**9**(12):899-910.
28. Slade, R and McCallum, H. Overdominant vs. frequency-dependent selection at MHC loci. *Genetics* 1992;**132**(3):861–64.
29. Tian, R, Chen, M, Chai, S, *et al*. Divergent Selection of Pattern Recognition Receptors in Mammals with Different Ecological Characteristics. *J Mol Evol* 2018;**86**(2):138-49.
30. Lokesh, J and Kiron, V. Transition from freshwater to seawater reshapes the skin-associated microbiota of Atlantic salmon. *Sci Rep* 2016;**25**(6):19707.
31. Yuan, Y, Zhang, Y, Zhang, P, *et al*. Comparative genomics provides insights into the aquatic adaptations of mammals. *Proc Natl Acad Sci U S A* 2021;**118**(37):e2106080118.
32. Guo, B, Sun, Y, Wang, Y, *et al*. Evolutionary genetics of pulmonary anatomical adaptations in deep-diving cetaceans. *BMC Genomics* 2024;**25**(1):339.
33. Zhou, X, Sun, F, Xu, S, *et al*. Baiji genomes reveal low genetic variability and new insights into secondary aquatic adaptations. *Nat Commun* 2013;**4**:2708.
34. Li, L, Rong, X, Li, G, *et al*. Genomic organization and adaptive evolution of IGHC genes in marine mammals. *Mol Immunol* 2018;**99**:75-81.
35. Ming, Y, Jian, J, Yu, F, *et al*. Molecular footprints of inshore aquatic adaptation in Indo-Pacific humpback dolphin (*Sousa chinensis*). *Genomics* 2019;**111**(5):1034-42.
36. Chatterjee, N and Walker, G. Mechanisms of DNA damage, repair, and mutagenesis. *Environ Mol Mutagen* 2017;**58**(5):235-63.
37. Puschhof, J and Sears, C. Microbial metabolites damage DNA. *Science* 2022;**378**(6618):358-59.
38. Lv, W, Gu, H, He, D, *et al*. Polystyrene nanospheres-induced hepatotoxicity in swamp eel (*Monopterus albus*): From biochemical, pathological and transcriptomic perspectives. *Sci Total Environ* 2023;**893**:164844.
39. Zhang, K, Qian, Z, Ruan, Y, *et al*. First evaluation of legacy persistent organic pollutant contamination status of stranded Yangtze finless porpoises along the Yangtze River Basin,

China. *Sci Total Environ* 2020;**710**:136446.

40. Xiong, X, Qian, Z, Mei, Z, *et al*. Trace elements accumulation in the Yangtze finless porpoise (*Neophocaena asiaeorientalis asiaeorientalis*)-A threat to the endangered freshwater cetacean. *Sci Total Environ* 2019;**686**:797-804.
41. Shoemaker, V and Nagy, K. Osmoregulation in amphibians and reptiles. *Annu Rev Physiol*. 1977;**39**: 449-71.
42. Ruan, R, Guo, A, Hao, Y, *et al*. De novo assembly and characterization of narrow-ridged finless porpoise renal transcriptome and identification of candidate genes involved in osmoregulation. *Int J Mol Sci* 2015;**16**(1):2220-38.
43. Yan, M, Wei, G, Pan, X *et al*. A method suitable for extracting genomic DNA from animal and plant-modified CTAB method. *Agric Sci Technol*, 2008, **9**(2):39-41.
44. Chen Y, Chen Y, Shi C, *et al*. SOAPnuke: a MapReduce acceleration-supported software for integrated quality control and preprocessing of high-throughput sequencing data. *Gigascience* 2018;**7**(1):1-6.
45. Chen Y, Nie F, Xie S, *et al*. Efficient assembly of nanopore reads via highly accurate and intact error correction. *Nat Commun* 2021;**12**(1):60.
46. Cheng, H, Concepcion, G, Feng, X, *et al*. Haplotype-resolved de novo assembly using phased assembly graphs with hifiasm. *Nat Methods* 2021;**18**(2):170-5.
47. Roach, M, Schmidt, S and Borneman, A. Purge Haplotigs: allelic contig reassignment for third-gen diploid genome assemblies. *BMC bioinformatics* 2018;**19**(1):460.
48. Durand, N, Shamim, M, Machol, I, *et al*. Juicer Provides a One-Click System for Analyzing Loop-Resolution Hi-C Experiments. *Cell systems* 2016;**3**(1):95-8.
49. Dudchenko, O, Batra, S, Omer, A, *et al*. De novo assembly of the Aedes aegypti genome using Hi-C yields chromosome-length scaffolds. *Science* 2017;**356**(6333):92-5.
50. Xu, G, Xu, T, Zhu, R, *et al*. LR_Gapcloser: a tiling path-based gap closer that uses long reads to complete genome assembly. *Gigascience* 2019;**8**(1):giy157.
51. Xu M, Guo L, Gu S, *et al*. TGS-GapCloser: A fast and accurate gap closer for large genomes with low coverage of error-prone long reads. *Gigascience* 2020;**9**(9):giaa094.
52. Waterhouse, R, Seppey, M, Simão, F, *et al*. BUSCO Applications from Quality Assessments to Gene Prediction and Phylogenomics. *Mol Biol Evol* 2018;**35**(3):543-8.
53. Li, H. Minimap2: pairwise alignment for nucleotide sequences. *Bioinformatics* 2018;**34** (18):3094-100.
54. Kim, D, Paggi, J, Park, C, *et al*. Graph-based genome alignment and genotyping with HISAT2 and HISAT-genotype. *Nat Biotechnol* 2019;**37**(8):907-15.
55. Lin, Y, Ye, C, Li, X, *et al*. quarTeT: a telomere-to-telomere toolkit for gap-free genome assembly and centromeric repeat identification. *Hortic Res* 2023;**10**(8):uhad127.
56. Chen N. Using RepeatMasker to identify repetitive elements in genomic sequences. *Curr Protoc Bioinformatics* 2004;**Chapter 4**:Unit 4.10.
57. Xu, Z and Wang, H. LTR_FINDER: an efficient tool for the prediction of full-length LTR retrotransposons. *Nucleic Acids Res* 2007;**35**:W265-8.
58. Price, A, Jones, N and Pevzner, P. De novo identification of repeat families in large genomes. *Bioinformatics* 2005;**21**(Suppl 1):i351-8.
59. Benson, G. Tandem repeats finder: a program to analyze DNA sequences. *Nucleic Acids Res* 1999;**27**(2):573-80.

60. Kim, D, Langmead, B and Salzberg, S. HISAT: a fast spliced aligner with low memory requirements. *Nat Methods* 2015;**12**(4):357-60.
61. Kovaka, S, Zimin, A, Pertea, G, *et al*. Transcriptome assembly from long-read RNA-seq alignments with StringTie2. *Genome Biol* 2019;**20**(1):278.
62. Haas, B, Salzberg, S, Zhu W, *et al*. Automated eukaryotic gene structure annotation using EVidenceModeler and the Program to Assemble Spliced Alignments. *Genome Biol* 2008;**9**(1):R7.
63. Keilwagen, J, Hartung, F and Grau, J. GeMoMa: Homology-Based Gene Prediction Utilizing Intron Position Conservation and RNA-seq Data. *Methods Mol Biol* 2019;**1962**:161-77.
64. Stanke, M and Waack, S. Gene prediction with a hidden Markov model and a new intron submodel. *Bioinformatics* 2003;**19**(Suppl 2):ii215-25.
65. Bairoch, A and Apweiler, R. The SWISS-PROT protein sequence data bank and its supplement TrEMBL. *Nucleic Acids Res* 1997;**25**(1):31-6.
66. Kanehisa, M, Sato, Y, Kawashima, M, *et al*. KEGG as a reference resource for gene and protein annotation. *Nucleic Acids Res* 2016;**44**(D1):D457-62.
67. Jones, P, Binns, D, Chang, H, *et al*. InterProScan 5: genome-scale protein function classification. *Bioinformatics* 2014;**30**(9):1236-40.
68. Ashburner, M, Ball, CA, Blake, J, *et al*. Gene ontology: tool for the unification of biology. The Gene Ontology Consortium. *Nat Genet* 2000;**25**(1):25-9.
69. Li, T, Xu, X, Zhao, J, *et al*. Genome assembly of KA105, a new resource for maize molecular breeding and genomic research. *The Crop Journal* 2023;**11**(6):1793-1804.
70. Marçais, G, Delcher, A, Phillippy, A, *et al*. MUMmer4: A fast and versatile genome alignment system. *PLoS Comput Biol* 2018;**14**(1):e1005944.
71. Goel, M, Sun, H, Jiao, W, *et al*. SyRI: finding genomic rearrangements and local sequence differences from whole-genome assemblies. *Genome Biol* 2019;**20**(1):277.
72. Wang, K, Li, M and Hakonarson, H. ANNOVAR: functional annotation of genetic variants from high-throughput sequencing data. *Nucleic Acids Res* 2010;**38**(16):e164.
73. Yin, L, Zhang, H, Tang, Z, *et al*. rMVP: A Memory-efficient, Visualization-enhanced, and Parallel-accelerated tool for Genome-Wide Association Study. *Genomics, Proteomics & Bioinformatics* 2021;**19**(4):619-28.
74. Emms, D and Kelly, S. OrthoFinder: phylogenetic orthology inference for comparative genomics. *Genome Biol* 2019;**20**(1):238.
75. Nakamura, T, Yamada, K, Tomii, K, *et al*. Parallelization of MAFFT for large-scale multiple sequence alignments. *Bioinformatics* 2018;**34**(14):2490-2.
76. Guindon, S, Delsuc, F, Dufayard, J, *et al*. Estimating maximum likelihood phylogenies with PhyML. *Methods Mol Biol* 2009;**537**:113-37.
77. Yang, Z. PAML 4: phylogenetic analysis by maximum likelihood. *Mol Biol Evol* 2007;**24**(8):1586-91.
78. Altschul, S, Gish, W, Miller, W, *et al*. Basic local alignment search tool. *J Mol Biol* 1990;**215**(3):403-10.
79. Wang, Y, Tang, H, Debarry, J, *et al*. MCScanX: a toolkit for detection and evolutionary analysis of gene synteny and collinearity. *Nucleic Acids Res* 2012;**40**(7):e49.
80. Bie, T, Cristianini, N, Demuth, J, *et al*. CAFE: a computational tool for the study of gene family evolution. *Bioinformatics* 2006;**22**(10):1269-71.

81. Liao, Y, Smyth, G and Shi, W. featureCounts: an efficient general purpose program for assigning sequence reads to genomic features. *Bioinformatics* 2014;**30**(7):923-30.

Tables

Table 1. Genome assembly statistics of YFP and EFP.

	**YFP v2.0**	**YFP v1.0**	**EFP v2.0**	**EFP v1.0**
Total size of assembled genome (Gb)	2.48	2.27	2.50	2.50
Contig N50 (Mb)	125.12	0.09	128.00	84.69
Contig N90 (Mb)	83.62	0.02	80.21	29.54
Number of contigs	~~23~~22	66,345	~~24~~ 23	~~52~~ 51
Scaffold N50 (Mb)	125.12	6.34	128.00	122.40
Scaffold N90 (Mb)	83.62	1.13	80.21	80.21
Scaffolds number	22	13,698	23	23
Number of base chromosomes	22	22	23	23
Number of gap-free chromosomes	22	0	23	7
Number of gaps	0	52,647	0	28
Number of telomeres (pairs/single)	20/2	0/0	21/2	21/2
Number of estimated centromeres	22	0	23	23
TE size	42.54%	NA	42.80%	42.23%
GC content	41.70%	41.00%	41.70%	41.70%
BUSCO* (Genome)	95.2%	94.0%	95.3%	95.4%
Gene Number	23,139	18,479	23,101	22,814
New-found gene number	5,480	NA	1,453	NA
Functional proteins	96.21%	NA	96.20%	97.31%
BUSCO* (Protein)	97.5%	94.6%	97.6%	97.9%
Data source	This study (PRJNA915046)	GCF_003031525.2	This study (PRJNA859258)	GCA_026225855.1

Note: The term "v2.0" denotes the updated genome assembly and annotation produced in the present study, while "v1.0" refers to the initial draft of the genome assembly and annotation that was previously published. "BUSCO*" indicated the percentage of complete BUSCO evaluation. "pairs/single": "pairs" indicated that the telomeres were found at both ends of the chromosomes; "single" indicated that the telomeres were found only at the one end of chromosomes. "New-found genes" indicated that the genes were predicted in the extra sequence segments from the current assembly and were annotated in the current assembly.

Figure legends

Figure 1. Sample site and Genome assessment of YFP v2.0 and EFP v2.0.

A: Location distribution and sampling site of the YFP and EFP.

B: Proportions of genes that could be functionally annotated and transcriptionally detected in YFP v2.0 and EFP v2.0.

Figure 2. T2T-resolved assembly of YPF v2.0 and EFP v2.0 and functional enrichment of genes in the centromere region.

Structure of T2T and gap-free chromosomes in A) YFP v2.0, and B) EFP v2.0. All 21+X/Y chromosomes are drawn to scale and the ruler indicates chromosome length. Triangles indicate the presence of telomere sequence repeats. Circles represent the locations of centromeric regions. The gap positions in the v1.0 genome assemblies are marked with squares to the right of the chromosome in the v2.0 genome assemblies.

Figure 3. Synteny analysis of YFP v2.0 and EFP v2.0 genomes: A) chromosomes scale, Unit length is Mb; B) syntenic blocks between YFP v2.0 and EFP v2.0.

Figure 4. Structure variant between YFP v2.0 and EFP v2.0 genome assembly with YFP v2.0 genome assembly for reference.

The density plot of A) SNPs and B) InDels between YFP v2.0 and EFP v2.0 genome assembly. The numerical values adjacent to the color legend indicate the count of SNPs/InDels per megabase (MB) window, where the gray legend representing zero. Each color corresponds to a specific range of values. For instance, in Figure 4A, the initial blue legend represents the range from 1 to 4440.

Figure 5. Genome evolution of YFP v2.0 and EFP v2.0.

A: Divergence time between YFP v2.0 and EFP v2.0, and number of expanded and contracted gene families. Green and red numbers indicate gene family expansions and contractions, respectively. MRCA: Most Recent Common Ancestor. Ma: Million years ago.

B: A comparison of gene families associated with orthologs and paralogs in YFP v2.0 and EFP v2.0, and other 24 mammal species.

Figure 6. Functional Enrichment of genes.

Significant A) KEGG and B) GO enrichment of expanded gene families in YFP and EFP lineage. KEGG enrichment analysis of positively selected genes in C) YFP and D) EFP, respectively.

Response to reviewer Click here to access/download;Manuscript;Response to reviewer.docx

Dear Dr. Hans Zauner,

Thanks for your kind consideration and comments regarding to our manuscript entitled "Telomere-to-telomere gap-free genome assembly of the endangered Yangtze finless porpoise and East Asian finless porpoise" (GIGA-D-23-00359R1). We have revised our manuscript (details are provided below) in accordance with all comments and suggestions from the reviewer 2. We hope the revised manuscript have been improved and meet the standard requirement. We look forward to hearing from *GigaScience* soon.

Sincerely yours,

Correspondence: Kai Liu

Freshwater Fisheries Research Center, Chinese Academy of Fishery Sciences, Wuxi 214081, China.

E-mail: liuk@ffrc.cn

Response to reviewer 2's comments:

Reviewer #2: In this manuscript, Denghua Yin and colleagues present two new, high-quality, de novo genome assemblies of two narrow-ridged finless porpoise (*Neophocaena asiaeorientalis*) subspecies (Yangtze, YFP and East Asia, EFP) as well as a comprehensive report on its quality and analyses that identify molecular differences related to their different habitats (fresh and sea water). This is the second time I'm reading this manuscript and I think the manuscript was substantially improved by the changes made in response to the last review. I still regard these genomes to be of the highest quality and a valuable resource for future studies. The methodological shortcomings which I highlighted last time were completely addressed and the language problems were amended in most cases. The only general issue that wasn't covered enough since last time is in the discussion where the authors fail to provide more comparisons to assemblies from the literature. The authors mention in the conclusion that these assemblies are potentially the "most continuous" genomes of cetacean today and I tend to believe them. However, the manuscript is lacking a clear comparison to other assemblies to make this statement. I already suggested this in my last review, and I consider this to be crucial, although I'm aware that this conflicts to some extent with the suggestions made by reviewer 1. Hence, I suggest to the authors to add just a brief comparison (2-3 sentences) to the discussion to validate this claim, to provide some contextual statistics and to give the reader an idea about the improved quality of these genomes. In the following, I'll list some additional suggestions that may improve the manuscript. Given that these suggestions require only minor changes to the text, I recommend a minor revision to the editor.

Response: Thank you very much for your comments on our manuscript. The comments provided are all valuable and very helpful for revising and improving our paper. We have addressed the only general issue raised in comment 1. Based on the comments we received, detailed modifications are provided below.

General issues:

Comment 1: As mentioned already, I suggest adding a small comparative paragraph to the discussion to compare the quality of these genomes to previous genomes of Cetacea.

Response: According to the reviewer's suggestion, we have compared the genome assemblies presented in this study with 34 cetacean genomes available from public repositories such as NCBI and CNGB. The comprehensive analysis confirms that our assemblies are the most continuous among cetaceans known to date. Notably, our contig N50 and scaffold N50 values significantly surpass those of other genomes, while the number of contigs is considerably lower, highlighting the improved continuity and quality of our assemblies. In addition, a table summarizing the statistical comparison of the genomes has been included. This table is now cited in the main text and is presented as Supplementary Table S3. This table provides a concise overview of the data that underpins our assertions and enables a quick assessment of the comparative genomic consistency among cetacean species.

We have added a comparative paragraph at lines 120-129 in the revised MS: "As of May 2024, the killer whale genome assembly (GCF_937001465.1) stands out among the 34 publicly available cetacean genomes sourced from the NCBI (National Center for Biotechnology Information) and CNGBdb (China National GeneBank DataBase) databases. It features an N50 length of 45.6 Mb and is composed of 570 contigs. In our study, the two newly assembled genomes exhibit a marked improvement in contiguity. The contig N50 length achieved in our assemblies is threefold that of the killer whale genome, with the number of contigs comprising only 4.03% of the latter. Moreover, the analysis of the blue whale genome (GCF_009873245.2) shows a contig N50 value that is only 4.0% of our assemblies, while its contig count is 42 times higher [1] (Supplementary Table S3). The comparative metrics highlight the superior continuity of the YFP and EFP genome assemblies in this study."

Ref:

[1] Bukhman, Y, Morin, P, Meyer, S, *et al*. A high-quality blue whale genome, segmental duplications, and historical demography. *Mol Biol Evol* 2024; **41**(3): msae036.

Supplementary Table S3 Assembly statistics for 36 species of cetaceans.

Species	Common name	Genome Size/Gb	Contig N50/Mb	Contig Numbers	Super-scaffold N50/Mb	Scaffold Number	Data Source
Neophocaena asiaeorientalis sunameri	East Asian finless porpoise (EFP v2.0)	2.50	128.00	23	128.00	23	This study (PRJNA859258)
Neophocaena asiaeorientalis asiaeorientalis	Yangtze finless porpoise (YEP v2.0)	2.48	125.12	22	125.12	22	This study (PRJNA915046)
Neophocaena asiaeorientalis asiaeorientalis	East Asian finless porpoise (EFP v1.0)	2.50	84.69	52	122.40	23	GCA_026225855.1
Orcinus orca	Killer whale	2.6	45.6	570	114.20	447	GCF_937001465.1
Kogia breviceps	Pygmy sperm whale	2.60	42.80	723	122.40	580	GCF_026419965.1
Inia geoffrensis	Boutu	2.70	40.90	1,186	117.40	902	GCA_036417435.1
Eschrichtius robustus	Grey whale	3.00	39.2	982	126.50	703	GCA_028021215.1
Eubalaena glacialis	North Atlantic right whale	2.80	37.50	1,155	129.60	788	GCF_028564815.1
Balaenoptera ricei	Rice's whale	2.70	36.50	1,108	122.10	774	GCF_028023285.1
Phocoena sinus	vaquita	2.40	20.20	272	115.50	64	GCF_008692025.1
Tursiops truncatus	Bottlenosed dolphins	2.37	9.73	1,035	108.43	361	GCF_011762595.1
Balaenoptera musculus	Blue whale	2.40	6.30	972	110.30	105	GCF_009873245.2
Phocoena phocoena	Harbor porpoise	2.50	3.7	1,405	110.2	439	GCA_963924725.1
Delphinus delphis	saddleback dolphin	2.70	3.60	1,598	107.1	630	GCF_949987515.1
Stenella coeruleoalba	Striped dolphin	2.70	3.60	1,630	105.30	637	GCA_951394435.1
Lagenorhynchus albirostris	White-beaked dolphin	2.5	3.40	1,479	110.7	409	GCF_949774975.1
Globicephala melas	Long-finned pilot whale	2.70	3.30	2,076	105.10	992	GCF_963455315.1
Hyperoodon ampullatus	Northern bottlenose whale	2.80	3.10	1,854	117.4	756	GCA_949752795.1
Balaenoptera acutorostrata	Minke whale	2.80	3.00	2,551	116.50	1,374	GCF_949987535.1
Lagenorhynchus obliquidens	Pacific white-sided dolphin	2.46	0.28	29,411	30.04	13,174	CNA0007259

Delphinapterus leucas	Beluga whale	2.32	0.20	29,443	31.18	5,904	GCF_002288925.2
Sousa chinensis	Indo-Pacific humpback dolphin	2.34	0.18	46,900	9.01	20,903	GCA_003521335.2
Pseudorca crassidens	False killer whale	2.43	0.15	60,880	32.50	32,690	CNA0007265
Tursiops aduncus	Indo-Pacific bottlenose dolphin	2.41	0.11	160,669	104.71	118,124	CNA0007267
Neophocaena asiaeorientalis asiaeorientalis	Yangtze finless porpoise (YEP v1.0)	2.27	0.09	66,342	6.34	13,695	GCF_003031525.2
Balaenoptera musculus	Blue whale	2.37	0.08	79,155	8.28	28,500	CNA0007254
Balaenoptera bryde	Bryde’s whale	2.37	0.07	140,815	103.91	74,190	CNA0007253
Grampus griseus	Risso's dolphin	2.50	0.06	813,233	93.20	773,381	GCA_028646425.1
Kogia sima	Dwarf sperm whale	2.59	0.06	503,547	26.52	428,024	CNA0007257
Steno bredanensis	Rough-toothed dolphin	2.30	0.06	266,806	94.90	221,678	GCA_028646385.1
Lagenorhynchus australis	Peale’s dolphin	2.39	0.05	225,716	0.38	151,990	CNA0007258
Balaena mysticetus	Bowhead whale	2.31	0.04	118,156	0.88	7,227	bowhead-whale.org
Physeter catodon	Sperm whale	2.51	0.04	133,554	122.18	12,168	GCF_002837175.3
Lipotes vexillifer	Yangtze River dolphin	2.37	0.03	148,597	2.44	24,809	GCF_000442215.2
Mesoplodon densirostris	Blainville’s beaked whale	2.47	0.03	1,071,210	0.80	944,262	CNA0007260
Neophocaena phocaenoides	Indo-Pacific finless porpoise	2.62	0.01	2,048,710	0.43	1,806,060	CNA0007261

Abstract:

Comment 2: Line 31: I do not think you can "study comparative genomes". Consider "study structural similarities" instead.

Response: Thank you very much for your suggestion. We have changed "comparative genomes" to "structural similarities".

Introduction:

Comment 3: Line 95-98: Same as in line 30. Furthermore, the article "the" is used in a confusing way here. Consider changing the sentence to: "We compare the quality of the newly drafted assemblies with previously available versions and explore synteny blocks, structural similarities, phylogenetic relationships, gene family expansion and inference of selection in relation to several other mammals."

Response: We are sorry for the mistake. We have changed the sentence to: "We compare the quality of the newly drafted assemblies with previously available versions and explore synteny blocks, structural similarities, phylogenetic relationships, gene family expansion and inference of selection in relation to several other mammals."

Results and Discussion:

Comment 4: Line 127: Consider changing "a gap-free genome" to a plural form.

Response: Thank you very much for your suggestion. We have changed "a gap-free genome" to "gap-free genomes".

Comment 5: Line 132: Thank you for choosing to explain the Merqury scores. However, I'm missing the sentences: "These values indicated the superior quality of our assemblies. It is essential to emphasize that Merqury quality values span from 0 to 255, with higher values denoting superior quality." They were mentioned in the response to the reviewer but not in the manuscript. Is there a reason for this?

Response: We apologize for the mistake. The sentences "These values indicated the superior quality of our assemblies. It is essential to emphasize that Merqury quality values span from 0 to 255, with higher values denoting superior quality." have been

included at lines 131-133 in the revised MS.

Comment 6: Line 220-221: A citation is missing for this statement.

Response: We are sorry for the mistake. We have added a citation for this statement.

Ref:

Brar, G, Amon, A. Emerging roles for centromeres in meiosis I chromosome segregation. *Nat Rev Genet* 2008;**9**(12):899-910.

Comment 7: Line 283: Although the authors probably mentioned the paper somewhere already, I would suggest adding a citation for this first subspecies classification here.

Response: Thank you for your suggestion. We have added a citation for this first subspecies classification here.

Ref:

Zhou, X, Guang, X, Sun, D, *et al*. Population genomics of finless porpoises reveal an incipient cetacean species adapted to freshwater. *Nat Commun* 2018;**9**(1):1276.

Comment 8: Line 312: The genus name of *Lipotes vexillifer* is already shortened here to a "L." although its not written out beforehand in the manuscript. I would suggest writing out the name here and shorten it at line 329.

Response: Thank you very much for your suggestion. We have written out "*Lipotes vexillifer*" at line 283, and shorten it as "*L. vexillifer*" at line 300 in the new revised MS. In addition, we have abbreviated "*N. a. asiaeorientalis*" and "*N. a. sunameri*" at line 296-298, as well as "*B. mysticetus*", "*B. acutorostrata*", "*S. chinensis*" and "*T. truncates*" at line 453-454 since they were previously mentioned in the manuscript.

Methods:

Comment 9: Line 444: I appreciate that the authors clarified the RepBase usage. However, for this workflow here to be reproduceable, you should usually provide the version number of RepBase and/or the date when the database was retrieved. Consider adding this information to ensure reproducibility.

Response: Thank you very much for your suggestion. We have provided the version number of RepBase database (v21.12) at line 401 in the revised MS.

Comment 10: Line 467: Consider changing the capital to a lower case "I" in "Information".

Response: We are sorry for the mistake. We have changed "Genome comparison and Identification of newly assembled genes" to "Genome comparison and identification of newly assembled genes".

Comment 11: Line 469: described where? Please add details to this statement.

Response: We are sorry for the unclear description. We have added a citation for this statement.

Ref:

Li, T, Xu, X, Zhao, J, *et al*. Genome assembly of KA105, a new resource for maize molecular breeding and genomic research. *The Crop Journal* 2023;**11**(6):1793-1804.

Comment 12: Line 499+501: I'm not sure if the phrases "a widely utilized tool for multiple sequence alignment" and "a commonly employed software tool for phylogenetic analysis" are the result of some corrections meant to answer suggestions made by reviewer 1, but for me, these phrases sound unnecessary and strange.

Response: We are sorry for the unclear description. We have changed "Single-copy orthologous genes (1:1:1) were aligned using MAFFT (v7.310), a widely utilized tool for multiple sequence alignment. Subsequently, a maximum-likelihood phylogenetic tree was generated using PhyML (v3.3), a commonly employed software tool for phylogenetic analysis." to "We utilized MAFFT (v7.310) and PhyML (v3.3) software tools to align single-copy orthologous genes (1:1:1) and construct a maximum-likelihood phylogenetic tree, respectively."

Conclusion:

Comment 13: Line 569: Here the authors clearly state that these genomes are probably the "most continuous" genomes of cetacean thus far. Again, for this statement to be justified, I strongly recommend adding numbers and citations to this claim somewhere in the discussion.

Response: Thank you very much for your suggestion. We have addressed the only general issue raised in comment 1. In addition, we have added the sentence in the Conclusion section: Compared to the 34 cetacean genomes sourced from public databases, the two genomes newly assembled in our study exhibit the longest contig N50 and scaffold N50, along with the lowest number of contigs, thus achieving superior continuity.

Figures:

Comment 14: Figure 5 (Phylogeny): Although beautiful, I'm very sure that the time axis in the bottom of the graph won't be readable when printed out or implemented within a PDF version of the article. This axis is just too small.

Response: Thank you for your feedback. We have increased the size of the time axis at the bottom of the graph to ensure it is readable when printed or viewed in the PDF version of the article.

Click here to access/download;Figure;Figure 1.pdf

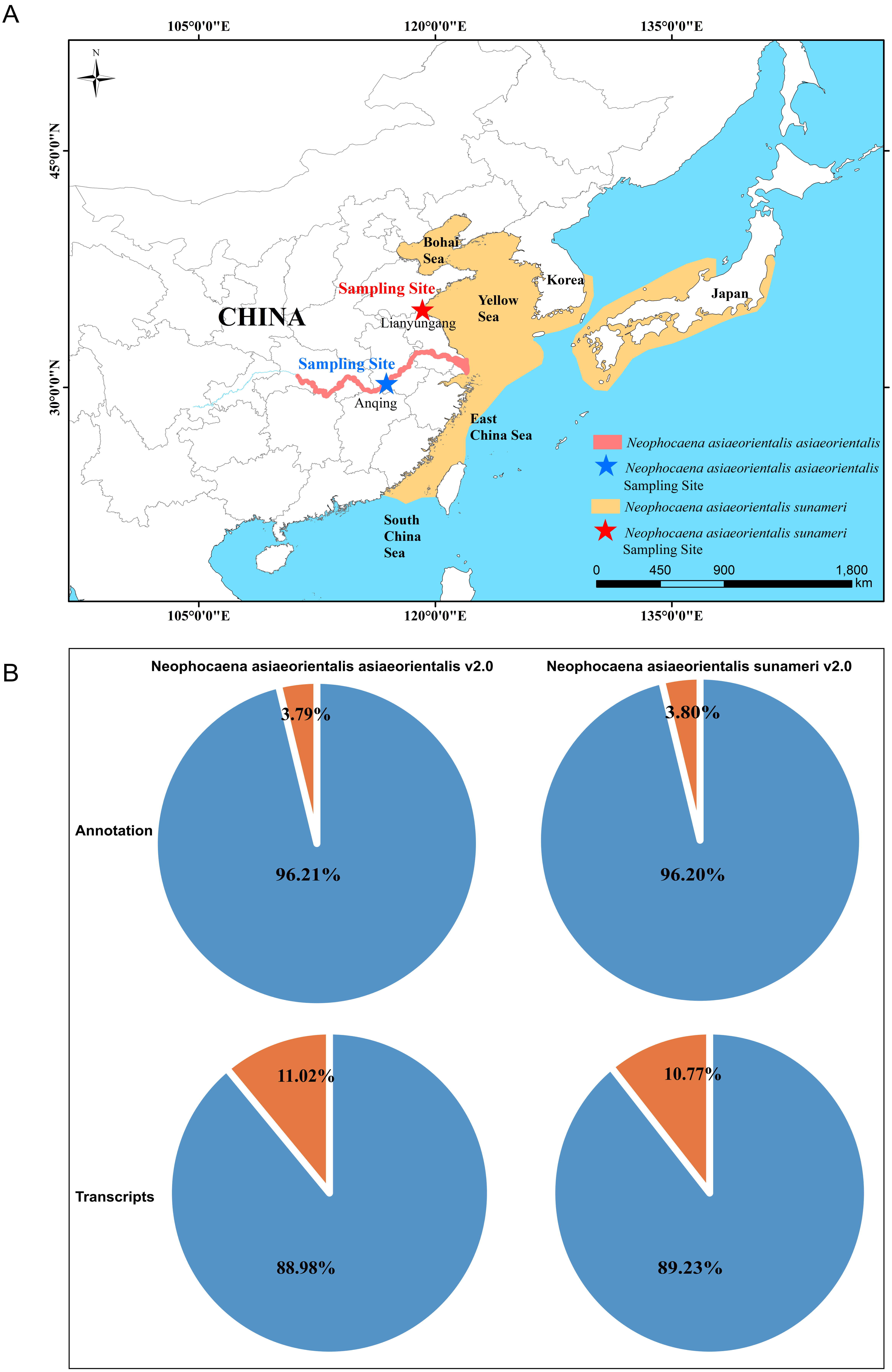

Figure 2

Click here to access/download;Figure;Figure 2.pdf

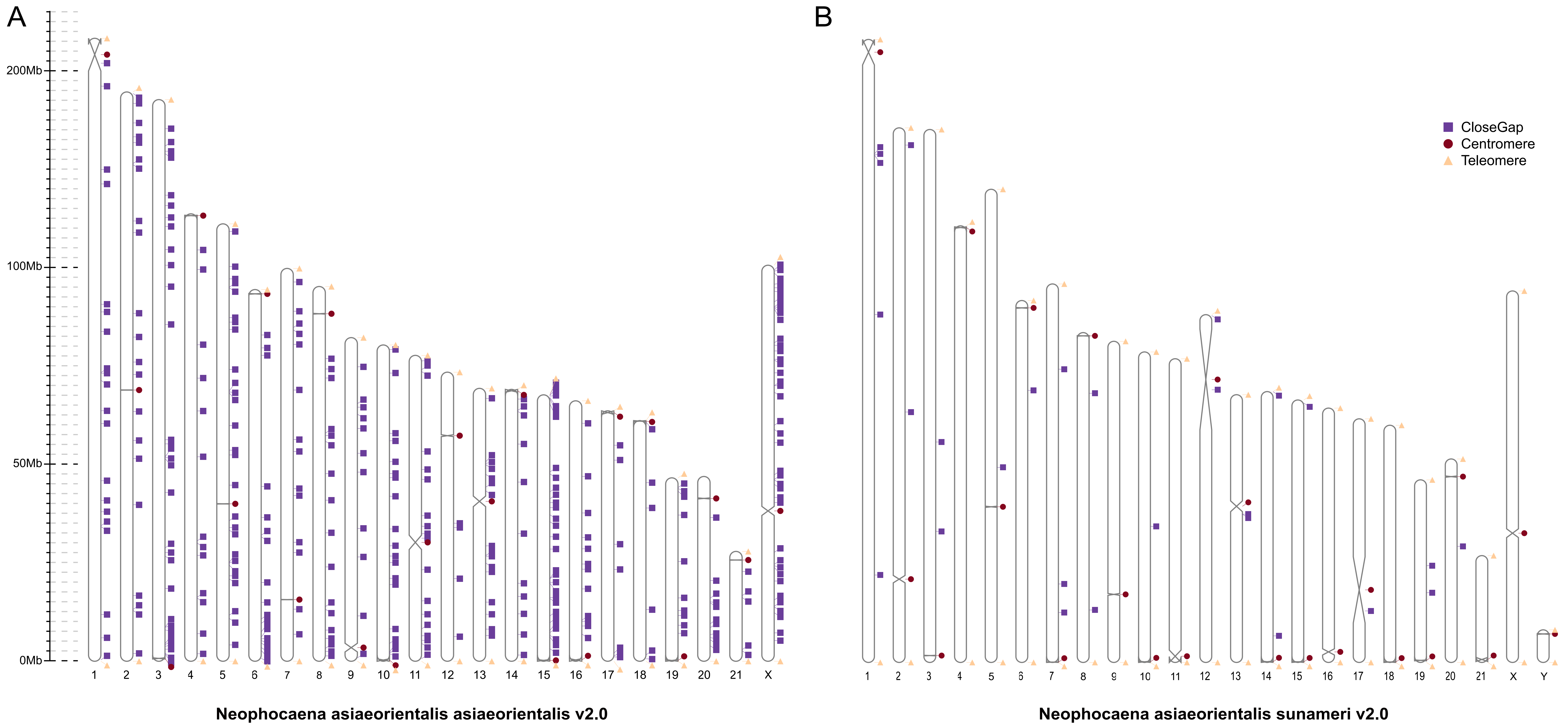

Figure 3

Click here to access/download;Figure;Figure 3.pdf

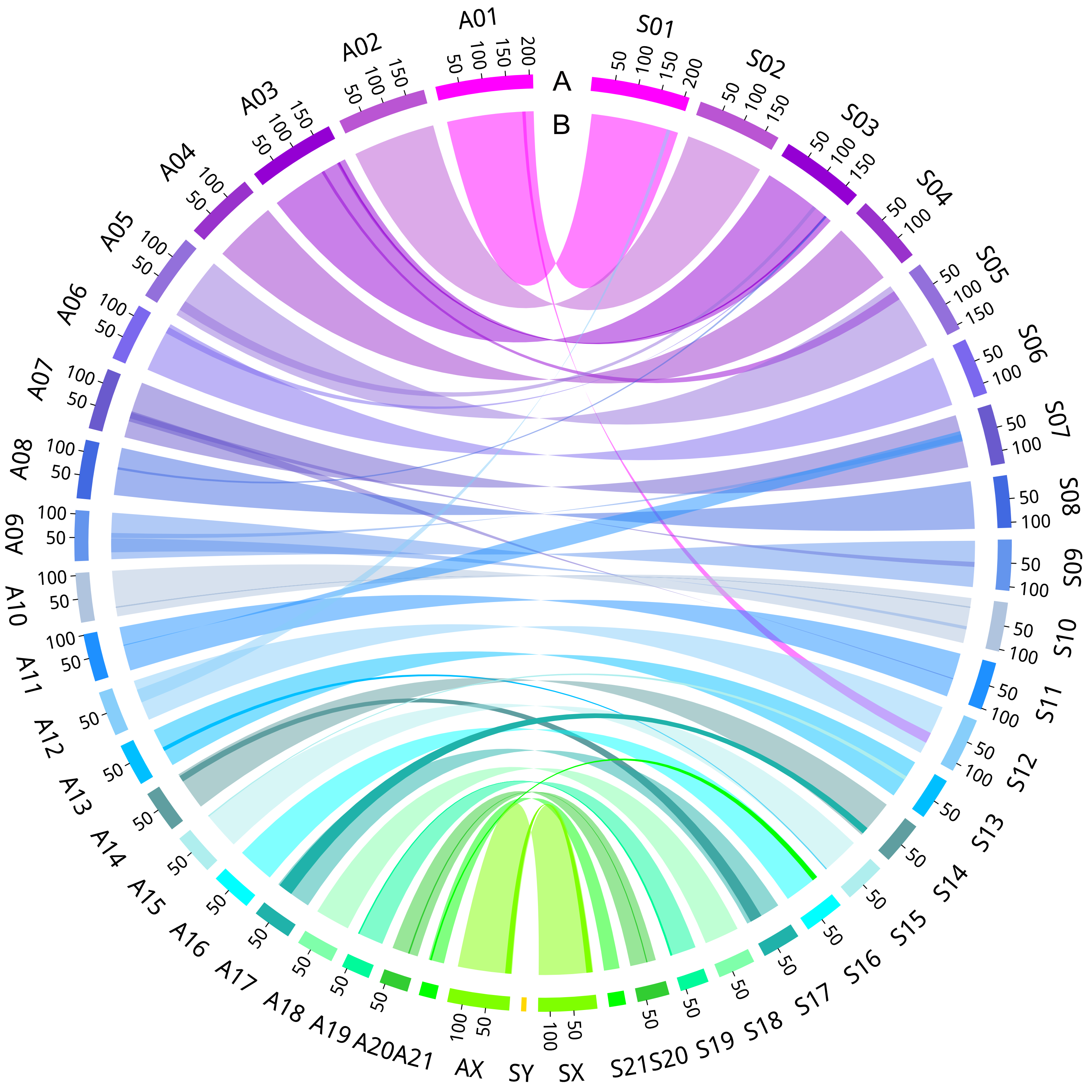

Figure 4

Click here to access/download;Figure;Figure 4.pdf

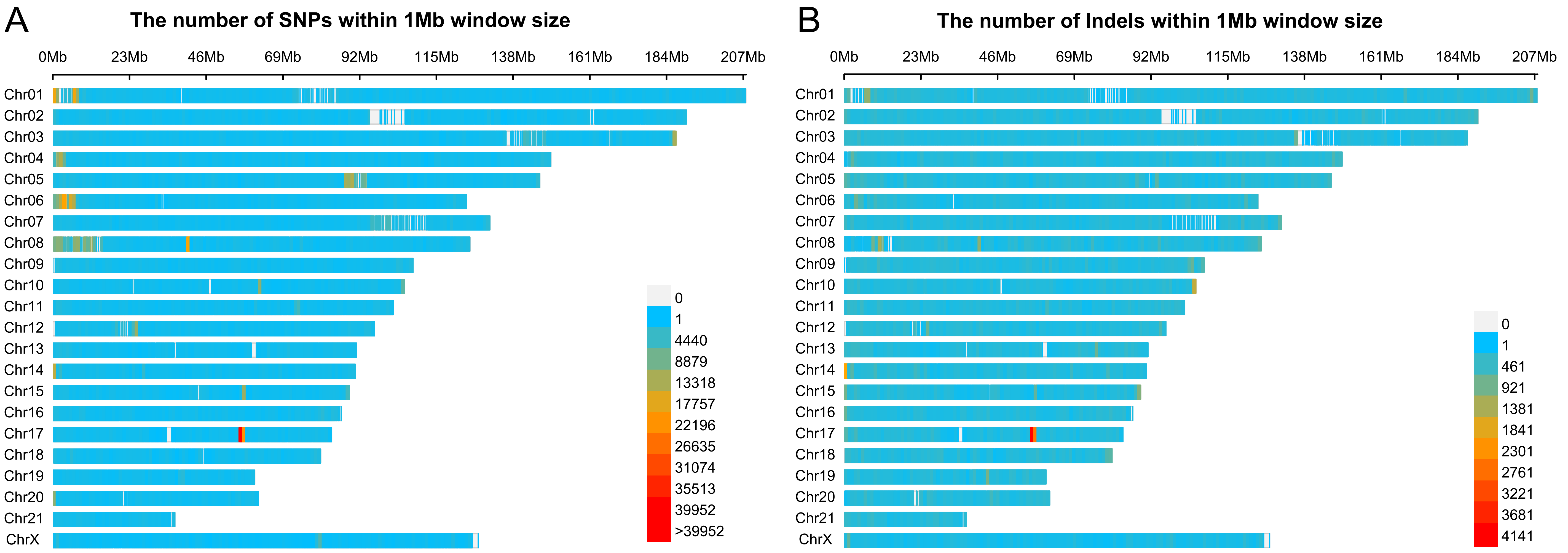

Figure 6
Click here to access/download;Figure;Figure 6.pdf

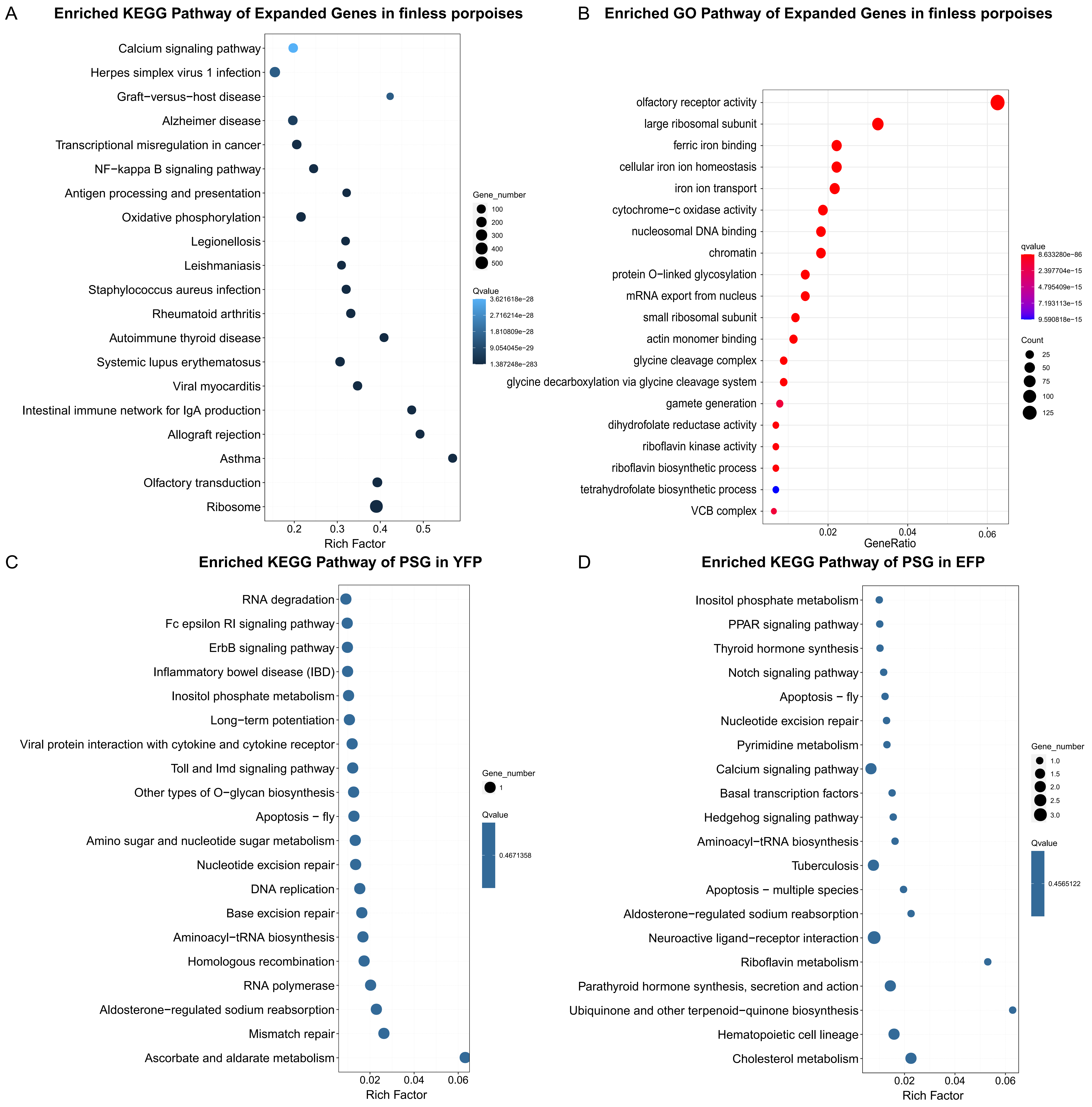

Click here to access/download;Figure;Figure 5.pdf

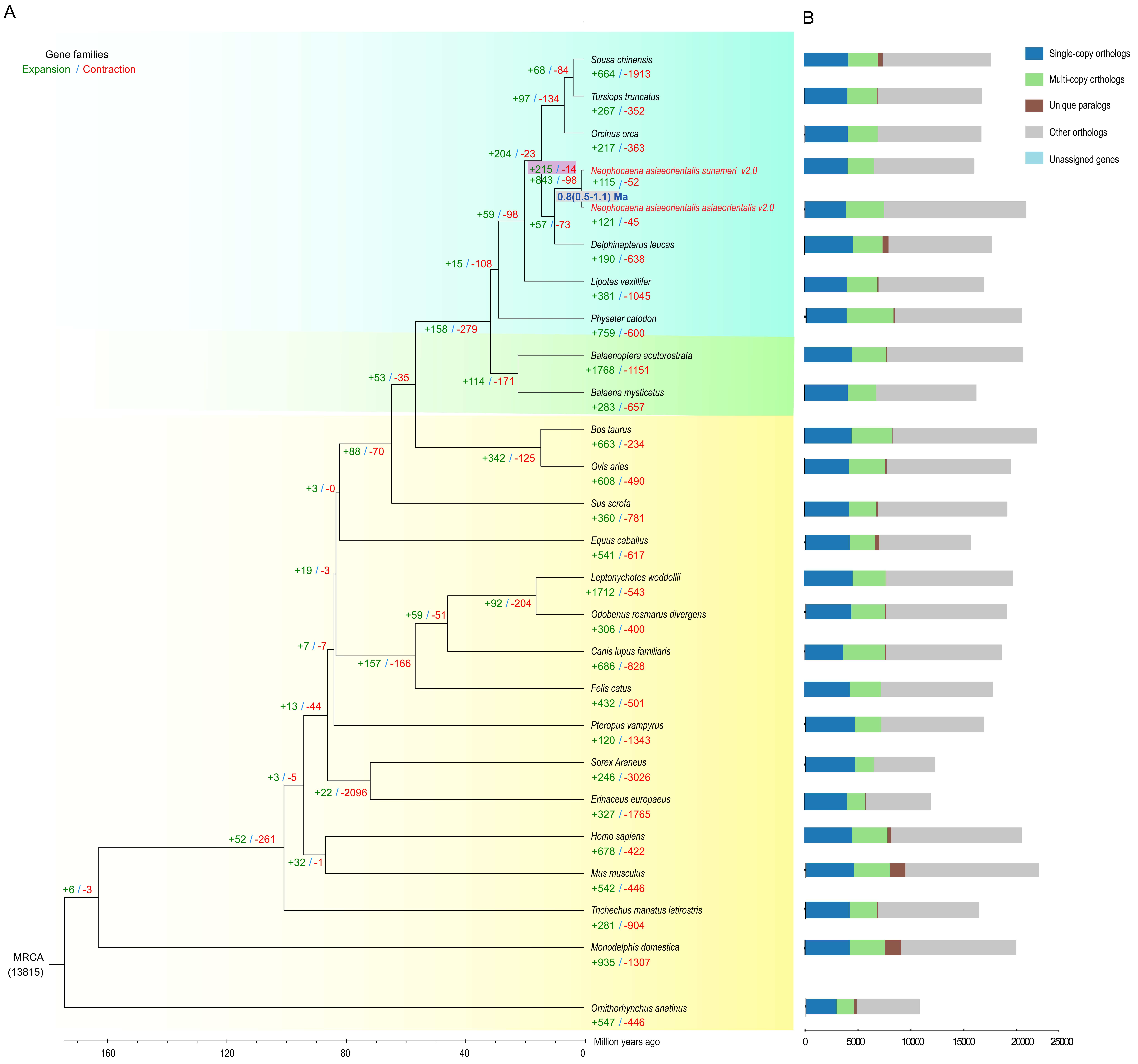

Click here to access/download
Supplementary Material
Supplementary Materials.docx

Supplementary Table S17

Click here to access/download
Supplementary Material
Supplementary Table S17.xls

Click here to access/download
Supplementary Material
Supplementary Table S18.xls

Click here to access/download
Supplementary Material
Supplementary Table S19.xls

Supplementary Table S20

Click here to access/download
Supplementary Material
Supplementary Table S20.xls

Supplementary Table S21

Click here to access/download
Supplementary Material
Supplementary Table S21.xls

Supplementary Table S23

Click here to access/download
Supplementary Material
Supplementary Table S23.xls

Supplementary Table S25

Click here to access/download
Supplementary Material
Supplementary Table S25.xls

Supplementary Table S26

Click here to access/download
Supplementary Material
Supplementary Table S26.xls

Supplementary Table S27

Click here to access/download
Supplementary Material
Supplementary Table S27.xls

Supplementary Table S28

Click here to access/download
Supplementary Material
Supplementary Table S28.xls

Supplementary Table S29

Click here to access/download
Supplementary Material
Supplementary Table S29.xls

Supplementary Table S30

Click here to access/download
Supplementary Material
Supplementary Table S30.xls
