# Supplementary material for: Telomere-to-telomere gap-free genome assembly of the endangered Yangtze finless porpoise and East Asian finless porpoise
Source: Gigascience. 2024 Sep 16;13:giae067. doi: 10.1093/gigascience/giae067 (PMC11403816; doi:10.1093/gigascience/giae067)
Supplement: giae067_GIGA-D-23_00359_Original_Submission [file giae067_giga-d-23_00359_original_submission.pdf]

GigaScience

Telomere-to-telomere gap-free genome assembly of the endangered Yangtze finless porpoise and East Asian finless porpoise

--Manuscript Draft--

**Manuscript Number:**	GIGA-D-23-00359
**Full Title:**	Telomere-to-telomere gap-free genome assembly of the endangered Yangtze finless porpoise and East Asian finless porpoise
**Article Type:**	Research
**Funding Information:**	the National Key R&D Program of China (2021YFD1200304) — Doctor Danqing Lin
**Abstract:**	The Yangtze finless porpoise (Neophocaena asiaeorientalis asiaeorientalis, YFP) and the East Asian finless porpoise (Neophocaena asiaeorientalis sunameri, EFP) are two subspecies of the narrow-ridged finless porpoise that live in fresh and salt water, respectively. They serve as ideal models for the study of freshwater adaptation and evolution. The main objective of this study was to provide contiguous chromosome-level genome assemblies for YFP and EFP. Here, we generated and upgraded genomes of YFP and EFP at the telomere-to-telomere level by combining PacBio HiFi long reads, ultra-long ONT reads and Hi-C sequencing data, with a total size of 2.48 Gb and 2.50 Gb respectively. The scaffold N50 of two genomes was 125.12 Mb (YFP) and 128 Mb (EFP) with one contig for one chromosome. The telomere repeat and centromere position were clearly identified in both YFP and EFP genomes. Telomeric repeat units were detected at 85% and 90% of the chromosome both ends in YFP and EFP genomes, respectively. In total, 5,480 new-found genes were detected in the YFP genome, including 56 genes located in the newly identified centromere regions. Additionally, synteny blocks, comparative genomes, phylogenetic relationships, gene family expansion and selection pressure were studied in connection with the genomes of other related mammals. Significant improvements were observed in genome contiguity, the number of scaffolds and gaps, and annotation compared to the first drafts of the YFP (GCF_000442215) and EFP (GCA_026225855) genome assemblies. The Telomere-to-telomere gap-free reference genomes will support conservation genetics and population management for finless porpoises.
**Corresponding Author:**	Kai Liu, Ph.D<br>CAFS FFRC: Chinese Academy of Fishery Sciences Freshwater Fisheries Research Center<br>Wuxi, CHINA
**Corresponding Author Secondary Information:**	
**Corresponding Author's Institution:**	CAFS FFRC: Chinese Academy of Fishery Sciences Freshwater Fisheries Research Center
**Corresponding Author's Secondary Institution:**	
**First Author:**	Denghua Yin
**First Author Secondary Information:**	
**Order of Authors:**	Denghua Yin
	Chunhai Chen
	Danqing Lin
	Zhong Hua
	Congping Ying
	Jialu Zhang
	Chenxi Zhao

Powered by Editorial Manager® and ProduXion Manager® from Aries Systems Corporation

	Yan Liu
	Zhichen Cao
	Han Zhang
	Chenhe Wang
	Liping Liang
	Pao Xu
	Jianbo Jian
	Kai Liu, Ph.D
**Order of Authors Secondary Information:**	
**Additional Information:**	
**Question**	**Response**
Are you submitting this manuscript to a special series or article collection?	No
**Experimental design and statistics**<br><br>Full details of the experimental design and statistical methods used should be given in the Methods section, as detailed in our Minimum Standards Reporting Checklist. Information essential to interpreting the data presented should be made available in the figure legends.<br><br>Have you included all the information requested in your manuscript?	Yes
**Resources**<br><br>A description of all resources used, including antibodies, cell lines, animals and software tools, with enough information to allow them to be uniquely identified, should be included in the Methods section. Authors are strongly encouraged to cite Research Resource Identifiers (RRIDs) for antibodies, model organisms and tools, where possible.<br><br>Have you included the information requested as detailed in our Minimum Standards Reporting Checklist?	Yes
**Availability of data and materials**	Yes

All datasets and code on which the conclusions of the paper rely must be either included in your submission or deposited in publicly available repositories (where available and ethically appropriate), referencing such data using a unique identifier in the references and in the "Availability of Data and Materials" section of your manuscript.<br><br>Have you have met the above requirement as detailed in our Minimum Standards Reporting Checklist?	

Powered by Editorial Manager® and ProduXion Manager® from Aries Systems Corporation

Manuscript Click here to access/download;Manuscript;manuscript.docx

Telomere-to-telomere gap-free genome assembly of the endangered Yangtze finless porpoise and East Asian finless porpoise

Denghua Yin[1†], Chunhai Chen[2†], Danqing Lin[1†], Zhong Hua[1], Congping Ying[3], Jialu Zhang[1], Chenxi Zhao[2], Yan Liu[1], Zhichen Cao[4], Han Zhang[4], Chenhe Wang[2], Liping Liang[2], Pao Xu[1,3,*], Jianbo Jian[2,*] and Kai Liu[1,3,4,*]

[1]Key Laboratory of Freshwater Fisheries and Germplasm Resources Utilization, Ministry of Agriculture and Rural Affairs, Freshwater Fisheries Research Center, Chinese Academy of Fishery Sciences, Wuxi 214081, China.

[2]BGI Genomics, BGI-Shenzhen, Shenzhen 518083, China.

[3]Wuxi Fisheries College, Nanjing Agricultural University, Wuxi 214081, China.

[4]National Demonstration Center for Experimental Fisheries Science Education, Shanghai Ocean University, Shanghai 201306, China.

[†]Authors contributed equally to this work.

[*]Correspondence address: E-mail: xup@ffrc.cn, jianjianbo@bgi.com, liuk@ffrc.cn.

Abstract

The Yangtze finless porpoise (*Neophocaena asiaeorientalis asiaeorientalis*, YFP) and the East Asian finless porpoise (*Neophocaena asiaeorientalis sunameri*, EFP) are two subspecies of the narrow-ridged finless porpoise that live in fresh and salt water, respectively. They serve as ideal models for the study of freshwater adaptation and evolution. The main objective of this study was to provide contiguous chromosome-level genome assemblies for YFP and EFP. Here, we generated and upgraded genomes of YFP and EFP at the telomere-to-telomere level by combining PacBio HiFi long reads, ultra-long ONT reads and Hi-C sequencing data, with a total size of 2.48 Gb and 2.50 Gb respectively. The scaffold N50 of two genomes was 125.12 Mb (YFP) and 128 Mb (EFP) with one contig for one chromosome. The telomere repeat and centromere position were clearly identified in both YFP and EFP genomes. Telomeric repeat units were detected at 85% and 90% of the chromosome both ends in YFP and EFP genomes, respectively. In total, 5,480 new-found genes were detected in the YFP genome, including 56 genes located in the newly identified centromere regions. Additionally, synteny blocks, comparative genomes, phylogenetic relationships, gene family expansion and selection pressure were studied in connection with the genomes of other related mammals. Significant improvements were observed in genome contiguity, the number of scaffolds and gaps, and annotation compared to the first drafts of the YFP (GCF_000442215) and EFP (GCA_026225855) genome assemblies. The Telomere-to-telomere gap-free reference genomes will support conservation genetics and population management for finless porpoises.

Keywords: telomere-to-telomere; genome assembly; Yangtze finless porpoise; gap-free; HiFi sequencing; Hi-C sequencing

Introduction

Finless porpoises (*Neophocaena* spp.) are uniquely small toothed whales capable of inhabiting freshwater (Yangtze River) and saltwater (coastal waters of southern and eastern Asia) environments [1, 2]. They are characterized by a blunt, rounded head, an equal width upper and lower jaw, and lack of a clearly dorsal fin [3, 4]. Based on morphological characteristics, geographic distribution and molecular genetic evidence, it is generally believed that the finless porpoise can be divided into two species, namely the Indo-Pacific finless porpoise (*N. phocaenoides*) and the narrow-ridged finless porpoise (*N. asiaeorientalis*). In China, there exist two subspecies of the narrow-ridged finless porpoise: one is the freshwater population (Yangtze finless porpoise, *N. a. asiaeorientalis*), which exclusively inhabits the middle and lower reaches of the Yangtze River and adjacent Dongting and Poyang lakes; while the other is the marine population (East Asian finless porpoise, *N. a. sunameri*), which occurs in the coastal waters of the Yellow Sea and Bohai Sea, as well as the northern waters of the East China Sea[5, 6] (Figure 1A).

The investigation of the evolutionary origins and conservation genetics of finless porpoises is an urgent priority for scientists. Yang et al. identified significant genetic structure between the Indo-Pacific finless porpoise and the other two populations by analyzing the sequences of mtDNA control region of finless porpoises in Chinese waters [7]. This result was supported by subsequent mtDNA sequences, nuclear DNA microsatellites, single nucleotide polymorphisms (SNPs) and MHC loci [8-12]. The genetic diversity of East Asian finless porpoises surpasses that of the other two populations, indicating it as the likely center of origin for this species. Zheng et al. analyzed the sequences of the mtDNA control region of seven local populations of Yangtze finless porpoises in the middle and lower reaches of the Yangtze River, and found that the overall level of genetic diversity was low. Notably, the downstream population showed richer genetic variation than the midstream population. Such a genetic pattern reflects, to some extent, the marine origin and evolutionary history of the Yangtze population [13]. Based on genomic analysis of finless porpoise populations, significant genetic structure was identified among the three populations, indicating local adaptive evolution and emphasizing the evolutionary distinctiveness and conservation significance of the Yangtze finless porpoise [14].

Availability of high-quality genome assembly is not only critical for the genomic studies of

finless porpoises, but also would be a valuable resource for comparative genomics and evolutionary studies of cetacean. The first draft of the YFP genome assembly was published in 2018 with a size of 2.3 Gb, which was generated by short-read sequencing on the Illumina HiSeq 2000 platform [14]. However, this draft was highly fragmented and consisted of 104 scaffolds with an N50 of 6.3 Mb. Although progress had been made in genome-wide studies of YFP through the availability of this draft, such as immune changes with age and gene expression profiles in different habitats [15, 16], the lack of chromosomal information had led to some limitations in genomic studies of YFP. Recent advances in ultra-long ONT and PacBio HiFi sequencing technologies, as well as assembly algorithms, have facilitated the development of Telomere-to-telomere (T2T) genome assemblies. The completion of the T2T human genome sequence and the full Y chromosome sequence represents a significant milestone in the field of human genomics research, offering great potential for comprehensive genomic analysis in evolutionary studies [17, 18]. T2T genome has emerged as a hotspot genomic research fields now, extensive applications to other animal species such as chicken, fish [19, 20]. T2T genome assemblies can serve as a benchmark with enhanced accuracy and comprehensive genomic references for future studies, facilitating the confident identification and annotation of genes, regulatory elements, and other functional components.

High-quality genome can support finer genetic analyses, such as the length, number, and distribution of key indicators of inbreeding, such as ROH and IBD [21, 22], and these analyses are more urgent than ever for the conservation of endangered species. In this study, we utilized PacBio HiFi, Nanopore and Hi-C data to generate two improved telomere-to-telomere gap-free genomes of Yangtze and East Asian finless porpoises. We compared the quality of newly drafted assemblies with previously available versions and explored the synteny blocks, comparative genomes, phylogenetic relationships, gene family expansion and selection pressure in relation to the several mammals. Finless porpoises serve as a representative example for comprehending speciation, evolution, and population genetics. The high-quality chromosomal-level references facilitate the elucidation of adaptation mechanisms in aquatic mammals.

Results and Discussion

Genome sequencing and gap-free assembly

We integrated PacBio HiFi long reads, ultra-long ONT reads and Hi-C sequencing data to

generate chromosome-level genome assemblies for YFP and EFP. We generated approximately 123 Gb (49x) PacBio HiFi reads, 279 Gb (111x) Hi-C reads and 225 Gb (90x) ONT reads for YFP (Supplementary Table S1). Based on the previously sequenced 62x PacBio HiFi and 85x Hi-C reads of the EFP [15], we generated 215 Gb (86x) ONT reads in this study (Supplementary Table S1). The genome assembly of the YFP comprised 23 scaffolds, with both contig N50 and scaffold N50 measuring 125.12 Mb (Table 1). These scaffolds were assembled into 21 autosomal chromosomes, one X chromosome, and one mitochondrial chromosome, resulting in a final assembly size of 2.48 Gb (Supplementary Table S2). Similarly, the genome assembly for EFP consisted of 24 contigs or scaffolds, with both contig N50 and scaffold N50 measuring 128.00 Mb (Table 1). These scaffolds were also assembled into 21 autosomal chromosomes, one X+Y chromosome, and one mitochondrial chromosome, with a final assembly size of 2.50 Gb (Supplementary Table S2).

Compared to the YFP v1.0 (GCF_000442215) and EFP v1.0 (GCA_026225855) assemblies, we have significantly enhanced the contiguity, accuracy and completeness of these two genome assemblies. The contig N50 values of the two genomes were consistent with their respective chromosome lengths, and a single contig represented a complete chromosome, which is notably superior to the recently published finless porpoise genomes (e.g., 125.12 Mb vs. 0.09 Mb for YFP and 128.00 Mb vs 84.69 Mb for EFP) (Table 1). The YFP v1.0 and EFP v1.0 genome assemblies had 52,647 and 28 gaps, respectively, whereas in the new assembly we filled all the gaps and obtained a gap-free genome, greatly improving the contiguity of the assembled sequences (Figure 2A and Figure 2B). The Merqury estimated quality values of 60.18 and 64.38 based on k-mer analysis of YFP and EFP, respectively, which indicated that our assemblies were of high quality (Supplementary Table S3). Moreover, the mapping rates of RNA reads to the two genome assemblies were 95.47% and 95.42%, respectively, whereas they were 70.01% and 92.34%, correspondingly, for previously published assemblies (Supplementary Table S4). Using Benchmarking Universal Single-Copy Orthologs (BUSCO) evaluation, we achieved 95.20% completeness of YFP and 95.30% completeness of EFP (Table 1 and Figure 1E). Additionally, we have identified telomeric repeat units and centromere region in YFP and EFP (Figure 2A, Figure 2B and Supplementary Table S5-S6. Telomeric repeat units of YFP genome were detected at both ends of 18 chromosomes and at one end of 3 chromosomes. Similarly, telomeric repeat units of EFP genome were detected at both ends of 20 chromosomes and at one end of 2 chromosomes. Notably,

the centromere regions of each chromosome were predicted in the newly assembled YFP and EFP genomes, whereas the associated centromere sequences were not predicted in the first draft of YFP. The new genome assemblies of YFP and EFP were well-assembled without any gaps, achieving nearly telomere-to-telomere (T2T) completeness. Finally, we also utilized Hi-C data for chromosome sequencing and orientation (Figure 1C and Figure 1D).

Gene prediction and annotation

Two strategies including *de novo* and homolog-base methods were applied to annotate repeat elements. The genomes of YFP and EFP contained 1,058.09 Mb (42.54%) and 1,069.10 Mb (42.80%) of repetitive sequences, respectively (Supplementary Figure S1-S2 and Table S7). Long interspersed nuclear elements (LINEs) were the most abundant type of annotated transposable elements, constituting 38.88% and 39.10% of the genomes of YFP and EFP, respectively (Supplementary Table S8). For gene content assessment, 8 homologous proteins and 24 RNA-seq data were used (Supplementary Table S9-S10). In total, we predicted 23,139 and 23,101 protein-coding genes in the YFP and EFP genomes, respectively (Table 1), where the average length of coding sequence (CDS) was 1,507 bp and 1,510 bp, respectively. The average length of exon was both 175 bp, and the average length of intron was 6,082 and 6,107 bp, respectively (Supplementary Table S11-S12). The protein-coding genes in the YFP and EFP genomes were supported by at least one evidence with a CDS overlap ratio greater than 80% at a level of 99.96% and 99.95%, respectively (Supplementary Table S13-S14). It was worth noting that the length distribution of gene models at the levels of genes, CDS, exons and introns showed a similar trend when compared to those of YFP (GCF_000442215), EFP (GCA_026225855) and Bottlenose Dolphin (GCF_011762595) (Supplementary Figure S3). In the predicted gene models of YFP and EFP, the BUSCO analysis identified 97.5 % and 97.6% complete conserved single copy mammalian genes (odb10), respectively (Table 1 and Figure 1E). In total, 22,263 (96.21%) gene models in the YFP genome and 22,224 (96.20%) gene models in the EFP genome were annotated in at least one database (NR, SwissProt, KEGG, KOG, TrEMBL, Gene Ontology and InterPro) (Table 1, Figure 1F), whereas 71.22% (16,480) of YFP genes and 71.24% (16,457) of EFP genes are annotated in five functional databases (NR, SwissProt, KEGG, KOG and InterPro) (Supplementary Table S15 and Figure S4-S5). Finally, 20, 589 (88.98%) and 20, 613 (89.23%) could be transcriptionally detected by the 24 RNA-seq datasets (Figure 1F).

Analysis of centromere related genes

The centromere is an important functional structure of eukaryotic chromosomes, and plays an important role in ensuring the correct segregation of chromosomes during cell division. The mystery of the evolution of centromere structure among different species has not been fully revealed due to the challenge of assembling highly repetitive sequences [23]. In this study, we detected repeat monomers in the YFP and EFP genomes that may constitute the centromere (Figure 2A, Figure 2B and Supplementary Table S16-S17). In total, 235 and 237 genes were identified in the YFP and EFP candidate centromere region, respectively, while 56 and 20 genes of YFP and EFP were discovered in the newly identified centromere regions. We further compared the genes located in the centromere and non-centromere regions in the genomes of YFP and EFP, respectively, and found that there were no significant differences in the expression patterns of these genes in different transcripts (Figure 3A, Figure 3B and Supplementary Table S18-S19). Additionally, we analyzed the functional enrichment of genes located in the centromere regions of the two genomes (Supplementary Figure S6-S7). These genes were mainly involved in localization, locomotion, transcription receptor activity and cytoskeletal motor activity in GO enrichment (Figure 3C, Figure 3D and Supplementary Table S20-S23).

Variations between YFP and EFP genomes

Comparative analysis of the YFP and EFP genomes yielded numerous variations, including 3,887,060 single-nucleotide polymorphisms (SNPs) (Figure 4A) and 704,944 short insertions/deletions (Indels) (Figure 4B). The variations in SNPs were primarily located in the intergenic regions (65.28%) and intronic regions (32.66%), and rarely in the exon regions (0.64%). Similarly, variations in Indels were less concentrated in exon regions (0.23%) (Supplementary Table S24). Of the exon region variations, 12,203 SNPs (11,987 non-synonymous SNPs, 189 stop-gain SNPs, and 27 stop-loss SNPs) and 953 Indels (464 frameshift deletion, 467 frameshift insertion, 20 stop-gain Indels, and 2 stop-loss Indels) were identified and functionally associated with 6,158 and 582 genes, respectively. KEGG enrichment analysis revealed that these genes were significantly (P value $< =0.05$) enriched in “calcium signaling pathway”, “NF-kappa B signaling pathway”, “complement and coagulation cascades”, “antigen processing and presentation” and “glycerolipid metabolism” (Figure 4C and Supplementary Table S25).

Here, the genes coding for the mutated regions of the YFP and EFP are widely enriched in immune-related pathways, which may be closely related to their different habitats in freshwater and seawater, respectively. The microbial categories and pathogenicity of freshwater and seawater environments differ greatly, and the effects of pathogenic microorganisms on the organisms are also different. Therefore, the YFP and EFP will undergo adaptive evolution in order to adapt to the pathogen stresses of the two ecological environments, respectively, in freshwater and seawater. Alterations in a considerable number of immune-related genes may be important to facilitate the adaptation of YFP and EFP to freshwater and seawater habitats, respectively.

Specifically, by comparing the assembly results of the YFP and EFP versions, it was found that YFP assembled 5480 new genes, while EFP assembled 1453 new genes compared to the previous version of the assembly (Table 1 and Supplementary Table S26). These genes were expressed in all 24 samples (Supplementary Figure S8-S9 and Table S27-S28). GO functional enrichment analysis discovered that these genes are mainly enriched in cellular process, metabolic process, cellular anatomical entity, binding and catalytic activity (Figure 4D, Figure 4E, Supplementary Table S29-S32 and Figure S10-S11).

Phylogeny and synteny analysis

Gene family analysis was performed on 506,098 protein coding sequences from ten cetaceans and sixteen terrestrial mammals, and clustered into 22,196 gene families, which including 594 species-specific genes in YFP and EFP (Supplementary Table S7 and Table S33). A total of 2,161 single-copy orthologous genes were aligned using MAFFT (Supplementary Figure S12). Our analysis had revealed that the divergence time between the YFP and EFP ranges from 0.5 to 1.1 million years ago (Figure 5A, Figure 5B and Supplementary Figure S13), which is the first estimate at the molecular level since their classification as two distinct subspecies.

Synteny analysis demonstrated that YFP displayed a greater level of conservation than EFP (Figure 1B). We observed similar patterns in the distribution of gene frequency, gene density, TE density, and GC density between the two genomes, with most of their chromosomes being aligned with each other. Notably, certain chromosomes in the YFP genome were found to match multiple chromosomes in the EFP genome, indicating that chromosomal rearrangements occurred in both genomes after their speciation.

Gene family and positive selection analysis

We used CAFÉv4.0 to analyze the evolution of gene families based on orthologous clusters of protein coding sequences from twenty-six mammals. When comparing the genomes of YFP and EFP with their last common ancestor, it was found that 843 gene families expanded while 98 contracted (Figure 5A and Figure 5B). Out of the 215 expanded gene families identified in the YFP and EFP lineage, a total of 2,674 genes were found to be involved with statistical significance (P<0.05) (Supplementary Table S34). We observed an expansion for genes significantly enriched in several KEGG pathways, including "antigen processing and presentation", "intestinal immune network for IgA production", "oxidative phosphorylation" and "calcium signaling pathway" (Figure 5C). The significantly enriched GO terms, including "ferric iron binding", "iron ion transport", "riboflavin biosynthetic process", "tetrahydrofolate biosynthetic process" and "cytochrome-c oxidase activity", were also expanded (Figure 5D). We postulated that these genes played a crucial role in regulating osmotic pressure, enhancing immune resistance and facilitating hypoxic tolerance in finless porpoises, thereby reflecting potential mechanisms of adaptation to the aquatic environment. Further investigations are required to elucidate the specific functions of these gene families and their potential significance in the biology of finless porpoises.

The Codeml program in PAML with a branch-site model was employed for selective pressure analyses based on orthologous clusters of 10 cetaceans, including Yangtze finless porpoise, East Asian finless porpoise, Bottlenose dolphin, Killer whale, Yangtze River dolphin, Sperm whale, Minke whale, Bowhead whale, Beluga whale, Chinese white dolphin. We identified 41 positively selected genes (PSGs) in the YFP lineage, which were functionally enriched in "RNA degradation", "nucleotide excision repair", "DNA replication", "mismatch repair", and "homologous recombination" (P<0.05) (Figure 5E and Supplementary Table S35). The evolution of DNA damage repair pathways implied the existence of additional triggers for genomic instability in the Yangtze River, including human activities such as wading projects, dredging and quarrying. Interestingly, 44 PSGs in the EFP lineage were involved in "sodium-dependent phosphate transport", "sodium symporter activity", "aldosterone-regulated sodium reabsorption", and "calcium signaling pathway" (Figure 5E and Supplementary Table S36). Among these PSGs, six were potentially associated with the adaptation of EFP to high osmolarity environment, including Na(+)/H(+) exchange regulatory cofactor (*NHE-RF2*) and sodium-dependent phosphate cotransporter (*SLC34*). These indicated that

Yangtze and East Asian finless porpoises may possess distinct adaptation strategies to their aquatic environment, which warrant further investigation.

Methods

Sample collection, DNA extraction, and sequencing

We collected an adult dead female YFP sample from Lianzhou Lake, Anqing City, Anhui Province, China (N30°15′32″, E116°54′38″) in 2021 and a dead juvenile male EFP sample from the Yellow Sea near Lianyungang City, Jiangsu Province, China (N34°55′27″, E119°11′37″) in 2019 for sequencing (Figure 1A). No ethical considerations were taken into account in this study. DNA were extracted from muscle tissues following the phenol/chloroform DNA extraction method. DNA extracted from the YFP was utilized to construct PacBio HiFi, Hi-C, and Oxford Nanopore Technologies (ONT) libraries. DNA extracted from an EFP was utilized to construct an ONT library. According to the manufacturer's instructions (QIAGEN, Germany), a PacBio HiFi library was constructed using a QIAGEN Blood & Cell Culture DNA Midi Kit and subsequently sequenced on the PacBio Sequel II system in circular consensus sequence (CCS) mode. A Hi-C library was generated using the *Mbo* I restriction enzyme and subsequently sequenced on BGI MGISEQ platform. To generate and sequence ONT libraries, we isolated genomic DNA using the CTAB method, selected fragments exceeding 5 kb in size with the SageHLS HMW library system (Sage Science), processed the DNA with the Ligation sequencing 1D kit (SQK-LSK109, Oxford Nanopore Technologies, Oxford, UK), and subsequently sequenced the ONT libraries on a PromethION platform (Oxford Nanopore Technologies) at the BGI (Wuhan, China).

Gap-free genome assembly and quality assessment

We utilized SMRTLink v11.0.0 (https://www.pacb.com/support/software-downloads) to polish the PacBio HiFi reads, SOAPNUKE v2.0 [24] to filter the Hi-C reads, and self-designed Perl programs to refine the ONT reads. The errors in the cleaned ONT reads were subsequently corrected using the Necat pipeline. (v 20200119) [25]. To achieve gap-free chromosome-level assemblies, we employed Hifiasm (v0.15.1) [26] and Necat pipeline (v20200119) [25] to separately assemble PacBio HiFi reads and ONT corrected reads into the initial contigs. The Purge-Haplotigs [27] program was applied to remove redundant contigs with parameters “-j 80 -s 80 -a 30”. Juicer (v1.5) [28] and 3D-DNA (v180922) [29] pipeline for clustering, ordering, and orienting the contigs into

pseudo-chromosomes using Hi-C data. Additionally, we utilized ultra-long ONT reads and contigs to bridge the gaps in the gapless PacBio assembly through LR_Gapcloser (v1.0) [30] and TGSgapcloser (v 1.0.1)[31] pipeline.

Various methods were employed to evaluate the quality of the gap-free genome assemblies, including contiguity, correctness, and completeness. First, we calculated the length metrics of genomic sequences to evaluate contiguity and subsequently used Merqury (v1.3) [32] with k-mer set to 21 to assess the accuracy. Second, Benchmarking Universal Single-Copy Orthologs (BUSCO) [33] evaluation were conducted to assess the completeness. Third, we also mapped PacBio HiFi, ONT and RNA-seq data into the genome assemblies using Minimap2 [34] and Hisat2 (v2.1.0) [35] to assess the completeness. In addition, we utilized quartet pipeline [36] to search for telomere repeat sequences and centromere region in YFP and EFP.

Gene structure annotation

Repetitive sequences annotation was identified by *de novo* and homolog-base prediction. For *de novo* prediction, RepeatModeler (v1.0.4) [37] and LTR-FINDER (v1.0.7) [38] were conducted to identify repetitive elements and annotate long terminal repeats, respectively. For homolog-based prediction, DNA and protein transposable elements (TEs) were detected by RepeatMasker (v4.0.7) [39] and RepeatProteinMasker (v4.0.7), respectively. Tandem Repeat Finder (v4.10.0) [40] was used to identify Tandem repeats.

A combination of RNA-seq, homology-based and *de novo* prediction strategies was utilized to identify protein-coding genes in the genomes of YFP and EFP. RNA-seq data [15, 16] were mapped to genome assembly with Hisat2 v2.1.0 [41] with the following parameters: --sensitive --no-discordant --no-mixed -I 1 -X 1000 --max-intronlen 1000000. The produced BAM alignments were further assembled into gene models with StringTie v1.3.5 [42] with the following parameters: -f 0.3 -j 3 -c 5 -g 100 -s 10000 and validated using PASA v2.5.2 [43]. The coding sequences were identified by TransDecoder (v5.5.0) (https://github.com/TransDecoder/TransDecoder) with default parameters. Combing RNA-seq data with homolog sequences of eight cetaceans (Supplementary Table S7), the homology-like coding sequences were predicted using GeMoMa v1.9 [44]. One thousand high quality genes were randomly filtered out to train the predictors by Augustus v3.2.1 [45]. The Augustus v3.2.1 program was used to perform *de novo* prediction. We used GeMoMa software to integrate all predicted protein-coding genes, and annotated them with NR, Swissprot

[46], KEGG [47], KOG, TrEMBL, InterPro [48] and GO [49] databases.

Genome comparison

With the v2.0 genome assembly as a reference, we utilized the software MUMmer [50] and Syri (v1.6.3) [51] to explore the structural variations between the v1.0 and v2.0 genome assemblies. Genes were categorized as newly assembled if the gene region of first draft genome assembly exhibited a deletion of at least 50 bp and a minimum overlap of 30% within that region. The package ANNOVAR (v 2013-06-21) [52] was utilized to annotate the variations.

Identification of new assembled genes

The software Syri (v1.6.3) was employed to detect structural variations between the v2.0 genome assembly and the previously published genome assembly of finless porpoises. A gene was classified as newly assembled if the previously published genome assembly exhibited a deletion of at least 50 bp and the gene region had a minimum overlap of 30% with that region.

Gene family and phylogenomic analysis

Gene families of 26 species (Supplementary Table S7) were identified and clustered by OrthoFinder (v2.3.11) [53]. Single-copy orthologous genes (1:1:1) were aligned using MAFFT [54] (v7.310), and a maximum-likelihood phylogenetic tree was constructed with PhyML (v3.3) [55] using HKY85 model. Consistent phylogeny with previous study was demonstrated by all branches, as evidenced by 100/100 bootstrap support. [56]. Species divergence time was calculated using MCMCTREE in PAML (v4.9) [57]. Four divergence time points from TimeTree (http://timetree.org.cn) were used to calibrate the divergence times: (a) *Ornithorhynchus anatinus* and *Monodelphis domestica* (163.7–185.9 Ma), (b) *Homo sapiens* and *Mus musculus* (81.3–91.0 MYA), (c) *Balaena mysticetus* and *Balaenoptera acutorostrata* (21.3–28.8 Ma) and (d) *Sousa chinensis* and *Tursiops truncatus* (2.0–3.8 Ma). The core-orthologous gene sets were identified by Blast (v2.0.14) [58] with an E-value threshold of $1\times E^{-10}$ (at least 10 syntenic genes allowed), and defined syntenic blocks by MCscanX v1.5.2 [59]. Circos was used to plot the synteny results.

Gene family expansion and contraction analysis

Protein sequences of YFP, EFP and 24 published mammals were used to search homologs. Based on the gene families clustered by OrthoFinder, the CAFÉ (v4.0) [60] software was used to perform expansion and contraction analyses in branch of finless porpoises. Random birth and death models were employed to study gains and losses of gene families in a user-specified phylogeny. The global

parameter λ, which describes both the gene birth (λ) and death (μ = −λ) rate for gene families in all branches of the tree, was estimated using maximum likelihood. Then the P-value was calculated for each gene family, and P-value ≤0.01 was defined as a "significantly expanded and contracted gene family". KEGG and GO enrichment analyses were conducted among these significantly expanded and contracted gene family.

Gene positive analysis

Protein sequences of two finless porpoise and other 8 published cetaceans were used to identify single copy orthologs with OrthoFinder. Then Ka/Ks ratios for these single copy orthologs were calculated by following steps. Firstly, global alignment among these single copy orthologs was executed by PRANK and then filtered the alignment with Gblocks. Finally, Ka/Ks ratios on different branches was calculated by Codeml in the PAML package [57] with the free-ratio model. Genes that showed values of Ka/Ks higher than 1 along the branch, leading to finless porpoise were reanalyzed using the codon-based branch site tests implemented in PAML (PAML, RRID:SCR_014932). The branch site model allowed ω to vary both among sites in the protein and across branches, and it was used to detect episodic positive selection.

Gene expression analysis

The raw RNA-seq reads were quality controlled by SOAPnuke (v2.0), and the clean reads were subsequently aligned to the EFS v2.0 genome using Hisat2 (v2.1.0), with the following parameters: '--phred33 -p 5 --sensitive --no-discordant --no-mixed -I 1 -X 1000'. We utilized featureCounts [61] and transcripts per million (TPM) method to generate an estimated mapped read count matrix and calculate the gene expression level, respectively.

Conclusion

The availability of reliable chromosome-level genome assembly provides remarkable improvements in identifying genes, characterizing genomic regions and performing comparative genomic analyses. In the present study, we assembled two telomere-to-telomere and gap-free Yangtze finless porpoise and the East Asian finless porpoise genomes by combining PacBio long reads, Hi-C and short-read sequencing technologies. The new assemblies have higher contiguity and completeness, as well as more complete single-copy BUSCO genes with fewer fragmented or missing genes than the first drafts. Genome synteny analysis revealed a robust collinear relationship between the Yangtze finless porpoise and the East Asian finless porpoise. Reconstructing ancestral chromosomes enabled the identification of chromosomal rearrangement events in the finless porpoise. The reconstructed phylogeny determined that the YFP and EFP constitute a clade and diverged approximately 0.5-1.1 million years ago (Ma). Gene family expansion analysis revealed significantly enriched pathways and GO terms associated with the regulation of osmotic pressure, immune resistance, and hypoxic tolerance. Selection pressure analysis identified genes associated with DNA damage repair in the YFP and high salt tolerance in the EFP, respectively. The acquisition of the centromere, telomere, and associated genes can serve as valuable resources for comprehensively understanding chromosome stability, undesired recombination, repair mechanisms, and evolutionary processes. Overall, this is the most continuous genome assembly to date, with chromosome-scale contigs and no gaps. This study will lay a foundation for population genomics studies at the whole genome level, and deepen the scientific issues related to population conservation and adaptation mechanisms.

DATA AVAILABILITY

Raw sequencing data and genome assemblies in this study have been deposited in the NCBI database (BioProject ID PRJNA915046 and PRJNA859258). Furthermore, results of repeat annotation, gene structure annotation and gene functional annotation had been deposited in the *figshare* https://figshare.com/s/1fc632fd4f3cab36b776.

COMPETING INTERESTS

The authors declare that they have no competing interests.

AUTHORS' CONTRIBUTIONS

K.L., J.B.J. and P.X. designed and conceived the study. D.H.Y., C.P.Y. and J.L.Z. collected and prepared the samples. C.H.C. and C.X.Z. performed the data analysis. D.H.Y., C.H.C. and J.B.J. wrote the manuscript with significant contributions from Y.L., Z.C.C., H.Z., C.H.W. and L.P.L. K.L., Z.H. and D.Q.L. provided the financial support. All authors read and approved the final version of the manuscript.

ACKNOWLEDGMENTS

This work was funded by the National Key R&D Program of China (2021YFD1200304), the Central Public-interest Scientific Institution Basal Research Fund, Freshwater Fisheries Research Center, CAFS (2021JBFM15) and Project of Implementation of Yangtze Finless Porpoise Protection in the Middle and Lower Reaches of Yangtze River (2021).

REFERENCES

1. Gao, A and Zhou, K. Growth and reproduction of three populations of finless porpoise, *Neophocaena phocaenoides*, in Chinese waters. *Aquat Mamm* 1993;**19** (1):3-12.
2. Jefferson, T. Preliminary analysis of geographic variation in cranial morphometrics of the finless porpoise (*Neophocaena phocaenoides*). *Raffles Bull Zool* 2002;**10**:3-14.
3. Wang, P. The morphological characters and the problem of subspecies identifications of the finless porpoise. *Fish Sci* 1992;**11**:4-8.
4. Gao, A and Zhou, K. Geographical variation of external measurements and three subspecies of *Neophocaena phocaenoides* in Chinese waters. *Acta Theriol Sin* 1995;**15**(2):81-92.
5. Wang, J, Frasier, T, Yang, S, *et al*. Detecting recent speciation events: the case of the finless porpoise (genus *Neophocaena*). *Heredity* 2008;**101**(2):145-55.
6. Jefferson, T and Wang, J. Revision of the taxonomy of finless porpoises (genus *Neophocaena*): The existence of two species. *J Mar Anim Ecol* 2011;**4**(1):3-16.
7. Yang, G, Ren, W, Zhou, K, *et al*. Population genetic structure of finless porpoises, *Neophocaena phocaenoides*, in Chinese waters, inferred from mitochondrial control region sequences. *Marine mammal science* 2002;**18**(2):336-47.
8. Xu, S, Sun, P, Zhou, K, *et al*. Sequence variability at three MHC loci of finless porpoises (*Neophocaena phocaenoides*). *Immunogenetics* 2007;**59**(7):581-92.
9. Chen, M, Zheng, J, Wu, M, *et al*. Genetic diversity and population structure of the critically endangered Yangtze finless porpoise (*Neophocaena asiaeorientalis asiaeorientalis*) as revealed by mitochondrial and microsatellite DNA. *Int J Mol Sci* 2014;**15**(7):11307-23.
10. Chen, M, Fontaine, M, Chehida Y, *et al*. Genetic footprint of population fragmentation and contemporary collapse in a freshwater cetacean. *Sci Rep* 2017;**7**(1):14449.
11. Lin, W, Frère, C, Karczmarski, L, *et al*. Phylogeography of the finless porpoise (genus *Neophocaena*): testing the stepwise divergence hypothesis in the northwestern Pacific. *Sci Rep* 2014;**4**:6572.
12. Li, S, Xu, S, Wan, H, *et al*. Genome-wide SNP and population divergence of finless porpoises. *Genome Biol Evol* 2013;**5**(4):758-68.
13. Zheng, J, Xia, J, He, S, *et al*. Population genetic structure of the Yangtze finless porpoise (*Neophocaena phocaenoides asiaeorientalis*): implications for management and conservation. *Biochem Genet* 2005;**43**(5-6):307-20.
14. Zhou, X, Guang, X, Sun, D, *et al*. Population genomics of finless porpoises reveal an incipient cetacean species adapted to freshwater. *Nat Commun* 2018;**9**(1):1276.
15. Yin, D, Lin, D, Guo, H, *et al*. Integrated analysis of blood mRNAs and microRNAs reveals immune changes with age in the Yangtze finless porpoise (*Neophocaena asiaeorientalis*). Comp *Biochem Physiol B Biochem Mol Biol* 2021;**256**:110635.
16. Liu, W, Yin, D, Lin, D, *et al*. Blood Transcriptome Analysis Reveals Gene Expression Differences between Yangtze Finless Porpoises from Two Habitats: Natural and Ex Situ Protected Waters. *fishes* 2022;**7**:96.
17. Nurk, S, Koren, S, Rhie, A, *et al*. The complete sequence of a human genome. *Science* 2022;**376**(6588):44-53.
18. Rhie A, Nurk S, Cechova M, *et al*. The complete sequence of a human Y chromosome. *Nature* 2023;**621**(7978):344-54.
19. Huang, Z, Xu, Z, Bai, H, *et al*. Evolutionary analysis of a complete chicken genome. *Proc*

Natl Acad Sci U S A 2023;**120**(8):e2216641120.

20. Xue, L, Gao, Y, Wu, M *et al*. Telomere-to-telomere assembly of a fish Y chromosome reveals the origin of a young sex chromosome pair. *Genome Biol* 2021;**22**(1):203.
21. Zhang, L, Lan, T, Lin, C, *et al*. Chromosome-scale genomes reveal genomic consequences of inbreeding in the South China tiger: A comparative study with the Amur tiger. *Mol Ecol Resour* 2023;**23**(2):330-47.
22. Shukla, H, Suryamohan, K, Khan, A, *et al*. Near-chromosomal de novo assembly of Bengal tiger genome reveals genetic hallmarks of apex predation. *Gigascience* 2022;**12**:giac112.
23. Zhang, A, Kong, T, Sun, B, *et al*. A telomere-to-telomere genome assembly of Zhonghuang 13, a widely-grown soybean variety from the original center of Glycine max. *The Crop Journal* 2023. doi:https://doi.org/10.1016/j.cj.2023.10.003.
24. Chen Y, Chen Y, Shi C, *et al*. SOAPnuke: a MapReduce acceleration-supported software for integrated quality control and preprocessing of high-throughput sequencing data. *Gigascience* 2018;**7**(1):1-6.
25. Chen Y, Nie F, Xie S, *et al*. Efficient assembly of nanopore reads via highly accurate and intact error correction. *Nat Commun* 2021;**12**(1):60.
26. Cheng, H, Concepcion, G, Feng, X, *et al*. Haplotype-resolved de novo assembly using phased assembly graphs with hifiasm. *Nat Methods* 2021;**18**(2):170-5.
27. Roach, M, Schmidt, S and Borneman, A. Purge Haplotigs: allelic contig reassignment for third-gen diploid genome assemblies. *BMC bioinformatics* 2018;**19**(1):460.
28. Durand, N, Shamim, M, Machol, I, *et al*. Juicer Provides a One-Click System for Analyzing Loop-Resolution Hi-C Experiments. *Cell systems* 2016;**3**(1):95-8.
29. Dudchenko, O, Batra, S, Omer, A, *et al*. De novo assembly of the Aedes aegypti genome using Hi-C yields chromosome-length scaffolds. *Science* 2017;**356**(6333):92-5.
30. Xu, G, Xu, T, Zhu, R, *et al*. LR_Gapcloser: a tiling path-based gap closer that uses long reads to complete genome assembly. *Gigascience* 2019;**8**(1):giy157.
31. Xu M, Guo L, Gu S, *et al*. TGS-GapCloser: A fast and accurate gap closer for large genomes with low coverage of error-prone long reads. *Gigascience* 2020;**9**(9):giaa094.
32. Rhie, A, Walenz, B, Koren, S, *et al*. Merqury: reference-free quality, completeness, and phasing assessment for genome assemblies. *Genome Biol* 2020;**21**(1):245.
33. Waterhouse, R, Seppey, M, Simão, F, *et al*. BUSCO Applications from Quality Assessments to Gene Prediction and Phylogenomics. *Mol Biol Evol* 2018;**35**(3):543-8.
34. Li, H. Minimap2: pairwise alignment for nucleotide sequences. *Bioinformatics* 2018;**34** (18):3094-100.
35. Kim, D, Paggi, J, Park, C, *et al*. Graph-based genome alignment and genotyping with HISAT2 and HISAT-genotype. *Nat Biotechnol* 2019;**37**(8):907-15.
36. Lin, Y, Ye, C, Li, X, *et al*. quarTeT: a telomere-to-telomere toolkit for gap-free genome assembly and centromeric repeat identification. *Hortic Res* 2023;**10**(8):uhad127.
37. Chen N. Using RepeatMasker to identify repetitive elements in genomic sequences. *Curr Protoc Bioinformatics* 2004;**Chapter 4**:Unit 4.10.
38. Xu, Z and Wang, H. LTR_FINDER: an efficient tool for the prediction of full-length LTR retrotransposons. *Nucleic Acids Res* 2007;**35**:W265-8.
39. Price, A, Jones, N and Pevzner, P. De novo identification of repeat families in large genomes. *Bioinformatics* 2005;**21**(Suppl 1):i351-8.

40. Benson, G. Tandem repeats finder: a program to analyze DNA sequences. *Nucleic Acids Res* 1999;**27**(2):573-80.
41. Kim, D, Langmead, B and Salzberg, S. HISAT: a fast spliced aligner with low memory requirements. *Nat Methods* 2015;**12**(4):357-60.
42. Kovaka, S, Zimin, A, Pertea, G, *et al*. Transcriptome assembly from long-read RNA-seq alignments with StringTie2. *Genome Biol* 2019;**20**(1):278.
43. Haas, B, Salzberg, S, Zhu W, *et al*. Automated eukaryotic gene structure annotation using EVidenceModeler and the Program to Assemble Spliced Alignments. *Genome Biol* 2008;**9**(1):R7.
44. Keilwagen, J, Hartung, F and Grau, J. GeMoMa: Homology-Based Gene Prediction Utilizing Intron Position Conservation and RNA-seq Data. *Methods Mol Biol* 2019;**1962**:161-77.
45. Stanke, M and Waack, S. Gene prediction with a hidden Markov model and a new intron submodel. *Bioinformatics* 2003;**19**(Suppl 2):ii215-25.
46. Bairoch, A and Apweiler, R. The SWISS-PROT protein sequence data bank and its supplement TrEMBL. *Nucleic Acids Res* 1997;**25**(1):31-6.
47. Kanehisa, M, Sato, Y, Kawashima, M, *et al*. KEGG as a reference resource for gene and protein annotation. *Nucleic Acids Res* 2016;**44**(D1):D457-62.
48. Jones, P, Binns, D, Chang, H, *et al*. InterProScan 5: genome-scale protein function classification. *Bioinformatics* 2014;**30**(9):1236-40.
49. Ashburner, M, Ball, CA, Blake, J, *et al*. Gene ontology: tool for the unification of biology. The Gene Ontology Consortium. *Nat Genet* 2000;**25**(1):25-9.
50. Marçais, G, Delcher, A, Phillippy, A, *et al*. MUMmer4: A fast and versatile genome alignment system. *PLoS Comput Biol* 2018;**14**(1):e1005944.
51. Goel, M, Sun, H, Jiao, W, *et al*. SyRI: finding genomic rearrangements and local sequence differences from whole-genome assemblies. *Genome Biol* 2019;**20**(1):277.
52. Wang, K, Li, M and Hakonarson, H. ANNOVAR: functional annotation of genetic variants from high-throughput sequencing data. *Nucleic Acids Res* 2010;**38**(16):e164.
53. Emms, D and Kelly, S. OrthoFinder: phylogenetic orthology inference for comparative genomics. *Genome Biol* 2019;**20**(1):238.
54. Nakamura, T, Yamada, K, Tomii, K, *et al*. Parallelization of MAFFT for large-scale multiple sequence alignments. *Bioinformatics* 2018;**34**(14):2490-2.
55. Guindon, S, Delsuc, F, Dufayard, J, *et al*. Estimating maximum likelihood phylogenies with PhyML. *Methods Mol Biol* 2009;**537**:113-37.
56. Yuan, Y, Zhang, Y, Zhang, P, *et al*. Comparative genomics provides insights into the aquatic adaptations of mammals. *Proc Natl Acad Sci U S A* 2021;**118**(37):e2106080118.
57. Yang, Z. PAML 4: phylogenetic analysis by maximum likelihood. *Mol Biol Evol* 2007;**24** (8):1586-91.
58. Altschul, S, Gish, W, Miller, W, *et al*. Basic local alignment search tool. *J Mol Biol* 1990;**215**(3):403-10.
59. Wang, Y, Tang, H, Debarry, J, *et al*. MCScanX: a toolkit for detection and evolutionary analysis of gene synteny and collinearity. *Nucleic Acids Res* 2012;**40**(7):e49.
60. Bie, T, Cristianini, N, Demuth, J, *et al*. CAFE: a computational tool for the study of gene family evolution. *Bioinformatics* 2006;**22**(10):1269-71.

61. Liao, Y, Smyth, G and Shi, W. featureCounts: an efficient general purpose program for assigning sequence reads to genomic features. *Bioinformatics* 2014;**30**(7):923-30.

Tables

Table 1. Genome assembly statistics of Yangtze and East Asian finless porpoises

	Yangtze finless porpoise v2.0	Yangtze finless porpoise v1.0	East Asian finless porpoise v2.0	East Asian finless porpoise v1.0
Total size of assembled genome (Gb)	2.48	2.27	2.50	2.50
Contig N50 (Mb)	125.12	0.09	128.00	84.69
Contig N90 (Mb)	83.62	0.02	80.21	29.54
Number of contigs	23	47,942	24	52
Scaffold N50 (Mb)	125.12	6.34	128.00	122.40
Scaffold N90 (Mb)	83.62	1.13	80.21	80.21
Scaffolds number	22	13,695	23	23
Number of base chromosomes	22	22	23	23
Number of gap-free chromosomes	22	0	23	7
Number of gaps	0	52,647	0	28
Number of telomeres (pairs/single)	20/2	0/0	21/2	21/2
Number of estimated centromeres	22	0	23	23
TE size	42.54%	NA	42.80%	42.23%
GC content	41.70%	41.00%	41.70%	41.70%
BUSCO (Genome)	C:95.2%	C:94.0%	C:95.3%	C:95.4%
Gene Number	23,139	18,479	23,101	22,814
New-found gene number	5,480	NA	1,453	NA
Functional proteins	96.21%	NA	96.20%	97.31%
BUSCO (Protein)	C:97.5%	C:94.6%	C:97.6%	C:97.9%
Data source	This study	GCF_000442215	This study	GCA_026225855

Note: “v2.0” indicated the new genome assembly and annotation generated in this study; “v1.0” indicated previously published first draft of genome assembly and annotation. “C” indicated the percentage of complete BUSCO evaluation. “pairs/single”: “pairs” indicated that the telomeres were found at both ends of the chromosomes; “single” indicated that the telomeres were found only at the one end of chromosomes. “New-found genes” indicated that the genes were predicted in the extra sequence segments from the current assembly and were annotated in the current assembly.

Figure legends

Figure 1. Genome analysis and quality assessment of Yangtze and East Asian finless porpoises v2.0.

A: Location distribution and sampling site of the Yangtze and East Asian finless porpoises.

B: Synteny analysis of Yangtze finless porpoise and East Asian finless porpoise v2.0 genomes: a) chromosomes length; b) frequency of genes; c) density of genes; d) repeat density; e) GC density and f) syntenic blocks between Yangtze and East Asian finless porpoises.

C: Heat map displaying Hi-C interactions of Yangtze finless porpoises v2.0.

D: Heat map displaying Hi-C interactions of East Asian finless porpoises v2.0.

E: BUSCO assessments exhibiting proportions classified as Complete and single-copy (S, blue), Complete and duplicated (D, green), Fragmented (F, yellow), and Missing (M, red) categories.

F: Proportions of genes that could be functionally annotated and transcriptionally detected in Yangtze and East Asian finless porpoises v2.0.

Figure 2. T2T-resolved assembly of Yangtze and East Asian finless porpoises v2.0.

A: Structure of T2T and gap-free chromosomes in Yangtze finless porpoises v2.0. All 21+X chromosomes of Yangtze finless porpoises v2.0 are drawn to scale and the ruler indicates chromosome length. Triangles indicate the presence of telomere sequence repeats. Circles represent the locations of centromeric regions. The gap positions in the Yangtze finless porpoises v1.0 genome assembly are marked with squares corresponding to the right side of the chromosome in the Yangtze finless porpoises v2.0 genome assembly.

B: Structure of T2T and gap-free chromosomes in East Asian finless porpoises v2.0. All 21+X/Y chromosomes of East Asian finless porpoises v2.0 are drawn to scale and the ruler indicates chromosome length. Triangles indicate the presence of telomere sequence repeats. Circles represent the locations of centromeric regions. The gap positions in the East Asian finless porpoises v1.0 genome assembly are marked with squares corresponding to the right side of the chromosome in the East Asian finless porpoises v2.0 genome assembly.

Figure 3. Expression patterns and functional enrichment of genes in the centromere region.

A: Heatmaps of the gene expression levels in centromere region and non-centromere region of Yangtze finless porpoises v2.0.

B: Heatmaps of the gene expression levels in centromere region and non-centromere region of East Asian finless porpoises v2.0.

C: GO enrichment analysis of genes in centromere region of Yangtze finless porpoises v2.0.

D: GO enrichment analysis of genes in centromere region of East Asian finless porpoises v2.0.

Figure 4. Structure variant between Yangtze and East Asian finless porpoises v2.0 genome assembly with Yangtze finless porpoises v2.0 genome assembly for reference.

A: The density plot of SNPs between Yangtze and East Asian finless porpoises v2.0 genome assembly.

B: The density plot of Indels between Yangtze and East Asian finless porpoises v2.0 genome assembly.

C: KEGG enrichment analysis of genes located in SNP and Indel region of Yangtze finless porpoises v2.0 genome assembly. Blue bar charts indicate the genes located in Indel region; while red bar charts indicate the genes located in the SNP region.

D: GO enrichment of new-found genes of Yangtze finless porpoise v2.0.

E: GO enrichment of new-found genes of East Asian finless porpoise v2.0.

Figure 5. Genome evolution of Yangtze and East Asian finless porpoises.

A: Divergence time between Yangtze finless porpoises and East Asian finless porpoises, and number of expanded and contracted gene families. green and red numbers indicate gene family expansions and contractions, respectively. MRCA: Most Recent Common Ancestor. Ma: Million years ago.

B: A comparison of gene families associated with orthologs and paralogs in Yangtze finless porpoises and East Asian finless porpoises and other 24 mammal species.

C: Significant KEGG and GO enrichment of expanded gene families in Yangtze and East Asian finless porpoise lineage.

D: KEGG enrichment analysis of positively selected genes in Yangtze finless porpoises (Neaa) and East Asian finless porpoise (Neas), respectively.

Figure 1

Click here to access/download;Figure;Figure 1.pdf

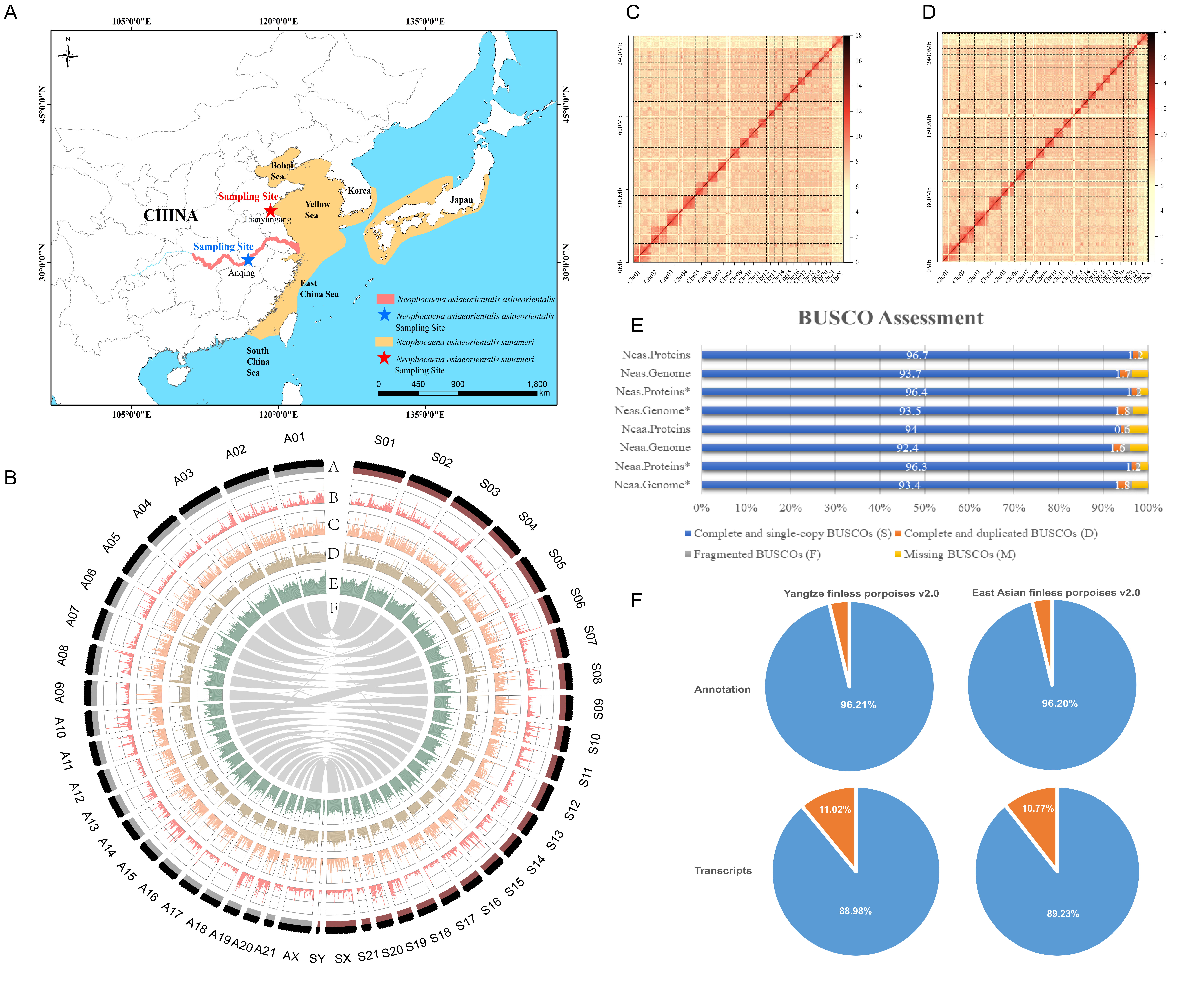

Figure 2

Click here to access/download;Figure;Figure 2.pdf

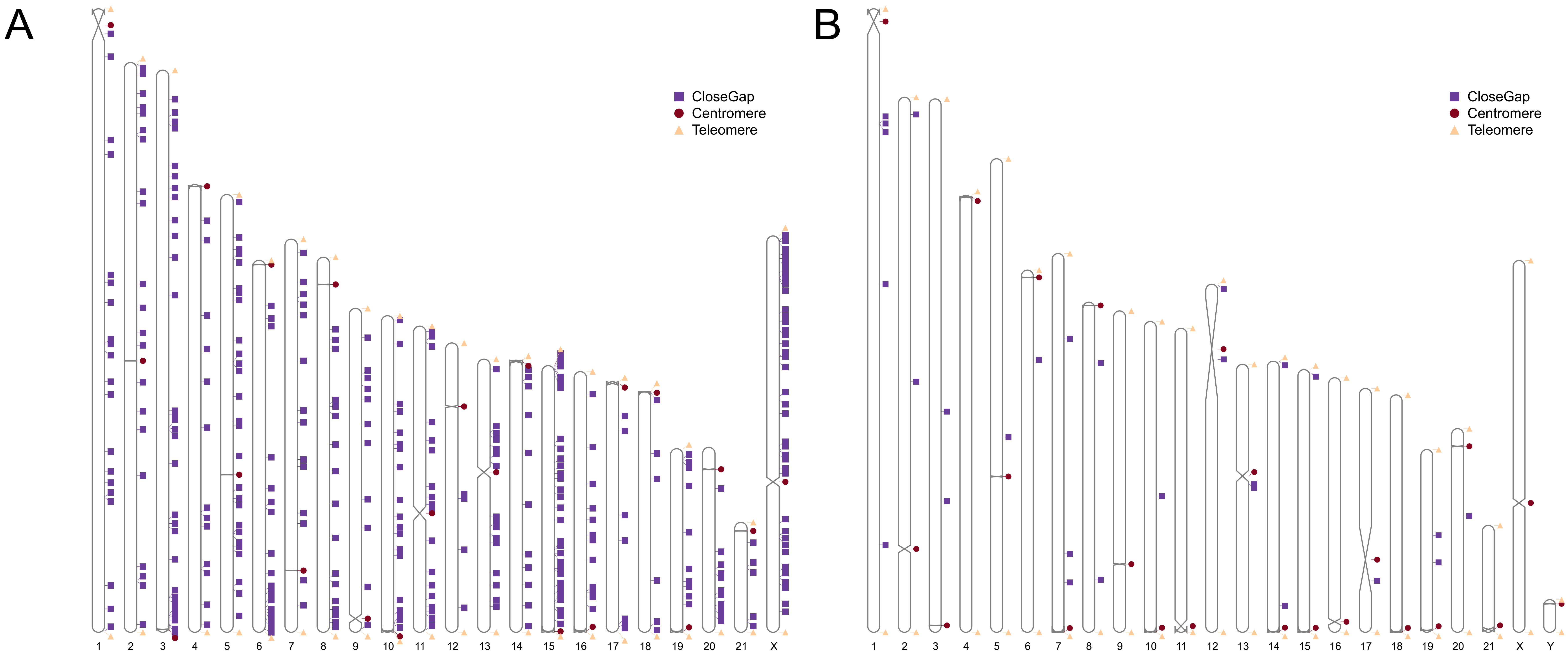

Figure 3

Click here to access/download;Figure;Figure 3.pdf

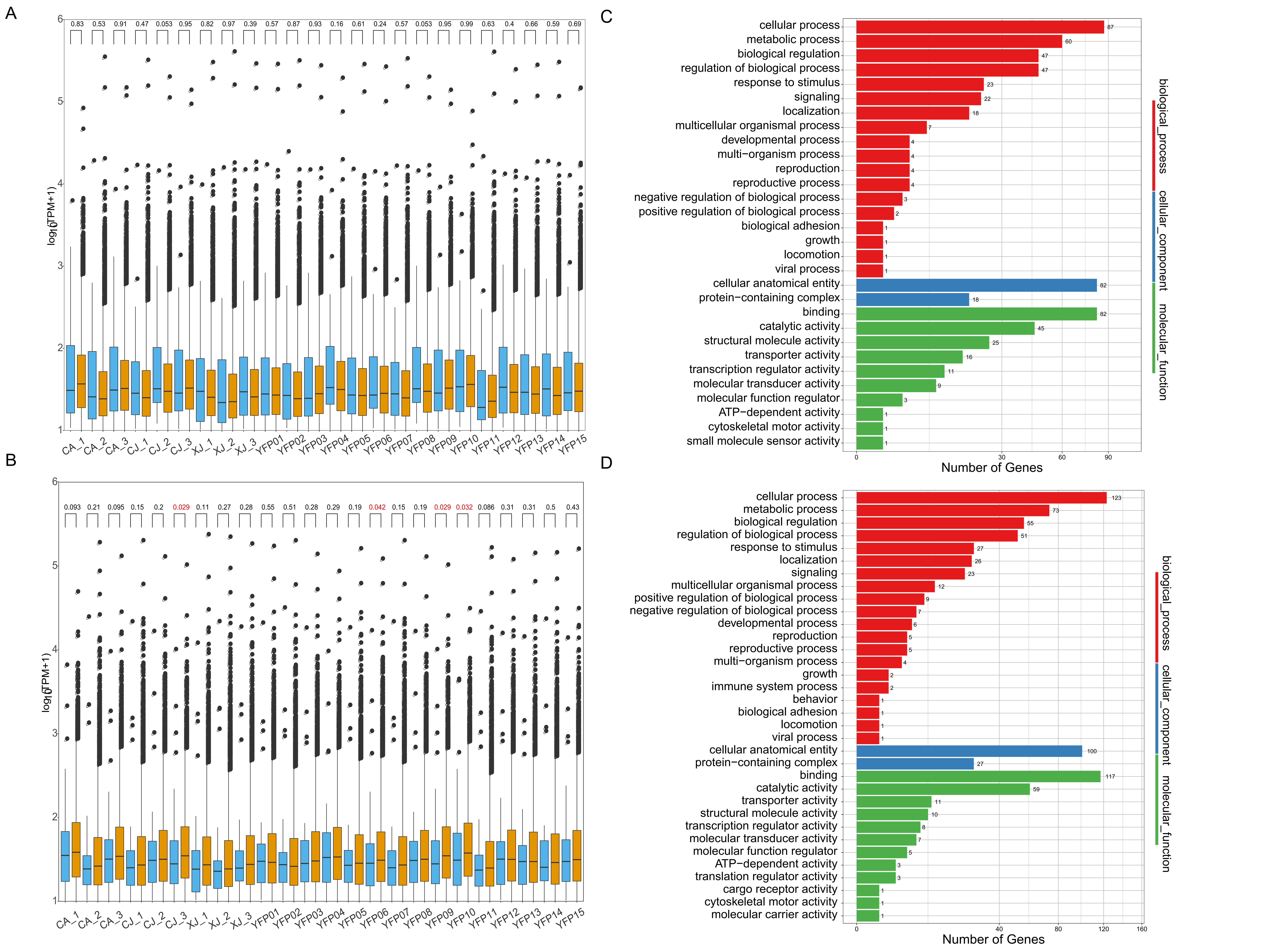

Click here to access/download;Figure;Figure 4.pdf

Figure 4

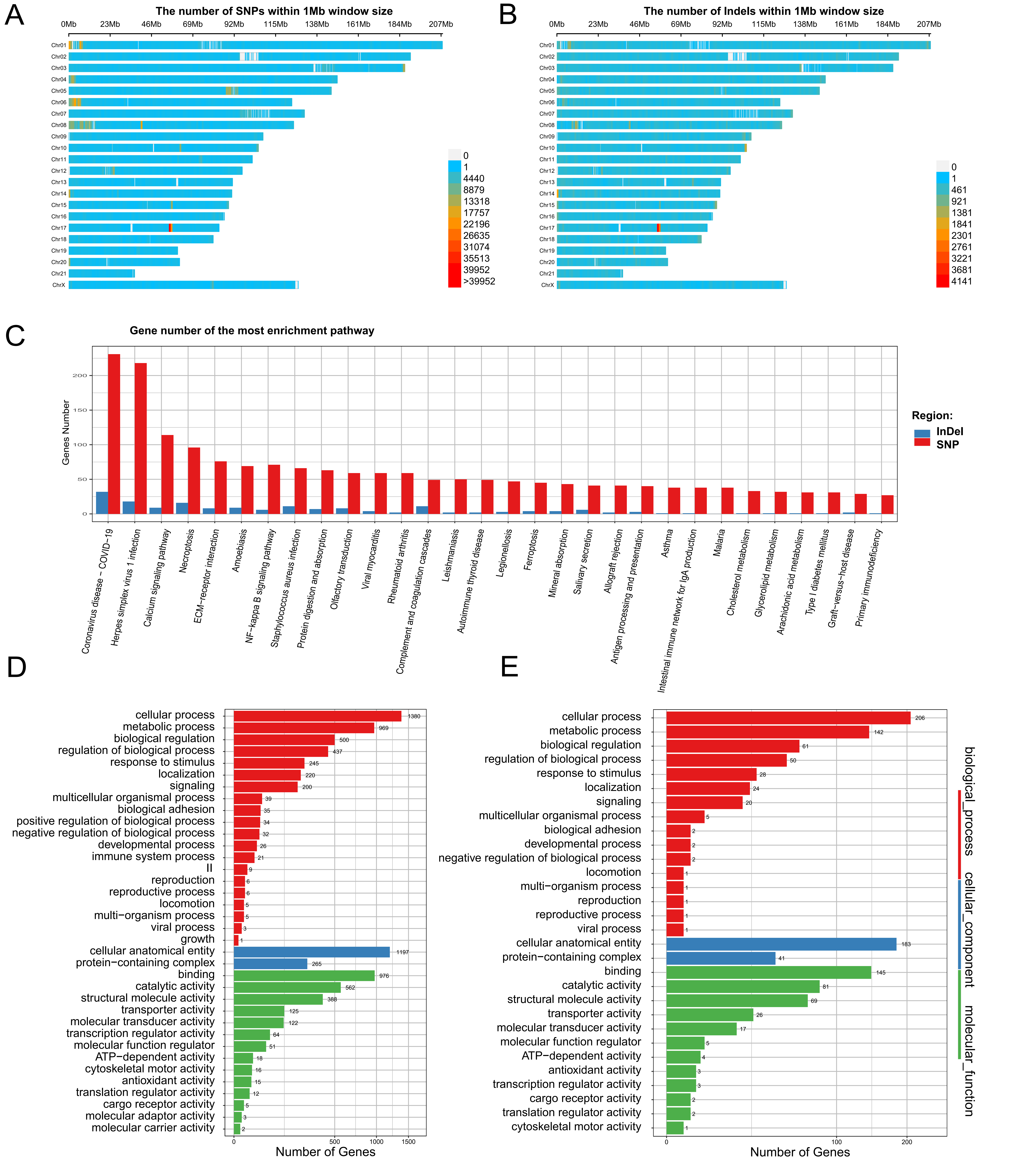

II: biological process involved in interspecies interaction between organisms

Figure 5
Click here to access/download;Figure;Figure 5.pdf

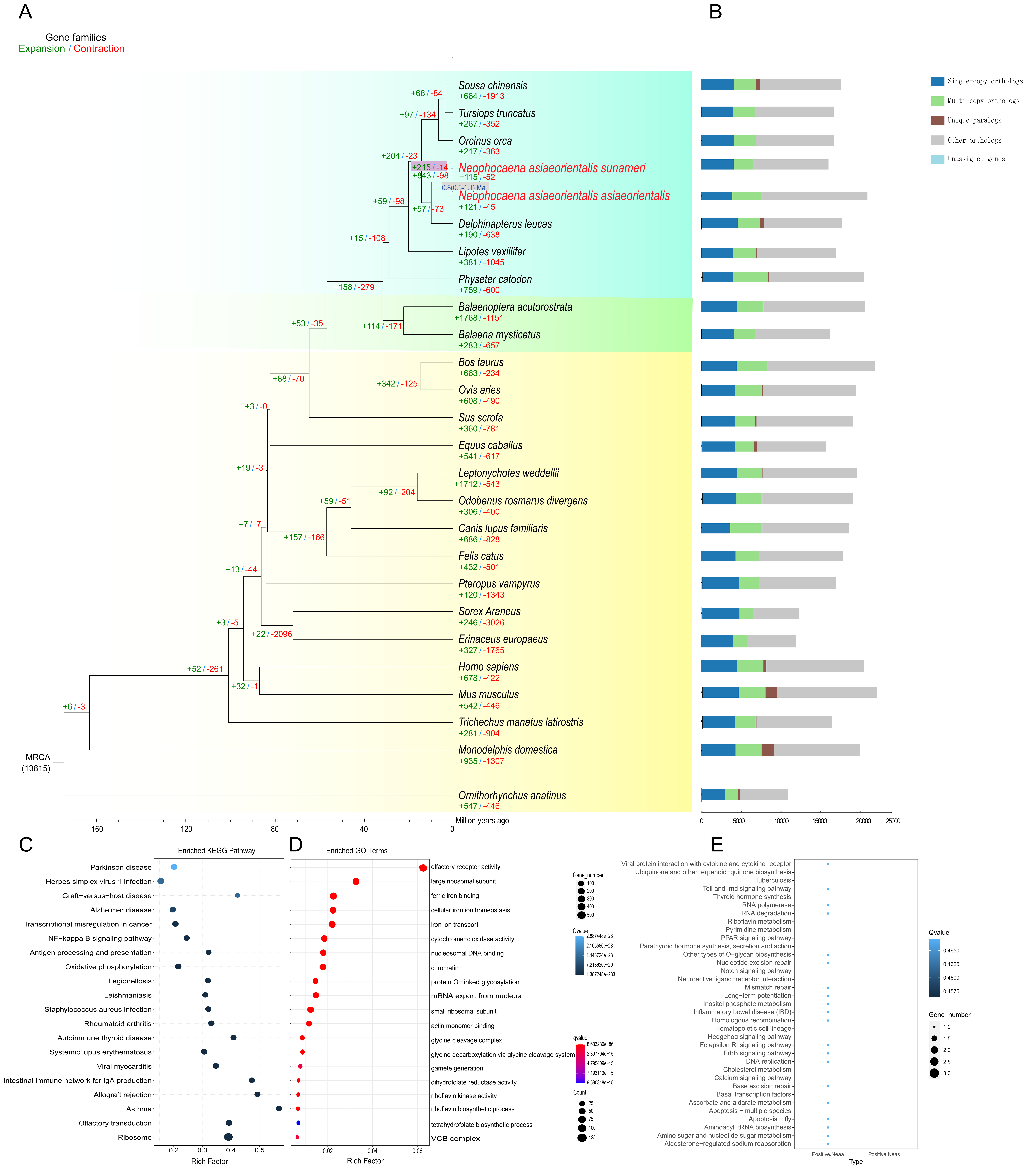

Click here to access/download
Supplementary Material
Supplementary Materials.docx

Supplementary Table S18

Click here to access/download
Supplementary Material
Supplementary Table S18.xls

Supplementary Table S19

Click here to access/download
Supplementary Material
Supplementary Table S19.xls

Supplementary Table S20

Click here to access/download
Supplementary Material
Supplementary Table S20.xls

Supplementary Table S21

Click here to access/download
Supplementary Material
Supplementary Table S21.xls

Supplementary Table S22

Click here to access/download
Supplementary Material
Supplementary Table S22.xls

Supplementary Table S23

Click here to access/download
Supplementary Material
Supplementary Table S23.xls

Supplementary Table S25

Click here to access/download
Supplementary Material
Supplementary Table S25.xls

Supplementary Table S27

Click here to access/download
Supplementary Material
Supplementary Table S27.xls

Supplementary Table S28

Click here to access/download
Supplementary Material
Supplementary Table S28.xls

Click here to access/download
Supplementary Material
Supplementary Table S29.xls

Supplementary Table S30

Click here to access/download
Supplementary Material
Supplementary Table S30.xls

Supplementary Table S31

Click here to access/download
Supplementary Material
Supplementary Table S31.xls

Supplementary Table S32

Click here to access/download
Supplementary Material
Supplementary Table S32.xls
