# Supplementary material for: Telomere-to-telomere gap-free genome assembly of the endangered Yangtze finless porpoise and East Asian finless porpoise
Source: Gigascience. 2024 Sep 16;13:giae067. doi: 10.1093/gigascience/giae067 (PMC11403816; doi:10.1093/gigascience/giae067)
Supplement: giae067_Response_to_Reviewer_Comments_Original_Submission [file giae067_response_to_reviewer_comments_original_submission.pdf]

Author's Response To Reviewer Comments

Dear Dr. Hans Zauner,
Thanks for your kind consideration and comments regarding to our manuscript entitled “Telomere-to-telomere gap-free genome assembly of the endangered Yangtze finless porpoise and East Asian finless porpoise” (GIGA-D-23-00359). We have revised our manuscript (details are provided below) in accordance with all comments and suggestions from the reviewers. We hope the revised manuscript have been improved and meet the standard requirement. We look forward to hearing from GigaScience soon.

Sincerely yours,

Correspondence: Kai Liu
Freshwater Fisheries Research Center, Chinese Academy of Fishery Sciences, Wuxi 214081, China.
E-mail: liuk@ffrc.cn

Response to reviewer 1's comments:
Reviewer #1: The authors have assembled new telomer-to-telomer reference genomes for two finless porpoise species, the fresh-water Yangtze finless porpoise and the oceanic East Asian finless porpoise. These genome assemblies appear to be ultra-high quality, with substantial description of the genomic features (repeats, genes, assembly characteristics), improvements over previous assemblies for the same species, and comparison of the genomes for some features including genomic structure, variation, novel genes and genes under selection. These represent substantial contributions to cetacean genomics and to understanding the evolution of these two sister species in their unique habitats.
The manuscript requires substantial revision to provide adequate methods for the reader to understand how the genomes were assembled and analyzed, and the figures need to be modified substantially to be useful. As they are, the legends do not adequately describe what's in many of the figures, the figures often try to present too much information in a single plot or set of plots, and the text is often too small to read. In my detailed comments below, I suggest ways to modify the figures and legends to make them more informative and useful.
The manuscript is generally well written, but requires some editing for standard English, and several sections repeat information presented in tables and figures, and could be edited to reduce length and focus on interpretation rather than just repetition. In particular, although the improvements in the new genome assemblies are substantial compared to previous assemblies, these improvements are a normal process in genome assembly, and not of great biological importance. I suggest reducing description and discussion of the upgrades to the genomes, and focus on the interesting characteristics of, and comparisons between, the two new genomes.
Response: Thanks for your valuable suggestions on our work. The comments are providing important guiding significance to our research. Based on the comments we received, detailed modifications are provided below. According to your advice, this manuscript was edited for proper English language, grammar, punctuation, spelling, and overall style by one native English speaker.
Specific comments (by line number)
Comment 1:28. change 'the chromosome both ends' to 'the chromosomes at both ends'
Response: We are sorry for the mistake. We have changed 'the chromosome both ends' to 'the chromosomes at both ends' in the revised manuscript.

Comment 2:31. 'selection pressure' was not studied, only inference of selection.
Response: Thank you very much for your suggestion. We have revised 'selection pressure' to 'inference of selection'.

Comment 3:33. The accession ID given for YFP, GCF000442215, is for Lipotes vexillifer, not for the YFP genome.
Response: We are sorry for the mistake. We have changed 'GCF000442215' to 'GCA_003031525.2'

Comment 4:43. is there a word missing between 'clearly' and 'dorsal fin? Or, change from 'a clearly dorsal

fin' to 'an obvious dorsal fin'.
Response: Thank you very much for your suggestion. We have revised 'a clearly dorsal fin' to 'an obvious dorsal fin'.

Comment 5:54. It's not clear what you mean by 'populations', since the comparisons are among species and subspecies. The term 'population' means something different from species and subspecies, so this section should be revised to use the appropriate terms.
Response: Thank you very much for your suggestion. We have revised the sentence 'Yang et al. identified significant genetic structure between the Indo-Pacific finless porpoise and the other two populations…' to 'Yang et al. identified significant genetic structure between either the Yangtze River population or the Yellow Sea population and the South China Sea population…' in the revised manuscript.

Comment 6:67. Insert 'a' before 'high-quality genome'
Response: We have inserted 'a' before 'high-quality genome' in the revised manuscript.

Comment 7:69. change 'cetacean' to 'cetaceans'
Response: We have change 'cetacean' to 'cetaceans' in the revised manuscript.

Comment 8:70. delete 'which was'
Response: We have deleted 'which was' in the revised manuscript.

Comment 9:71. The number of scaffolds for the previous version of the genome is misleading. The number given, 104, is based only scaffolds that are larger than the N50 length. According to the supplemental materials for Zhou et al. 2018, there were actually 97,387 scaffolds >100bp, 2179 scaffolds >2kb, and 423 scaffolds larger than the N90 size. If not all scaffolds are going to be counted, then it's important to specify which scaffolds were counted.
Response: Thank you for bringing this to our attention. We have made revisions in the description of the initial genome assembly of YFP. As reported on the NCBI website (https://www.ncbi.nlm.nih.gov/datasets/genome/GCF_003031525.1/), the assembly comprised 13,698 scaffolds, with a scaffold N50 of 6.3 Mb, excluding the minimum sequence length (100 bp) consideration.

Comment 10:80. change to 'a hotspot of genomic research, with extensive applications…'
Response: We have changed '…a hotspot genomic research fields now, extensive applications…' to '…a hotspot of genomic research, demonstrating extensive applications…'

Comment 11:81. insert 'and' between 'chicken' and 'fish'.
Response: We have inserted 'and' between 'chicken' and 'fish'.

Comment 12:99. change 'Based on' to 'In addition to'
Response: We have changed 'Based on the previously sequenced 62x PacBio HiFi and 85x Hi-C reads of the EFP, we generated 215 Gb (86x) ONT reads in this study' to 'In this study, we supplemented the existing dataset comprising 62 × PacBio HiFi and 85 × Hi-C reads of the EFP with an additional 215 Gb (86 ×) of ONT reads'.

Comment 13:116. What do the Merqury quality values mean? Are these from a range (e.g., 1-100), or do they represent a log value? The reader can't interpret this without some context, and you can't assume they are familiar with every program.
Response: The quality values obtained from Merqury's k-mer analysis for YFP v2.0 and EFP v2.0 were calculated as 60.18 and 64.38, respectively. These values indicate a foundational accuracy level of 99.999%, confirming the high quality of our assembly for each component. It is essential to emphasize that Merqury quality values span from 0 to 255, with higher values denoting superior quality.
Ref:
Rhie, A, Walenz, B, Koren, S, et al. Merqury: reference-free quality, completeness, and phasing assessment for genome assemblies. Genome Biol 2020;21(1):245.

Comment 14:133-156. The whole section on gene prediction and annotation results could be reduced so that it repeats less of what is already presented in Table 1. Simple summary values such as average length

of coding sequences don't need to be presented in the results, especially if they are not discussed in the discussion section as being important.
Response: Thank you for your suggestion concerning 'Gene prediction and annotation' section. We have revised the text with simple summary values at lines 163-172 in the revised MS to minimize redundancy and emphasize the main discoveries. The revised section now offers a succinct overview of the predicted protein-coding genes in the YFP and EFP genomes, supported by evidence and a comparison of length distributions. Supplementary Tables 9-12 contain further information, such as the average lengths of coding sequences, exons, and introns, as recommended.
We have changed 'In total, we predicted 23,139 and 23,101 protein-coding genes in the YFP and EFP genomes, respectively (Table 1), where the average length of coding sequence (CDS) was 1,507 bp and 1,510 bp, respectively. The average length of exon was both 175 bp, and the average length of intron was 6,082 and 6,107 bp, respectively (Supplementary Table S11-S12). The protein-coding genes in the YFP and EFP genomes were supported by at least one evidence with a CDS overlap ratio greater than 80% at a level of 99.96% and 99.95%, respectively (Supplementary Table S13-S14). ' to 'In total, the number of predicted protein-coding genes was 23,139 in the YFP v2.0 genome and 23,101 in the EFP v2.0 genome (Table 1). The roughly comparable number of predicted protein-coding genes for both T2T genomes is further evidence supporting the gene models (Supplementary Table S9-S12).'

Comment 15:138. The sentence starting 'For gene content assessment…' should be in the methods, not results. It's also not clear what you mean by '8 homologous proteins' (proteins, or databases, or ???). Also, change 'RNAseq data' to 'RNAseq datasets' (as in line 156).
Response: We apologize for any inconvenience caused. What I meant to convey is: "Utilizing 149,956 genes from 8 closely related cetacean species and transcriptomic sequencing data from 24 Yangtze finless porpoises as input files, the GeMoMa software was employed to conduct homology-based prediction analysis."

Comment 16:143-145. The sentence starting with 'The protein-coding genes in…' is difficult to understand, and I'm not sure it's important. Does it belong in the methods, or does it say something about the quality of the gene identification?
Response: We have revised the text with simple summary values at lines 163-172 in the revised MS to minimize redundancy and emphasize the main discoveries.
We have changed 'In total, we predicted 23,139 and 23,101 protein-coding genes in the YFP and EFP genomes, respectively (Table 1), where the average length of coding sequence (CDS) was 1,507 bp and 1,510 bp, respectively. The average length of exon was both 175 bp, and the average length of intron was 6,082 and 6,107 bp, respectively (Supplementary Table S11-S12). The protein-coding genes in the YFP and EFP genomes were supported by at least one evidence with a CDS overlap ratio greater than 80% at a level of 99.96% and 99.95%, respectively (Supplementary Table S13-S14). ' to 'In total, the number of predicted protein-coding genes was 23,139 in the YFP v2.0 genome and 23,101 in the EFP v2.0 genome (Table 1). The roughly comparable number of predicted protein-coding genes for both T2T genomes is further evidence supporting the gene models (Supplementary Table S9-S12).'

Comment 17:147. The wrong accession ID is given for the YFP, and I don't understand why the accession numbers are being presented here at all, since this whole section is about the new YFP and EFP genomes.
Response: We are sorry for the mistake. We have corrected the accession ID and changed the sentence to 'It is worth noting that the length distribution of gene models at the levels of genes, CDS, exons and introns showed a similar trend when compared to those of YFP v1.0 (GCA_003031525.2), EFP v1.0 (GCA_026225855.1) and Bottlenose Dolphin (GCF_011762595).'

Comment 18:155. 'Finally, 20, 589 (88.98%) and 20, 613 (89.23%)' of what? Genes? It's unclear what you are referring to for these numbers.
Response: We are sorry for the unclear description. We have changed 'Finally, 20,589 (88.98%) and 20,613 (89.23%) could be transcriptionally detected by the 24 RNA-seq datasets.' to 'Finally, 20,589 (88.98%) and 20,613 (89.23%) genes from the YFP v2.0 and EFP v2.0 genomes, respectively, were determined to be transcriptionally active based on the analysis of 24 RNA-seq datasets.'

Comment 19:162. What are 'repeat monomers'? is this a particular sequence that is unique to centromere repeats? One more sentence to describe what you are using to infer centromeres would be useful.

Response: We are sorry for the unclear description. We have added the description: 'In this study, we conducted predictions on repeat monomers within the YFP v2.0 and EFP v2.0 genomes, potentially constituting the centromere. The monomeric sequences vary in length from 99 to 201 bp, with the 144-bp, 150-bp, and 138-bp monomers being the most abundant. Centromeres are composed of more than one repeat monomer and are located within TE- and TR-enriched regions, which are areas with relatively lower gene density.'

Comment 20:165. Does 'newly identified centromere regions' refer to regions that are newly discovered in centromeres, or newly discovered in the new genome assemblies (compared to the old ones)? The sentence is a little confusing.
Response: We are sorry for the unclear description. We have changed 'In total, 235 and 237 genes were identified in the YFP and EFP candidate centromere region, respectively, while 56 and 20 genes of YFP and EFP were discovered in the newly identified centromere regions.' to 'A total of 235 and 237 genes were identified in the candidate centromere regions for YFP v2.0 and EFP v2.0, respectively, through predictions generated by centromere-finding software. Moreover, the newly discovered centromere regions for YFP v2.0 hosted approximately 56 genes, while only 20 genes were found in analogous regions for EFP v2.0. The "newly identified centromere regions" refer to specific areas identified in the genome that have been recently assembled but were not included in the previously published version. This discovery may suggest the presence of novel, previously uncharacterized centromere sites.'

Comment 21:179-180. please explain what 'stop-gain SNPs' and 'stop-loss SNPs' are. I think this means SNPs that result in the gain or loss of a stop codon, but I don't know the specific term (jargon).
Response: I apologize for any inconvenience caused. In genetics, these terms mean:
'stop-gain SNPs' (nonsense): A mutation that results in the premature termination of protein synthesis by introducing a premature stop codon, leading to the production of a truncated and often non-functional protein.
'stop-loss SNPs': A mutation where a stop codon is lost, allowing the translation machinery to continue synthesizing the protein beyond its intended termination point.

Comment 22:183. Why did you identify these 5 pathways from Figure 4C? Were they the only ones with functional variants, or are they just random examples from all of the ones in Figure 4C?
Response: We are sorry for the unclear description. We have modified the description of these pathways: 'KEGG enrichment analysis revealed that these genes were significantly (P value < =0.05) enriched in "NF-kappa B signaling pathway", "complement and coagulation cascades", "antigen processing and presentation" and "Intestinal immune network for IgA production"'.
In addition, we have enriched the discussion on these pathways: "The genes coding for the mutated regions of the YFP and EFP are widely enriched in immune-related pathways. This association may be intricately linked to the distinct pathogenic microorganisms unique to freshwater and seawater environments. Marine mammals exhibit a diminished histocompatibility complex (MHC) diversity attributed to decreased encounters with microparasitic diversity in their marine habitat relative to their terrestrial origin. This phenomenon implies that mammals encounter distinct pathogenic pressures in varied ecological settings, potentially influencing the evolution of immune-related genes. [1]. Evolutionary analyses of the innate immune pattern recognition receptor (TLRs) in the YFP and the marine finless porpoise indicate that the YFP has undergone specific adaptive changes [2]. The microbial diversity and pathogenicity of freshwater and seawater environments vary, leading to distinct effects of pathogenic microorganisms on the organisms in these two types of environments [3]. Therefore, the YFP and EFP would be expected to undergo adaptive evolution to adapt to the pathogen stresses specific to their respective ecological environments, freshwater and seawater."
Ref
[1] Slade, R and McCallum, H. Overdominant vs. frequency-dependent selection at MHC loci. Genetics 1992;132(3):861–64.
[2] Tian, R, Chen, M, Chai, S, et al. Divergent Selection of Pattern Recognition Receptors in Mammals with Different Ecological Characteristics. J Mol Evol 2018;86(2):138-49.
[3] Lokesh, J and Kiron, V. Transition from freshwater to seawater reshapes the skin-associated microbiota of Atlantic salmon. Sci Rep 2016;25(6):19707.

Comment 23:186. Delete 'Here', and start the sentence with 'The genes coding…'

Response: Thank you very much for your suggestion. We have deleted 'Here' in the revised manuscript.

Comment 24:190. change 'will' to 'would be expected to'
Response: Thank you very much for your suggestion. We have changed 'will' to 'would be expected to'.

Comment 25:192-193. Delete the last sentence of the paragraph, as it is just repeating what is said above.
Response: Thank you very much for your suggestion. We have deleted the repeated sentence.

Comment 26:194. Delete 'Specifically,' and start the sentence with 'By comparing…' .
Response: Thank you very much for your suggestion. We have deleted 'Specifically,' and start the sentence with 'By comparing…' in the revised manuscript.

Comment 27:195. change 'YFP assembled 5480 new genes, while EFP assembled 1453…' to 'The YFP assembly included 5480 new genes, while the EFP assembly included 1453…'
Response: Thank you very much for your suggestion. We have corrected it in the revised manuscript.

Comment 28:198. Change 'discovered' to 'indicated'
Response: Thank you very much for your suggestion. We have changed 'discovered' to 'indicated' in the revised manuscript.

Comment 29:203. Change 'including' to 'include'
Response: Thank you very much for your suggestion. We have changed 'including' to 'include' in the revised manuscript.

Comment 30:209. Insert 'structural' or 'chromosomal' before 'conservation'
Response: Thank you very much for your suggestion. We have changed 'Synteny analysis demonstrated that YFP displayed a greater level of conservation than EFP' to 'Synteny analysis of the gene order between YFP v2.0 and EFP v2.0 revealed 907 large shared syntenic blocks, encompassing 89.59% (41428) genes, and 17 chromosomal rearrangements' in the revised manuscript.

Comment 31:209-214. Figure 1B should be its own figure, and changed to include only the synteny information. It's too small and too dense to be useful as it is. The full figure can be presented in supplemental materials. The text describing the figure could be expanded to describe what was found rather than just general observations of 'similar patterns' gene frequency, density, etc., or it could be reduced to just focus on what was different (specific chromosome rearrangements).
Response: Thank you for the valuable suggestions provided. We have separated subfigures A and F from Figure 1B into individual new Figure 3. The remaining subfigures have been consolidated to create a new figure, now referred to as Supplemental Figure S7.

Comment 32:218. What do you mean by the 'last common ancestor'? This 'ancestor' doesn't exist, so is it a reconstructed ancestral genome?
Response: We apologize for the error in the description. We have changed 'When comparing the genomes of YFP and EFP with their last common ancestor, it was found that 843 gene families expanded while 98 contracted. 'to 'Upon comparing the genomes of YFP v2.0 and EFP v2.0 with their most recent common ancestor, it was observed that 843 gene families underwent expansion, while 98 gene families experienced contraction.'
When studying the evolution of gene families, the root of the phylogenetic tree is typically described using the term "Most Recent Common Ancestor" (MRCA), indicating the point of origin for these gene families during the evolutionary process, where they initially diversified into different genes. This term emphasizes the shared origin among different members of the gene family and their relationship to the common ancestor.

Comment 33:220. What does 'involved with statistical significance' mean?
Response: We are sorry for the unclear description. We have changed 'Out of the 215 expanded gene families identified in the YFP and EFP lineage, a total of 2,674 genes were found to be involved with statistical significance ($P<0.05$)' to 'Among the 215 expanded gene families identified in the YFP v2.0 and EFP v2.0 lineage, a total of 2,674 genes were determined to be significantly associated ($P<0.05$)'.

Comment 34:232-234. The first time these species are mentioned, the Latin name needs to be included. In the methods, only the Latin names are used, so the reader can't easily know what species the common names refer to (e.g. the Chinese white dolphin is called the Indo-Pacific humpback dolphin in the Society for Marine Mammalogy's taxonomy list).
Response: We are sorry for the unclear description. We have supplemented the Latin names in the sentence 'Neophocaena asiaeorientalis asiaeorientalis (Yangtze finless porpoise), Neophocaena asiaeorientalis sunameri (East Asian finless porpoise), Tursiops truncatus (Bottlenose dolphin), Orcinus orca (Killer whale), Lipotes vexillifer (Yangtze River dolphin), Physeter catodon (Sperm whale), Balaenoptera acutorostrata (Minke whale), Balaena mysticetus (Bowhead whale), Delphinapterus leucas (Beluga whale), Sousa chinensis (Indo-Pacific humpback dolphin)'.
In addition, we have added the common names in the methods '(a) Ornithorhynchus anatinus (Platypus) and Monodelphis domestica (Opossum) (163.7–185.9 Ma), (b) Homo sapiens (Human) and Mus musculus (Mouse) (81.3–91.0 MYA), (c) Balaena mysticetus and Balaenoptera acutorostrata (21.3–28.8 Ma) and (d) Sousa chinensis and Tursiops truncatus (2.0–3.8 Ma).'

Comment 35:237-239. What evidence is there for the statement that 'The evolution of DNA damage repair pathways implied the existence of additional triggers for genomic instability in the Yangtze River, including human activities such as wading projects, dredging and quarrying'? It seems odd to invoke pressure from the last century or less for evolutionary changes at the gene pathway level.
Response: We are sorry for the mistake. We have revised and expanded upon this paragraph: ' The selective pressure to evolve DNA damage repair pathways implied that the Yangtze finless porpoise might be experiencing increased threats to genome stability. The mechanism of DNA damage repair plays a crucial role in preserving genome integrity by enabling cells to identify and repair DNA damage, thereby averting the accumulation of harmful mutations [1]. In a comparative genomic analysis between the South China tiger and the Amur tiger, it was noted that genes related to DNA repair underwent positive selection in the South China tiger [2]. The observed phenomenon could be explained by the higher probability of genome instability in the temperate and subtropical habitats of the South China tiger. This may be linked to metabolites generated by intestinal microflora, which possess the ability to trigger DNA damage [3]. The stability of the genome or gene expression system in the Yangtze finless porpoise across different organs and life stages remains uncertain. However, one potential interpretation of this data is the suggestion that the Yangtze finless porpoise could be vulnerable to genomic instability triggers in the Yangtze River, such as water pollutants, which may increase the likelihood of DNA damage [4]. Pollutants found in the Yangtze River possess the capacity to accumulate within the food chain, leading to cellular DNA damage and impacting the genome stability of the Yangtze finless porpoise [5-6]. This makes a compelling case for the improved conservation of the species and the development of more rigorous water pollution mitigation practices.'
Ref
[1] Chatterjee, N and Walker, G. Mechanisms of DNA damage, repair, and mutagenesis. Environ Mol Mutagen 2017;58(5):235-63.
[2] Zhang, L, Lan, T, Lin, C, et al. Chromosome-scale genomes reveal genomic consequences of inbreeding in the South China tiger: A comparative study with the Amur tiger. Mol Ecol Resour 2023;23(2):330-47.
[3] Puschhof, J and Sears, C. Microbial metabolites damage DNA. Science 2022;378(6618):358-59.
[4] Lv, W, Gu, H, He, D, et al. Polystyrene nanospheres-induced hepatotoxicity in swamp eel (Monopterus albus): From biochemical, pathological and transcriptomic perspectives. Sci Total Environ 2023;893:164844.
[5] Zhang, K, Qian, Z, Ruan, Y, et al. First evaluation of legacy persistent organic pollutant contamination status of stranded Yangtze finless porpoises along the Yangtze River Basin, China. Sci Total Environ 2020;710:136446.
[6] Xiong, X, Qian, Z, Mei, Z, et al. Trace elements accumulation in the Yangtze finless porpoise (Neophocaena asiaeorientalis asiaeorientalis)-A threat to the endangered freshwater cetacean. Sci Total Environ 2019;686:797-804.

Comment 36:256-257. The 'QIAGEN Blood & Cell Culture DNA Midi Kit' appears to be a DNA extraction kit, not a library preparation kit, so it cannot have been used to construct PacBio HiFi libraries. This section needs more detail to specify exactly what kits or methods were used to construct libraries, as the library methods impact downstream DNA sequence processing.

Response: Thanks for the suggestion. We have modified and enriched the methods: 'The PacBio HiFi library was constructed using SMRTbell Prep Kit 3.0 (Pacific Biosciences, USA) and subsequently sequenced on the PacBio Sequel II system in circular consensus sequence (CCS) mode. To collect data for the Hi-C library, the muscle tissues were first fixed in 1% formaldehyde (Sigma) for cross-linking and resuspended in lysis buffer. Then, MboI (NEB) restriction endonucleases were used to fragment the chromatin in the muscle to fragment DNA. The DNA fragments were captured by utilizing Streptavidin-coated magnetic beads (Thermo Fisher SCIENTIFIC) following biotin labeling and crosslinking using T4 DNA Ligase (ENZYMATICS). The Hi-C library was finally sequenced on a BGI MGISEQ platform.'

Comment 37:261. Cite a reference for the 'CTAB method'
Response: Thanks for bringing this to our attention. We have cited a reference for the 'CTAB method'.
Ref:
Yan, M, Wei, G, Pan, X et al. A method suitable for extracting genomic DNA from animal and plant-modified CTAB method. Agric Sci Technol, 2008, 9(2):39-41.

Comment 38:267. What filters were used to 'filter the Hi-C reads? Are there specific quality filters, or read trimming that are needed?
Response: Thank you for drawing our attention to this. To filter Hi-C reads, index reads were removed and reads were filtered using the following SOAPNUKE v2.0 parameters: N rate >= 0.01, low quality <= 20, low quality rate >= 0.1. Correspondingly, we have amended the relevant descriptions in the revised MS at line 406-408.

Comment 39:268. What do you mean by 'refine' the ONT reads? This is a meaningless term, and since the Perl scripts are described as 'self-designed', this provides no information about what was done. Likewise, the statement that 'ONT reads were subsequently corrected' is also too vague. What was actually done to 'correct' the reads?
Response: Thank you for your valuable suggestions. ONT reads were filtered based on a length < 5 kb and a quality value < 7. The Necat pipeline (v 20200119) was utilized for the enhancement of ONT reads. This was achieved through the application of error correction algorithms to evaluate quality scores, k-mer frequencies, and alignment methods. This process enhanced the accuracy of reading and generated refined results suitable for further analyses. Correspondingly, we have amended the relevant descriptions in the revised MS at line 408-414.

Comment 40:272. There are a lot of programs used to process data, and while it's good that the actual parameters are given for some of them, it would be useful to also state whether the parameters were the default parameters or modified for these data, and describe what the parameter do (e.g., what does '-j 80 -s 80 -a 30' do?)
Response: Thank you for your valuable suggestions. The Purge-Haplotigs program was utilized to eliminate redundant contigs that exhibited similar sequences but distinct haplotypes, specifically targeting those with aligned coverage below 30%. This strategic approach significantly optimizes the assembly process by removing redundant information, thus improving the accuracy of genome assembly. Correspondingly, we have amended the relevant descriptions in the revised MS at line 416-420.

Comment 41:275. What do you mean by 'bridge the gaps in the gapless PacBio assembly? Do you mean that ONT reads and contigs were used to bridge gaps in the contig assembly in order to generate the gapless scaffolds?
Response: We are sorry for the unclear description. We have changed the sentence to ' Ultra-long ONT reads and contigs were used to generate gapless scaffolds through the LR_Gapcloser (v1.0) and TGSgapcloser (v 1.0.1) pipelines.'

Comment 42:281. Change 'were' to 'was'
Response: We have changed 'were' to 'was' in the revised manuscript.

Comment 43:283-4. insert 'the' before 'quartet pipeline' and change 'region' to 'regions'.
Response: We have inserted 'the' before 'quartet pipeline' and changed 'region' to 'regions'.

Comment 44:287. Change 'conducted' to 'used'

Response: We have changed '…RepeatModeler (v1.0.4) and LTR-FINDER (v1.0.7) were conducted to identify repetitive elements and annotate long terminal repeats, respectively.' to '…RepeatModeler (v1.0.4) was employed to identify repetitive elements, whereas LTR-FINDER (v1.0.7) was used for the specific annotation of long terminal repeats.' in the revised manuscript.

Comment 45:294. If RNAseq data were mapped to both assemblies, then change 'assembly' to 'assemblies'.
Response: We have changed 'assembly' to 'assemblies' in the revised manuscript.

Comment 46:299. I think 'combing' should be changed to 'combining'
Response: We have changed 'combing' to 'combining'.

Comment 47:320. Change 'Consistent phylogeny with previous study' to 'A phylogeny consistent with a previous study'
Response: We have changed 'Consistent phylogeny with previous study' to 'A phylogeny consistent with a previous study'.

Comment 48:327. Change 'Blast' to 'BLAST'
Response: We have changed 'Blast' to 'BLAST'.

Comment 49:328. It's unclear what you mean by 'defined syntenic blocks by MCscanX'. Do mean that 'syntenic blocks were defined using MCscanX'?
Response: We have changed 'defined syntenic blocks by MCscanX' to 'syntenic blocks were defined using MCscanX'

Comment 50:332. delete 'branch of', or change to 'analysis in the finless porpoise clade'.
Response: We have changed 'analysis in branch of finless porpoise' to 'analysis in the finless porpoise clade'

Comment 51:336. should the meaning of P-value≤0.01 be 'significantly expanded and contracted gene families', or is it either expanded OR contracted 'significantly expanded or contracted gene families'?
Response: We have changed 'significantly expanded and contracted gene families' to 'significantly expanded or contracted gene families'.

Comment 52:338. Change 'family' to 'families'
Response: We have changed 'family' to 'families'.

Comment 53:339. Change the section title to 'Gene positive selection analysis'
Response: We have changed the section title to 'Gene positive selection analysis'.

Comment 54:343. What filtering of alignments was done with Gblocks?
Response: Thank you for drawing our attention to this. Alignment refinement via Gblocks was utilized to remove inadequately aligned positions and divergent regions, thereby isolating conserved blocks from the multiple alignment. Correspondingly, we have amended the relevant descriptions in the revised MS at line 528-530.

Comment 55:345. delete the comma after 'branch'
Response: We have deleted the comma after 'branch'.

Comment 56:350. 'quality controlled' is vague. What was done to the reads to make them 'clean'? Please include the parameters or steps, e.g., reads were trimmed to remove adapter sequences and base calls with quality <30 (or whatever your parameters were).
Response: Thank you for your valuable suggestions. The raw RNA-seq reads underwent quality control using SOAPnuke (v2.0). Reads were filtered out if they had an N rate ≥ 0.01, low quality ≤ 20, low quality rate ≥ 0.1, or contained index sequences. Subsequently, the clean reads were aligned to the EFS v2.0 genome utilizing the Hisat2 high sensitivity model, excluding discordant pairs and mixed alignments. The alignment process involved setting a minimum insert size of 1 bp and a maximum insert size of 1000 bp.

Correspondingly, we have amended the relevant descriptions in the manuscript at line 539-543.

Comment 57:357. change 'assembly' to 'assemblies'
Response: We have change 'assembly' to 'assemblies'.

Comment 58:359. delete 'two' (not needed because you name the species genomes in the same sentence)
Response: We have deleted 'two'.

Comment 69:372. What is 'undesired recombination'? Undesired by whom? I think you can just delete that word, and say the genome data can serve as a valuable resources for understanding… recombination, etc.
Response: We have deleted 'undesired '.

Comment 60:373. change 'Overall, this is the most continuous genome assembly to date' to 'Overall, these are the most continuous cetacean genome assemblies to date'
Response: We have changed 'Overall, this is the most continuous genome assembly to date' to 'Overall, these are the most continuous cetacean genome assemblies to date'.

Comment 61:375. change 'deepen the scientific issues' to 'deepen the scientific understanding of issues'
Response: We have changed 'deepen the scientific issues' to 'deepen the scientific understanding of issues'.

Comment 62:387. Authors' contributions: The initials vary between 2 and 3 initials to represent authors' names, but the names in the authors list are all only two names, so there should be only 2 initials for each.
Response: We have corrected the initials of each name to 2. ' K.L., J.J. and P.X. designed and conceived the study. D.Y., C.Y. and J.Z. collected and prepared the samples. C.C. and C.Z. performed the data analysis. D.Y., C.C. and J.J. wrote the manuscript with significant contributions from Y.L., Z.C., H.Z., C.W. and L.L. K.L., Z.H. and D.L. provided the financial support. All authors read and approved the final version of the manuscript.'

Comment 63: Table 1. In the legend, provide the accession ID's for each genome version in the note below the table.
It's not clear why there is "C:" in front of each percentage for the two BUSCO lines. I think it would be clearer to remove "C:" from each number, put an asterisk (*) after BUSCO, and in the note, write "* percentage of complete BUSCO evaluation".
Response: Thank you for your valuable suggestions. We have provided the accession IDs for each genome except for the finless porpoise v2.0 genome assembly, as it is under control by NCBI but not yet public. Additionally, we have replaced "C:" with "BUSCO*" in the legend of Table 1, and mentioned "BUSCO*" as the percentage of complete BUSCO evaluation.

Figures.
Comment 64: Figure 1 contains too many unrelated figures, and too dense information, some of which is never discussed in the paper. I suggest that Figure 1 should include only A and F, B should be its own figure, and C,D,E should be moved to supplemental materials.
For the new figure (B), only the rings A (chromosome length) and F (syntenic blocks) should be included, as the others are too tiny and dense to be useful to the reader. Those can be put into supplemental materials with better descriptions to allow the reader to see the detail and to explain the colors and scale.
All colors within the figures need to be explained or include a color legend.
Response: Thank you very much for your suggestion. We have separated subfigures A and F from Figure 1B into individual new Figure 3. The remaining subfigures have been consolidated to create a new figure, now referred to as Supplemental Figure S7.

Figure 1. Sample site and Genome assessment of YFP v2.0 and EFP v2.0.
A: Location distribution and sampling site of the YFP and EFP.
B：Proportions of genes that could be functionally annotated and transcriptionally detected in YFP v2.0 and EFP v2.0.

Figure 3. Synteny analysis of YFP v2.0 and EFP v2.0 genomes: A) chromosomes scale, Unit length is Mb;

B) syntenic blocks between YFP v2.0 and EFP v2.0.

Comment 65: Figure 2. There is no visible "ruler" in the figure (but described in the legend).
The legends for A and B repeat most of the information. They can be combined to reduce repetition, e.g., Structure of T2T and gap-free chromosomes in A) Yangtze finless porpoises v2.0, and B) East Asian finless porpoises (v2.0). All 21+Xchromosomes are drawn to scale and the ruler indicates chromosome length. Triangles indicate the presence of telomere sequence repeats. Circles represent the locations of centromeric regions. The gap positions in the v1.0 genome assemblies are marked with squares to the right of the chromosome in the v2.0 genome assemblies.
Response: We have added "ruler" to the figure and combined the legends of A and B according to your helpful suggestion: 'Structure of T2T and gap-free chromosomes in A) YFP v2.0, and B) EFP v2.0. All 21+X/Y chromosomes are drawn to scale and the ruler indicates chromosome length. Triangles indicate the presence of telomere sequence repeats. Circles represent the locations of centromeric regions. The gap positions in the v1.0 genome assemblies are marked with squares to the right of the chromosome in the v2.0 genome assemblies'.

Comment 66: Figure 3. A and B are described as 'Heatmaps', but they appear to be box plots, not heatmaps. They are only described in one sentence in the text, so I suggest that they could be moved to supplemental materials, and include only C and D in the figure.
In C and D, the legend says "GO enrichment analysis of genes", but the figure is not showing analysis, but results. Please describe the plot and results, e.g., number of genes in centromere regions associated with GO pathways" (that may not be correct, but I'm not sure what the figure is showing, so it needs to be described better).
Figure 4 D and E could be added to Figure 3 to show all of the same types of plots in one figure instead of splitting data into different figures.
Response: Thank you for your suggestion. Inspired by your guidance, We have made the following adjustments to Figure 3：
A. Figures 3A and 3B have been relocated to the supplemental materials and re-titled as follows: Supplementary Figure S8 Box plots illustrating the gene expression levels in the centromere region and non-centromere region of YFP v2.0 and EFP v2.0. Corresponding gene expression data are presented in Supplementary Tables S16 and S17.
B. We sincerely apologize for the previous error in presenting the enrichment analysis in Figures 3C and 3D. In the revised manuscript, we have corrected this and now display the revised enrichment analysis results in the supplemental materials as: Supplementary Figure S9 KEGG enrichment of centromere genes of YFP v2.0 and EFP v2.0. Additionally, the KEGG enrichment analysis are presented in Supplementary Tables S19 and S20.
C. We extend our sincere apologies for the earlier error regarding the presentation of the enrichment analysis in Figure 4D and 4E. In the revised manuscript, we have made the necessary corrections by removing Figure 4D and 4E. The updated enrichment analysis results are now appropriately presented in the supplemental material, specifically in Supplementary Tables S26 to S29.

Comment 67: Figure 4.
A. what do the numbers next to the color legend represent? Is that the maximum number of SNPs per MB window? Is each color a range of values?
C. I don't understand what you mean by genes located in the Indel region or in the SNP region. What are the Indel and SNP 'regions'? These terms have not been used anywhere else or defined.
D-E. Consider moving to Figure 3. Are these 'new-found' genes relative to the old (V1) versions of the genomes, or genes that are unique to each species?
Response: I apologize for any inconvenience caused.
A. To improve the clarity of the presentation, we have made the following revisions to Figure 4 in the revised manuscript: the new Figure 4 now only retains sub-figures A and B; sub-figures C-E have been removed and replaced with the results of the KEGG enrichment analysis, which are now presented in Supplementary Figure S10 and Table S22.
B. The numerical values adjacent to the color legend indicate the count of SNPs/InDels per megabase (MB) window, where the gray legend representing zero. Each color corresponds to a specific range of values. For instance, in Figure 4A, the initial blue legend represents the range from 1 to 4440.
C. To address your inquiries regarding the terms "genes located in the Indel region" and "genes located in

the SNP region," allow me to provide clarification:
Following the detection of SNPs (single nucleotide polymorphisms) and InDels (insertions or deletions), we employed Annovar for functional annotation. This process evaluates how these genetic variations might impact gene structure. The phrases in question describe the spatial relationship between genes and the aforementioned genetic alterations:
Indel Region: An Indel refers to a segment of DNA that has been inserted or deleted within the genome. When we mention a gene located in an Indel region, it indicates that the gene is situated at the site of such an insertion or deletion event.
SNP Region: A SNP represents a variation involving a single nucleotide within the genome. If a gene is described as being in a SNP region, it suggests that the gene contains one or more SNPs.
In summary, these terms denote the proximity of genes to genetic variations that may have functional implications.
D. The 'new-found' genes mentioned in this manuscript are relative to the old (V1) versions of the genomes, and the associated gene functional enrichment results are presented in Supplementary Tables S26 to S29.
Ref:
Wang, K, Li, M and Hakonarson, H. ANNOVAR: functional annotation of genetic variants from high-throughput sequencing data. Nucleic Acids Res 2010;38(16):e164.

Comment 68: Figure 5.
A. "Ma: Million years ago" can be deleted, as "Ma" is not used in the figure.
B. what are the units for the scale? Is this a count of gene families?
C-D. define what "Rich Factor" means. What do the colors mean?
E. There was no description of E, and it is too small to read. I suggest moving it to supplemental materials to allow it to be described in detail and large enough for the reader to see if interested.
Response: We have retained A and B of Figure 5 and treated C, D, E as a separate Figure 6.
A."Ma" is used in the Figure 5A.
B. The units of the scale are "millions of years ago," which are used to represent the divergence times between species.
C-D. In the context of KEGG enrichment analysis, the "Rich Factor" is a measure that represents the ratio of the number of candidate genes annotated to a specific pathway to the total number of genes annotated to that pathway. It is an indicator of how significantly the differentially expressed genes are enriched within a particular KEGG pathway compared to the whole genome background. A higher Rich Factor implies a greater degree of enrichment, suggesting that the pathway is more relevant to the biological changes observed in the study.
The colors in a KEGG enrichment plot typically represent the significance of the enrichment. They are often associated with the q-value, which is a corrected p-value that adjusts for multiple hypothesis testing. The color intensity or shade—commonly ranging from blue to red—indicates the level of significance, with darker shades or redder colors signifying more significant enrichment (lower q-values). This visual representation helps researchers quickly identify pathways that are potentially important in the biological context of their study.
To summarize, the "Rich Factor" is a measure of enrichment significance, and the colors in a KEGG enrichment plot are a visual representation of the statistical significance of the enrichment, with redder colors indicating more significant enrichment.
E. To present the information more clearly, we have replaced Figure 5E with Figure 6C and 6D.

Figure 5. Genome evolution of YFP v2.0 and EFP v2.0.
A: Divergence time between YFP v2.0 and EFP v2.0, and number of expanded and contracted gene families. green and red numbers indicate gene family expansions and contractions, respectively. MRCA: Most Recent Common Ancestor. Ma: Million years ago.
B: A comparison of gene families associated with orthologs and paralogs in YFP v2.0 and EFP v2.0, and other 24 mammal species.

Figure 6. Functional Enrichment of genes.
Significant A) KEGG and B) GO enrichment of expanded gene families in YFP and EFP lineage.
KEGG enrichment analysis of positively selected genes in C) YFP and D) EFP, respectively.

Reviewer #2: In this work, Yin et al. present two newly assembled genomes of two narrow-ridged finless porpoise (Neophocaena asiaeorientalis) subspecies (Yangtze, YFP and East Asia, EFP) as well as a comprehensive report on its quality and differences in terms of genotype changes related to their different habitats. I overall enjoyed reading this manuscript, I did not find too many downfalls in their methods, and I think these two genomes are of the highest quality and one of if not the most continuous reference genomes for cetaceans published thus far. Apart of this, however, I found several downfalls of the manuscript text itself which leads me to conclude that a major revision is needed for this manuscript to be considered for publication in GigaScience. I will first talk about general issues, point out the few downfalls within their methods and then provide a more detailed list of text issues which may help the authors to revise the manuscript.
Response: Thank you very much for your comments on our manuscript. The comments are all valuable and very helpful for revising and improving our paper. Based on the comments we received, detailed modifications are provided below.

General issues:
Comment 1: The text of the manuscript has multiple grammatical errors and I invite the authors to revise the language of the manuscript critically and carefully. I will list the errors I found down below but will not guarantee that I found everything. Furthermore, many statements made by the authors come without a proper citation which I will also highlight down below.
Response: We value your correction of language and citation errors. In the revised version, we have double-checked the paper for grammar, spelling, formatting, and citation issues and corrected them.

Comment 2: The "Results and Discussion" part is missing a discussion. The authors invest the vast majority of this part describing their results and do not attempt to contextualize their results with recent literature. For example, the authors could have compared the quality of these reference genomes and annotations to other cetacean genomes to emphasize their superior quality. Furthermore, the authors did find, through various analyses, genes that might have played a role in their adaptions to different habitats but do not describe what is known about these genes and gene categories and do not describe how these gene might have help in their adaption. Also, the few sentences that do mention these possibilities come without proper citations, again.
Response: Thank you very much for your suggestion. We have added detailed description about genes with proper citations, which can be found in comment 23,29,30 and 31.

Comment 3: The material and method section is missing details. I assumed to find them in some sort of supplementary methods or data repository but failed to find them. If these details are indeed missing, I strongly suggest to provide more details in one of both ways (supplementary methods or within a data repository). I'll list the missing parts down below. Furthermore, the data repository made by the authors on figshare does lack a proper commentary README file and interested scientists are left to figure out the content of these files by themselves. I would strongly recommend providing an extensive README file for this repository to assure reproducibility.
Response: We have made revisions based on your suggestions provided. An enhanced version of the documentation file has been revised and is now accessible on the Figshare database. The documentation file contains the following contents:
For more details, please visit the Figshare website: https://figshare.com/s/1fc632fd4f3cab36b776.

Methods:
Comment 4: One part that is entirely missing in the Methods is the description of how SNPs were generated. Hence, I cannot evaluate the quality of their calls. I highly recommend adding the respective description because otherwise, these steps are not reproducible.
Response: Thank you for your insightful recommendations. Single nucleotide polymorphisms (SNPs) and insertions/deletions (InDels) were identified using methods similar to those previously described [1]. Genome alignment was conducted utilizing the NUCmer program integrated within MUMmer4 (v4.0.0) [2] to compare the v2.0 assembly with the v1.0 assembly, as well as the YFP assembly with the EFP assembly. Utilizing the Maximum Unique Matches (MUM) mode involved setting parameters such as a minimum MUM length of 1000 bp, a minimum similarity threshold of 90%, and the exclusion of matches below 40 bp. Alignment blocks were identified using the delta-filter program, while SNPs and InDels were detected using the show-snps program and Syri (v1.6.3) [3], respectively. Functional annotation of SNPs and InDels is

conducted using ANNOVAR [4] to assess their impacts on gene structure and function. Additionally, we utilize the CMplot R package [5] to visually depict the density distribution of these genetic variations. The objective of these analyses was to reveal structural variations between the v1.0 and v2.0 genome assemblies, as well as between the YFP and EFP genome assemblies. Variants were meticulously annotated utilizing the ANNOVAR package (v 2013-06-21) [4]. Correspondingly, we have amended the relevant descriptions in the revised MS at line 468-480.

Ref:

[1] Li, T, Xu, X, Zhao, J, et al. Genome assembly of KA105, a new resource for maize molecular breeding and genomic research. The Crop Journal 2023;11(6):1793-1804.

[2] Marçais, G, Delcher, A, Phillippy, A, et al. MUMmer4: A fast and versatile genome alignment system. PLoS Comput Biol 2018;14(1):e1005944.

[3] Goel, M, Sun, H, Jiao, W, et al. SyRI: finding genomic rearrangements and local sequence differences from whole-genome assemblies. Genome Biol 2019;20(1):277.

[4] Wang, K, Li, M and Hakonarson, H. ANNOVAR: functional annotation of genetic variants from high-throughput sequencing data. Nucleic Acids Res 2010;38(16):e164.

[5] Yin, L, Zhang, H, Tang, Z, et al. rMVP: A Memory-efficient, Visualization-enhanced, and Parallel-accelerated tool for Genome-Wide Association Study. Genomics, Proteomics & Bioinformatics 2021;19(4):619-28.

Comment 5: The authors use gene enrichment analyses multiple times but do not mention any false discovery rate correction. If this was not done, I highly recommend repeating these analyses with corrected p-values. If it was done, this information needs to be added to the manuscript. I further failed to find what was used as a reference gene set to compare the enrichment to. This is another information that needs to be provided to ensure reproducibility.

Response: We apologize for the oversight; the gene enrichment analysis mentioned in our previous Figures 3 and 4 was indeed incorrect. In the revised manuscript, we have corrected the gene enrichment analysis and have applied the q value to adjust the p values. The updated results are now presented in Figure 6, Supplementary Tables 19-20, 22, 26-29. Specifically, the reference gene set used for enrichment comparison in Figure 6 C, Tables S19, 22, 26, 27 is from YFP v2.0; the reference gene set used for enrichment comparison in Figure 6D, Tables 20, 21, 28, 29 is from EFP v2.0; and the reference gene sets used for enrichment comparison in Figure 6A and B are both from YFP v2.0 and EFP v2.0.

List:

In the following, I'll list the more specific issues I found within the manuscript which may help the authors to revise their manuscript:

Introduction:

Comment 6: Line 43-46: This sentence implies the results of a study, but no citation is provided.

Response: We are sorry for the mistake. We have added the following citations to the sentence: "Based on morphological characteristics, geographic distribution and molecular genetic evidence, it is generally believed that the finless porpoise can be divided into two species, namely the Indo-Pacific finless porpoise (N. phocaenoides) and the narrow-ridged finless porpoise (N. asiaeorientalis) [1-2]."

Ref

[1] Wang, J, Frasier, T, Yang, S, et al. Detecting recent speciation events: the case of the finless porpoise (genus Neophocaena). Heredity 2008;101(2):145-55.

[2] Jefferson, T and Wang, J. Revision of the taxonomy of finless porpoises (genus Neophocaena): The existence of two species. J Mar Anim Ecol 2011;4(1):3-16.

Comment 7: Line 53: I think the "urgent priority for scientists" needs to be more justified. Consider toning this down or being more specific.

Response: Thank you very much for your suggestion. We have changed "urgent priority for scientists" to "a pressing concern for scientists".

Comment 8: Line 57-58: This sentence implies the results of a study, but no citation is provided.

Response: We are sorry for the mistake. We have added the following citation to the sentence: "The genetic diversity of East Asian finless porpoises surpasses that of the other two populations, indicating it as the likely center of origin for this species [1]."

Ref

[1] Yang, G, Ren, W, Zhou, K, et al. Population genetic structure of finless porpoises, Neophocaena phocaenoides, in Chinese waters, inferred from mitochondrial control region sequences. Marine mammal science 2002;18(2):336-47.

Comment 9: Line 67: "Availability of a high-quality genome assembly."
Response: We are sorry for the mistake. We have changed "Availability of high-quality genome assembly…" to "Availability of a high-quality genome assembly…"

Comment 10: Line 79-81: This is not a sentence.
Response: We are sorry for the mistake. We have changed the sentence to "The T2T genome has emerged as a hotspot genomic research, demonstrating extensive applications to other animal species like chicken and fish."

Comment 11: Line 84: I would suggest to either make the word "genome" plural or add an "a" at the beginning of the sentence.
Response: We are sorry for the mistake. We have added an "a" at the beginning of the sentence.

Comment 12: Line 86-91: Consider changing these sentences to present.
Response: Thank you very much for your suggestion. We have changed these sentences to present.

Comment 13: Line 92-93: This statement is extremely vague. Consider being more specific about what we can learn from their genomes.
Response: Thank you very much for your suggestion. We have changed this statement to "The high-quality chromosomal-level references help elucidate ecological and aquatic adaptation mechanisms in cetaceans."

Results and Discussion:
Comment 14: Line 99: "Based on" doesn't make sense when speaking about additional sequencing efforts.
Response: Thank you very much for your suggestion. We have changed "Based on the previously sequenced 62x PacBio HiFi and 85x Hi-C reads of the EFP, we generated 215 Gb (86x) ONT reads in this study" to "In this study, we supplemented the existing dataset comprising 62x PacBio HiFi and 85x Hi-C reads of the EFP with an additional 215 Gb (86x) of ONT reads."

Comment 15: Line 104-105: How can scaffold and contig statistics be completely identical?
Response: Thank you for drawing our attention to this. The genomic assembly in this study initially progressed at the contig level by assembling PacBio data using the hifiasm software. Subsequently, redundant contigs were removed through alignment of the assembly with the PacBio data using the purge_haplotigs software, which utilized alignment coverage depth and other relevant metrics. Furthermore, the improved assembly was compared to the complete genome of Neophocaena asiaeorientalis mitochondrion (accession number: NC_026456.1) using the minimap2 software to detect possible mitochondrial sequences. The contig with the highest alignment score (temporarily designated as: ptg000094l) was selected as the mitochondrial genome sequence for the species under study, while other potential sequences were excluded. Next, the ultimate contig-level genomic assembly was precisely anchored to the chromosome level utilizing Hi-C data. Finally, gap filling was performed using Nanopore and PacBio data to achieve a chromosome-level assembly that is free of gaps. Upon completion, the scaffold and contig statistics produced identical results.

Comment 16: Line 116-118: In this sentence is a verb missing. Also, consider explaining the mercury score in one sentence since this is something not known by the generally informed reader.
Response: We are sorry for the mistake. We have changed "The Merqury estimated quality values of 60.18 and 64.38 based on k-mer analysis of YFP and EFP, respectively, which indicated that our assemblies were of high quality" to "The quality values obtained from Merqury's k-mer analysis for YFP and EFP were calculated as 60.18 and 64.38, respectively. These values indicated the superior quality of our assemblies. It is essential to emphasize that Merqury quality values span from 0 to 255, with higher values denoting superior quality."

Comment 17: Line 137: You may want to replace "constituting" with "reaching" or "accounting for"

Response: We have replaced "constituting" with "accounting for".

Comment 18: Line 139-143: You have an excessive use of the word "respectively" in this paragraph.
Response: Thank you very much for your suggestion. We have changed " In total, we predicted 23,139 and 23,101 protein-coding genes in the YFP and EFP genomes, respectively…" to "In total, the number of predicted protein-coding genes was 23,139 in the YFP genome and 23,101 in the EFP genome…"
In addition, we have revised the text with simple summary values at lines 163-172 in the revised MS to minimize redundancy and emphasize the main discoveries. The revised section now offers a succinct overview of the predicted protein-coding genes in the YFP and EFP genomes, supported by evidence and a comparison of length distributions. Supplementary Tables 9-12 contain further information, such as the average lengths of coding sequences, exons, and introns, as recommended.

Comment 19: Line 141-142: Consider maxing the word "exon" and "intron" plural.
Response: Thank you very much for your suggestion. We have removed the average lengths of coding sequences, exons, and introns to supplementary Tables.

Comment 20: Line 145: Consider turning the "was" into present.
Response: We have changed the word "was" to "is".

Comment 21: Line 159: Consider rephrasing "mystery" to something more objective.
Response: Thank you very much for your suggestion. We have changed "The mystery of the evolution of centromere structure…" to "The intrinsic mechanisms underlying the evolution of centromere structure…"

Comment 22: Line 163-165: It is not directly clear what the difference between "candidate centromere region" and "newly identified centromere regions" is. Consider adding a sentence to explain these differences.
Response: We are sorry for the unclear description. The term "candidate centromere region" refers to regions identified by centromere-finding software quartet pipeline as potential centromeres. "Newly identified centromere regions" indicate areas predicted by the software, which are present in the freshly assembled assembly of this study but absent in the previously published v1.0 version of the Neophocaena asiaeorientalis genome.
The method for obtaining these newly assembled regions in this study can be found in the "Genome comparison and Identification of newly assembled genes” section. We have changed the sentence to "A total of 235 and 237 genes were identified in the candidate centromere regions for YFP and EFP, respectively, through predictions generated by centromere-finding software. Moreover, 56 genes were identified in the newly discovered centromere regions for YFP, while 20 genes were found in analogous regions for EFP. The "newly identified centromere regions" refer to specific areas identified in the genome that have been recently assembled but were not included in the previously published version. This discovery may suggest the presence of previously unknown centromere sites."

Comment 23: Line 188: It is not clear what the authors meant with "microbial category". Please add further explanation to this.
Response: We are sorry for the mistake. We have changed "microbial category" to "microbial diversity", and expanded upon this paragraph. Please refer to the following question (Comment 24) for further details.

Comment 24: Line: 188-193: These sentences imply the results of a study, but a citation is missing. The paragraph is also very vague. Is there something known about different microbial stressors in sea- and freshwater?
Response: We are sorry for the unclear description. We have revised and expanded upon this paragraph: "The genes coding for the mutated regions of the YFP and EFP are widely enriched in immune-related pathways. This association may be intricately linked to the distinct pathogenic microorganisms unique to freshwater and seawater environments. Marine mammals exhibit a diminished histocompatibility complex (MHC) diversity attributed to decreased encounters with microparasitic diversity in their marine habitat relative to their terrestrial origin. This phenomenon implies that mammals encounter distinct pathogenic pressures in varied ecological settings, potentially influencing the evolution of immune-related genes. [1]. Evolutionary analyses of the innate immune pattern recognition receptor (TLRs) in the YFP and the marine finless porpoise indicate that the YFP has undergone specific adaptive changes [2]. The microbial diversity

and pathogenicity of freshwater and seawater environments vary, leading to distinct effects of pathogenic microorganisms on the organisms in these two types of environments [3]. Therefore, the YFP and EFP would be expected to undergo adaptive evolution to adapt to the pathogen stresses specific to their respective ecological environments, freshwater and seawater."
Ref
[1] Slade, R and McCallum, H. Overdominant vs. frequency-dependent selection at MHC loci. Genetics 1992;132(3):861–64.
[2] Tian, R, Chen, M, Chai, S, et al. Divergent Selection of Pattern Recognition Receptors in Mammals with Different Ecological Characteristics. J Mol Evol 2018;86(2):138-49.
[3] Lokesh, J and Kiron, V. Transition from freshwater to seawater reshapes the skin-associated microbiota of Atlantic salmon. Sci Rep 2016;25(6):19707.

Comment 25: Line 195: I don't think it is correct to say that a subspecies "assembled" new genes.
Response: We are sorry for the mistake. We have changed "… the YFP assembled 5480 new genes, while EFP assembled 1453 new genes..." to "…the YFP assembly included 5480 new genes, while the EFP assembly included 1453 new genes..."

Comment 26: Line 203: Consider changing "including" to: "which include 594 species-specific genes"
Response: We are sorry for the mistake. We have changed "including" to "which include 594 species-specific genes".

Comment 27: Line 205-206: You jump from the alignment of genes to the phylogenetic dating without mentioning the phylogenetic tree itself. I would add a sentence between these two to briefly describe the tree you made before talking about the specific dates you received for the emergence of the two subspecies.
Response: Thank you for your insightful recommendations. We have modified line 273-279 of the article with the following sentences: "Among the dataset, a total of 2,161 single-copy gene families were identified. Subsequently, multiple sequence alignments were conducted, which were then followed by the reconstruction of evolutionary trees. The resulting phylogenetic tree exhibited a consistent topology with previous studies, highlighting its reliability and alignment with established scientific knowledge. This analysis notably revealed the topological arrangement within branches of mammals. The emergence of the EFP branch alongside its YFP counterpart was particularly noteworthy."

Comment 28: Line 209: I do not think it is correct to call one of both subspecies more conserved than the other. Figure 1B is a synteny comparison between both genomes and without an outgroup it is not possible to infer the ancestral state of both possible rearrangements. If a comparison between other more distantly related genomes happened, I would mention them here as well, otherwise, I would remove this statement and directly talk about the possible rearrangements.
Response: Thank you for your suggestion; our previous description was indeed lacking in rigor. Following your advice, we have discussed the genomic rearrangements of the two subspecies in line 223-225 of the revised manuscript.

Comment 29: Line 212-214: My first suspicion reading this sentence was that the chromosomes simply weren't fully assembled in the EFP genome. But comparing this sentence with Figure 1B, I think the authors intended to say that parts of YFP chromosomes matched parts of multiple chromosomes within the EFP genome. If so, please consider rephrasing this sentence to avoid this confusion.
Response: Thank you very much for your suggestion. We have changed the sentence to " Synteny analysis of the gene order between YFP v2.0 and EFP v2.0 revealed 907 large shared syntenic blocks, encompassing 89.59% (41,428) genes, and 17 chromosomal rearrangements (Figure 3)."

Figure 3. Synteny analysis of YFP v2.0 and EFP v2.0 genomes: A) chromosomes scale, Unit length is Mb; B) syntenic blocks between YFP v2.0 and EFP v2.0.

Comment 30: Line 229-230: Here is a point where the authors should definitely try to contextualize their findings with recent literature. Are these functions that are convergently evolved by other species adapting to freshwater?

Response: Thank you very much for your suggestion. We have revised and expanded upon this paragraph: 'Gene families associated with "oxidative phosphorylation", "cytochrome-c oxidase activity", "riboflavin biosynthetic process", "ferric iron binding" and "iron ion transport" exhibited expansion in finless porpoises. Iron ions play a crucial role in numerous essential physiological functions within living organisms, such as oxygen transportation, electron transport chains, and various metabolic pathways. Several sets of redox reactions are necessary to maintain effective gas exchange in water for cetaceans, and iron is often used in these reactions as an electron acceptor [1]. The expansion of redox reaction and iron ion gene families in cetaceans potentially improved the efficacy of oxygen utilization and facilitated adaptation to hypoxic conditions in aquatic habitats. An increase in the number of oxidation-reduction and iron-binding gene families was also observed in L.vexillifer, a species that encounters hypoxic conditions during dives [2]. The changes in pathogenic microorganisms that occur during the reintroduction of cetaceans from land to sea present a significant challenge to their survival and may have influenced the evolution and adaptation of immune genes [3]. The expansion of immune-related gene families in finless porpoises in this study enhanced the process of antigen presentation and conferred resistance against various pathogenic microorganisms amidst changes in their habitat. Genomic studies of the S. chinensis have revealed that cetaceans have developed various species-specific gene families related to immunity and DNA repair, which are linked to potential adaptive mechanisms [4]. We suggest these genes play a crucial role in facilitating hypoxic tolerance and enhancing immune resistance in finless porpoises, thereby reflecting potential mechanisms of adaptation to the aquatic environment. Further investigations are required to elucidate the specific functions of these gene families and their potential significance in the biology of finless porpoises.'

Ref:
[1] Guo, B, Sun, Y, Wang, Y, et al. Evolutionary genetics of pulmonary anatomical adaptations in deep-diving cetaceans. BMC Genomics 2024;25(1):339.
[2] Zhou, X, Sun, F, Xu, S, et al. Baiji genomes reveal low genetic variability and new insights into secondary aquatic adaptations. Nat Commun 2013;4:2708.
[3] Li, L, Rong, X, Li, G, et al. Genomic organization and adaptive evolution of IGHC genes in marine mammals. Mol Immunol 2018;99:75-81.
[4] Ming, Y, Jian, J, Yu, F, et al. Molecular footprints of inshore aquatic adaptation in Indo-Pacific humpback dolphin (Sousa chinensis). Genomics 2019;111(5):1034-42.

Comment 31: Line 239: The authors definitely need to elaborate what might specifically cause these genomic instabilities. Is their something known about specific components and how they function as mutagens?

Response: We are sorry for the mistake. We have revised and expanded upon this paragraph: 'The selective pressure to evolve DNA damage repair pathways implied that the Yangtze finless porpoise might be experiencing increased threats to genome stability. The mechanism of DNA damage repair plays a crucial role in preserving genome integrity by enabling cells to identify and repair DNA damage, thereby averting the accumulation of harmful mutations [1]. In a comparative genomic analysis between the South China tiger and the Amur tiger, it was noted that genes related to DNA repair underwent positive selection in the South China tiger [2]. The observed phenomenon could be explained by the higher probability of genome instability in the temperate and subtropical habitats of the South China tiger. This may be linked to metabolites generated by intestinal microflora, which possess the ability to trigger DNA damage [3]. The stability of the genome or gene expression system in the Yangtze finless porpoise across different organs and life stages remains uncertain. However, one potential interpretation of this data is the suggestion that the Yangtze finless porpoise could be vulnerable to genomic instability triggers in the Yangtze River, such as water pollutants, which may increase the likelihood of DNA damage [4]. Pollutants found in the Yangtze River possess the capacity to accumulate within the food chain, leading to cellular DNA damage and impacting the genome stability of the Yangtze finless porpoise [5-6]. This makes a compelling case for the improved conservation of the species and the development of more rigorous water pollution mitigation practices.'

Ref
[1] Chatterjee, N and Walker, G. Mechanisms of DNA damage, repair, and mutagenesis. Environ Mol Mutagen 2017;58(5):235-63.
[2] Zhang, L, Lan, T, Lin, C, et al. Chromosome-scale genomes reveal genomic consequences of inbreeding in the South China tiger: A comparative study with the Amur tiger. Mol Ecol Resour 2023;23(2):330-47.
[3] Puschhof, J and Sears, C. Microbial metabolites damage DNA. Science 2022;378(6618):358-59.
[4] Lv, W, Gu, H, He, D, et al. Polystyrene nanospheres-induced hepatotoxicity in swamp eel (Monopterus

albus): From biochemical, pathological and transcriptomic perspectives. Sci Total Environ 2023;893:164844.
[5] Zhang, K, Qian, Z, Ruan, Y, et al. First evaluation of legacy persistent organic pollutant contamination status of stranded Yangtze finless porpoises along the Yangtze River Basin, China. Sci Total Environ 2020;710:136446.
[6] Xiong, X, Qian, Z, Mei, Z, et al. Trace elements accumulation in the Yangtze finless porpoise (Neophocaena asiaeorientalis asiaeorientalis)-A threat to the endangered freshwater cetacean. Sci Total Environ 2019;686:797-804.

Comment 32: Line 240: Why are these PSGs "interesting"? Again, no context is given.
Response: Thank you very much for your suggestion. We have revised and expanded upon this paragraph: Interestingly, a total of 44 PSGs within the EFP lineage were involved in “sodium-dependent phosphate transport”, “sodium symporter activity”, “aldosterone-regulated sodium reabsorption”, and “calcium signaling pathway”. These metabolic pathways play an important role in regulating sodium levels in the body [1]. Among these PSGs, six were potentially associated with the adaptation of EFP to high osmolarity environment, including the Na(+)/H(+) exchange regulatory cofactor NHE-RF2 and sodium-dependent phosphate cotransporter SLC34. These genes identified are likely to have significant implications in the control of urine formation and the preservation of water-salt metabolic balance [1], suggesting that the East Asian finless porpoise may have a different urine formation process. Previous genome-selective sweep analyses in the finless porpoises revealed that the SLC14A in the East Asian finless porpoise underwent positive selection [2]. Comparative analyses conducted at the transcriptome level between the Yangtze finless porpoise and the East Asian finless porpoise revealed a notable upregulation of the NHE3 in the kidneys of the East Asian finless porpoise. These results imply some adaptive enhanced osmoregulatory capability in the East Asian finless porpoise [3]. The aforementioned studies suggest that the East Asian finless porpoise has evolved a complex and efficient osmoregulatory mechanism as it acclimatized to the hypertonic marine environment, demonstrating adaptations at both molecular and transcriptional levels.
Ref:
[1] Shoemaker, V and Nagy, K. Osmoregulation in amphibians and reptiles. Annu Rev Physiol. 1977;39: 449-71.
[2] Zhou, X, Guang, X, Sun, D, et al. Population genomics of finless porpoises reveal an incipient cetacean species adapted to freshwater. Nat Commun 2018;9(1):1276.
[3] Ruan, R, Guo, A, Hao, Y, et al. De novo assembly and characterization of narrow-ridged finless porpoise renal transcriptome and identification of candidate genes involved in osmoregulation. Int J Mol Sci 2015;16(1):2220-38.

Methods:
Comment 33: Line 252: I would invite the authors to provide more details of why no ethical considerations were taken into account. I'm also not sure if this is in conflict with GigaScience's own ethical guidelines. But I would leave this decision to the Editor and the Journal.
Response: The specimens of the YFP and EFP utilized for genome sequencing were obtained from dead individuals found in their natural habitat. The Office of Fishery Supervision and Management in the Yangtze River Basin, Ministry of Agriculture and Rural Affairs of the People's Republic of China has designated our research institution to perform post-mortem analysis and genetic preservation on deceased porpoises.

Comment 34: Line 259: I cannot find which library kit was used for the Hi-C library.
Response: Thanks for the suggestion. We have modified and enriched the methods: 'The PacBio HiFi library was constructed using SMRTbell Prep Kit 3.0 (Pacific Biosciences, USA) and subsequently sequenced on the PacBio Sequel II system in circular consensus sequence (CCS) mode. To collect data for the Hi-C library, the muscle tissues were first fixed in 1% formaldehyde (Sigma) for cross-linking and resuspended in lysis buffer. Then, MboI (NEB) restriction endonucleases were used to fragment the chromatin in the muscle to fragment DNA. The DNA fragments were captured by utilizing Streptavidin-coated magnetic beads (Thermo Fisher SCIENTIFIC) following biotin labeling and crosslinking using T4 DNA Ligase (ENZYMATICS). The Hi-C library was finally sequenced on a BGI MGISEQ platform.'

Comment 35: Line 268: The code for the self-designed Perl program is not provided in any repository and it is not described how the ONT reads were refined in this step.
Response: Thank you for your valuable suggestion. We have revised the text in Line 409: "ONT reads were

filtered based on a length < 5 kb and a quality value < 7."

Comment 36: Line 272-274: This is not a sentence. A verb is missing.
Response: We are sorry for the mistake. We have changed "Juicer (v1.5) and 3D-DNA (v180922) pipeline for clustering, ordering, and orienting the contigs into pseudo-chromosomes using Hi-C data." to "We utilized the Hi-C data to cluster, order, and orient the contigs into pseudo-chromosomes through the implementation of the Juicer (v1.5) and 3D-DNA (v180922) pipeline."

Comment 37: Line 281: The authors chose the OrthoDB for mammals (not stated here but in the results) and I wonder why they did not use the Cetacea specific one. Just a minor comment.
Response: Thank you for your valuable comment. Given that the majority of research on cetaceans has relied on the OrthoDB for mammals for assessments, we chose to ensure consistency in data comparison by employing the same OrthoDB for mammals database in our analysis. For example, Gao, H., et al. (2023) employed the OrthoDB database specifically designed for mammals in their research.
Ref:
Gao, H, Kang, H, Zhang, Y, et al. Chromosome-Level Genome Assembly of the Rough-Toothed Dolphin (Steno bredanensis). Journal of Marine Science and Engineering 2023;11(2): 418.

Comment 38: Line 286-291: The homology-based repeat annotation was not based on any databases like RepBase? What homologous information was used? Please specify.
Response: Thank you for your reminder. Our previous description was incomplete, as we failed to mention the utilization of the Repbase database in the Repeat analysis process. Consequently, the description in line 441-444 has been revised as follows:
"For homolog-based prediction, RepeatMasker (v4.0.7) was utilized to detect DNA transposable elements (TEs), while RepeatProteinMasker (v4.0.7) was employed to identify protein-based TEs, both based on the Repbase database."

Comment 39: Line 301: How is "high quality genes" defined? No information is given.
Response: Thank you for your reminder. "high-quality genes" refers to those genes predicted by the GeMoMa software and validated by OrthoDB for mammals. We have made modifications in the manuscript at line 461-463: "A set of one thousand high-quality genes, which were predicted by the GeMoMa software and validated by OrthoDB for mammals, was randomly selected for training the predictors in Augustus v3.2.1."

Comment 40: Line 305-315: The paragraph "Genome comparison" and "Identification of new assembled genes" is extremely similarly phrased. Would it be possible to merge them into one, shorter paragraph to avoid redundancy?
Response: Thank you very much for your suggestion. We have merged "Genome comparison" and "Identification of new assembled genes" into one paragraph: "Genome comparison and Identification of newly assembled genes

Comment 41: Line 320: Was there no model testing done? Please specify. Also, there is no information given of how many bootstrap replications were conducted. I guess 1000 but this information is not given.
Response: Thank you for your reminder. In this study, model testing was omitted, and the default HKY85 model was used for the phylogenetic analysis. A phylogenetic tree was constructed using the maximum-likelihood method in PhyML, and the reliability of the results was assessed through 1000 bootstrap replications. The phylogenetic relationship among the three lineages of marine mammals is consistent with previous research findings.
Consequently, the description in line 482-488 has been revised as follows: "Single-copy orthologous genes (1:1:1) were aligned using MAFFT (v7.310), a widely utilized tool for multiple sequence alignment. Subsequently, a maximum-likelihood phylogenetic tree was generated using PhyML (v3.3), a commonly employed software tool for phylogenetic analysis. The HKY85 model, known for its capacity to accommodate nucleotide substitution patterns, was applied in the tree construction procedure. To evaluate the reliability of the generated tree, 1000 bootstrap replications were performed, providing statistical evidence for the branching structures."
Ref:
Yuan Y, Zhang Y, Zhang P, et al. Comparative genomics provides insights into the aquatic adaptations of

mammals[J]. Proceedings of the National Academy of Sciences, 2021, 118(37): e2106080118.
M. R. McGowen, J. Gatesy, D. E. Wildman, Molecular evolution tracks macroevolutionary transitions in Cetacea. Trends Ecol. Evol. 29, 336–346 (2014).
Z. Chen, S. Xu, K. Zhou, G. Yang, Whale phylogeny and rapid radiation events revealed using novel retroposed elements and their flanking sequences. BMC Evol. Biol. 11, 314 (2011).
M. R. McGowen, Toward the resolution of an explosive radiation—Amultilocusphylogeny of oceanic dolphins (Delphinidae). Mol. Phylogenet. Evol. 60,345–357 (2011). 17. M. R. McGowen et al., Phylogenomic resolution of the cetacean tree of life using target sequence capture. Syst. Biol. 69, 479–501 (2020).

Comment 42: Line 337-338: Gene enrichment analysis usually requires some sort of correction for false discovery rates. However, nothing like this was described in the manuscript. If this was done, I would mention it here. Otherwise, I would definitely recommend correcting for this!
Response: We apologize for the oversight; the gene enrichment analysis mentioned in our previous Figures 3 and 4 was indeed incorrect. In the revised manuscript, we have corrected the gene enrichment analysis and have applied the q value to adjust the p values. The updated results are now presented in Figure 6, Supplementary Tables 19-20, 22, 26-29.

Comment 43: Line 339: I guess the word "selection" is missing in this paragraph headline.
Response: We are sorry for the mistake. We have changed the section title to 'Gene positive selection analysis'.
