# Supplementary material for: Telomere-to-telomere gap-free genome assembly of the endangered Yangtze finless porpoise and East Asian finless porpoise
Source: Gigascience. 2024 Sep 16;13:giae067. doi: 10.1093/gigascience/giae067 (PMC11403816; doi:10.1093/gigascience/giae067)
Supplement: giae067_Response_to_Reviewer_Comments_Revision_1 [file giae067_response_to_reviewer_comments_revision_1.pdf]

Author's Response To Reviewer Comments

Dear Dr. Hans Zauner,
Thanks for your kind consideration and comments regarding to our manuscript entitled "Telomere-to-telomere gap-free genome assembly of the endangered Yangtze finless porpoise and East Asian finless porpoise" (GIGA-D-23-00359R1). We have revised our manuscript (details are provided below) in accordance with all comments and suggestions from the reviewer 2. We hope the revised manuscript have been improved and meet the standard requirement. We look forward to hearing from GigaScience soon.

Sincerely yours,

Correspondence: Kai Liu
Freshwater Fisheries Research Center, Chinese Academy of Fishery Sciences, Wuxi 214081, China.
E-mail: liuk@ffrc.cn

Response to reviewer 2's comments:
Reviewer #2: In this manuscript, Denghua Yin and colleagues present two new, high-quality, de novo genome assemblies of two narrow-ridged finless porpoise (Neophocaena asiaeorientalis) subspecies (Yangtze, YFP and East Asia, EFP) as well as a comprehensive report on its quality and analyses that identify molecular differences related to their different habitats (fresh and sea water). This is the second time I'm reading this manuscript and I think the manuscript was substantially improved by the changes made in response to the last review. I still regard these genomes to be of the highest quality and a valuable resource for future studies. The methodological shortcomings which I highlighted last time were completely addressed and the language problems were amended in most cases. The only general issue that wasn't covered enough since last time is in the discussion where the authors fail to provide more comparisons to assemblies from the literature. The authors mention in the conclusion that these assemblies are potentially the "most continuous" genomes of cetacean today and I tend to believe them. However, the manuscript is lacking a clear comparison to other assemblies to make this statement. I already suggested this in my last review, and I consider this to be crucial, although I'm aware that this conflicts to some extent with the suggestions made by reviewer 1. Hence, I suggest to the authors to add just a brief comparison (2-3 sentences) to the discussion to validate this claim, to provide some contextual statistics and to give the reader an idea about the improved quality of these genomes. In the following, I'll list some additional suggestions that may improve the manuscript. Given that these suggestions require only minor changes to the text, I recommend a minor revision to the editor.
Response: Thank you very much for your comments on our manuscript. The comments provided are all valuable and very helpful for revising and improving our paper. We have addressed the only general issue raised in comment 1. Based on the comments we received, detailed modifications are provided below.

General issues:
Comment 1: As mentioned already, I suggest adding a small comparative paragraph to the discussion to compare the quality of these genomes to previous genomes of Cetacea.
Response: According to the reviewer's suggestion, we have compared the genome assemblies presented in this study with 34 cetacean genomes available from public repositories such as NCBI and CNGB. The comprehensive analysis confirms that our assemblies are the most continuous among cetaceans known to date. Notably, our contig N50 and scaffold N50 values significantly surpass those of other genomes, while the number of contigs is considerably lower, highlighting the improved continuity and quality of our assemblies. In addition, a table summarizing the statistical comparison of the genomes has been included. This table is now cited in the main text and is presented as Supplementary Table S3. This table provides a concise overview of the data that underpins our assertions and enables a quick assessment of the comparative genomic consistency among cetacean species.
We have added a comparative paragraph at lines 120-129 in the revised MS: "As of May 2024, the killer whale genome assembly (GCF_937001465.1) stands out among the 34 publicly available cetacean genomes sourced from the NCBI (National Center for Biotechnology Information) and CNGBdb (China National GeneBank DataBase) databases. It features an N50 length of 45.6 Mb and is composed of 570

contigs. In our study, the two newly assembled genomes exhibit a marked improvement in contiguity. The contig N50 length achieved in our assemblies is threefold that of the killer whale genome, with the number of contigs comprising only 4.03% of the latter. Moreover, the analysis of the blue whale genome (GCF_009873245.2) shows a contig N50 value that is only 4.0% of our assemblies, while its contig count is 42 times higher [1] (Supplementary Table S3). The comparative metrics highlight the superior continuity of the YFP and EFP genome assemblies in this study."

Ref:
[1] Bukhman, Y, Morin, P, Meyer, S, et al. A high-quality blue whale genome, segmental duplications, and historical demography. Mol Biol Evol 2024; 41(3): msae036.

Supplementary Table S3 Assembly statistics for 36 species of cetaceans.

Species Common name Genome Size/Gb Contig N50/Mb Contig Numbers Super-scaffold N50/Mb Scaffold Number Data Source

Neophocaena asiaeorientalis sunameri East Asian finless porpoise (EFP v2.0) 2.50 128.00 23 128.00 23 This study (PRJNA859258)

Neophocaena asiaeorientalis asiaeorientalis Yangtze finless porpoise (YEP v2.0) 2.48 125.12 22 125.12 22 This study (PRJNA915046)

Neophocaena asiaeorientalis asiaeorientalis East Asian finless porpoise (EFP v1.0) 2.50 84.69 52 122.40 23 GCA_026225855.1

Orcinus orca Killer whale 2.6 45.6 570 114.20 447 GCF_937001465.1

Kogia breviceps Pygmy sperm whale 2.60 42.80 723 122.40 580 GCF_026419965.1

Inia geoffrensis Boutu 2.70 40.90 1,186 117.40 902 GCA_036417435.1

Eschrichtius robustus Grey whale 3.00 39.2 982 126.50 703 GCA_028021215.1

Eubalaena glacialis North Atlantic right whale 2.80 37.50 1,155 129.60 788 GCF_028564815.1

Balaenoptera ricei

Rice's whale 2.70 36.50 1,108 122.10 774 GCF_028023285.1

Phocoena sinus vaquita 2.40 20.20 272 115.50 64 GCF_008692025.1

Tursiops truncatus Bottlenosed dolphins 2.37 9.73 1,035 108.43 361 GCF_011762595.1

Balaenoptera musculus Blue whale 2.40 6.30 972 110.30 105 GCF_009873245.2

Phocoena phocoena Harbor porpoise 2.50 3.7 1,405 110.2 439 GCA_963924725.1

Delphinus delphis saddleback dolphin 2.70 3.60 1,598 107.1 630 GCF_949987515.1

Stenella coeruleoalba Striped dolphin 2.70 3.60 1,630 105.30 637 GCA_951394435.1

Lagenorhynchus albirostris White-beaked dolphin 2.5 3.40 1,479 110.7 409 GCF_949774975.1

Globicephala melas Long-finned pilot whale 2.70 3.30 2,076 105.10 992 GCF_963455315.1

Hyperoodon ampullatus Northern bottlenose whale 2.80 3.10 1,854 117.4 756 GCA_949752795.1

Balaenoptera acutorostrata Minke whale 2.80 3.00 2,551 116.50 1,374 GCF_949987535.1

Lagenorhynchus obliquidens Pacific white-sided dolphin 2.46 0.28 29,411 30.04 13,174 CNA0007259

Delphinapterus leucas Beluga whale 2.32 0.20 29,443 31.18 5,904 GCF_002288925.2

Sousa chinensis Indo-Pacific humpback dolphin 2.34 0.18 46,900 9.01 20,903 GCA_003521335.2

Pseudorca crassidens False killer whale 2.43 0.15 60,880 32.50 32,690 CNA0007265

Tursiops aduncus Indo-Pacific bottlenose dolphin 2.41 0.11 160,669 104.71 118,124 CNA0007267

Neophocaena asiaeorientalis asiaeorientalis Yangtze finless porpoise (YEP v1.0) 2.27 0.09 66,342 6.34 13,695 GCF_003031525.2

Balaenoptera musculus Blue whale 2.37 0.08 79,155 8.28 28,500 CNA0007254

Balaenoptera bryde Bryde’s whale 2.37 0.07 140,815 103.91 74,190 CNA0007253

Grampus griseus Risso's dolphin 2.50 0.06 813,233 93.20 773,381 GCA_028646425.1

Kogia sima Dwarf sperm whale 2.59 0.06 503,547 26.52 428,024 CNA0007257

Steno bredanensis Rough-toothed dolphin 2.30 0.06 266,806 94.90 221,678 GCA_028646385.1

Lagenorhynchus australis Peale’s dolphin 2.39 0.05 225,716 0.38 151,990 CNA0007258

Balaena mysticetus Bowhead whale 2.31 0.04 118,156 0.88 7,227 bowhead-whale.org

Physeter catodon Sperm whale 2.51 0.04 133,554 122.18 12,168 GCF_002837175.3

Lipotes vexillifer Yangtze River dolphin 2.37 0.03 148,597 2.44 24,809 GCF_000442215.2

Mesoplodon densirostris Blainville’s beaked whale 2.47 0.03 1,071,210 0.80 944,262 CNA0007260

Neophocaena phocaenoides Indo-Pacific finless porpoise 2.62 0.01 2,048,710 0.43 1,806,060 CNA0007261

Abstract:
Comment 2: Line 31: I do not think you can "study comparative genomes". Consider "study structural similarities" instead.
Response: Thank you very much for your suggestion. We have changed "comparative genomes" to "structural similarities".

Introduction:
Comment 3: Line 95-98: Same as in line 30. Furthermore, the article "the" is used in a confusing way here. Consider changing the sentence to: "We compare the quality of the newly drafted assemblies with previously available versions and explore synteny blocks, structural similarities, phylogenetic relationships, gene family expansion and inference of selection in relation to several other mammals."
Response: We are sorry for the mistake. We have changed the sentence to: "We compare the quality of the newly drafted assemblies with previously available versions and explore synteny blocks, structural similarities, phylogenetic relationships, gene family expansion and inference of selection in relation to several other mammals."

Results and Discussion:
Comment 4: Line 127: Consider changing "a gap-free genome" to a plural form.
Response: Thank you very much for your suggestion. We have changed "a gap-free genome" to "gap-free genomes".

Comment 5: Line 132: Thank you for choosing to explain the Merqury scores. However, I'm missing the sentences: "These values indicated the superior quality of our assemblies. It is essential to emphasize that Merqury quality values span from 0 to 255, with higher values denoting superior quality." They were mentioned in the response to the reviewer but not in the manuscript. Is there a reason for this?
Response: We apologize for the mistake. The sentences "These values indicated the superior quality of our assemblies. It is essential to emphasize that Merqury quality values span from 0 to 255, with higher values denoting superior quality." have been included at lines 131-133 in the revised MS.

Comment 6: Line 220-221: A citation is missing for this statement.
Response: We are sorry for the mistake. We have added a citation for this statement.
Ref:
Brar, G, Amon, A. Emerging roles for centromeres in meiosis I chromosome segregation. Nat Rev Genet 2008;9(12):899-910.

Comment 7: Line 283: Although the authors probably mentioned the paper somewhere already, I would suggest adding a citation for this first subspecies classification here.
Response: Thank you for your suggestion. We have added a citation for this first subspecies classification here.
Ref:
Zhou, X, Guang, X, Sun, D, et al. Population genomics of finless porpoises reveal an incipient cetacean species adapted to freshwater. Nat Commun 2018;9(1):1276.

Comment 8: Line 312: The genus name of Lipotes vexillifer is already shortened here to a "L." although its not written out beforehand in the manuscript. I would suggest writing out the name here and shorten it at line 329.
Response: Thank you very much for your suggestion. We have written out "Lipotes vexillifer" at line 283, and shorten it as "L. vexillifer" at line 300 in the new revised MS. In addition, we have abbreviated "N. a. asiaeorientalis" and "N. a. sunameri" at line 296-298, as well as "B. mysticetus", "B. acutorostrata", "S. chinensis" and "T. truncates" at line 453-454 since they were previously mentioned in the manuscript.

Methods:
Comment 9: Line 444: I appreciate that the authors clarified the RepBase usage. However, for this workflow here to be reproduceable, you should usually provide the version number of RepBase and/or the date when the database was retrieved. Consider adding this information to ensure reproducibility.
Response: Thank you very much for your suggestion. We have provided the version number of RepBase database (v21.12) at line 401 in the revised MS.

Comment 10: Line 467: Consider changing the capital to a lower case "I" in "Information".
Response: We are sorry for the mistake. We have changed "Genome comparison and Identification of newly assembled genes" to "Genome comparison and identification of newly assembled genes".

Comment 11: Line 469: described where? Please add details to this statement.
Response: We are sorry for the unclear description. We have added a citation for this statement.
Ref:
Li, T, Xu, X, Zhao, J, et al. Genome assembly of KA105, a new resource for maize molecular breeding and genomic research. The Crop Journal 2023;11(6):1793-1804.

Comment 12: Line 499+501: I'm not sure if the phrases "a widely utilized tool for multiple sequence alignment" and "a commonly employed software tool for phylogenetic analysis" are the result of some corrections meant to answer suggestions made by reviewer 1, but for me, these phrases sound unnecessary and strange.
Response: We are sorry for the unclear description. We have changed "Single-copy orthologous genes (1:1:1) were aligned using MAFFT (v7.310), a widely utilized tool for multiple sequence alignment. Subsequently, a maximum-likelihood phylogenetic tree was generated using PhyML (v3.3), a commonly employed software tool for phylogenetic analysis." to "We utilized MAFFT (v7.310) and PhyML (v3.3) software tools to align single-copy orthologous genes (1:1:1) and construct a maximum-likelihood phylogenetic tree, respectively."

Conclusion:
Comment 13: Line 569: Here the authors clearly state that these genomes are probably the "most continuous" genomes of cetacean thus far. Again, for this statement to be justified, I strongly recommend adding numbers and citations to this claim somewhere in the discussion.
Response: Thank you very much for your suggestion. We have addressed the only general issue raised in comment 1. In addition, we have added the sentence in the Conclusion section: Compared to the 34 cetacean genomes sourced from public databases, the two genomes newly assembled in our study exhibit the longest contig N50 and scaffold N50, along with the lowest number of contigs, thus achieving superior continuity.

Figures:
Comment 14: Figure 5 (Phylogeny): Although beautiful, I'm very sure that the time axis in the bottom of the graph won't be readable when printed out or implemented within a PDF version of the article. This axis is just too small.
Response: Thank you for your feedback. We have increased the size of the time axis at the bottom of the graph to ensure it is readable when printed or viewed in the PDF version of the article.
