# Supplementary material for: Telomere-to-telomere gap-free genome assembly of the endangered Yangtze finless porpoise and East Asian finless porpoise
Source: Gigascience. 2024 Sep 16;13:giae067. doi: 10.1093/gigascience/giae067 (PMC11403816; doi:10.1093/gigascience/giae067)
Supplement: giae067_Response_to_Reviewer_Comments_Revision_2 [file giae067_response_to_reviewer_comments_revision_2.pdf]

Author's Response To Reviewer Comments

Dear Dr. Hans Zauner,
Thanks for your kind consideration and comments regarding to our manuscript entitled “Telomere-to-telomere gap-free genome assembly of the endangered Yangtze finless porpoise and East Asian finless porpoise” (GIGA-D-23-00359R2). We wish to publish the article in the "Research" section, and have included more details from the actual research findings in the abstract. We hope the revised manuscript have been improved and meet the journal style. Additionally, we would like to express our sincere gratitude to the reviewer #2. We look forward to hearing from GigaScience soon.

Sincerely yours,

Correspondence: Kai Liu
Freshwater Fisheries Research Center, Chinese Academy of Fishery Sciences, Wuxi 214081, China.
