# Supplementary material for: Telomere-to-telomere gap-free genome assembly of the endangered Yangtze finless porpoise and East Asian finless porpoise
Source: Gigascience. 2024 Sep 16;13:giae067. doi: 10.1093/gigascience/giae067 (PMC11403816; doi:10.1093/gigascience/giae067)
Supplement: giae067_Reviewer_1_Report_Original_Submission — Phillip A Morin, Ph.D. -- 1/10/2024 [file giae067_reviewer_1_report_original_submission.pdf]

Reviewer Report

Title: Telomere-to-telomere gap-free genome assembly of the endangered Yangtze finless porpoise and East Asian finless porpoise

Version: Original Submission **Date:** 1/10/2024

Reviewer name: Phillip A Morin, Ph.D.

Reviewer Comments to Author:

General comments:
The authors have assembled new telomer-to-telomer reference genomes for two finless porpoise species, the fresh-water Yangtze finless porpoise and the oceanic East Asian finless porpoise. These genome assemblies appear to be ultra-high quality, with substantial description of the genomic features (repeats, genes, assembly characteristics), improvements over previous assemblies for the same species, and comparison of the genomes for some features including genomic structure, variation, novel genes and genes under selection. These represent substantial contributions to cetacean genomics and to understanding the evolution of these two sister species in their unique habitats.
The manuscript requires substantial revision to provide adequate methods for the reader to understand how the genomes were assembled and analyzed, and the figures need to be modified substantially to be useful. As they are, the legends do not adequately describe what's in many of the figures, the figures often try to present too much information in a single plot or set of plots, and the text is often too small to read. In my detailed comments below, I suggest ways to modify the figures and legends to make them more informative and useful.
The manuscript is generally well written, but requires some editing for standard English, and several sections repeat information presented in tables and figures, and could be edited to reduce length and focus on interpretation rather than just repetition. In particular, although the improvements in the new genome assemblies are substantial compared to previous assemblies, these improvements are a normal process in genome assembly, and not of great biological importance. I suggest reducing description and discussion of the upgrades to the genomes, and focus on the interesting characteristics of, and comparisons between, the two new genomes.
Specific comments (by line number)
28. change 'the chromosome both ends' to 'the chromosomes at both ends'
31. 'selection pressure' was not studied, only inference of selection.
33. The accession ID given for YFP, GCF000442215, is for Lipotes vexillifer, not for the YFP genome.
43. is there a word missing between 'clearly' and 'dorsal fin? Or, change from 'a clearly dorsal fin' to 'an obvious dorsal fin'
54. It's not clear what you mean by 'populations', since the comparisons are among species and subspecies. The term 'population' means something different from species and subspecies, so this section should be revised to use the appropriate terms.
67. Insert 'a' before 'high-quality genome'
69. change 'cetacean' to 'cetaceans'

70. delete 'which was'
71. The number of scaffolds for the previous version of the genome is misleading. The number given, 104, is based only scaffolds that are larger than the N50 length. According to the supplemental materials for Zhou et al. 2018, there were actually 97,387 scaffolds >100bp, 2179 scaffolds >2kb, and 423 scaffolds larger than the N90 size. If not all scaffolds are going to be counted, then it's important to specify which scaffolds were counted.
80. change to 'a hotspot of genomic research, with extensive applications…'
81. insert 'and' between 'chicken' and 'fish'.
99. change 'Based on' to 'In addition to'
116. What do the Merqury quality values mean? Are these from a range (e.g., 1-100), or do they represent a log value? The reader can't interpret this without some context, and you can't assume they are familiar with every program.
133-156. The whole section on gene prediction and annotation results could be reduced so that it repeats less of what is already presented in Table 1. Simple summary values such as average length of coding sequences don't need to be presented in the results, especially if they are not discussed in the discussion section as being important.
138. The sentence starting 'For gene content assessment…' should be in the methods, not results. It's also not clear what you mean by '8 homologous proteins' (proteins, or databases, or ???). Also, change 'RNAseq data' to 'RNAseq datasets' (as in line 156).
143-145. The sentence starting with 'The protein-coding genes in…' is difficult to understand, and I'm not sure it's important. Does it belong in the methods, or does it say something about the quality of the gene identification?
147. The wrong accession ID is given for the YFP, and I don't understand why the accession numbers are being presented here at all, since this whole section is about the new YFP and EFP genomes.
155. 'Finally, 20, 589 (88.98%) and 20, 613 (89.23%)' of what? Genes? It's unclear what you are referring to for these numbers.
162. What are 'repeat monomers'? is this a particular sequence that is unique to centromere repeats? One more sentence to describe what you are using to infer centromeres would be useful.
165. Does 'newly identified centromere regions' refer to regions that are newly discovered in centromeres, or newly discovered in the new genome assemblies (compared to the old ones)? The sentence is a little confusing.
179-180. please explain what 'stop-gain SNPs' and 'stop-loss SNPs' are. I think this means SNPs that result in the gain or loss of a stop codon, but I don't know the specific term (jargon).
183. Why did you identify these 5 pathways from Figure 4C? Were they the only ones with functional variants, or are they just random examples from all of the ones in Figure 4C?
186. Delete 'Here', and start the sentence with 'The genes coding…'
190. change 'will' to 'would be expected to'
192-193. Delete the last sentence of the paragraph, as it is just repeating what is said above.
194. Delete 'Specifically,' and start the sentence with 'By comparing…' .
195. change 'YFP assembled 5480 new genes, while EFP assembled 1453…' to 'The YFP assembly included 5480 new genes, while the EFP assembly included 1453…'
198. Change 'discovered' to 'indicated'

203. Change 'including' to 'include'
209. Insert 'structural' or 'chromosomal' before 'conservation'
209-214. Figure 1B should be its own figure, and changed to include only the synteny information. It's too small and too dense to be useful as it is. The full figure can be presented in supplemental materials. The text describing the figure could be expanded to describe what was found rather than just general observations of 'similar patterns' gene frequency, density, etc., or it could be reduced to just focus on what was different (specific chromosome rearrangements).
218. What do you mean by the 'last common ancestor'? This 'ancestor' doesn't exist, so is it a reconstructed ancestral genome?
220. What does 'involved with statistical significance' mean?
232-234. The first time these species are mentioned, the Latin name needs to be included. In the methods, only the Latin names are used, so the reader can't easily know what species the common names refer to (e.g. the Chinese white dolphin is called the Indo-Pacific humpback dolphin in the Society for Marine Mammalogy's taxonomy list).
237-239. What evidence is there for the statement that 'The evolution of DNA damage repair pathways implied the existence of additional triggers for genomic instability in the Yangtze River, including human activities such as wading projects, dredging and quarrying'? It seems odd to invoke pressure from the last century or less for evolutionary changes at the gene pathway level.
256-257. The 'QIAGEN Blood & Cell Culture DNA Midi Kit' appears to be a DNA extraction kit, not a library preparation kit, so it cannot have been used to construct PacBio HiFi libraries. This section needs more detail to specify exactly what kits or methods were used to construct libraries, as the library methods impact downstream DNA sequence processing.
261. Cite a reference for the 'CTAB method'
267. What filters were used to 'filter the Hi-C reads? Are there specific quality filters, or read trimming that are needed?
268. What do you mean by 'refine' the ONT reads? This is a meaningless term, and since the Perl scripts are described as 'self-designed', this provides no information about what was done. Likewise, the statement that 'ONT reads were subsequently corrected' is also too vague. What was actually done to 'correct' the reads?
272. There are a lot of programs used to process data, and while it's good that the actual parameters are given for some of them, it would be useful to also state whether the parameters were the default parameters or modified for these data, and describe what the parameter do (e.g., what does '-j 80 -s 80 -a 30' do?)
275. What do you mean by 'bridge the gaps in the gapless PacBio assembly? Do you mean that ONT reads and contigs were used to bridge gaps in the contig assembly in order to generate the gapless scaffolds?
281. Change 'were' to 'was'
283-4. insert 'the' before 'quartet pipeline' and change 'region' to 'regions'.
287. Change 'conducted' to 'used'
294. If RNAseq data were mapped to both assemblies, then change 'assembly' to 'assemblies'.
299. I think 'combing' should be changed to 'combining'
320. Change 'Consistent phylogeny with previous study' to 'A phylogeny consistent with a previous

study'
327. Change 'Blast' to 'BLAST'
328. It's unclear what you mean by 'defined syntenic blocks by MCscanX'. Do mean that 'syntenic blocks were defined using MCscanX'?
332. delete 'branch of', or change to 'analysis in the finless porpoise clade'.
336. should the meaning of P-valueâ‰¤0.01 be 'significantly expanded and contracted gene families', or is it either expanded OR contracted 'significantly expanded or contracted gene families'?
338. Change 'family' to 'families'
339. Change the section title to 'Gene positive selection analysis'
343. What filtering of alignments was done with Gblocks?
345. delete the comma after 'branch'
350. 'quality controlled' is vague. What was done to the reads to make them 'clean'? Please include the parameters or steps, e.g., reads were trimmed to remove adapter sequences and base calls with quality <30 (or whatever your parameters were).
357. change 'assembly' to 'assemblies'
359. delete 'two' (not needed because you name the species genomes in the same sentence)
372. What is 'undesired recombination'? Undesired by whom? I think you can just delete that word, and say the genome data can serve as a valuable resources for understanding… recombination, etc.
373. change 'Overall, this is the most continuous genome assembly to date' to 'Overall, these are the most continuous cetacean genome assemblies to date'
375. change 'deepen the scientific issues' to 'deepen the scientific understanding of issues'
387. Authors' contributions: The initials vary between 2 and 3 initials to represent authors' names, but the names in the authors list are all only two names, so there should be only 2 initials for each.
Table 1. In the legend, provide the accession ID's for each genome version in the note below the table. It's not clear why there is "C:" in front of each percentage for the two BUSCO lines. I think it would be clearer to remove "C:" from each number, put an asterisk (*) after BUSCO, and in the note, write "* percentage of complete BUSCO evaluation".
Figures.
Figure 1 contains too many unrelated figures, and too dense information, some of which is never discussed in the paper. I suggest that Figure 1 should include only A and F, B should be its own figure, and C,D,E should be moved to supplemental materials.
For the new figure (B), only the rings A (chromosome length) and F (syntenic blocks) should be included, as the others are too tiny and dense to be useful to the reader. Those can be put into supplemental materials with better descriptions to allow the reader to see the detail and to explain the colors and scale. All colors within the figures need to be explained or include a color legend.
Figure 2. There is no visible "ruler" in the figure (but described in the legend).
The legends for A and B repeat most of the information. They can be combined to reduce repetition, e.g.,
Structure of T2T and gap-free chromosomes in A) Yangtze finless porpoises v2.0, and B) East Asian finless porpoises (v2.0). All 21+Xchromosomes are drawn to scale and the ruler indicates chromosome length. Triangles indicate the presence of telomere sequence repeats. Circles represent the locations of centromeric regions. The gap positions in the v1.0 genome assemblies are marked with squares to the

right of the chromosome in the v2.0 genome assemblies.
Figure 3. A and B are described as 'Heatmaps', but they appear to be box plots, not heatmaps. They are only described in one sentence in the text, so I suggest that they could be moved to supplemental materials, and include only C and D in the figure.
In C and D, the legend says "GO enrichment analysis of genes", but the figure is not showing analysis, but results. Please describe the plot and results, e.g., number of genes in centromere regions associated with GO pathways" (that may not be correct, but I'm not sure what the figure is showing, so it needs to be described better).
Figure 4 D and E could be added to Figure 3 to show all of the same types of plots in one figure instead of splitting data into different figures.
Figure 4.
A. what do the numbers next to the color legend represent? Is that the maximum number of SNPs per MB window? Is each color a range of values?
C. I don't understand what you mean by genes located in the Indel region or in the SNP region. What are the Indel and SNP 'regions'? These terms have not been used anywhere else or defined.
D-E. Consider moving to Figure 3. Are these 'new-found' genes relative to the old (V1) versions of the genomes, or genes that are unique to each species?
Figure 5.
A. "Ma: Million years ago" can be deleted, as "Ma" is not used in the figure.
B. what are the units for the scale? Is this a count of gene families?
C-D. define what "Rich Factor" means. What do the colors mean?
E. There was no description of E, and it is too small to read. I suggest moving it to supplemental materials to allow it to be described in detail and large enough for the reader to see if interested.

Methods

Are the methods appropriate to the aims of the study, are they well described, and are necessary controls included? Choose an item.

Conclusions

Are the conclusions adequately supported by the data shown? Choose an item.

Reporting Standards

Does the manuscript adhere to the journal's guidelines on [minimum standards of reporting?] Choose an item.

Choose an item.

Statistics

Are you able to assess all statistics in the manuscript, including the appropriateness of statistical tests used? Choose an item.

Quality of Written English

Please indicate the quality of language in the manuscript: Choose an item.

Declaration of Competing Interests

Please complete a declaration of competing interests, considering the following questions:

- Have you in the past five years received reimbursements, fees, funding, or salary from an organisation that may in any way gain or lose financially from the publication of this manuscript, either now or in the future?
- Do you hold any stocks or shares in an organisation that may in any way gain or lose financially from the publication of this manuscript, either now or in the future?
- Do you hold or are you currently applying for any patents relating to the content of the manuscript?
- Have you received reimbursements, fees, funding, or salary from an organization that holds or has applied for patents relating to the content of the manuscript?
- Do you have any other financial competing interests?
- Do you have any non-financial competing interests in relation to this paper?

If you can answer no to all of the above, write 'I declare that I have no competing interests' below. If your reply is yes to any, please give details below.

I declare that I have no competing interests.

I agree to the open peer review policy of the journal. I understand that my name will be included on my report to the authors and, if the manuscript is accepted for publication, my named report including any attachments I upload will be posted on the website along with the authors' responses. I agree for my report to be made available under an Open Access Creative Commons CC-BY license (http://creativecommons.org/licenses/by/4.0/). I understand that any comments which I do not wish to be included in my named report can be included as confidential comments to the editors, which will not be published.

Choose an item.

To further support our reviewers, we have joined with Publons, where you can gain additional credit to further highlight your hard work (see: https://publons.com/journal/530/gigascience). On publication of this paper, your review will be automatically added to Publons, you can then choose whether or not to claim your Publons credit. I understand this statement.

Yes Choose an item.
