# Supplementary material for: Telomere-to-telomere gap-free genome assembly of the endangered Yangtze finless porpoise and East Asian finless porpoise
Source: Gigascience. 2024 Sep 16;13:giae067. doi: 10.1093/gigascience/giae067 (PMC11403816; doi:10.1093/gigascience/giae067)
Supplement: giae067_Reviewer_2_Report_Original_Submission — Magnus Wolf -- 1/11/2024 [file giae067_reviewer_2_report_original_submission.pdf]

Reviewer Report

Title: Telomere-to-telomere gap-free genome assembly of the endangered Yangtze finless porpoise and East Asian finless porpoise

Version: Original Submission **Date:** 1/11/2024

Reviewer name: Magnus Wolf

Reviewer Comments to Author:

Review of: Telomere-to-telomere gap-free genome assembly of the endangered Yangtze finless porpoise and East Asian finless porpoise.

In this work, Yin et al. present two newly assembled genomes of two narrow-ridged finless porpoise (Neophocaena asiaeorientalis) subspecies (Yangtze, YFP and East Asia, EFP) as well as a comprehensive report on its quality and differences in terms of genotype changes related to their different habitats. I overall enjoyed reading this manuscript, I did not find too many downfalls in their methods, and I think these two genomes are of the highest quality and one of if not the most continuous reference genomes for cetaceans published thus far. Apart of this, however, I found several downfalls of the manuscript text itself which leads me to conclude that a major revision is needed for this manuscript to be considered for publication in GigaScience. I will first talk about general issues, point out the few downfalls within their methods and then provide a more detailed list of text issues which may help the authors to revise the manuscript.

General issues:

1.) The text of the manuscript has multiple grammatical errors and I invite the authors to revise the language of the manuscript critically and carefully. I will list the errors I found down below but will not guarantee that I found everything. Furthermore, many statements made by the authors come without a proper citation which I will also highlight down below.

2.) The "Results and Discussion" part is missing a discussion. The authors invest the vast majority of this part describing their results and do not attempt to contextualize their results with recent literature. For example, the authors could have compared the quality of these reference genomes and annotations to other cetacean genomes to emphasize their superior quality. Furthermore, the authors did find, through various analyses, genes that might have played a role in their adaptions to different habitats but do not describe what is known about these genes and gene categories and do not describe how these gene might have help in their adaption. Also, the few sentences that do mention these possibilities come without proper citations, again.

3.) The material and method section is missing details. I assumed to find them in some sort of supplementary methods or data repository but failed to find them. If these details are indeed missing, I strongly suggest to provide more details in one of both ways (supplementary methods or within a data repository). I'll list the missing parts down below. Furthermore, the data repository made by the authors on figshare does lack a proper commentary README file and interested scientists are left to figure out the content of these files by themselves. I would strongly recommend providing an extensive README file for this repository to assure reproducibility.

Methods:
1.) One part that is entirely missing in the Methods is the description of how SNPs were generated. Hence, I cannot evaluate the quality of their calls. I highly recommend adding the respective description because otherwise, these steps are not reproducible.
2.) The authors use gene enrichment analyses multiple times but do not mention any false discovery rate correction. If this was not done, I highly recommend repeating these analyses with corrected p-values. If it was done, this information needs to be added to the manuscript. I further failed to find what was used as a reference gene set to compare the enrichment to. This is another information that needs to be provided to ensure reproducibility.
List:
In the following, I'll list the more specific issues I found within the manuscript which may help the authors to revise their manuscript:
Introduction:
Line 43-46: This sentence implies the results of a study, but no citation is provided.
Line 53: I think the "urgent priority for scientists" needs to be more justified. Consider toning this down or being more specific.
Line 57-58: This sentence implies the results of a study, but no citation is provided.
Line 67: "Availability of a high-quality genome assembly."
Line 79-81: This is not a sentence.
Line 84: I would suggest to either make the word "genome" plural or add an "a" at the beginning of the sentence.
Line 86-91: Consider changing these sentences to present.
Line 92-93: This statement is extremely vague. Consider being more specific about what we can learn from their genomes.
Results and Discussion:
Line 99: "Based on" doesn't make sense when speaking about additional sequencing efforts.
Line 104-105: How can scaffold and contig statistics be completely identical?
Line 116-118: In this sentence is a verb missing. Also, consider explaining the mercury score in one sentence since this is something not known by the generally informed reader.
Line 137: You may want to replace "constituting" with "reaching" or "accounting for"
Line 139-143: You have an excessive use of the word "respectively" in this paragraph.
Line 141-142: Consider maxing the word "exon" and "intron" plural.
Line 145: Consider turning the "was" into present.
Line 159: Consider rephrasing "mystery" to something more objective.
Line 163-165: It is not directly clear what the difference between "candidate centromere region" and "newly identified centromere regions" is. Consider adding a sentence to explain these differences.
Line 188: It is not clear what the authors meant with "microbial category". Please add further explanation to this.
Line: 188-193: These sentences imply the results of a study, but a citation is missing. The paragraph is also very vague. Is there something known about different microbial stressors in sea- and freshwater?
Line 195: I don't think it is correct to say that a subspecies "assembled" new genes.
Line 203: Consider changing "including" to: "which include 594 species-specific genes"

Line 205-206: You jump from the alignment of genes to the phylogenetic dating without mentioning the phylogenetic tree itself. I would add a sentence between these two to briefly describe the tree you made before talking about the specific dates you received for the emergence of the two subspecies.
Line 209: I do not think it is correct to call one of both subspecies more conserved than the other. Figure 1B is a synteny comparison between both genomes and without an outgroup it is not possible to infer the ancestral state of both possible rearrangements. If a comparison between other more distantly related genomes happened, I would mention them here as well, otherwise, I would remove this statement and directly talk about the possible rearrangements.
Line 212-214: My first suspicion reading this sentence was that the chromosomes simply weren't fully assembled in the EFP genome. But comparing this sentence with Figure 1B, I think the authors intended to say that parts of YFP chromosomes matched parts of multiple chromosomes within the EFP genome. If so, please consider rephrasing this sentence to avoid this confusion.
Line 229-230: Here is a point where the authors should definitely try to contextualize their findings with recent literature. Are these functions that are convergently evolved by other species adapting to freshwater?
Line 239: The authors definitely need to elaborate what might specifically cause these genomic instabilities. Is their something known about specific components and how they function as mutagens?
Line 240: Why are these PSGs "interesting"? Again, no context is given.
Methods:
Line 252: I would invite the authors to provide more details of why no ethical considerations were taken into account. I'm also not sure if this is in conflict with GigaScience's own ethical guidelines. But I would leave this decision to the Editor and the Journal.
Line 259: I cannot find which library kit was used for the Hi-C library.
Line 268: The code for the self-designed Perl program is not provided in any repository and it is not described how the ONT reads were refined in this step.
Line 272-274: This is not a sentence. A verb is missing.
Line 281: The authors chose the OrthoDB for mammals (not stated here but in the results) and I wonder why they did not use the Cetacea specific one. Just a minor comment.
Line 286-291: The homology-based repeat annotation was not based on any databases like RepBase? What homologous information was used? Please specify.
Line 301: How is "high quality genes" defined? No information is given.
Line 305-315: The paragraph "Genome comparison" and "Identification of new assembled genes" is extremely similarly phrased. Would it be possible to merge them into one, shorter paragraph to avoid redundancy?
Line 320: Was there no model testing done? Please specify. Also, there is no information given of how many bootstrap replications were conducted. I guess 1000 but this information is not given.
Line 337-338: Gene enrichment analysis usually requires some sort of correction for false discovery rates. However, nothing like this was described in the manuscript. If this was done, I would mention it here. Otherwise, I would definitely recommend correcting for this!
Line 339: I guess the word "selection" is missing in this paragraph headline.

Methods

Are the methods appropriate to the aims of the study, are they well described, and are necessary controls included? Choose an item.

Conclusions

Are the conclusions adequately supported by the data shown? Choose an item.

Reporting Standards

Does the manuscript adhere to the journal's guidelines on minimum standards of reporting? Choose an item.

Choose an item.

Statistics

Are you able to assess all statistics in the manuscript, including the appropriateness of statistical tests used? Choose an item.

Quality of Written English

Please indicate the quality of language in the manuscript: Choose an item.

Declaration of Competing Interests

Please complete a declaration of competing interests, considering the following questions:

- Have you in the past five years received reimbursements, fees, funding, or salary from an organisation that may in any way gain or lose financially from the publication of this manuscript, either now or in the future?
- Do you hold any stocks or shares in an organisation that may in any way gain or lose financially from the publication of this manuscript, either now or in the future?
- Do you hold or are you currently applying for any patents relating to the content of the manuscript?
- Have you received reimbursements, fees, funding, or salary from an organization that holds or has applied for patents relating to the content of the manuscript?
- Do you have any other financial competing interests?
- Do you have any non-financial competing interests in relation to this paper?

If you can answer no to all of the above, write 'I declare that I have no competing interests' below. If your reply is yes to any, please give details below.

I declare that I have no competing interests.

I agree to the open peer review policy of the journal. I understand that my name will be included on my report to the authors and, if the manuscript is accepted for publication, my named report including any attachments I upload will be posted on the website along with the authors' responses. I agree for my report to be made available under an Open Access Creative Commons CC-BY license (http://creativecommons.org/licenses/by/4.0/). I understand that any comments which I do not wish to be included in my named report can be included as confidential comments to the editors, which will not be published.

Choose an item.

To further support our reviewers, we have joined with Publons, where you can gain additional credit to further highlight your hard work (see: https://publons.com/journal/530/gigascience). On publication of this paper, your review will be automatically added to Publons, you can then choose whether or not to claim your Publons credit. I understand this statement.

Yes Choose an item.
