# Supplementary material for: Telomere-to-telomere gap-free genome assembly of the endangered Yangtze finless porpoise and East Asian finless porpoise
Source: Gigascience. 2024 Sep 16;13:giae067. doi: 10.1093/gigascience/giae067 (PMC11403816; doi:10.1093/gigascience/giae067)
Supplement: giae067_Reviewer_2_Report_Revision_1 — Magnus Wolf -- 5/21/2024 [file giae067_reviewer_2_report_revision_1.pdf]

Reviewer Report

Title: Telomere-to-telomere gap-free genome assembly of the endangered Yangtze finless porpoise and East Asian finless porpoise

Version: Revision 1 **Date:** 5/21/2024

Reviewer name: Magnus Wolf

Reviewer Comments to Author:

Review (Round 2): Denghua Yin et al.: Telomere-to-telomere gap-free genome assembly of the endangered Yangtze finless porpoise and East Asian finless porpoise

In this manuscript, Denghua Yin and colleagues present two new, high-quality, de novo genome assemblies of two narrow-ridged finless porpoise (Neophocaena asiaeorientalis) subspecies (Yangtze, YFP and East Asia, EFP) as well as a comprehensive report on its quality and analyses that identify molecular differences related to their different habitats (fresh and sea water). This is the second time I'm reading this manuscript and I think the manuscript was substantially improved by the changes made in response to the last review. I still regard these genomes to be of the highest quality and a valuable resource for future studies. The methodological shortcomings which I highlighted last time were completely addressed and the language problems were amended in most cases.

The only general issue that wasn't covered enough since last time is in the discussion where the authors fail to provide more comparisons to assemblies from the literature. The authors mention in the conclusion that these assemblies are potentially the "most continuous" genomes of cetacean today and I tend to believe them. However, the manuscript is lacking a clear comparison to other assemblies to make this statement. I already suggested this in my last review, and I consider this to be crucial, although I'm aware that this conflicts to some extent with the suggestions made by reviewer 1. Hence, I suggest to the authors to add just a brief comparison (2-3 sentences) to the discussion to validate this claim, to provide some contextual statistics and to give the reader an idea about the improved quality of these genomes.

In the following, I'll list some additional suggestions that may improve the manuscript. Given that these suggestions require only minor changes to the text, I recommend a minor revision to the editor.

General issues:

1.) As mentioned already, I suggest adding a small comparative paragraph to the discussion to compare the quality of these genomes to previous genomes of Cetacea.

Abstract:

Line 31: I do not think you can "study comparative genomes". Consider "study structural similarities" instead.

Introduction:

Line 95-98: Same as in line 30.

Line 95-98: Furthermore, the article "the" is used in a confusing way here. Consider changing

the sentence to: "We compare the quality of the newly drafted assemblies with previously available versions and explore synteny blocks, structural similarities, phylogenetic relationships, gene family expansion and inference of selection in relation to several other mammals."

Results and Discussion:

Line 127: Consider changing "a gap-free genome" to a plural form.

Line 132: Thank you for choosing to explain the Merqury scores. However, I'm missing the sentences: "These values indicated the superior quality of our assemblies. It is essential to emphasize that Merqury quality values span from 0 to 255, with higher values denoting superior quality." They were mentioned in the response to the reviewer but not in the manuscript. Is there a reason for this?

Line 220-221: A citation is missing for this statement.

Line 283: Although the authors probably mentioned the paper somewhere already, I would suggest adding a citation for this first subspecies classification here.

Line 312: The genus name of Lipotes vexillifer is already shortened here to a "L." although its not written out beforehand in the manuscript. I would suggest writing out the name here and shorten it at line 329.

Methods:

Line 444: I appreciate that the authors clarified the RepBase usage. However, for this workflow here to be reproduceable, you should usually provide the version number of RepBase and/or the date when the database was retrieved. Consider adding this information to ensure reproducibility.

Line 467: Consider changing the capital to a lower case "I" in "Information".

Line 469: described where? Please add details to this statement.

Line 499+501: I'm not sure if the phrases "a widely utilized tool for multiple sequence alignment" and "a commonly employed software tool for phylogenetic analysis" are the result of some corrections meant to answer suggestions made by reviewer 1, but for me, these phrases sound unnecessary and strange.

Conclusion:

Line 569: Here the authors clearly state that these genomes are probably the "most continuous" genomes of cetacean thus far. Again, for this statement to be justified, I strongly recommend adding numbers and citations to this claim somewhere in the discussion.

Figures:

Figure 5 (Phylogeny): Although beautiful, I'm very sure that the time axis in the bottom of the graph won't be readable when printed out or implemented within a PDF version of the article. This axis is just too small.

Methods

Are the methods appropriate to the aims of the study, are they well described, and are necessary controls included? Choose an item.

Conclusions

Are the conclusions adequately supported by the data shown? Choose an item.

Reporting Standards

Does the manuscript adhere to the journal's guidelines on minimum standards of reporting? Choose an item.

Choose an item.

Statistics

Are you able to assess all statistics in the manuscript, including the appropriateness of statistical tests used? Choose an item.

Quality of Written English

Please indicate the quality of language in the manuscript: Choose an item.

Declaration of Competing Interests

Please complete a declaration of competing interests, considering the following questions:

- Have you in the past five years received reimbursements, fees, funding, or salary from an organisation that may in any way gain or lose financially from the publication of this manuscript, either now or in the future?
- Do you hold any stocks or shares in an organisation that may in any way gain or lose financially from the publication of this manuscript, either now or in the future?
- Do you hold or are you currently applying for any patents relating to the content of the manuscript?
- Have you received reimbursements, fees, funding, or salary from an organization that holds or has applied for patents relating to the content of the manuscript?
- Do you have any other financial competing interests?
- Do you have any non-financial competing interests in relation to this paper?

If you can answer no to all of the above, write 'I declare that I have no competing interests' below. If your reply is yes to any, please give details below.

I declare that I have no competing interests.

I agree to the open peer review policy of the journal. I understand that my name will be included on my report to the authors and, if the manuscript is accepted for publication, my named report including any attachments I upload will be posted on the website along with the authors' responses. I agree for my report to be made available under an Open Access Creative Commons CC-BY license (http://creativecommons.org/licenses/by/4.0/). I understand that any comments which I do not wish to

be included in my named report can be included as confidential comments to the editors, which will not be published.

Choose an item.

To further support our reviewers, we have joined with Publons, where you can gain additional credit to further highlight your hard work (see: https://publons.com/journal/530/gigascience). On publication of this paper, your review will be automatically added to Publons, you can then choose whether or not to claim your Publons credit. I understand this statement.

Yes Choose an item.
